# Supplementary material for: Human cancer-targeted immunity via transgenic hematopoietic stem cell progeny
Source: Nat Commun. 2025 Jul 1;16:5599. doi: 10.1038/s41467-025-60816-z (PMC12219382; doi:10.1038/s41467-025-60816-z)
Supplement: Supplementary file 1 — Supplementary Information [file 41467_2025_60816_MOESM1_ESM.pdf]

Supplemental Information for "Human Cancer-Targeted Immunity via Transgenic Hematopoietic Stem Cell Progeny"

Theodore S. Nowicki[1,2,3,4,5,6], Nataly Naser Al Deen[7], Cole W. Peters[1], Begoña Comin-Anduix[3,8,9], Egmidio Medina[7], Cristina Puig-Saus[2,3,4,9,10], Ignacio Baselga Carretero[7], Paula Kaplan-Lefko[7], Mignonette H. Macabali[7], Ivan Perez Garcilazo[7], Daniel Chen[7], Jia Pang[7], Beata Berent-Maoz[7], Salem Haile[3,6,7], Jonathan Rodriguez[6], Moe Kawakami[1], Conner K. Kidd[1], Ameya Champhekar[7], Giuseppe Carlucci[11], Agustin Vega-Crespo[7], Bartosz Chmielowski[3,7], Arun Singh[7], Noah Federman[1,3,4,6,12], Gary M. Schiller[3,7], Sarah J. Larson[3,7], Martin Allen-Auerbach[6,8], Alexandra M. Klomhaus[13], Jerome Zack[2,7], David Baltimore[14], Lili Yang[2,3,4,5], Donald B. Kohn[1,2], Owen N. Witte[2,3,4,5,6,10], and Antoni Ribas[3,4,6,7,9,10,11]

Author affiliations:

1. Division of Pediatric Hematology-Oncology, Department of Pediatrics, University of California Los Angeles, Los Angeles, California.
2. Department of Microbiology, Immunology, and Molecular Genetics, University of California Los Angeles, Los Angeles, California
3. Jonsson Comprehensive Cancer Center, University of California Los Angeles, Los Angeles, California.
4. Eli and Edythe Broad Center for Regenerative Medicine and Stem Cell Research, University of California Los Angeles, Los Angeles, California.
5. Molecular Biology Institute, University of California Los Angeles, Los Angeles, California
6. David Geffen School of Medicine, University of California Los Angeles, Los Angeles, California
7. Division of Hematology-Oncology, Department of Medicine, University of California Los Angeles, Los Angeles, California.

8. Ahmanson Translational Theranostics Division, Department of Molecular and Medical Pharmacology, University of California Los Angeles, Los Angeles, California.

9. Division of Surgical Oncology, Department of Surgery, University of California Los Angeles, Los Angeles, California.

10. Parker Institute for Cancer Immunotherapy, UCLA, Los Angeles, CA, USA

11. Department of Molecular and Medical Pharmacology, University of California Los Angeles, Los Angeles, California

12. Department of Orthopaedic Surgery, University of California Los Angeles, Los Angeles, California

13. Department of Medicine Statistics Core, David Geffen School of Medicine, University of California Los Angeles, Los Angeles, CA

14. Division of Biology and Biological Engineering, California Institute of Technology, Pasadena, CA, USA.

Corresponding author: Theodore S. Nowicki, M.D., Ph.D.; Jonsson Comprehensive Cancer Center (JCCC) at the University of California Los Angeles (UCLA), 12-159 Factor Building, 10833 Le Conte Avenue, Los Angeles, CA, 90095. Phone: 310-267-5145; Fax: 310-825-2493; Email: tnowicki@mednet.ucla.edu.

Additional supplementary data files provided as separate files include:

- **Supplementary Data 1. Summary of adverse events experienced and their respective attributions.**
- **Supplementary Data 2. Lentivirus (pRRL-NYESOsr39TK_LTR_to_LTR) pRRL_TRCB nucleotide sequence.**
- **Supplementary Data 3. Clinical Trial Protocol**
- **Supplementary Data 4. Retrovirus (pMSGV1_LTR_to_LTR) pMSGV1-retro nucleotide sequence.**
- **Supplementary Data 5. Gene sets used for celltype.l1 (filtered for the most ubiquitously expressed genes across all nuclei to avoid over-calling) from the Azimuth Reference for human PBMC**
- **Supplementary Data 6. The gene sets used for celltype.l2 filtered for the most ubiquitously expressed genes across all nuclei to avoid over-calling) from the Azimuth Reference for human PBMC**

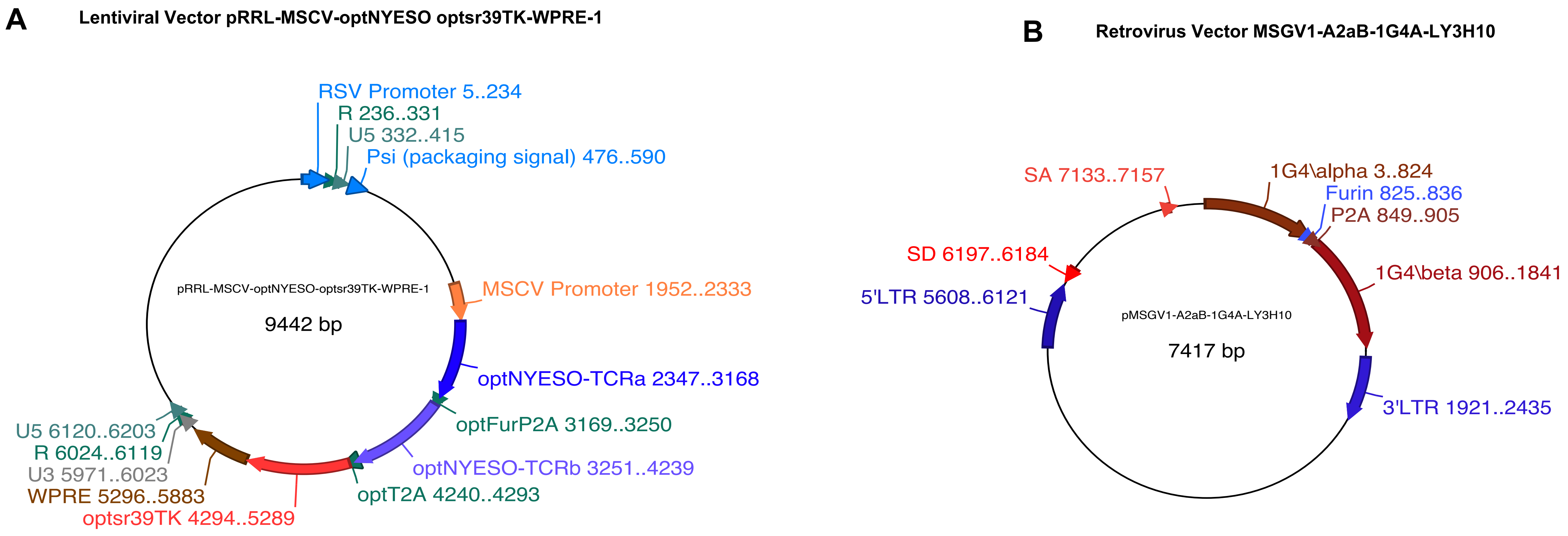

Supplementary Figure 1. Plasmid maps of the lentiviral Vector pRRL-MSCV-optNYESO optsr39TK-WPRE-1 **(A)** and retrovirus Vector MSGV1-A2aB-1G4A-LY3H10 **(B)** used in the manufacturing of PBSC and PBMC cell products, respectively.

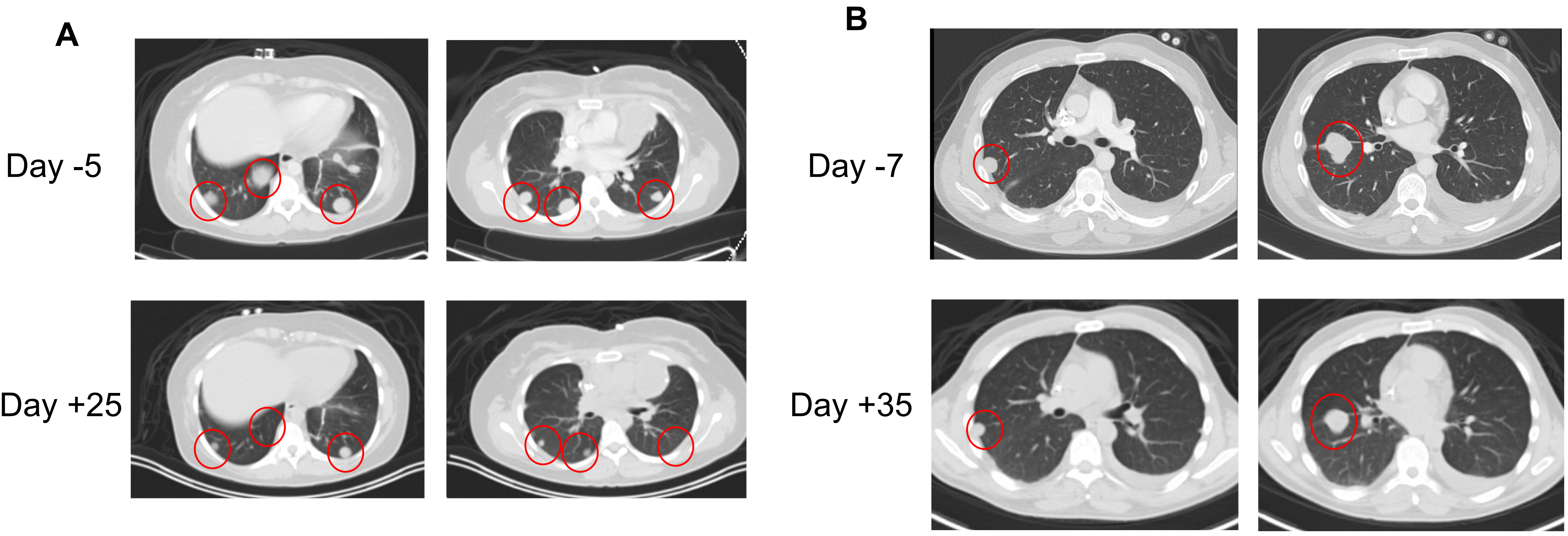

Supplementary Figure 2. Baseline and post-treatment CT scans demonstrating initial reduction in tumor volume in response to therapy for patients NYSCT-03 **(A)** and NYSCT-05 **(B).**

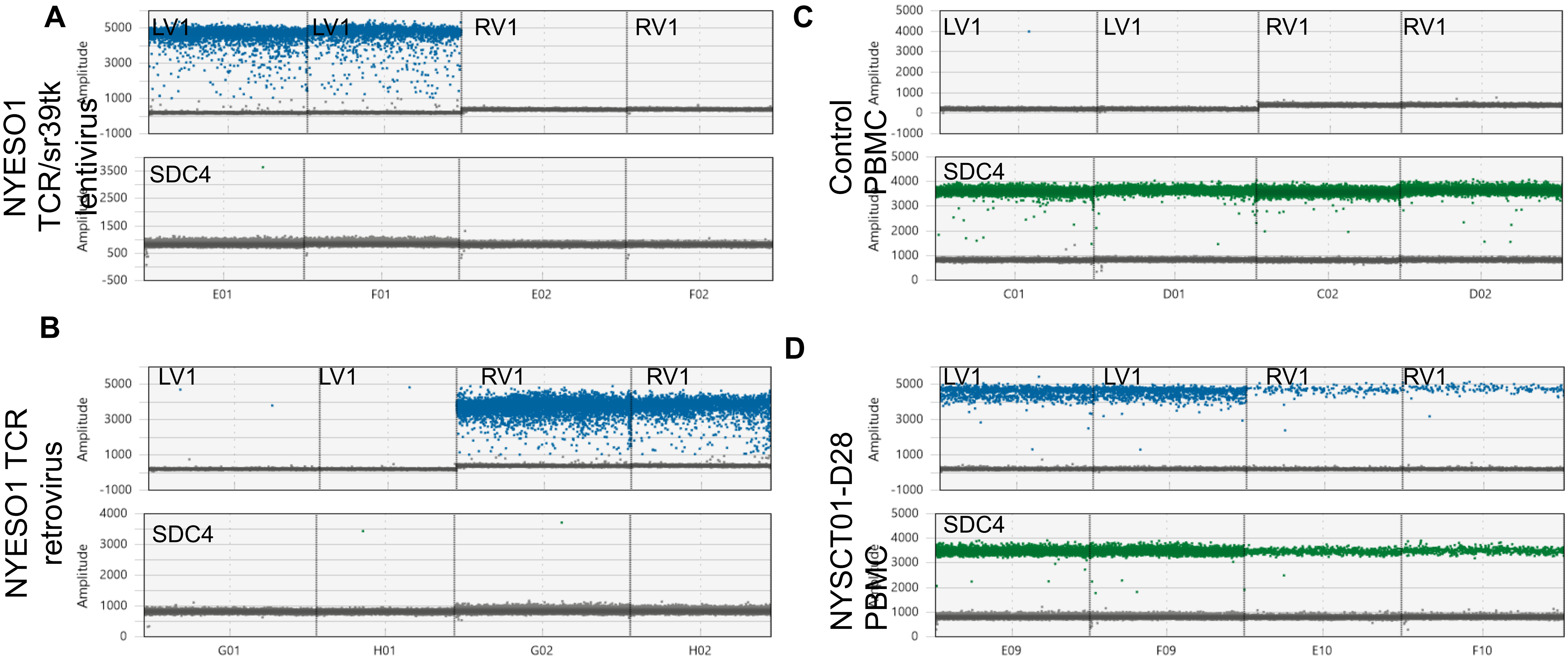

Supplementary Figure 3. ddPCR to distinguish between retroviral and lentiviral vector inserts in PBMCs. Specific primers were designed to recognize either the NY-ESO-1 TCR/sr39TK lentivirus **(A)** or the NY-ESO-1 TCR retrovirus **(B).** When utilized in healthy control PBMCs **(C),** there is no signal detected from lentiviral (LV1) or retroviral (RV1) inserts, but housekeeping gene SDC4 maintains robust signal. PBMCs from NYSCT-01 obtained following engraftment at day +28 **(D)** demonstrate co-existing signal from both the lentiviral and retroviral inserts.

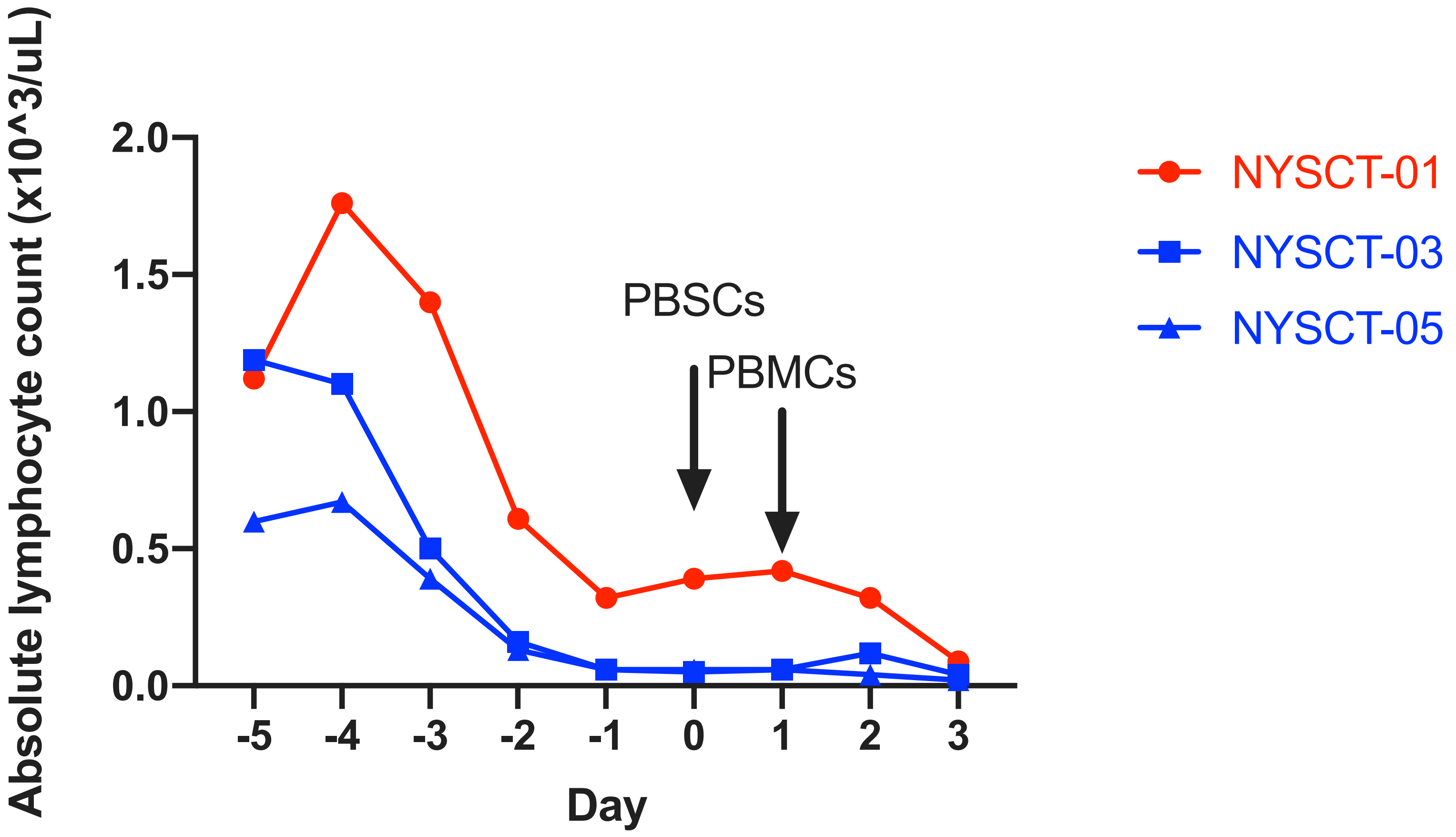

Supplementary Figure 4. Increased fludarabine dosing results in superior lymphodepletion in vivo. While all patients received 70,000-80,000 ng/mL*hour of busulfan over days -5 through -2 as their myeloablative conditioning, fludarabine was used as the sole lymphodepleting conditioning agent. Patient NYSCT-01 received 30mg/m2/day on days -4 and -3, while patients NYSCT-03 and NYSCT-05 received 40mg/m2/day on days -5 through -2. This resulted in superior lymphodepletion (as assessed by daily absolute lymphocyte counts), and was also associated with superior expansion of transgenic TCR-T cells for the latter two patients compared to NYSCT-01 (as shown in **Figures 2A,3A,** and **4A**).

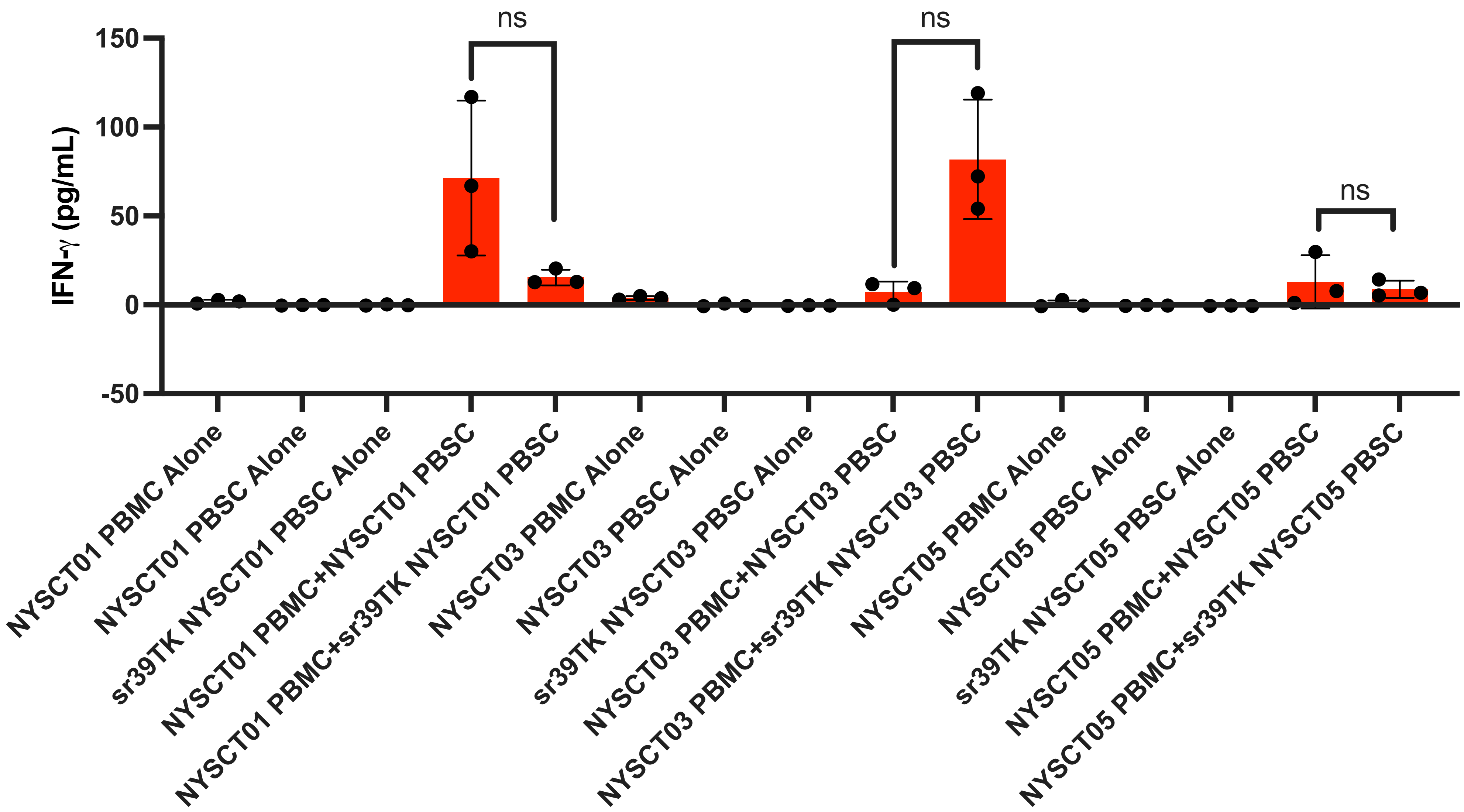

Supplementary Figure 5. No apparent auto/allo-reactivity of subject PBMCs with transduced or untransduced PBSCs. Patient PBMC and PBSC samples were cultured alone, co-cultured together (patient-matched), or co-cultured together following NY-ESO-1 TCR/sr39tk lentiviral transduction, and culture supernatants were subjected to acellular interferon-gamma quantification analysis after 24 hours via ELISA. No significant differences in interferon-gamma secretion levels were observed between conditions.

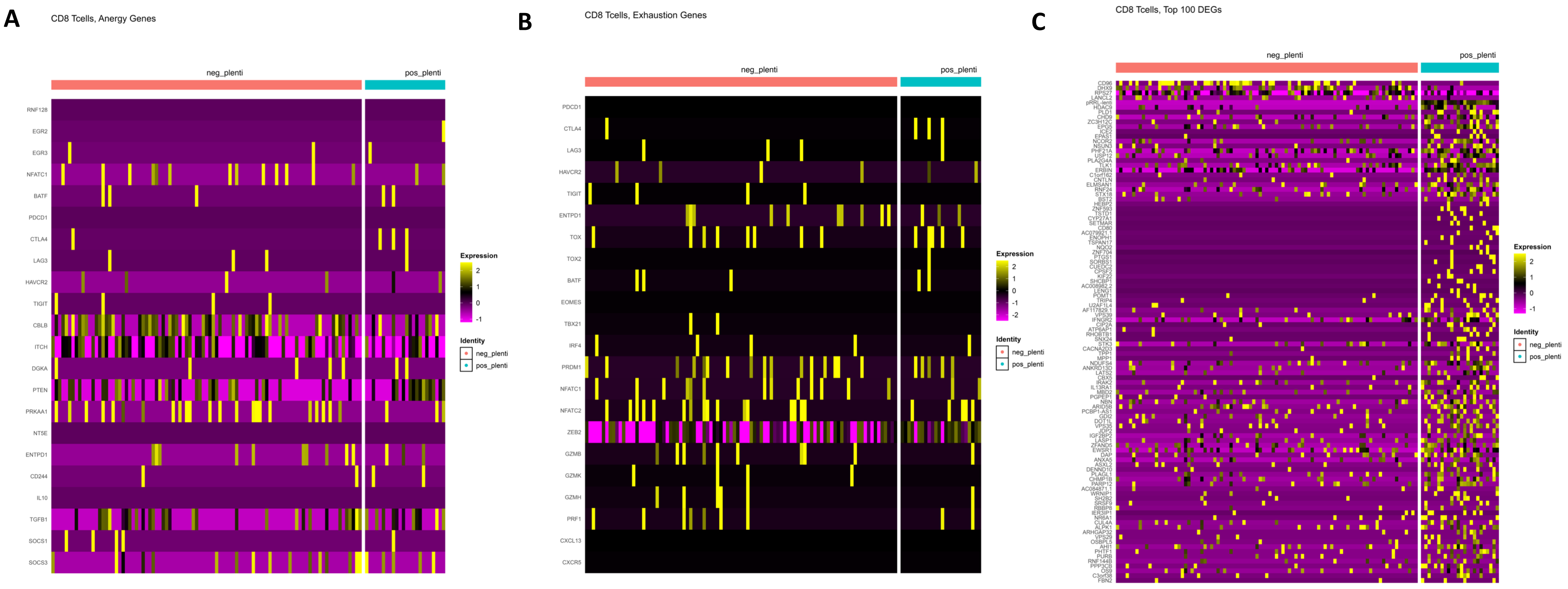

Supplementary Figure 6. No difference in anergy and exhaustion markers detected in the CD8 Tcell sub-population across the pLenti+ and pLenti- cells. A heatmap for the expression of anergy genes **(A)** and exhaustion marker genes **(B)** revealed no apparent difference in the levels of anergy or exhaustion markers in the pLenti+ CD8 T cells as compared to pLenti- CD8 T cells. A heatmap for the top 100 differentially expressed genes between the two CD8 T cell populations **(C)** did not reveal key transcriptional differences between the pLenti+ CD8 T cells as compared to pLenti- CD8 T cells.

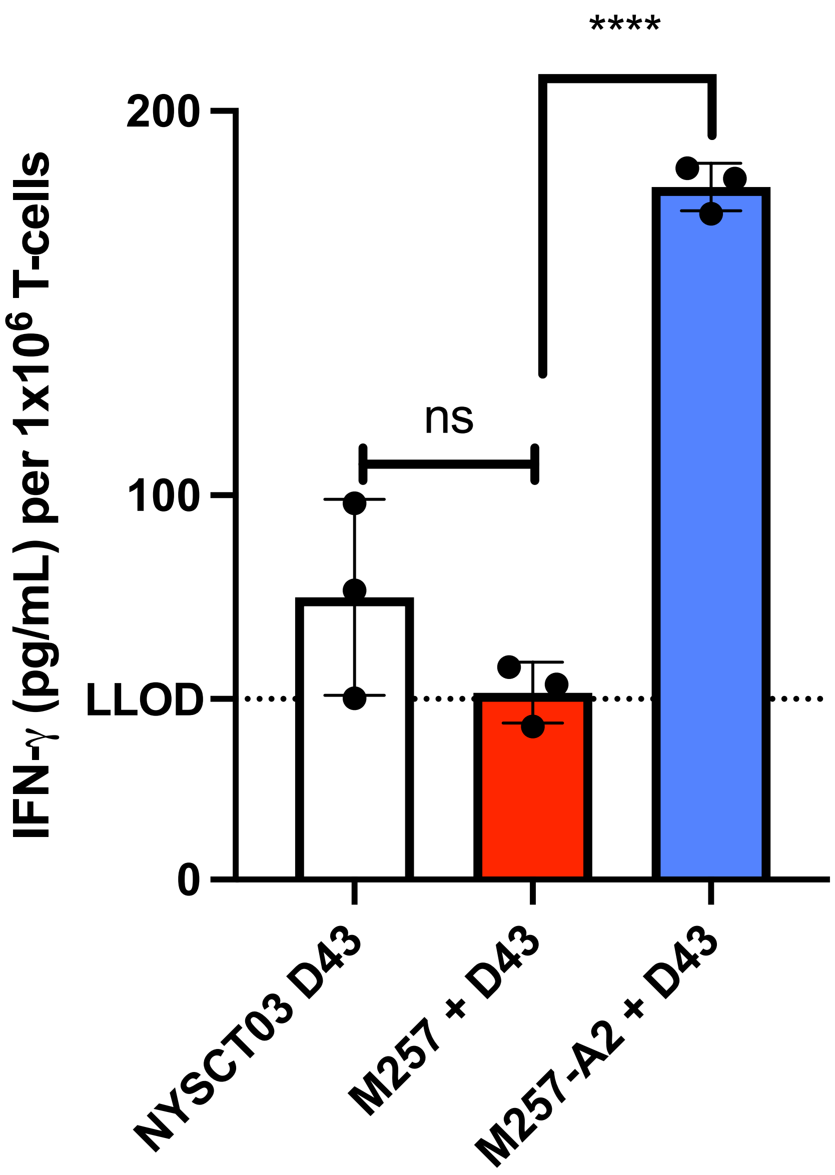

Supplementary Figure 7. Lentiviral HSC-derived TCR-T cells maintain NY-ESO-1 antigen-specific cytokinetic functionality. Day +43 PBMC samples from NYSCT-03 were cultured alone, co-cultured with human melanoma cells M257 (which are positive for NY-ESO-1), or with M257 cells which were transduced to express HLA-A:02:01 (M257-HLA) in triplicate, and culture supernatants were subjected to acellular interferon-gamma quantification analysis after 24 hours via ELISA. While the Day +43 PBMCs alone or co-cultured with M257 cells did not express interferon-gamma above the lower limit of detection (LLOD), co-culture with M257-HLA cells yielded a significant increase in interferon-gamma release. Data from three technical replicates, +/- SD. LLOD = lower limit of detection; **** $p<0.0001$, unpaired t-test.

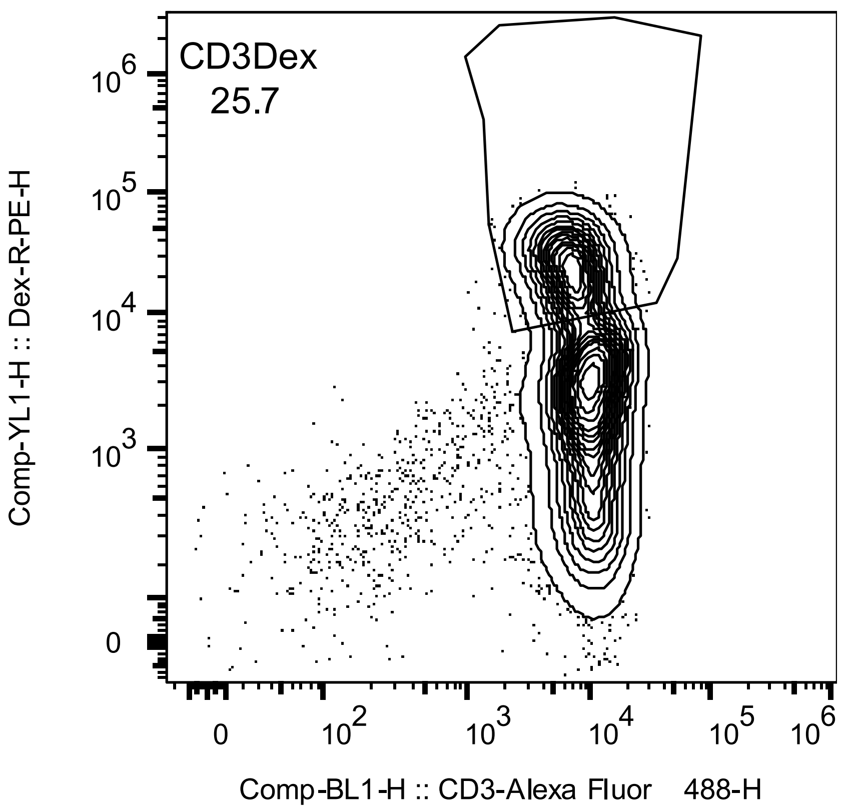

Supplementary Figure 8. Representative flow cytometry plot of MHC dextramer immunologic monitoring.

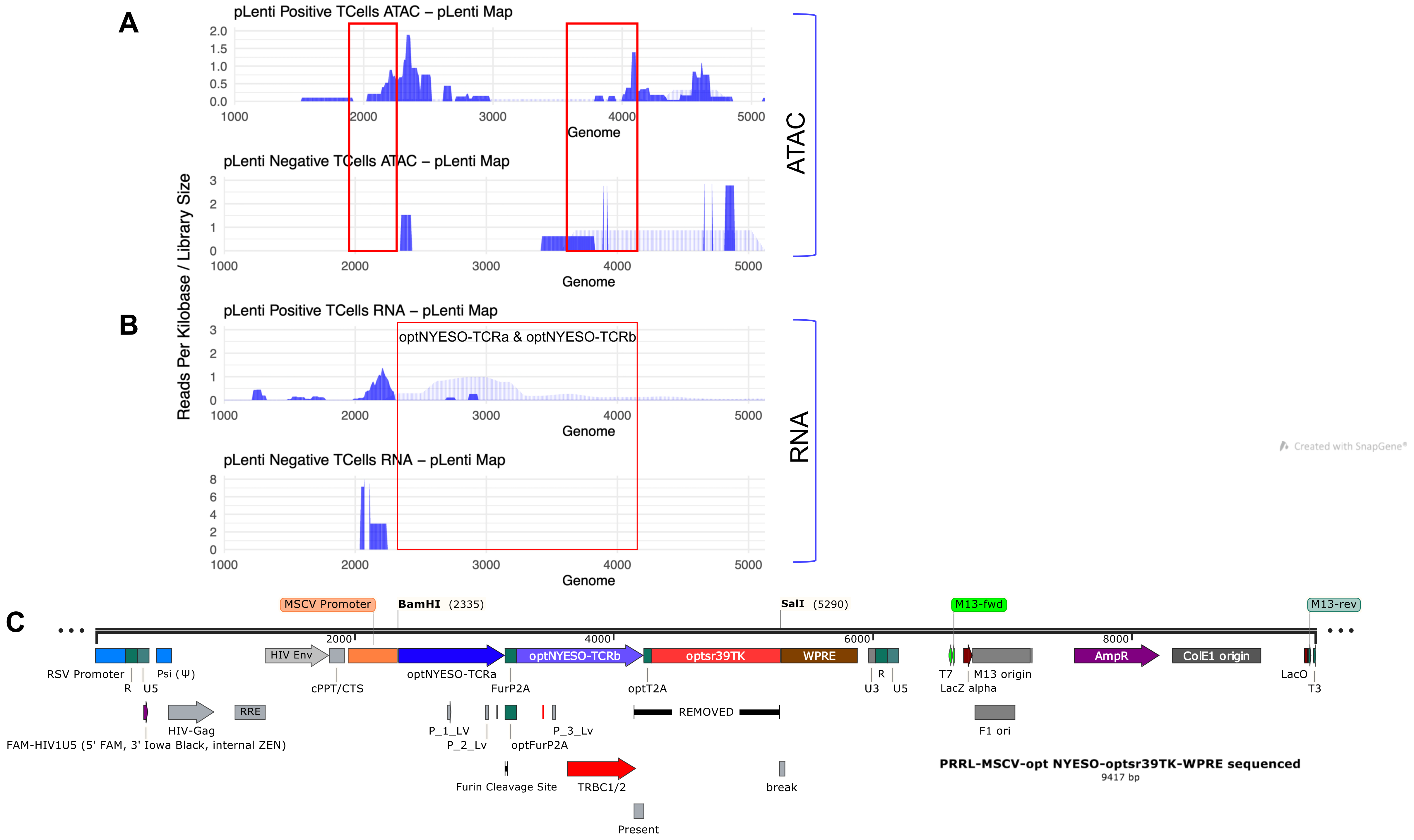

Supplementary Figure 9. ATAC and RNA read peaks at the lentiviral vector promoter and TCR region, respectively, are exclusively found in the pLenti +ve T cell nuclei but not the pLenti -ve T cell ones. ATAC read peaks at the prompter region of the lentiviral vector pRRL-MSCV-optNYESO optsr39TK-WPRE-1 were found only in the pLenti +ve T cell nuclei, and the peaks at the TRBC1/2 genomic map of the optNYESO-TCRb region(≈3700 – 4200) are higher in pLenti +ve T cell nuclei compared to pLenti -ve ones **(A).** Additionally, RNA reads at the optNYESO-TCRa & optNYESO-TCRb and the TRBC1/2 genomic map of the optNYESO-TCRb region are only present in the pLenti +ve T cell nuclei but not the pLenti -ve T cell ones **(B),** as mapped to the genome of the lentiviral vector pRRL-MSCV-optNYESO optsr39TK-WPRE-1 **(C).**

**Patient Study Number**	**No. LV-NYESO TCR/sr39TK PBSC cells infused**	**CD34+ cells/kg infused**	**% CD34+ cell purity**	**% Viability**	**Vector Copy No. (copies/cell)**	**% LV-NYESO/TK+ Colonies by CFU Assay**	**% LV-NYESO/TK+ Colonies by ddPCR**	**Vβ13.1 Expression (% positive cells)**
NYSCT-01	$258.2x10^6$	$3.96x10^6$	99	96	0.47	46	25	15.2
NYSCT-03	$523.380x10^6$	$6x10^6$	99	93	0.55	20	43	26.7
NYSCT-05	$380.47x10^6$	$4.79x10^6$	98	96	0.222	57	17	6.3
Criteria	**$2.5x10^6$/kg**	***$2.5\text{-}6x10^6$***	***≥50%***	***≥70%***	***0.1-2***	***≥10%***	***5-50%***	***5-50%***

Supplementary Table 1. Patient manufacturing outcomes. Lot release criteria are highlighted in the bottom row.

**Patient Study Number**	**No. RV-NYESO TCR PBMC cells infused**	**% Viability**	% NYESO TCR Positive Cells	IFNγ release following co-culture with M257-A2 cells
NYSCT-01	1.0×10^9	94%	69.6%	32,149 pg/mL/ 10^6 cells
NYSCT-03	1.0×10^9	96%	60.4%	33719 pg/mL/ 10^6 cells
NYSCT-05	1.0×10^9	98%	25.7%	27859 pg/mL/ 10^6 cells
Criteria	**1.0×10^9**	***≥70%***	***≥10%***	***>600pg/mL/ 10^6cells***

Supplementary Table 2. Patient PBMC manufacturing outcomes. Lot release criteria are highlighted in the bottom row.

Patient Study Number	Timepoint	L5 SUV_{max}	L5 SUV_{peak}	spleen SUV_{max}	spleen SUV_{peak}
NYSCT-01	day +29	9.5	8.5	7.8	7.1
NYSCT-01	day +120	0.5	0.4	0.6	0.5
NYSCT-03	day +25	12	10.8	10.9	10.3
NYSCT-05	day +34	0.6	0.5	0.8	0.6

Supplementary Table 3. Biodistribution data for [18F]-FHBG PET/CT visualization of sr39tk transgenic HSCs. 7-7.5mCi of [18F] FHBG was injected for each PET/CT scan for determination of sr39tk transgenic HSCs. The normal biodistribution of FHBG does not demonstrate significant uptake in bone marrow or splenic tissue. Visual assessment was used for evaluation of the Day +25 post-transplant scan and the Day +120 scan available for of one of the patients. In addition VOI (1cm^3) of representative marrow (L5 vertebra) and splenic regions were placed and the SUV_{max} and SUV_{peak} calculated for all scans. Visual assessment demonstrated altered biodistribution on the +25 day scan for 2 patients (NYSCT-01 day +25, NYSCT-03 day +25) with high FHBG uptake throughout the marrow and spleen. The scan of NYSCT-01 at day +120, as well as the third patient (NYSCT-05 day +34) did not show altered biodistribution.

Supplementary Table 4. LV-optNYESOTCR/TK-transduced CD34+ Cells: Final Product Lot Release Criteria (Results Available Before Administration to Patients)

Test	Method	Acceptance Criteria
CD34 enumeration	ISHAGE flow cytometric method	≥2.0 - 6.0 $\times 10^6$ CD34+cells/kg
% CD34 purity	ISHAGE flow cytometric method	≥50%
Cell viability	Trypan Blue manual count or AO/DAPI Nucleocounter NC200 automated count	≥70%
Vector Copy Number	ddPCR	0.1-2 copies/cell
Sterility[b]	Sterility culture	Negative
	Fungal culture	Negative
Endotoxin	Endosafe®PTS	≤ 5 EU/Kg
Mycoplasma[a]	MycoAlert test	Ratio<1
CFU assay	CFU potential Methylcellulose	≥10%
% LV-NYESO+ CFUs	ddPCR	5%-50% LV-NYESO+ colonies*
Vβ13.1 expression	Flow Cytometry	5%-50% of Vb13.1+ cells*

*In order to make sure that the repertoire of endogenous TCRs will not be compromised, at least 50% of the CD34+ PBSC delivered to the patient will be untransduced.

Supplementary Table 5. RV-NYESOTCR-transduced PBMC: Final Product Lot Release Criteria

Test	Method	Acceptance Criteria
Cell viability	Trypan Blue manual count or AO/DAPI Nucleocounter NC200 automated count	≥70%
Sterility[b]	Sterility culture	Negative
	Fungal culture	Negative
Endotoxin	Endosafe®PTS	≤ 5 EU/Kg
Mycoplasma[a]	MycoAlert test	Ratio<1
CFU assay	CFU potential Methylcellulose	≥10%
Antigen-specific antitumor functionality	Co-culture of transduced PBMCs with M257-A2 cells, followed by ELISA of co-culture media for IFN-γ	> 600 pg/ml/million cells of NY-ESO-1 specific IFN-γ production by ELISA
NY-ESO-1 TCR expression	Flow Cytometry	>10% of cells
