# Supplementary material for: Human cancer-targeted immunity via transgenic hematopoietic stem cell progeny
Source: Nat Commun. 2025 Jul 1;16:5599. doi: 10.1038/s41467-025-60816-z (PMC12219382; doi:10.1038/s41467-025-60816-z)
Supplement: Supplementary file 2 — Description of Additional Supplementary Files [file 41467_2025_60816_MOESM2_ESM.pdf]

Description of Additional Supplementary Files

Supplementary Data 1. Summary of adverse events experienced and their respective attributions

Supplementary Data 2. Lentivirus (pRRL-NYESOsr39TK_LTR_to_LTR) pRRL_TRCB nucleotide sequence

Supplementary Data 3. Clinical Trial Protocol

Supplementary Data 4. Retrovirus (pMSGV1_LTR_to_LTR) pMSGV1-retro nucleotide sequence

Supplementary Data 5. Gene sets used for celltype.l1 (filtered for the most ubiquitously expressed genes across all nuclei to avoid over-calling) from the Azimuth Reference for human PBMC

Supplementary Data 6. The gene sets used for celltype.l2 filtered for the most ubiquitously expressed genes across all nuclei to avoid over-calling) from the Azimuth Reference for human PBMC
