# Supplementary material for: Human cancer-targeted immunity via transgenic hematopoietic stem cell progeny
Source: Nat Commun. 2025 Jul 1;16:5599. doi: 10.1038/s41467-025-60816-z (PMC12219382; doi:10.1038/s41467-025-60816-z)
Supplement: Supplementary file 3 — Supplementary Data 1 [file 41467_2025_60816_MOESM3_ESM.docx]

# Supplementary Data 1. Summary of adverse events experienced and their respective attributions.

# Adverse Events Definitely Related to Conditioning Chemotherapy (Busulfan and/or Fludarabine)

| **AE Description** | **Grade** | | | | | **Number of Subjects that Experienced AE** |
| --- | --- | --- | --- | --- | --- | --- |
|  | **1** | **2** | **3** | **4** | **5** |  |
| Anemia |  | 1 |  |  |  | 1 |
| Neutrophil count decreased |  | 1 |  | 1 |  | 1 |
| Thrombocytopenia |  |  |  | 1 |  | 1 |
| White cell count decreased |  |  |  | 1 |  | 1 |

**Adverse Events Probably Related to Conditioning Chemotherapy (Busulfan and/or Fludarabine)**

| **AE Description** | **Grade** | | | | | **Number of Subjects that Experienced AE** |
| --- | --- | --- | --- | --- | --- | --- |
|  | **1** | **2** | **3** | **4** | **5** |  |
| Bone pain | 1 |  |  |  |  | 1 |
| GERD | 1 |  |  |  |  | 1 |
| Proteinuria |  | 1 |  |  |  | 1 |
| Pruritis | 1 |  |  |  |  | 1 |
| Weight loss | 1 |  |  |  |  | 1 |

**Adverse Events Possibly Related to Conditioning Chemotherapy (Busulfan and/or Fludarabine)**

| **AE Description** | **Grade** | | | | | **Number of Subjects that Experienced AE** |
| --- | --- | --- | --- | --- | --- | --- |
|  | **1** | **2** | **3** | **4** | **5** |  |
| Anemia |  | 1 |  |  |  | 1 |
| Ear pain | 1 |  |  |  |  | 1 |
| Hypophosphatemia |  |  |  | 1 |  | 1 |
| Hypotension | 1 |  |  |  |  | 1 |
| Hypoxia |  |  |  | 1 | 1 | 1 |
| Lymphopenia |  |  |  | 1 |  | 1 |
| Nausea |  | 1 |  |  |  | 1 |
| Neutrophil count decreased |  |  |  | 1 |  | 1 |
| Platelet count decreased |  |  |  | 1 |  | 1 |
| Shortness of breath |  |  |  | 1 | 1 | 1 |
| WBC decreased |  |  |  | 1 |  | 1 |

**Adverse Events Possibly Related to RV-NYESO TCR PBMC**

| **AE Description** | **Grade** | | | | | **Number of Subjects that Experienced AE** |
| --- | --- | --- | --- | --- | --- | --- |
|  | **1** | **2** | **3** | **4** | **5** |  |
| Agitation |  | 1 |  |  |  | 1 |
| Confusion |  | 1 |  |  |  | 1 |
| Cough |  | 1 |  |  |  | 1 |
| Cytokine release syndrome |  | 1 |  |  |  | 1 |
| Delirium |  | 1 |  |  |  | 1 |
| Hallucinations |  | 1 |  |  |  | 1 |
| Headache |  | 1 |  |  |  | 1 |
| Hypoalbuminemia |  | 1 |  |  |  | 1 |
| Hypophosphatemia |  | 1 |  |  |  | 1 |
| Hypotension |  |  | 1 |  |  | 1 |
| Hypoxia |  | 1 |  |  |  | 1 |
| Non-cardiac chest pain | 1 |  |  |  |  | 1 |
| Somnolence |  | 1 |  |  |  | 1 |

**Adverse Events Probably Related to LV-NYESO TCR/sr39TK PBSC**

| **AE Description** | **Grade** | | | | | **Number of Subjects that Experienced AE** |
| --- | --- | --- | --- | --- | --- | --- |
|  | **1** | **2** | **3** | **4** | **5** |  |
| **Pruritis** | **1** |  |  |  |  | **1** |

**Adverse Events Possibly Related to LV-NYESO TCR/sr39TK PBSC**

| **AE Description** | **Grade** | | | | | **Number of Subjects that Experienced AE** |
| --- | --- | --- | --- | --- | --- | --- |
|  | **1** | **2** | **3** | **4** | **5** |  |
| Chills, intermittent | 1 |  |  |  |  | 1 |
| Fever | 1 |  |  |  |  | 1 |

**Adverse Events Definitely Related to Interleukin-2**

| **AE Description** | **Grade** | | | | | **Number of Subjects that Experienced AE** |
| --- | --- | --- | --- | --- | --- | --- |
|  | **1** | **2** | **3** | **4** | **5** |  |
| Diarrhea | 1 |  |  |  |  | 1 |
| Fever | 1 |  |  |  |  | 1 |
| Hypophosphatemia |  |  | 1 |  |  | 1 |
| Macular rash |  |  | 1 |  |  | 1 |
| Sinus tachycardia | 1 |  |  |  |  | 1 |
| Vomiting |  | 1 |  |  |  | 1 |
| Weight loss | 1 |  |  |  |  | 1 |

**Adverse Events Probably Related to Interleukin-2**

| **AE Description** | **Grade** | | | | | **Number of Subjects that Experienced AE** |
| --- | --- | --- | --- | --- | --- | --- |
|  | **1** | **2** | **3** | **4** | **5** |  |
| Proteinuria |  | 1 |  |  |  | 1 |
| Pruritis | 1 |  |  |  |  | 1 |
| Weight Loss | 1 |  |  |  |  | 1 |

**Adverse Events Possibly Related to Interleukin-2**

| **AE Description** | **Grade** | | | | | **Number of Subjects that Experienced AE** |
| --- | --- | --- | --- | --- | --- | --- |
|  | **1** | **2** | **3** | **4** | **5** |  |
| Agitation |  | 1- |  |  |  | 1 |
| Chills, intermittent | 1 |  |  |  |  | 1 |
| Cough |  | 1 |  |  |  | 1 |
| Cytokine release syndrome |  | 1 |  |  |  | 1 |
| Delirium |  | 1 |  |  |  | 1 |
| Fever |  | 1 |  |  |  | 1 |
| Hallucinations |  | 1 |  |  |  | 1 |
| Headache | 1 |  |  |  |  | 1 |
| Hypoalbuminemia |  | 1 |  |  |  | 1 |
| Hypophosphatemia |  | 1 |  |  |  | 1 |
| Hypotension |  |  | 1 |  |  | 1 |
| Hypoxia |  | 1 |  |  |  | 1 |
| Non-cardiac chest pain | 1 |  |  |  |  | 1 |
| Somnolence |  | 1 |  |  |  | 1 |
| Thrombocytopenia |  |  | 1 |  |  | 1 |

**Adverse Events Definitely Related to Other (disease, intercurrent illness, or concomitant medication)**

| **AE Description** | **Grade** | | | | | **Number of Subjects that Experienced AE** |
| --- | --- | --- | --- | --- | --- | --- |
|  | **1** | **2** | **3** | **4** | **5** |  |
| Cough ^a^ | 1 |  |  |  |  | 1 |
| Hypothyroidism ^c^ |  | 1 |  |  |  | 1 |
| Palmar-plantar syndrome ^c^ |  | 1 |  |  |  | 1 |

a Disease; b Intercurrent illness; c Concomitant medication

# Adverse Events Probably Related to Other (disease, intercurrent illness, or concomitant medication)

| **AE Description** | **Grade** | | | | | **Number of Subjects that Experienced AE** |
| --- | --- | --- | --- | --- | --- | --- |
|  | **1** | **2** | **3** | **4** | **5** |  |
| Anemia ^a^ | 1 |  |  |  |  | 1 |
| Alanine aminotransferase increased ^c^ | 1 |  |  |  |  | 1 |
| Aspartate aminotransferase increased ^c^ |  | 1 |  |  |  | 1 |
| Decreased appetite ^c^ | 1 |  |  |  |  | 1 |
| Diarrhea ^c^ | 1 |  |  |  |  | 1 |
| Fever ^c^ |  |  | 1 |  |  | 1 |
| Hypophosphatemia ^c^ |  |  | 1 |  |  | 1 |
| Infections and infestations ^a,^ ^c^ |  | 1 |  |  |  | 1 |
| Insomnia ^c^ |  | 1 |  |  |  | 1 |
| Leukopenia ^c^ |  | 1 |  |  |  | 1 |
| Macular rash ^c^ | 1 |  |  |  |  | 1 |
| Nausea ^c^ |  | 1 |  |  |  | 1 |
| Pulmonary embolism ^b,^ ^c^ |  | 1 |  |  |  | 1 |
| Rigors ^c^ |  | 1 |  |  |  | 1 |
| Thrombocytopenia ^c^ | 1 |  |  |  |  | 1 |
| Urticaria ^c^ |  | 1 |  |  |  | 1 |

a Disease; b Intercurrent illness; c Concomitant medication
