# Supplementary material for: Human cancer-targeted immunity via transgenic hematopoietic stem cell progeny
Source: Nat Commun. 2025 Jul 1;16:5599. doi: 10.1038/s41467-025-60816-z (PMC12219382; doi:10.1038/s41467-025-60816-z)
Supplement: Supplementary file 4 — Supplementary Data 2 [file 41467_2025_60816_MOESM4_ESM.docx]

### Supplementary Data 2. Lentivirus (pRRL-NYESOsr39TK_LTR_to_LTR ) pRRL_TRCB nucleotide sequence

>pRRL-NYESOsr39TK_LTR_to_LTR

taatgtagtcttatgcaatactcttgtagtcttgcaacatggtaacgatgagttagcaacatgccttacaaggagagaaaaagcaccgtgcatgccgattggtggaagtaaggtggtacgatcgtgccttattaggaaggcaacagacgggtctgacatggattggacgaaccactgaattgccgcattgcagagatattgtatttaagtgcctagctcgatacataaacgggtctctctggttagaccagatctgagcctgggagctctctggctaactagggaacccactgcttaagcctcaataaagcttgccttgagtgcttcaagtagtgtgtgcccgtctgttgtgtgactctggtaactagagatccctcagacccttttagtcagtgtggaaaatctctagcagtggcgcccgaacagggacttgaaagcgaaagggaaaccagaggagctctctcgacgcaggactcggcttgctgaagcgcgcacggcaagaggcgaggggcggcgactggtgagtacgccaaaaattttgactagcggaggctagaaggagagagatgggtgcgagagcgtcagtattaagcgggggagaattagatcgcgatgggaaaaaattcggttaaggccagggggaaagaaaaaatataaattaaaacatatagtatgggcaagcagggagctagaacgattcgcagttaatcctggcctgttagaaacatcagaaggctgtagacaaatactgggacagctacaaccatcccttcagacaggatcagaagaacttagatcattatataatacagtagcaaccctctattgtgtgcatcaaaggatagagataaaagacaccaaggaagctttagacaagatagaggaagagcaaaacaaaagtaagaccaccgcacagcaagcggccgctgatcttcagacctggaggaggagatatgagggacaattggagaagtgaattatataaatataaagtagtaaaaattgaaccattaggagtagcacccaccaaggcaaagagaagagtggtgcagagagaaaaaagagcagtgggaataggagctttgttccttgggttcttgggagcagcaggaagcactatgggcgcagcgtcaatgacgctgacggtacaggccagacaattattgtctggtatagtgcagcagcagaacaatttgctgagggctattgaggcgcaacagcatctgttgcaactcacagtctggggcatcaagcagctccaggcaagaatcctggctgtggaaagatacctaaaggatcaacagctcctggggatttggggttgctctggaaaactcatttgcaccactgctgtgccttggaatgctagttggagtaataaatctctggaacagatttggaatcacacgacctggatggagtgggacagagaaattaacaattacacaagcttaatacactccttaattgaagaatcgcaaaaccagcaagaaaagaatgaacaagaattattggaattagataaatgggcaagtttgtggaattggtttaacataacaaattggctgtggtatataaaattattcataatgatagtaggaggcttggtaggtttaagaatagtttttgctgtactttctatagtgaatagagttaggcagggatattcaccattatcgtttcagacccacctcccaaccccgaggggacccgacaggcccgaaggaatagaagaagaaggtggagagagagacagagacagatccattcgattagtgaacggatctcgacggtatcggttaacttttaaaagaaaaggggggattggggggtacagtgcaggggaaagaatagtagacataatagcaacagacatacaaactaaagaattacaaaaacaaattacaaaaattcaaaattttatcgatcacgagactagcctcgagggaatgaaagaccccacctgtaggtttggcaagctagcttaagtaacgccattttgcaaggcatggaaaatacataactgagaatagagaagttcagatcaaggttaggaacagagagacagcagaatatgggccaaacaggatatctgtggtaagcagttcctgccccggctcagggccaagaacagatggtccccagatgcggtcccgccctcagcagtttctagagaaccatcagatgtttccagggtgccccaaggacctgaaatgaccctgtgccttatttgaactaaccaatcagttcgcttctcgcttctgttcgcgcgcttctgctccccgagctcaataaaagagcccacaacccctcactcggcgcgccagtccggatccgccaccatggaaacactgctgggcctgctgatcctgtggctgcagctgcagtgggtgtccagcaagcaggaagtgacccagatccctgccgccctgtctgtgcctgagggcgagaacctggtgctgaactgcagcttcaccgacagcgccatctacaacctgcagtggttcagacaggaccccggcaagggcctgacaagcctgctgctgattcagagcagccagagagagcagaccagcggcagactgaacgccagcctggataagagcagcggccggtccaccctgtatatcgccgcttctcagcctggcgactccgccacatatctgtgtgctgtgcggcctctgtacggcggcagctacatccctaccttcggcagaggcaccagcctgatcgtgcacccctacatccagaaccccgaccccgccgtgtaccagctgagagacagcaagtccagcgacaagagcgtgtgcctgttcaccgacttcgacagccagaccaacgtgtcccagagcaaggacagcgacgtgtacatcaccgacaagaccgtgctggacatgcggagcatggacttcaagagcaacagcgccgtggcctggtccaacaagagcgatttcgcctgcgccaacgccttcaacaacagcattatccccgaggacacattcttcccaagccccgagagcagctgcgacgtgaagctggtggaaaagagcttcgagacagacaccaacctgaacttccagaacctgagcgtgatcggcttccggattctgctgctgaaggtggccggcttcaacctgctgatgaccctgagactgtggtccagccgggccaagagatctggcagcggcgccaccaatttcagcctgctgaaacaggccggcgacgtggaagagaaccctggccctatgagcatcggcctgctgtgttgtgccgctctgtccctgctgtgggccggacctgtgaatgctggcgtgacacagacccccaagttccaggtgctgaaaaccggccagagcatgaccctgcagtgcgcccaggacatgaaccacgagtacatgagctggtatcggcaggaccctggcatgggactgcggctgatccactactctgtgggcgccggcatcaccgatcagggcgaggtgcccaacggctacaatgtgtccagatccaccaccgaggacttcccactgagactgctgtctgccgcccctagccagacctccgtgtacttctgtgccagcagctacgtgggcaacaccggcgagctgttctttggcgagggcagcagactgacagtgctggaagatctgaagaacgtgttccccccagaggtggccgtgttcgagccttctgaggccgagatcagccacacccagaaagccaccctcgtgtgtctggccaccggcttctaccccgaccacgtggaactgtcttggtgggtcaacggcaaagaggtgcacagcggcgtgtccaccgatccccagcctctgaaagagcagcccgccctgaacgacagccggtactgtctgtcctcccggctgagagtgtccgccaccttctggcagaacccccggaaccacttcagatgccaggtgcagttctacggcctgagcgagaacgacgagtggacccaggacagagccaagcccgtgactcagatcgtgtctgccgaggcctggggcagagccgattgtggctttaccagcgagagctaccagcagggcgtgctgagcgccaccatcctgtacgagatcctgctgggcaaggccaccctgtacgccgtgctggtgtccgccctggtgctgatggccatggtcaaacggaaggacttcagagccaagcggggaaagcctatccctaatcctctgctgggactggactccaccggctctggcgagggcagaggctctctgctgacctgcggagatgtggaagaaaatcccggccctatgcccaccctgctgcgggtgtacatcgacggcccccacggcatgggcaagaccaccaccacacagctgctggtggccctgggcagcagggacgacatcgtgtacgtgcccgagcccatgacatactggcgggtgctgggcgccagcgagacaatcgccaacatctacaccacccagcacagactggaccagggcgagatttctgccggcgacgccgccgtggtcatgaccagcgcccagatcaccatgggaatgccctacgccgtgacagatgccgtgctggcccctcacattggcggcgaggccggatcttctcatgccccaccccctgctctgaccatcttcctggaccggcaccctatcgccttcatgctgtgctaccctgccgccagatacctgatgggcagcatgaccccacaggctgtgctggctttcgtggccctgatccctcctaccctgcccggcaccaatatcgtgctgggggccctgcccgaggacagacacatcgaccggctggccaagagacagcggcctggcgagagactggatctggccatgctggccgccatcagaagagtgtacggcctgctggccaacaccgtgcggtatctgcagtgcggcggctcttggagagaggactggggccagctgtctggaacagctgtgccacctcaaggcgccgagcctcagtctaatgccggccctagaccccacatcggcgacaccctgtttaccctgttcagagcccccgagctgctggcccccaacggcgacctgtacaacgtgttcgcctgggctctggatgtgctggccaagcggctgcggagcatgcacgtgttcatcctggactacgaccagagccctgccggctgtagagatgccctgctgcagctgaccagcggcatggtgcagacccacgtgaccacccctggcagcatccccaccatctgcgacctggcccggacctttgccagagagatgggcgaggccaactgagtcgacaatcaacctctggattacaaaatttgtgaaagattgactggtattcttaactatgttgctccttttacgctatgtggatacgctgctttaatgcctttgtatcatgctattgcttcccgtatggctttcattttctcctccttgtataaatcctggttgctgtctctttatgaggagttgtggcccgttgtcaggcaacgtggcgtggtgtgcactgtgtttgctgacgcaacccccactggttggggcattgccaccacctgtcagctcctttccgggactttcgctttccccctccctattgccacggcggaactcatcgccgcctgccttgcccgctgctggacaggggctcggctgttgggcactgacaattccgtggtgttgtcggggaagctgacgtcctttccatggctgctcgcctgtgttgccacctggattctgcgcgggacgtccttctgctacgtcccttcggccctcaatccagcggaccttccttcccgcggcctgctgccggctctgcggcctcttccgcgtcttcgccttcgccctcagacgagtcggatctccctttgggccgcctccccgcctggaattcgagctcggtacctttaagaccaatgacttacaaggcagctgtagatcttagccactttttaaaagaaaaggggggactggaagggctaattcactcccaacgaagacaagatctgctttttgcttgtactgggtctctctggttagaccagatctgagcctgggagctctctggctaactagggaacccactgcttaagcctcaataaagcttgccttgagtgcttcaagtagtgtgtgcccgtctgttgtgtgactctggtaactagagatccctcagacccttttagtcagtgtggaaaatctctagc
