# Supplementary material for: Human cancer-targeted immunity via transgenic hematopoietic stem cell progeny
Source: Nat Commun. 2025 Jul 1;16:5599. doi: 10.1038/s41467-025-60816-z (PMC12219382; doi:10.1038/s41467-025-60816-z)
Supplement: Supplementary file 5 — Supplementary Data 3 [file 41467_2025_60816_MOESM5_ESM.docx]

**California Institute for Regenerative Medicine (CIRM) Protocol**

**TITLE: Adoptive Transfer of NY-ESO-1 TCR Engineered Peripheral Blood Mononuclear Cells (PBMC) and Peripheral Blood Stem Cells (PBSC) after a Myeloablative Conditioning Regimen, with Administration of Interleukin-2, in Patients with Advanced Malignancies**

**UCLA IRB #: 15-000511**

**UCLA ISPRC/DSMB Ribas NYESO SCT Cancer**

**FDA IND #: 17471**

**NIH OBA/RAC #: 1706-1625**

**UCLA IBC #: BUA-2016-211-001**

**Clinical Trial Registration #: NCT03240861**

**PHASE: 1**

**VERSION DATE: 05/13/22 Amendment 19**

**Study Agents: 1) Retroviral Vector NY-ESO-1 TCR Engineered PBMC**

**(RV-NYESO TCR PBMC)**

**2) Lentiviral Vector NY-ESO-1 TCR Engineered PBSC**

**(LV-NYESO TCR/sr39TK PBSC)**

**Cross Referenced INDs: NY-ESO-1 TCR: 15167 (Ribas)**

**FHBG: 135251 (Ribas)**

**G1XCGD (Reference for conditioning regimen): 16141 (Kohn)**

**PRINCIPAL INVESTIGATOR:**

Theodore Scott Nowicki, M.D., Ph.D. UCLA tnowicki@mednet.ucla.edu 310-825-6708

**CO-PRINCIPAL INVESTIGATOR:**

Antoni Ribas, M.D., Ph.D. UCLA aribas@mednet.ucla.edu 310-206-3928

**CLINICAL CO-INVESTIGATORS:** (in alphabetical order) (with human subject responsibilities)

Martin Allen-Auerbach, M.D. UCLA mauerbach@mednet.ucla.edu 310-983-1444 Bartosz Chmielowski, M.D., Ph.D. UCLA bchmielowski@mednet.ucla.edu 310-206-8509

Alistair J. Cochran, M.D. UCLA acochran@mednet.ucla.edu 310-825-2743

Johannes Czernin, M.D. UCLA jczernin@mednet.ucla.edu 310-206-2179

James S. Economou, M.D., Ph.D. UCLA jeconomou@mednet.ucla.edu 310-825-2644

Noah Federman, M.D. UCLA nfederman@mednet.ucla.edu 310-825-6708

John A. Glaspy, M.D., MPH UCLA jglaspy@mednet.ucla.edu 310-794-4955

Jonathan Goldman, M.D. UCLA jwgoldman@mednet.ucla.edu 310-633-8400

Anusha Kalbasi, M.D. UCLA anushakalbasi@mednet.ucla.edu 310-825-9777

Sarah Larson, M.D. UCLA slarson@mednet.ucla.edu 310-206-8477

Joshua Sasine, M.D. UCLA jsasine@mednet.ucla.edu 310-206-9352

Gary Schiller, M.D. UCLA gschiller@mednet.ucla.edu 310-206-5755

Arun Singh, M.D. UCLA [asingh@mednet.ucla.edu](mailto:asingh@mednet.ucla.edu) 310-829-5471

Deborah Wong, M.D., Ph.D. UCLA dewong@mednet.ucla.edu 310-794-4955

Sant Chawla, M.D. UCLA/Sarcoma Oncology Center santchawla@sarcomaoncology.com 310-552-9999

**NON-CLINICAL CO-INVESTIGATORS:** (in alphabetical order) (no human subject responsibilities)

Ignacio Baselga, Ph.D. UCLA [ibaselga@mednet.ucla.edu](mailto:ibaselga@mednet.ucla.edu) 310-206-2090

Beatriz Campo, Ph.D. UCLA [bcampo@mednet.ucla.edu](mailto:bcampo@mednet.ucla.edu) 310-794-1940

Begonya Comin-Anduix, Ph.D. UCLA bcomin@mednet.ucla.edu 310-267-2211

Zoran Galic, Ph.D. UCLA zgalic@ucla.edu 310-206-1023

Donald Kohn, M.D. UCLA dkohn@mednet.ucla.edu 310-794-1964

Cole W. Peters, Ph.D. UCLA [cwpeters@mednet.ucla.edu](mailto:cwpeters@mednet.ucla.edu) 310-206-2090

Cristina Puig Saus, Ph.D. UCLA cpuigsaus@mednet.ucla.edu 310-267-0596

Xiaoyan Wang, Ph.D. UCLA xywang@mednet.ucla.edu 310-794-3114

Owen N. Witte M.D. UCLA owenw@microbio.ucla.edu 310-206-6411

Lili Yang, Ph.D. UCLA liliyang@mednet.ucla.edu 310-825-8609

Jerome A. Zack, Ph.D. UCLA jzack@ucla.edu 310-206-6828

TABLE OF CONTENTS

[1 STUDY SYNOPSIS 8](#_Toc75362365)

[1.1 Overview 8](#_Toc75362366)

[1.2 Study Rationale 9](#_Toc75362367)

[1.3 Study Objectives 12](#_Toc75362368)

[1.4 Study Population 13](#_Toc75362369)

[1.5 Trial Design and Number of Patients 13](#_Toc75362370)

[1.6 Study Duration 14](#_Toc75362371)

[1.7 Study Schematic 14](#_Toc75362372)

[1.8 Experimental Study Agents 17](#_Toc75362373)

[1.8.1 Retrovirus vector MSGV1-A2aB-1G4A-LY3H10 17](#_Toc75362374)

[1.8.2 Lentivirus vector pRRL-MSCV-optNYESO optsr39TK-WPRE-1 (LV-optNYESOTCR/TK) 18](#_Toc75362375)

[1.9 Timing of Events 18](#_Toc75362376)

[2 Schedule of Events 19](#_Toc75362377)

[3 TABLE OF ABBREVIATIONS 23](#_Toc75362378)

[4 STUDY OBJECTIVES 24](#_Toc75362379)

[4.1 Primary 24](#_Toc75362380)

[4.1.1 Safety 24](#_Toc75362381)

[4.2 Secondary 25](#_Toc75362382)

[4.2.1 Feasibility 25](#_Toc75362383)

[4.2.2 NY-ESO-1 TCR Transgenic T Cell Persistence. 25](#_Toc75362384)

[4.2.3 Clinical Response 25](#_Toc75362385)

[4.3 Exploratory 25](#_Toc75362386)

[4.3.1 *In Vivo* Imaging. 25](#_Toc75362387)

[4.3.2 Clinical Response per iRECIST. 25](#_Toc75362388)

[5 BACKGROUND 26](#_Toc75362389)

[5.1 NY-ESO-1 Antigen as a Target for Immunotherapy 26](#_Toc75362390)

[5.2 Rationale for Immune-based Therapies for Advanced Malignancies. 26](#_Toc75362391)

[5.3 Lessons Learned from Targeting Tumor Associated Antigens with Immunotherapy 26](#_Toc75362392)

[5.4 Why Gene Therapy 27](#_Toc75362393)

[5.5 TCR Gene Therapy 27](#_Toc75362394)

[5.6 Retroviral Vector-based Human Gene Medicine 27](#_Toc75362395)

[5.7 Safety and Regulatory Issues of Retroviral-based Gene Transfer Approaches 27](#_Toc75362396)

[5.8 Gene Therapy Utilizing Lentiviral Vectors 28](#_Toc75362397)

[5.9 Safety and Regulatory Issues Regarding Lentivirus Gene Transfer Approaches 28](#_Toc75362398)

[5.10 NY-ESO-1 TCR-engineered T Cell Experience In Humans 28](#_Toc75362399)

[6 PRELIMINARY DATA 29](#_Toc75362400)

[6.1 Origin and Functionality of the NY-ESO-1 TCR 29](#_Toc75362401)

[6.2 Preliminary Data at the Surgery Branch/NCI 29](#_Toc75362402)

[6.3 Preliminary Data at UCLA 29](#_Toc75362403)

[6.4 Preliminary Data by Adaptimmune 30](#_Toc75362404)

[7 ELIGIBILITY CRITERIA 30](#_Toc75362405)

[7.1 Inclusion Criteria 30](#_Toc75362406)

[7.2 Exclusion Criteria 30](#_Toc75362407)

[7.3 Definition of Study Enrollment Date 31](#_Toc75362408)

[8 STUDY PROCEDURES 32](#_Toc75362409)

[8.1 Baseline Screening Assessments 32](#_Toc75362410)

[8.2 Initial Protocol Procedures 33](#_Toc75362411)

[8.3 G-CSF Mobilized Leukapheresis (Day -180 to -21 including mDay 1 - 8) 33](#_Toc75362412)

[8.3.1 Collection of G-CSF and Plerixafor Mobilized Cells for Transduction and Unmodified PBSC for Cryopreservation. 33](#_Toc75362413)

[8.3.2 Confirm Vector Copy Number and Transduction Efficiency Prior to Initiating Unmobilized Partial Leukapheresis 34](#_Toc75362414)

[8.3.3 Supportive Therapy during G-CSF and Plerixafor Mobilization 35](#_Toc75362415)

[8.4 Unmobilized Partial Leukapheresis, Cell Processing and Admission to the Hospital 35](#_Toc75362416)

[8.5 Days –5 to –2 Conditioning Chemotherapy Regimen 35](#_Toc75362417)

[8.5.1 Supportive Therapy during Chemotherapy Administration: 35](#_Toc75362418)

[8.5.2 Chemotherapy Conditioning (Days -5 to -2): 36](#_Toc75362419)

[8.5.3 Prophylactic Supportive Care Throughout the Pancytopenic Period. 37](#_Toc75362420)

[8.6 Day 0-1: LV-NYESO TCR/sr39TK PBSC and RV-NYESO TCR PBMC Infusion 37](#_Toc75362421)

[8.6.1 Premedication 37](#_Toc75362422)

[8.6.2 Day 0: LV-NYESO TCR/sr39TK PBSC Infusion 38](#_Toc75362423)

[8.6.3 Day 1: RV-NYESO TCR PBMC Infusion 38](#_Toc75362424)

[8.6.4 Patient Monitoring After LV-NYESO TCR/sr39TK PBSC and RV-NYESO TCR PBMC Infusion 38](#_Toc75362425)

[8.7 Low Dose IL-2 and Blood Draws for Immune Response Assessments 39](#_Toc75362426)

[8.8 Hospital Discharge 40](#_Toc75362427)

[8.9 Day 25: NY-ESO-1 TCR Cell Tracking After Infusion 40](#_Toc75362428)

[8.10 Days 30, 45, 60, 75, 90 and 120: Follow Up Study Visits 40](#_Toc75362429)

[8.11 Permissible Systemic Therapies During the Protocol On-study Period 41](#_Toc75362430)

[8.12 Day 60: Re-staging Evaluation 41](#_Toc75362431)

[8.13 Day 120: NY-ESO-1 TCR Cell Tracking After Infusion 41](#_Toc75362432)

[8.14 Long-term Follow-up Plan 41](#_Toc75362433)

[8.15 Elimination of Transduced PBSC by Activation of Suicide Gene 41](#_Toc75362434)

[8.16 Bone Marrow Biopsy and Administration of Unmodified Back Up Cells in the Event of Marrow Recovery Failure 42](#_Toc75362435)

[9 EXPERIMENTAL STUDY AGENTS 42](#_Toc75362436)

[9.1 PBMC Expressing NY-ESO-1 TCR (RV-NYESO TCR PBMC) 42](#_Toc75362437)

[9.1.1 Retrovirus Vector MSGV1-A2aB-1G4A-LY3H10 42](#_Toc75362438)

[9.1.2 RV-NYESO TCR PBMC 44](#_Toc75362439)

[9.2 CD34+ Cells Expressing NY-ESO-1 TCR/sr39TK (LV-NYESO TCR/sr39TK PBSC) 46](#_Toc75362440)

[9.2.1 Lentiviral Vector pRRL-MSCV-optNYESO optsr39TK-WPRE-1 46](#_Toc75362441)

[9.2.2 LV-NYESO TCR/sr39TK PBSC 48](#_Toc75362442)

[9.3 Reported Adverse Events and Potential Risks 49](#_Toc75362443)

[9.3.1 Retroviral Vectors 49](#_Toc75362444)

[9.3.2 Lentiviral Vectors 49](#_Toc75362445)

[9.3.3 NY-ESO-1 TCR 50](#_Toc75362446)

[9.3.4 sr39TK 50](#_Toc75362447)

[9.3.5 RV-NYESO TCR PBMC 50](#_Toc75362448)

[9.4 LV-NYESO TCR/sr39TK PBSC 51](#_Toc75362449)

[9.4.1 Amplification of Toxicities. 51](#_Toc75362450)

[9.4.2 Marrow Recovery Failure. 51](#_Toc75362451)

[9.5 ADDITIONAL STUDY AGENTS 51](#_Toc75362452)

[9.5.1 IL-2 (aldesleukin, Proleukin, recombinant human Interleukin 2) 51](#_Toc75362453)

[9.5.2 Busulfan (Busulfex^®^) 51](#_Toc75362454)

[9.5.3 Fludarabine 53](#_Toc75362455)

[Mode of Action: Chemotherapy agent. 53](#_Toc75362456)

[9.5.4 Filgrastim [G-CSF] (Neupogen^®^) or Other Biosimilar 54](#_Toc75362457)

[9.5.5 Plerixafor (Mozobil) 54](#_Toc75362458)

[9.6 Laboratory Reagents 55](#_Toc75362459)

[9.6.1 OKT3 55](#_Toc75362460)

[9.6.2 Interleukin-2 (IL-2 ) 55](#_Toc75362461)

[9.6.3 LentiBOOST^TM^ 55](#_Toc75362462)

[9.6.4 Retronectin® 55](#_Toc75362462)

[9.6.5 Recombinant Human Stem Cell Factor (rhSCF) 5](#_Toc75362463)6

[9.6.6 Recombinant human Thrombopoietin (rhTPO) 5](#_Toc75362464)6

[9.6.7 Recombinant human Interleukin (IL-3) 5](#_Toc75362464)6

[9.6.8 Recombinant human Fms-Related Tyrosine Kinase 3 Ligand (rhFlt3L) 5](#_Toc75362464)6

[10 GENERATION OF EXPERIMENTAL CELL THERAPIES 56](#_Toc75362465)

[10.1 Generation of NY-ESO-1 TCR Transduced PBMC 56](#_Toc75362466)

[10.1.1 Leukapheresis Procedures 56](#_Toc75362467)

[10.1.2 PBMC Isolation 56](#_Toc75362468)

[10.1.3 PBMC Cell Activation. 56](#_Toc75362469)

[10.1.4 Clinical Grade Retrovirus MSGV1-A2aB-1G4A-LY3H10 Lot Release Testing 56](#_Toc75362470)

[10.1.5 Clinical Grade Retrovirus MSGV1-A2aB-1G4A-LY3H10 Annual Recertification 58](#_Toc75362471)

[10.1.6 Retrovirus Transduction Method 59](#_Toc75362472)

[10.1.7 Transgenic PBMC Product Washing and Bagging 60](#_Toc75362473)

[10.1.8 Transgenic PBMC Product Labeling 60](#_Toc75362474)

[10.1.9 Transgenic PBMC in-process Testing and Final Product Lot Release Testing 60](#_Toc75362475)

[10.2 Generation of NY-ESO-1 TCR/sr39TK Transduced CD34+ cells 62](#_Toc75362476)

[10.2.1 CD34+ Cell Mobilization Procedure and Leukapheresis 63](#_Toc75362477)

[10.2.2 Distribution of the CD34+ PBSCs obtained by Leukaphereses 63](#_Toc75362478)

[10.2.3 CD34+ Cell Isolation 63](#_Toc75362479)

[10.2.4 CD34+ Cell Activation. 64](#_Toc75362480)

[10.2.5 Clinical Grade Lentivirus LV-optNYESOTCR/TK Lot Release Testing 64](#_Toc75362481)

[10.2.6 Clinical Grade LV-optNYESOTCR/TK Annual Recertification 65](#_Toc75362482)

[10.2.7 LV-optNYESOTCR/TK Transduction Method 65](#_Toc75362483)

[10.2.8 LV-NYESO TCR/sr39TK PBSC (LV-optNYESOTCR/TK Transduced CD34+) Cell Formulation and Packaging 66](#_Toc75362484)

[10.2.9 LV-NYESO TCR/sr39TK PBSC (LV-optNYESOTCR/TK Transduced CD34+) Labeling and storage 66](#_Toc75362485)

[10.2.10 LV-NYESO TCR/sr39TK PBSC (LV-optNYESOTCR/TK Transduced CD34+ Cells) In-process Testing and Final Product Lot Release Testing 66](#_Toc75362486)

[11 EVALUATION OF STUDY ENDPOINTS 69](#_Toc75362487)

[11.1 Primary Study Endpoint 69](#_Toc75362488)

[11.1.1 Safety and Definition of Dose Limiting Toxicity (DLT) 69](#_Toc75362489)

[11.2 Secondary Study Endpoints: 71](#_Toc75362490)

[11.2.1 Feasibility 71](#_Toc75362491)

[11.2.2 Transgenic Cell Persistence. 71](#_Toc75362492)

[11.2.3 Objective Response 72](#_Toc75362493)

[11.3 Exploratory study endpoints: 74](#_Toc75362494)

[11.3.1 Evaluation of LV-NYESO TCR/sr39TK PBSC Biodistribution 74](#_Toc75362495)

[11.3.2 Evaluation of Delayed Clinical Response 75](#_Toc75362496)

[11.4 Definition of Study Failure Due to Failure of Meeting the Study Endpoints. 75](#_Toc75362497)

[12 ADVERSE EVENT REPORTING 76](#_Toc75362498)

[12.1 Definition of an Adverse Event (AE) 76](#_Toc75362499)

[12.2 Definition of a Serious Adverse Event (SAE) 76](#_Toc75362500)

[12.3 Pre-existing Conditions 77](#_Toc75362501)

[12.4 Procedures 77](#_Toc75362502)

[12.5 Assessing Severity of Adverse Events 77](#_Toc75362503)

[12.6 Classification of Causality 78](#_Toc75362504)

[12.7 Seriousness and Non-Serious Adverse Events 78](#_Toc75362505)

[12.8 Reporting of AEs and SAEs 78](#_Toc75362506)

[12.9 Follow-up of Serious Adverse Events 79](#_Toc75362507)

[12.10 Withdrawal Due to Adverse Events 80](#_Toc75362508)

[13 COMPLIANCE WITH GOOD CLINICAL PRACTICE, ETHICAL CONSIDERATIONS, INFORMED CONSENT 80](#_Toc75362509)

[13.1 Compliance with Good Clinical Practice and Ethical Considerations 80](#_Toc75362510)

[13.2 Patient Confidentiality 80](#_Toc75362511)

[13.3 Regulatory Approvals 80](#_Toc75362512)

[13.4 Subject Recruitment 80](#_Toc75362513)

[13.5 Gender and Minorities 81](#_Toc75362514)

[13.6 Children 81](#_Toc75362515)

[13.7 Informed Consent Procedure 81](#_Toc75362516)

[13.7.1 Informed Consent Form (ICF) 81](#_Toc75362517)

[13.7.2 Procedure of Consent 82](#_Toc75362518)

[13.8 Subject Enrollment 82](#_Toc75362519)

[14 QUALIFICATIONS OF PERSONNEL AND STUDY FACILITIES 82](#_Toc75362520)

[14.1 Qualifications of Investigators 82](#_Toc75362521)

[14.2 Personnel Training 85](#_Toc75362522)

[14.3 Human and Gene Cell Therapy Facility (HGCTF) at UCLA 85](#_Toc75362523)

[14.4 Gene Therapy Viral Bank 85](#_Toc75362524)

[14.5 Clinical Facilities 86](#_Toc75362525)

[14.6 Compliance with Protocol Procedures and Rights and Welfare of Study Participants 86](#_Toc75362526)

[15 DATA SAFETY MONITORING BOARD, STUDY OVERSIGHT, DATA COLLECTION AND ANALYSIS 86](#_Toc75362527)

[15.1 Data Safety Monitoring Board (DSMB) 86](#_Toc75362528)

[15.2 JCCC DSMB Internal Monitoring Plan 87](#_Toc75362529)

[15.2.1 Level of Risk of a Study 87](#_Toc75362530)

[15.2.2 Assignment of risk 87](#_Toc75362531)

[15.3 Monitoring/Auditing Activities 88](#_Toc75362532)

[15.4 Detailed Reporting Mechanism for Adverse Events 88](#_Toc75362533)

[15.5 Data Recording and Retention of Study Data 88](#_Toc75362534)

[16 STATISTICAL METHODS 89](#_Toc75362535)

[16.1 Sample Size Determination 89](#_Toc75362536)

[16.2 Definition of Evaluable Patients 89](#_Toc75362537)

[16.3 Primary Endpoint Analysis 89](#_Toc75362538)

[16.4 Secondary and Exploratory Endpoint Analysis 90](#_Toc75362539)

[16.5 Study Stopping and Non-stopping Rules. 90](#_Toc75362540)

[17 REFERENCES 91](#_Toc75362541)

[Figure 1. Clinical Trial Overview 15](#Figure1)

[Figure 2. Stem Cell Mobilization and Manufacture Scheme 16](#Figure2)

[Figure 3. T Cell Manufacture, Cell Administration Through Study Observation Period 16](#Figure3)

Table 1. Description of the dose and schedule of the proposed therapy combining PBMC with PBSC. 13

Figure 1. Clinical Trial Overview 14

Figure 2. Stem Cell Mobilization and Manufacture Scheme 15

Figure 3. T Cell Manufacture, Cell Administration Through Study Observation Period 15

Table 2. Schedule of Events 19

Table 3. Schedule of Events for Stem Cell Mobilization 22

Table 4. Clinical Grade MSGV1-A2aB-1G4A-LY3H10 Retrovirus Lot Release Criteria 57

Table 5. MSGV1-A2aB-1G4A-LY3H10 Retrovirus Annual Recertification 58

Table 6. Transgenic PBMC In-process Testing 60

Table 7. Transgenic PBMC Lot Release Testing 61

Table 8. NY-ESO-1 TCR Transgenic PBMC Optional Additional Testing 62

Table 9a. Clinical Grade Lentivirus LV-optNYESOTCR/TK Lot Release Criteria (Lot #040417L1) 64

Table 10. LV-optNYESOTCR/TK Annual Recertification 65

Table 10b. LV-optNYESOTCR/TK Lentivirus Annual Recertification 2019 (Lot #040417L1) 65

Table 4. Transduced CD34+ Cells In-process Testing 67

Table 12. LV-NYESO TCR/sr39TK PBSC (LV-optNYESOTCR/TK-transduced CD34+ Cells): 68

Final Product Lot Release Criteria 68

Table 13. LV-NYESO TCR/sr39TK PBSC (NY-ESO-1 TCR/sr39TK Transgenic CD34+ Cell) 69

Optional Additional Testing 69

Table 14. Criteria to Declare the Study Unfeasible 71

# STUDY SYNOPSIS

## Overview

This is a phase 1 clinical trial assessing the safety and toxicity of autologous peripheral blood stem cells (PBSC) transduced with an NY-ESO-1-specific T cell receptor (TCR) in combination with peripheral blood mononuclear cells (PBMC) transduced with an NY-ESO-1-specific TCR within an adoptive cell transfer (ACT) protocol.

Patients with NY-ESO-1-positive advanced malignancies who are human leukocyte antigen-A*0201 (HLA-A*0201)-positive, and human immunodeficiency virus (HIV), hepatitis B and C seronegative, will receive a reduced intensity conditioning regimen consisting of busulfan (2 mg/kg i.v., q12 hours on Days -5 to Day -2) and fludarabine (40 mg/m^2^/day on Days -5 to -2). Patients will then receive the ACT (Day 0) of autologous PBSCs transduced with LV-optNYESOTCR/TK lentiviral vector (a third generation lentiviral vector referred to as LV-NYESO TCR/sr39TK PBSC; ≥ 2 up to 6 x 10^6^ CD34+ cells/kg) and a dose of up to 10^9^ autologous PBMCs transduced with the MSGV1-A2aB-1G4A-LY3H10 retroviral vector (referred to as RV-NYESO TCR PBMC)(Day 1). Both vectors express a high affinity TCR for the NY-ESO-1 cancer-testes antigen (NY-ESO-1 TCR). The lentiviral vector also expresses the marker gene, sr39 thymidine kinase (sr39TK). The LV-NYESO TCR/sr39TK PBSC will be thawed immediately prior to infusion on Day 0 whereas the RV-NYESO TCR PBMC will be infused fresh on the day of harvest (Day 1). The LV-NYESO TCR/sr39TK PBSC will be infused first approximately 24 hours prior to the RV-NYESO TCR PBMC infusion to allow for identifying reactions to either one of the two products. Additional unmodified PBSC will be collected and banked in case of engraftment failure. Following ACT (Day 0-1), patients will receive low dose interleukin-2 (IL-2) (Days 2-8).

The retroviral NY-ESO-1 TCR vector was manufactured at clinical grade at the Indiana University Vector Production Facility (IUVPF) based on a master cell bank that was provided by Drs. Steven A. Rosenberg and Paul F. Robbins from the Surgery Branch, National Cancer Institute (NCI). This MSGV1-A2aB-1G4A-LY3H10 TCR contains two amino acid substitutions in the third complementarity determining region (CDR) of the native 1G4-TCR alpha chain that confers an increased ability of CD4+ and CD8+ T cells to recognize the HLA-A*0201/NY-ESO-1_157-165_ complex while maintaining antigen specificity ([Robbins et al., 2008](#_ENREF_89)). This vector was used in a clinical trial run by the NCI (NCT00670748)([Robbins et al., 2011b](#_ENREF_91)). Moreover, there are several current clinical trials using adoptive transfer of T cells specific for NY-ESO-1 in the setting of advanced malignancies, in adults and in children (NCT01967823, NCT02062359, NCT01343043, NCT02319824, NCT01350401, NCT02059850, NCT01352286 and NCT01892293). In addition, at University of California Los Angeles (UCLA), we have three clinical trials open using an NY-ESO-1 TCR transgenic ACT protocol (IND# 15167: NCT01697527, NCT02070406 and NCT02775292). Both the NCI clinical trial and our current trials utilize the same retrovirus expressing the NY-ESO-1 TCR to transduce whole PBMC that are re-infused to patients after a lympho-depleting chemotherapy conditioning regimen. Major differences between the clinical trials at UCLA compared to NCI include the shorter *ex vivo* expansion of TCR transduced PBMC and using low dose IL-2 instead of the high dose IL-2 employed in the National Cancer Institute (NCI) trial.

The lentiviral LV-optNYESOTCR/TK vector has also been manufactured at clinical grade at the IUVPF. This vector consists of 9417 base pairs including the third generation lentiviral self-inactivating (SIN) long terminal repeats (LTRs), internal promoter from the murine stem cell virus (MSCV), packaging signal with the splicing donor and splicing acceptor sites, alpha chain and beta chain genes of the NY-ESO-1 TCR from tumor infiltrating lymphocyte (TIL) clone 1G4 α95:LY, and the positron emission tomography (PET) tracer marker/suicide gene sr39TK. The alpha and beta TCR chains and the sr39TK genes are linked by 2A self-cleaving sequences (P2A and T2A, respectively).

In this trial, the RV-NYESO TCR PBMC will be co-administered with LV-NYESO TCR/sr39TK PBSC to patients after a reduced intensity conditioning regimen. The primary endpoint is safety. Secondary and exploratory study endpoints are feasibility, efficacy based on standard response evaluation criteria in solid tumors (RECIST), persistence of TCR transduced PBMC and PBSC in humans and ability of the transduced PBMC and progeny of transduced PBSC to home to NY-ESO-1 positive malignancies, engraft and be functional. Analyses of secondary and exploratory endpoints will be performed by physical exam and computed tomography (CT) imaging scans, sampling of peripheral blood, tissue biopsy and PET scans for T cell persistence.

## Study Rationale

**Rationale for TCR Transduced ACT Therapy.** The adoptive transfer of large numbers of clonally-expanded TILs into patients with advanced malignancies that have received a lympho-depleting conditioning regimen together with high doses of IL-2, resulted in the highest rate of melanoma responses (50%) reported to date ([Dudley et al., 2002a](#_ENREF_33); [Dudley et al., 2005](#_ENREF_35)). This approach provides a proof-of-principle for adoptive cell transfer immunotherapy for metastatic melanoma, but it is difficult to implement outside of pilot studies given its requirement for extensive *ex vivo* manipulations. This roadblock may be overcome and may also be extended to other malignancies by the *ex vivo* transduction of specific TCR genes that recognize tumor associated antigens. In this project, we will test the hypothesis that the co-administration of human PBMC engineered to express a high affinity TCR specific for the human cancer-testes specific antigen NY-ESO-1 combined with CD34+ PBSC expressing the same TCR is safe and allows for persistence of the TCR transduced cells.

**Rationale for NY-ESO-1 as the Tumor Antigen Target for Gene Engineered ACT.** NY-ESO-1 is a cancer-testis antigen that is not expressed in somatic normal tissues in adult life, except for major histocompatibility complex (MHC) negative germinal cells. It is re-expressed in a significant percentage of all cancers, including advanced synovial sarcomas at high levels. It is a validated target for gene engineered ACT based on the experience at the NCI surgery branch and a multicenter clinical trial by Adaptimmune ([Rapoport et al., 2015](#_ENREF_85); [Robbins et al., 2015](#_ENREF_88); [Robbins et al., 2011a](#_ENREF_90)). Both experiences used the same TCR for NY-ESO-1 that is proposed in this protocol. Given the safety and antitumor efficacy of this TCR, we will use it to test our hypothesis of the safety and feasibility to insert a tumor-specific TCR into CD34+ PBSC to provide a continuous output of fully functional antitumor TCR transgenic T cells.

**Rationale for ACT of TCR transduced T cells combined with TCR transduced hematopoietic stem cells.** The main limitation of immunotherapy strategies utilizing ACT of T cells transduced with a tumor antigen specific TCR is usually the extremely low number of tumor-reactive T cells found in the periphery shortly after the transfer to the patient. Our emerging clinical data demonstrates that the ACT of TCR transduced mature lymphocytes results in an initial wave of highly active and tumor-killing lymphocytes. But within weeks, these cells lose antitumor effector functions and also decrease in frequency ([Chodon et al., 2014](#_ENREF_25)). This experience points to the need to provide a continuous source of the TCR transduced cells to maintain their frequency and function.

In this study, we hypothesize that TCR transduced PBSCs will allow a sustained production of fully active T cells with antitumor activity. This hypothesis is supported by original preclinical data from our group ([Vatakis et al., 2011](#_ENREF_111); [Yang and Baltimore, 2005](#_ENREF_121); [Yang et al., 2002](#_ENREF_122)) and by the data from our ongoing TCR transduced adoptive cell transfer (ACT) clinical trials ([Ma et al., 2011](#_ENREF_68)). As pioneered by Yang and Baltimore ([Yang and Baltimore, 2005](#_ENREF_121); [Yang et al., 2002](#_ENREF_122)) and confirmed by Vatakis and Zack ([Vatakis et al., 2011](#_ENREF_111)), TCR transduced PBSCs endogenously differentiate into fully active mature T cells. However, there is a delay in their appearance in the periphery. Therefore, we plan to conduct a clinical trial in which TCR transduced mature lymphocytes and TCR transduced PBSCs will be co-administered to patients with advanced cancer after a reduced intensity conditioning regimen. We hypothesize that the TCR transduced mature lymphocytes will expand *in vivo* and provide a first wave of transient antitumor activity, which will provide a bridge until the second wave of TCR transduced cells arising from the bone marrow engrafted gene modified PBSCs has gone through the T lymphocyte maturation process and partially repopulated the peripheral tissues.

**Rationale for Cell Dose of TCR Transduced CD34+ PBSC.** The dose of gene modified CD34+ PBSC utilized for the treatment of several hereditary disorders has been variable between studies ([Russell et al., 2002](#_ENREF_96); [Ryu et al., 2007](#_ENREF_97); [Sato et al., 2015](#_ENREF_99); [Xhaard et al., 2014](#_ENREF_116)). The dose for this trial is based on the minimum number of CD34+ cells necessary for hematopoietic reconstitution and the feasible number of CD34+ cells that could be obtained with a standard G-CSF mobilization protocol. Many studies report that G-CSF mobilization in healthy donors usually allows collecting ≥ 2.0×10^6^ CD34+ cells/ kg body weight in one to two leukapheresis collections and at least 2.0×10^6^ CD34+ cells/ kg body weight is sufficient for hematopoietic reconstitution ([Link, 1996](#_ENREF_66)). Therefore, we set our minimum amount of cells per cohort as 2.0 ×10^6^/kg CD34+ PBSC and the maximum amount of cells as 6.0 ×10^6^/kg CD34+ PBSC, assuming that more than one leukapheresis will be required.

Our preclinical studies in mice using transduced stem cell equivalent cells, termed lineage negative (Lin-), indicate that 1.0×10^6^ NY-ESO-1 TCR transduced Lin- cells per mouse with a vector copy number ranging from 0.3-3 did not alter mouse survival and led to similar reconstitution of lymphoid and myeloid hematopoietic lineages as the control group injected with 1.0×10^6^ mock transduced Lin- cells. However, it should be noted that it is not possible to directly extrapolate the cell number from mice to humans since Lin- cells in mice are not entirely equivalent to CD34+ cells in humans.

**Rationale for Administration of Transduced PBSCs with Transduction Efficiency of Less Than 50%.** It has been demonstrated in studies in humanized, HLA-A*0201-expressing mice (NSG-A2.1) that transduction of human CD34^+^ PBSC with a TCR transgene caused a high degree of allelic exclusion, potently suppressing rearrangement of endogenous human TCR-β genes during thymopoiesis ([Giannoni et al., 2013](#_ENREF_41)). In order to ensure that the repertoire of endogenous TCRs will not be compromised as a result of this suppression, at least 50% of the CD34+ PBSC delivered to the patient will be untransduced. The transduction efficiency will be assessed prior to delivery and confirmed to be less than 50%. While it is unlikely that the transduction efficiency is greater than 50%, in that scenario, the cells will either be diluted or will not be administered.

**Rationale for Cell Dose of TCR Transduced PBMC.** A phase 2 clinical trial conducted at the Surgery Branch/NCI reported using a cell dose of up to 10^11^ NY-ESO-1 TCR transduced PBMC with no toxicities attributed to the transgenic cells ([Robbins et al., 2011a](#_ENREF_90)). At UCLA, the number of cells infused has been up to 10^10^ cells in a similar trial using MART-1 F5 TCR transduced cells (IND #13589). This was in combination with high-dose IL-2 and 5 days of conditioning chemotherapy. However, due to toxicities seen with this combination, the number of infused cells was decreased to 10^9^ in the MART-1 F5 study and in the successive NY-ESO-1 TCR studies (IND #15167).

**Rationale for the Reduced Intensity Conditioning Chemotherapy Regimen.** In this study, we propose to use a reduced intensity conditioning regimen (IND 16141; Sponsor Kohn, UCLA)([Alyea et al., 2006](#_ENREF_3); [Ben-Barouch et al., 2016](#_ENREF_9); [Chae et al., 2007](#_ENREF_21); [de Lima et al., 2004](#_ENREF_30); [Rambaldi et al., 2015](#_ENREF_84)). The patient population for this trial has typically undergone multiple lines of chemotherapy and often have a low bone marrow reserve. Moreover, the goal of the full intensity myeloablation in the regular autologous or allogeneic transplant setting is to kill the cancer cells (multiple myeloma, lymphoma and leukemia) and is followed by marrow rescue. We do not propose a full intensity lympho-depleting myeloablative conditioning regimen, as it would be more detrimental to this solid tumor patient population. The goal of the reduced intensity conditioning chemotherapy in this trial is NOT to control the disease but rather to “make space” for the transduced PBSC to engraft the marrow and for the adoptive transfer of the transduced PBMC. This type of regimen consisting of 12 mg total of busulfan and 40 mg/m^2^/day fludarabine over 4 days is adequate to facilitate engraftment of transduced CD34+ cells and adoptive transfer of transduced T cells.

**Rationale for low dose IL-2.** Compared to the NCI trials that used high dose IL-2 post adoptive transfer, this trial uses low dose subcutaneous administration of IL-2. This modification is based on data which indicated that IL-2 receptors are saturated at low doses of IL-2 and that adoptively transferred T cell clones can persist *in vivo* in response to low-dose IL-2. These T cells preferentially localize to tumor sites and mediate an antigen-specific immune response characterized by the elimination of antigen positive tumor cells and regression of individual metastases ([Mackensen et al., 2006](#_ENREF_69); [Yee et al., 2002](#_ENREF_123)). Furthermore, there is evidence that high dose IL-2 may impair the generation of effector memory T cells ([June, 2007](#_ENREF_51)).

Subjects will receive two staggered intravenous infusions of the transduced cells, the first with LV-NYESO TCR/sr39TK PBSC with a vector copy number (VCN) within the range of 0.1–2.0 per cell, and the second with RV-NYESO TCR PBMC. If the VCN is <0.1, then the cells will not be administered. The cell doses are as follows:

- Up to 1.0 ×10^9^ RV-NYESO TCR PBMC
- ≥2.0 x 10^6^ to up to 6.0 ×10^6^ LV-NYESO TCR/sr39TK PBSC/kg

**Rationale for the introduction of sr39TK suicide gene in the lentiviral vector.** The inclusion of the PET imaging/suicide gene herpes simplex virus-1 HSV-1-sr39TK (sr39TK) allows for both the non-invasive tracking of progeny derived from gene modified PBSC to assess their biodistribution as well as their ablation in the very unlikely event of off target reactivity or hematopoietic dysplasia due to insertional mutagenesis of gene modified PBSC. The mutated sr39TK gene is more efficient than the parental gene in the utilization of acycloguanosines as substrates ([Black et al., 1996](#_ENREF_13)), improving the imaging of sr39TK genetically labeled cells using microPET imaging. The sr39TK genetic cell labeling approach was originally developed by UCLA investigators led by Harvey Herschman and Sanjiv Gambhir, and continued after Dr. Gambhir moved to Stanford ([Gambhir et al., 1998](#_ENREF_39); [Gambhir et al., 2000](#_ENREF_40); [Yaghoubi et al., 2005](#_ENREF_118); [Yaghoubi et al., 2009](#_ENREF_120)). Studies by UCLA investigators Owen Witte and Jonathan Braun demonstrated that sr39TK genetic marking could be used to image antitumor T cell responses *in vivo* in animal models ([Dubey et al., 2003](#_ENREF_32); [Kim et al., 2004b](#_ENREF_56); [Lee et al., 2005](#_ENREF_64); [Prins et al., 2008](#_ENREF_81); [Radu et al., 2007](#_ENREF_83); [Shu et al., 2005](#_ENREF_104); [Shu et al., 2009](#_ENREF_105)). HSV-1-sr39TK can be detected using the experimental PET tracer [^18^F]-FHBG ([Yaghoubi et al., 2009](#_ENREF_120)). In a preclinical study, [^18^F]FHBG PET imaging of the humanized NSG-A2.1 mouse model allowed the detection of NY-ESO-1 TCR/sr39TK positive cells in the marrow compartments of mice, namely the long bones of the legs and arms. Importantly, a strong signal in the thymus was also observed indicating a robust thymic population/thymopoiesis of gene modified cells. No specific signal was detected in NSG-A2.1 mice transplanted with mock transduced PBSC or in non-transplanted NSG-A2.1 mice ([Gschweng et al., 2014](#_ENREF_46)).

To examine the suicide gene function of the sr39TK cassette, we treated engineered humanized mice with vehicle control or ganciclovir, the prodrug for suicide gene function of sr39TK. Ganciclovir treatment resulted in ablation of the [^18^F]FHBG PET signal, while vehicle control did not. Mice were subsequently euthanized, and the presence of gene modified cells in organ compartments was examined by digital droplet polymerase chain reaction (PCR) for the lentiviral psi packaging element. We detected an order of magnitude decrease in the amount of gene modified cells in ganciclovir treated animals compared with controls ([Gschweng et al., 2014](#_ENREF_46)). These data suggest that the gene modified PBSC and their progeny can be localized in patients. Moreover, ganciclovir can be used to ablate the gene modified PBSCs and their progeny if necessary using the suicide function of the sr39TK transgene.

## Study Objectives

**Primary:**

- SAFETY. To determine the safety of administering the combination of autologous PBMC and CD34+ PBSC following a reduced intensity conditioning regimen, both of which have been genetically modified to express NY-ESO-1 TCR.

**Secondary:**

- FEASIBILITY. To determine the feasibility of delivering the combination of TCR transduced autologous PBMC and CD34+ PBSC to patients.
- NY-ESO-1 TCR TRANSGENIC CELL PERSISTENCE. To determine the persistence of NY-ESO-1 TCR transduced PBMC and the progeny of TCR transduced PBSC in serial peripheral blood samples.
- CLINICAL RESPONSE. Objective response rate (ORR) will be a secondary endpoint in this pilot clinical trial. ORR is determined by RECIST criteria (version 1.1), which is defined as adding the rate of complete response (CR) and partial response (PR) at restaging exams. To account for the frequently observed delayed responses with immunotherapy, patients with transient disease stabilization or progression at day 60 who do not receive further therapy but go on to meet RECIST criteria for CR or PR, will also be considered as having an objective response on study.

**Exploratory:**

- *IN VIVO* MOLECULAR IMAGING. To explore the use of PET-based imaging using the PET tracer 9-4-[^18^F]fluoro-3-(hydroxymethyl)butylguanine ([^18^F]FHBG) with the goal of determining whether the adoptively transferred NY-ESO-1 TCR transduced PBSC home to bone marrow, differentiate into T cells and expand in secondary lymphoid organs and tumor deposits. Findings from non-invasive PET imaging will be compared with results from immune monitoring assays in blood samples at different intervals after NY-ESO-1 TCR ACT.
- CLINICAL RESPONSE PER iRECIST. Immunotherapy and cellular therapy interventions against cancer can lead to unique response patterns, including durable stable disease, delayed response after initial tumor burden increase (flare response), and regression of a target lesion with appearance of new lesions. In order to classify these, we will also classify tumor responses per iRECIST ([Seymour et al., 2017](#_ENREF_103)). In brief, this will reset the classification if RECIST Progressive Disease (PD) is followed at next time point by tumor shrinkage, with new overall response defined as “iUPD” (immune unconfirmed progressive disease). iCPD (immune confirmed progressive disease) would then be confirmed by repeat imaging 4-8 weeks later.

## Study Population

Subjects must be HLA-A*0201, have locally advanced (unresectable stage IIIc) or metastatic malignancies (stage IV), whose tumor express NY-ESO-1, and who have exhausted (or ineligible) all current available treatment options, age greater than or equal to 16, clinical performance status of ECOG 0 or 1, no active autoimmune diseases, and be seronegative for HIV, Hepatitis B and C (seropositive subjects would be more likely to have toxic effects from the conditioning chemotherapy). Patients should not have received any systemic treatment for cancer within 30 days of starting conditioning chemotherapy in this trial. Patients must have adequate bone marrow and major organ function to undergo a PBSC transplant.

## Trial Design and Number of Patients

This phase 1 clinical trial includes one dosing regimen of RV-NYESO TCR PBMC and LV-NYESO TCR/sr39TK PBSC.

One cohort of 3 subjects will be enrolled initially.

- After the first 3 subjects are dosed, the cohort will be expanded to 6 subjects.
- If less than 2/6 Dose Limiting Toxicities (DLTs) are observed, the treatment will be considered safe.
- If DLTs are observed in 2 or more of 6 subjects, then the treatment will have exceeded the 33% DLT rate, and the study will be terminated.

If a subject withdraws early from participation voluntarily without a DLT or due to disease progression before the first 60 days of the DLT observation period, the subject will be substituted to allow adequate assessment of safety.

There will be a staggered enrollment of subjects as follows:

- All enrollees will start on study with a minimum of 1 week between Day 0 (gene-modified stem cell administration) of a subject and Day -5 (unmobilized leukapheresis for collection of cells for gene-modified T cell manufacture) of the next subject.
- There will be at least 3 months between Day 0 for the first subject and enrollment of the second subject.

All subjects will receive the same combined therapy of: 1) a reduced intensity conditioning regimen (busulfan and fludarabine), 2) autologous RV-NYESO TCR PBMC, 3) autologous LV-NYESO TCR/sr39TK PBSC and 4) low dose IL-2. This is a single center, open label, phase 1 safety study as described in Table 1.

Table 1. Description of the dose and schedule of the proposed therapy combining PBMC with PBSC.

| **Number of patients** | **Conditioning** | **RV-NYESO TCR PBMC dose** | **LV-NYESO TCR/sr39TK PBSC dose^a,b^** | **Unmodified PBSC (Cryopreserved Back Up)^b^** | **IL-2 dose and schedule** |
| --- | --- | --- | --- | --- | --- |
| 3-6 | Busulfan and Fludarabine | Up to 1 x 10^9^ i.v. x 1 | ≥2 – 6 x 10^6^ CD34+ Tg^d^ cells/kg i.v. x 1 | At least 2.0 x 10^6^ PBSC/kg | 500,000 IU/m^2^ q12 hours for a maximum of 14 doses |

^a^Target cell dose. If less than the planned number of CD34+ transgenic cells is obtained, subject continuation on the study will be decided on an individual basis by the study investigators.

^b^The transduction efficiency must be less than 50%. If it is greater than 50%, then the cells will either be diluted with unmodified PBSC or will not be administered. At least 2.0 x 10^6^ unmodified cells/kg will be banked in case of engraftment failure.

^c^Abbreviations: Tg = transgenic

At the completion of this trial when safety has been established, a separate Phase 1b or Phase 2 study will be designed to evaluate more subjects to provide more information regarding safety and efficacy.

## Study Duration

Total duration of the study is estimated to be approximately 36 months if recruitment proceeds optimally with 20 patients screened every year, resulting in approximately 2 patient entered in the trial every year.

## Study Schematic

Figure 1. Clinical Trial Overview

Figure 2. Stem Cell Mobilization and Manufacture Scheme

Figure 3. T Cell Manufacture, Cell Administration Through Study Observation Period


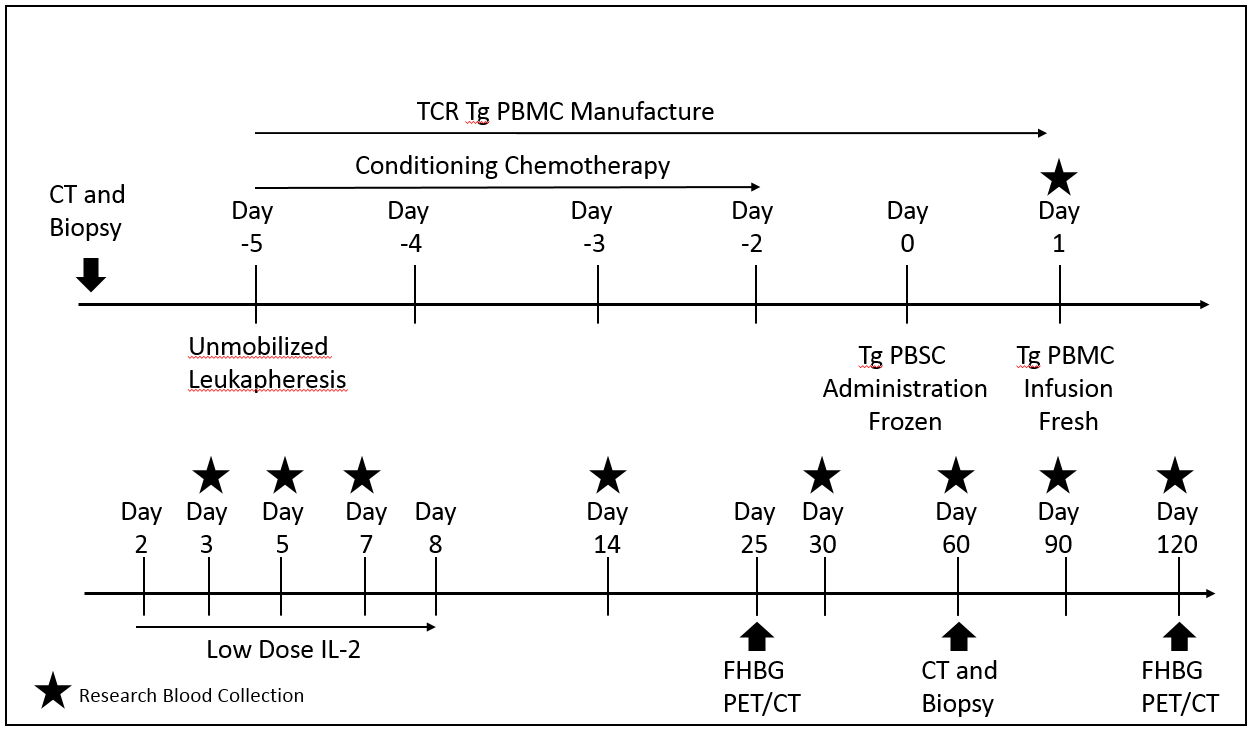


Patients will be selected on the basis of inclusion and exclusion criteria as detailed in section 7.1 and 7.2. Six months to 3 weeks prior to infusion of transduced cells, patients will undergo G-CSF and plerixafor mobilization of PBSC, that will be obtained by leukapheresis. G-CSF (8-10 µg/kg/day) will be administered to patients subcutaneously on mobilization days (mDay) 1-4 in the morning. On the evening of mDay 4, patients will begin receiving plerixafor (0.24 mg/kg/day subcutaneously). Plerixafor will be administered the evening before leukapheresis in the morning of mDay 5. Daily administration of G-CSF in the morning and plerixafor in the evening will continue until a sufficient number of cells for backup and transduction are collected up to mDay 8.

At least 2.0 x10^6^ CD34+ un-manipulated cells/kg (without CD34+ cell isolation) must be obtained for back up cells. Backup cells will be cryopreserved in liquid nitrogen vapors (≤ - 140°C) in the UCLA Bone Marrow/Stem Cell Transplant Laboratory according to the their SOP for possible future use if there is lack of engraftment for up to 2 years after collection ([Figure 2](#Figure2)). If the cells are not used within 2 years of collection, the cells will be de-identified and used for research.

Mobilized cells will be collected for transduction over a maximum of 3 days up to mDay 8. Since CD34+ cells can only be stored for 24 hours prior to transduction at room temperature or for 48 hours at 4°C, the patient’s participation in the trial will be terminated if a sufficient number of cells are not obtained within the 3 day period. At least ≥2.5 x 10^6^ CD34-enriched cells/kg are necessary for transduction with LV-optNYESOTCR/TK lentiviral vector to obtain the required final number of transduced cells.

If a sufficient number of cells are obtained, CD34+ PBSC will be isolated using the Miltenyi CliniMACS system, stimulated, transduced with the LV-optNYESOTCR/TK lentiviral vector and cryopreserved until ready for use after lot release testing (see 10.0). These transduced PBSC will be referred to as LV-NYESO TCR/sr39TK PBSC from this point in the manufacture forward. The transduction efficiency of LV-NYESO TCR/sr39TK will be assessed by average vector copy number (VCN), Vb13.1 (TCR β chain variant of NY-ESO-1 TCR) expression and percentage of transduced colony forming units (CFU). These assays require approximately 3 weeks. Transduction efficiency greater than 50% is a rare event. This has not occurred in any of the 5 manufacturing validation runs to date. If the transduction efficiency is greater than 50%, then the cells will either be diluted with unmodified cryopreserved PBSC (if available), will not be administered or the subject will be mobilized again to obtain additional PBSC with which to dilute the transduced cells.

After CliniMACS purification, if a sufficient number of CD34-enriched cells are not obtained, the patient’s participation in the trial will be terminated as the CD34+ cells can only be stored at room temperature for 24 hours or at 4°C for 48 hours prior to transduction.

If the transduced PBSC meet the lot release criteria and after the transduction efficiency of the LV-NYESO TCR/sr39TK PBSC has been determined, patients will then undergo an unmobilized partial leukapheresis to collect PBMC, which will be transduced with the NY-ESO-1 TCR retroviral vector (MSGV1-A2aB-1G4A-LY3H10). The manufacture of transduced T cells will be started on the day of leukapheresis. Immediately after processing the PBMC, approximately 1.5 x 10^9^ PBMC will be put in activation media containing OKT3 (anti-CD3 antibody) and IL-2 for two days, and the rest will be cryopreserved. While the transduced T cells are being manufactured and lot release testing is being performed, patients will undergo a conditioning regimen with busulfan and fludarabine. Following activation, PBMC will be transduced twice on two consecutive days with the MSGV1-A2aB-1G4A-LY3H10 retrovirus in retronectin-coate plates or bags. Transduced cells will be expanded *ex vivo* for 4 days after the first transduction, and then put into an infusion bag (or kept in bags if transduced in bags) to be infused i.v. fresh on the day of harvest as soon as the lot release is cleared. The transduced PBMC will be referred to as RV-NYESO TCR PBMC from this point in the manufacture forward.

Patients will be infused with LV-NYESO TCR/sr39TK PBSC on Day 0 and RV-NYESO TCR PBMC on Day 1. Beginning on Day 2, patients will receive subcutaneous (SC) low dose IL-2 twice daily for up to 7 days (maximum of 14 doses). Peripheral blood will be collected throughout the study for safety and immune monitoring. CT scans will be performed for antitumor activity and PET scans for transduced mature T cell and stem cell persistence and biodistribution (Figure 3). At least 5 ml of blood will be collected on days 0, 1, 3, 5, 7, 14, and 30 after ACT, and plasma derived from these samples will be cryopreserved and archived for cytokine analyses if necessary. Sixty to 100 ml of blood will be collected on days 30, 60, 90 and 120 for immune monitoring.

Subjects will be evaluated for antitumor activity after undergoing re-staging exams (RECIST criteria) at Day 60. PET/CT scans using [^18^F]FHBG as a tracer will be performed to explore the use of molecular imaging to assess the persistence and biodistribution of the stem cells and the transgenic progeny cells on Day 25 and 120 post-ACT. Biopsies will be performed whenever feasible to assess persistence of the transduced mature T cells, stem cells and their progeny at Day 60.

Clinical follow up will include an initial 2 years of active follow up in which subjects will be seen every 2-3 months followed by long term follow up for up to 15 years.

## Experimental Study Agents

### Retrovirus vector MSGV1-A2aB-1G4A-LY3H10

The retroviral vector, MSGV1-A2aB-1G4A-LY3H10, was generated at the Surgery Branch/NCI by Drs. Paul Robbins and Steven Rosenberg. This vector contains the 5’ and 3’ LTR of the murine stem cell virus (MSCV) as well as a packaging signal containing splicing donor and acceptor sites. In this TCR vector, the alpha and beta chains of the NY-ESO-1 TCR are separated by a P2A “self-cleaving” sequence. The particular vector that is being used in this trial contains a modified version of the parental 1G4 vector in which the amino acids 95 and 96 of the CDR3 of the alpha chain have been modified to leucine (L) and tyrosine(Y) ([Robbins et al., 2008](#_ENREF_89))([See 9.1.1 for map](#_Retrovirus_Vector_MSGV1-A2aB-1G4A-L)).

### Lentivirus vector pRRL-MSCV-optNYESO optsr39TK-WPRE-1 (LV-optNYESOTCR/TK)

The lentiviral vector LV-optNYESOTCR/TK was generated in the Ribas laboratory. The vector is composed of alpha and beta chains of the NY-ESO-1 TCR and the PET tracer marker/suicide gene sr39TK. The alpha and beta TCR chains and the sr39TK genes are linked by self-cleaving sequences P2A and T2A, respectively. The vector expression is driven by the murine stem cell virus (MSCV) promoter. The functionality and safety of this vector have been addressed in our pre-clinical studies ([See 9.2.1 for map](#_RV-NYESO_TCR_PBMC)).

## Timing of Events

To account for the pilot investigational nature of the procedures in this protocol and anticipated problems with patient scheduling, timing of events are approximate and the study team will attempt to perform them on the dates included in this protocol, but variations on these dates will not be considered protocol violations.

# Schedule of Events

|  | **Screening** | **Day**  **-180 to -21** | **Day -5** | **Day –4** | **Day –3** | **Day -2** | **Day 0** | **Day 1** | **Day**  **2 to 8 (daily)** | **Day 3** | **Day 5** | **Day 7** | **Day**  **14** | **Day 25** | **Day**  **30** | **Day**  **45** | **Day**  **60** | **Day**  **75** | **Day**  **90** | **Day 120** | **Q2-3 mo up to Year 2 ^c^** | **Q6 mo up to Year 5^c^** | **Q12 mo up to Year 15^c^** |
| --- | --- | --- | --- | --- | --- | --- | --- | --- | --- | --- | --- | --- | --- | --- | --- | --- | --- | --- | --- | --- | --- | --- | --- |
| Informed Consent | X |  |  |  |  |  |  |  |  |  |  |  |  |  |  |  |  |  |  |  |  |  |  |
| Visit and Physical | X |  | X |  |  |  |  |  |  |  |  |  |  |  | X | X | X | X | X | X | X | X | X |
| HLA Typing | X |  |  |  |  |  |  |  |  |  |  |  |  |  |  |  |  |  |  |  |  |  |  |
| PCR or IHC for NY-ESO-1 | X |  |  |  |  |  |  |  |  |  |  |  |  |  |  |  |  |  |  |  |  |  |  |
| CBC | X |  | X^b^ |  |  |  |  |  |  |  |  |  |  |  | X | X | X | X | X | X |  |  |  |
| Complete Metabolic Panel/Direct Bilirubin | X |  | X^b^ |  |  |  |  |  |  |  |  |  |  |  | X | X | X | X | X | X |  |  |  |
| LDH | X |  |  |  |  |  |  |  |  |  |  |  |  |  | X | X | X |  |  | X |  |  |  |
| CRP | X |  |  |  |  |  |  |  |  |  |  |  |  |  |  |  |  |  |  | X |  |  |  |
| Uric Acid | X |  | X |  |  |  |  |  |  |  |  |  |  |  |  |  |  |  |  |  |  |  |  |
| Magnesium | X |  | X |  |  |  |  |  |  |  |  |  |  |  |  |  |  |  |  |  |  |  |  |
| Phosphorous | X |  | X |  |  |  |  |  |  |  |  |  |  |  |  |  |  |  |  |  |  |  |  |
| Coagulation Tests PT/INR, PTT | X |  |  |  |  |  |  |  |  |  |  |  |  |  |  |  |  |  |  | X |  |  |  |
| Autoantibody Panel | X |  |  |  |  |  |  |  |  |  |  |  |  |  |  |  |  |  |  | X |  |  |  |
| Thyroid Hormones | X |  |  |  |  |  |  |  |  |  |  |  |  |  |  |  |  |  |  | X |  |  |  |
| Pregnancy Test^a^ | X |  | X |  |  |  |  |  |  |  |  |  |  |  |  |  |  |  |  |  |  |  |  |
| Infectious disease serologies | X |  |  |  |  |  |  |  |  |  |  |  |  |  |  |  |  |  |  |  |  |  |  |
| G-CSF and Plerixafor Administration (see Table 3) |  | X^d^ |  |  |  |  |  |  |  |  |  |  |  |  |  |  |  |  |  |  |  |  |  |
| G-CSF-mobilized Leukapheresis (see Table 3) |  | X^d^ |  |  |  |  |  |  |  |  |  |  |  |  |  |  |  |  |  |  |  |  |  |

Table 2. Schedule of Events

|  | **Screening** | **Day**  **-180 to -21** | **Day -5** | **Day –4** | **Day –3** | **Day -2** | **Day -1** | **Day 0** | **Day 1** | **Day**  **2 to 8 (daily)** | **Day 3** | **Day 5** | **Day 7** | **Day**  **14** | **Day 25** | **Day**  **30** | **Day**  **45** | **Day**  **60** | **Day**  **75** | **Day**  **90** | **Day 120** | **Q2-3 mo up to Year 2 ^c^** | **Q6 mo up to Year 5^c^** | **Q12 mo up to Year 15^c^** |
| --- | --- | --- | --- | --- | --- | --- | --- | --- | --- | --- | --- | --- | --- | --- | --- | --- | --- | --- | --- | --- | --- | --- | --- | --- |
| Supportive care during G-CSF therapy |  | X^d^ |  |  |  |  |  |  |  |  |  |  |  |  |  |  |  |  |  |  |  |  |  |  |
| Unmobilized Partial Leukapheresis |  |  | X |  |  |  |  |  |  |  |  |  |  |  |  |  |  |  |  |  |  |  |  |  |
| Partial Plasmapheresis |  | X | X |  |  |  |  |  |  |  |  |  |  |  |  |  |  |  |  |  |  |  |  |  |
| Unmobilized Partial Leukapheresis/  Plasmapheresis |  |  |  |  |  |  |  |  |  |  |  |  |  |  |  |  |  |  |  |  | X |  |  |  |
| Blood for Protein Analyses (cytokines – at least 5 ml) | X |  |  |  |  |  |  | X^i^ | X |  | X | X | X | X |  | X |  |  |  |  |  |  |  |  |
| Blood for Cell Analyses (Immune monitoring – 60-100ml)^f^ | X |  |  |  |  |  |  |  |  |  |  |  |  |  |  | X | X | X | X | X | X | X | X | X |
| ECHO | X |  |  |  |  |  |  |  |  |  |  |  |  |  |  |  |  |  |  |  |  |  |  |  |
| ECG | X |  |  |  |  |  |  |  |  |  |  |  |  |  |  |  |  |  |  |  |  |  |  |  |
| PFT | X |  |  |  |  |  |  |  |  |  |  |  |  |  |  |  |  |  |  |  |  |  |  |  |
| CAP PET/CT | X |  |  |  |  |  |  |  |  |  |  |  |  |  |  |  |  |  |  |  |  |  |  |  |
| CAP CT and MRI^g^ |  |  |  |  |  |  |  |  |  |  |  |  |  |  |  |  |  | X |  |  |  | X | X |  |
| FHBG-PET/CT Low Resolution^g^ |  |  |  |  |  |  |  |  |  |  |  |  |  |  | X |  |  |  |  |  | X |  |  |  |
| Catheter Placement | X | | | | | | | |  |  |  |  |  |  |  |  |  |  |  |  |  |  |  |  |
| Inpatient admission |  |  | X |  |  |  |  |  |  |  |  |  |  |  |  |  |  |  |  |  |  |  |  |  |
| Supportive Care During Chemotherapy |  |  |  | X | | | | |  |  |  |  |  |  |  |  |  |  |  |  |  |  |  |  |

|  | **Screening** | **Day**  **-180 to -21** | **Day -5** | **Day –4** | **Day –3** | **Day -2** | **Day -1** | **Day 0** | **Day 1** | **Day**  **2 to 8 (daily)** | **Day 3** | **Day 5** | | **Day 7** | **Day**  **14** | **Day 25** | **Day**  **30** | **Day**  **45** | **Day**  **60** | **Day**  **75** | **Day**  **90** | **Day 120** | **Q2-3 mo up to Year 2 ^c^** | **Q6 mo up to Year 5^c^** | **Q12 mo up to Year 15^c^** |
| --- | --- | --- | --- | --- | --- | --- | --- | --- | --- | --- | --- | --- | --- | --- | --- | --- | --- | --- | --- | --- | --- | --- | --- | --- | --- |
| Busulfan^k^ |  |  | X | X | X | X |  |  |  |  |  |  |  | |  |  |  |  |  |  |  |  |  |  |  |
| Blood for busulfan Level^j,k^ |  |  |  | X |  | X |  |  |  |  |  |  |  | |  |  |  |  |  |  |  |  |  |  |  |
| Fludarabine^k^ |  |  | X | X | X | X |  |  |  |  |  |  |  | |  |  |  |  |  |  |  |  |  |  |  |
| Prophylactic Supportive Care During Pancytopenic Period |  |  |  | X | | | | | | | | | | | | |  |  |  |  |  |  |  |  |  |
| NY-ESO-1 TCR/sr39TK PBSC Infusion |  |  |  |  |  |  |  | X^h^ |  |  |  |  |  | |  |  |  |  |  |  |  |  |  |  |  |
| NY-ESO-1 TCR PBMC Infusion |  |  |  |  |  |  |  |  | X |  |  |  |  | |  |  |  |  |  |  |  |  |  |  |  |
| Low dose IL-2 |  |  |  |  |  |  |  |  |  | X |  |  |  | |  |  |  |  |  |  |  |  |  |  |  |
| IVIG administration |  |  |  |  |  |  |  |  |  | X |  |  |  | | X | X | X | X | X | X | X |  |  |  |  |
| Blood Viral Copy Detection (PCR)^h^ |  |  |  |  |  |  |  |  |  | X |  |  |  | | X | X | X | X | X | X | X |  |  |  |  |
| Biopsy | X |  |  |  |  |  |  |  |  |  |  |  |  | |  |  |  |  | X |  |  |  |  |  |  |
| RCR and RCL Testing |  |  |  |  |  |  |  |  |  |  |  |  |  | |  |  |  |  |  |  |  |  | X | X | X |

^a^Females only. Repeat pregnancy test on day of hospital admission to ensure that the patient is not pregnant prior to initiation of chemotherapy.

^b^ Patients will be in the hospital. Throughout the inpatient hospital stay, physical, CBC and metabolic panel will be performed per J-Medicine protocol.

^c^Long term follow up is described in section 8.14. Collection of blood for transgenic cell persistence, RCR and RCL testing and insertion site analysis (per section 11.2.2) at 3 months, 6 months and 1 year. After 2 years, office visits or phone follow-up at least every 6 months. After 5 years, office visits or phone follow-up at least annually. Collection of blood for immune monitoring analysis, transgenic cell persistence, insertion site analysis and for RCR and RCL testing annually up to 15 years.

^d^G-CSF and plerixafor mobilized leukapheresis will be performed. The number of rounds of leukapheresis depends on the number of CD34+ obtained. CD34+ cells for transduction must be obtained within 3 leukaphereses. Up to 4 leukaphereses total can be performed to obtain a sufficient number of un-manipulated cells for backup and cells for transduction (Table 3).

^e^Additional blood for protein analyses in plasma or serum will only be collected after day 30 if there is a suspicion of cytokine storm or an acute event requiring additional analyses in blood. In addition to collecting PBMC at immune monitoring time points after 30 days, the plasma can be collected as well from the same blood sample.

^f^Blood draws on these days should be performed if considered safe due to the pancytopenia and hemodynamic state of the patient.

^g^Imaging scans can be done at day 25 ± 10 days and day 120 ± 30 days.

^h^Gene modified TCR engineered PBSCs will be administered on Day 0.

^i^Blood for cytokine analysis on Day 0 should be drawn prior to administration of gene-modified cells.

^j^Serum or plasma will be collected at 0, 1/2, 1, 2, 4 and 6 hours after the end of busulfan infusion after the 1^st^ and 5^th^ or 6^th^ dose for calculation of AUC and dose adjustment.

^k^If necessary, Busulfan and Fludarabine administration can be shifted forward 1 day to begin on Day -4.

^k^Detection of CMV, EBV and adenovirus.

In general, changes in the range of ± 10 days from the scheduled event until Day 120 will not be considered deviations from the study timeline. After Day 120,

changes in the range of ± 1 month from the scheduled event will not be considered deviations from the study timeline.

Table 3. Schedule of Events for Stem Cell Mobilization

|  | **mDay 1** | **mDay 2** | **mDay 3** | **mDay 4** | **mDay 5** | **mDay 6** | **mDay 7** | **mDay 8** | **~ 3-4 weeks** | **Day -5** |
| --- | --- | --- | --- | --- | --- | --- | --- | --- | --- | --- |
| G-CSF Administration | X | X | X | X | X | X | X | X |  |  |
| Plerixafor Administration |  |  |  | X | X | X | X |  |  |  |
| Mobilized Leukapheresis^a^ |  |  |  |  | X | X | X | X |  |  |
| Lot Release Testing |  |  |  |  |  |  |  |  | X |  |
| Unmobilized Partial Leukapheresis |  |  |  |  |  |  |  |  |  | X |

^a^ Up to 4 total leukaphereses on mDays 5-8 (depending on how many collections are required) will be performed to collect PBSC for transduction and for backup. PBSC collected for transduction must be obtained within 3 leukaphereses. Leukaphereses may occur up to mDay 8.

# TABLE OF ABBREVIATIONS

ACT: Adoptive cell transfer

ADL: Activities of daily living

AE: Adverse event

ALT: Alanine aminotransferase

ANC: absolute neutrophil count

AO/DAPI: Acridine Orange (AO) and 4’,6-diamidino-2-phenylindole (DAPI)

AUC: Area Under Curve

AST: Aspartate aminotransferase

BSL-2: Biosafety level-2

BUN: Blood urea nitrogen

CBC: Complete blood count

CLL: Chronic lymphocytic leukemia

CDR: Complementarity Determining Region

cGMP: Current Good Manufacturing Practices

CMV: Cytomegalovirus

CR: Complete response

CrCl: Creatinine clearance

CRF: Case report form

CRP: C reactive protein

CT: Computed tomography

CTAs: Cancer-testis antigens

CTCAE: Common Terminology Criteria for Adverse Events

CTLs: Cytotoxic T lymphocytes

DC: Dendritic cells

DLT: Dose limiting toxicity

DMSO: Dimethyl sulfoxide

DNA: Deoxyribonucleic acid

DSMB: Data Safety and Monitoring Board

EBV: Epstein-Barr virus

ECG: Electrocardiogram

ECOG: Eastern Cooperative Oncology Group

ELISA: Enzyme linked immunosorbent assay

FDA: Food and Drug Administration

[^18^F]FDG: [^18^F]fluorodeoxy-glucose

GCP: Good Clinical Practices

GM-CSF: Granulocyte-macrophage colony stimulating factor

GMP: Good Manufacturing Practices

HBSS: Hank’s Balanced Salts Solution

HEPA: High-efficiency particulate air

HEPES: 4-(2-hydroxyethyl)-1-piperazineethanesulfonic acid

HGCTF: Human Gene and Cell Therapy Facility

HIPAA: Health Insurance Portability and Accountability Act

HIV: Human immunodeficiency virus

HLA: Human leukocyte antigen-A*0201

HSA: Human serum albumin

ICF: Informed consent form

IBC: Institutional Biosafety Committee

IHC: Immunohistochemistry

IND: Investigational New Drug

IUVPF: Indiana University Vector Production Facility

i.v.: Intravenous

IRB: Institutional Review Board

ISPRC: Internal Scientific Peer Review Committee

IL: Interleukin

IU: International Units

JCCC: Jonsson Comprehensive Cancer Center

L: Leucine

LD: Longest diameter

LDH: Lactate dehydrogenase

LTR: Long terminal repeat

LVEF: Left ventricular ejection fraction

MCB: Master cell bank

MHC: Major histocompatibility complex

MRI: Magnetic resonance imaging

MSCV: Murine stem cell virus

NCI: National Cancer Institute

NIH: National Institutes of Health

PBMC: Peripheral blood mononuclear cells

PCR: Polymerase chain reaction

PD: Progressive disease

PBSC: Peripheral blood stem cells

PET: Positron emission tomography

PI: Principal investigator

PO: oral administration

PR: Partial response

PRN: pro re nata

PS: Performance status

PTT: Partial Thromboplastin Time

RAC: Recombinant Advisory Committee

RBC: Red blood cells

RCR: Replication competent retrovirus

RECIST: Response Evaluation Criteria in Solid Tumors

RNA: Ribonucleic acid

SAE: Serious adverse event

SC: Subcutaneous

SCID: Severe combined immune deficiency

SD: Stable disease

SOP: Standard operating procedure

SUV: Standardized uptake value

TCR: T cell receptor

TID: Three times a day

TIL: Tumor infiltrating lymphocytes

UCLA: University of California Los Angeles

USP: United States Pharmacopeial Convention

Y: Tyrosine

# STUDY OBJECTIVES

## Primary

### Safety

The safety of adding LV-optNYESOTCR/TK transduced PBSC (LV-NYESO TCR/sr39TK PBSC) to the basic NY-ESO-1 TCR ACT protocol (RV-NYESO TCR PBMC) will be assessed within the current protocol. The NYESO-1 TCR and the retroviral vector MSGV1-A2aB-1G4A-LY3H10 has already been tested in a completed and reported phase 2 clinical trial by investigators at the Surgery Branch/NCI ([Robbins et al., 2011b](#_ENREF_91)). In this trial, no grade 3 or 4 side effects were reported in 17 patients with melanoma and synovial cell sarcoma. In addition, CD4+ cells trained to recognize NY-ESO-1 were used successfully in one patient with metastatic melanoma without any acute or long term toxicities (26 months) ([Hunder et al., 2008](#_ENREF_48)). At UCLA, we have 3 trials open using an NY-ESO-1 TCR ACT protocol under IND #15167 (IRB #12-000153, IRB #13-001624 and IRB #15-001433). Ten subjects have been treated in these trials with RV-NYESO TCR PBMC within an ACT protocol. The majority of the SAEs were due to IL-2, the conditioning regimen (cyclophosphamide and fludarabine) or disease progression. The SAEs due to IL-2 include IL-2- induced eosinophilia, cytokine storm, tachycardia and respiratory distress. The risk of SAEs from IL-2 has been reduced by shortening the duration of IL-2 administration from 28 doses to 14 doses. The conditioning regimen, in this trial, has been changed to busulfan and fludarabine. No SAEs have been attributed to RV-NYESO TCR PBMC to date. Additional safety signals that become apparent in these studies will be incorporated into the current study as well. Although the combination of LV-NYESO TCR/sr39TK PBSC and RV-NYESO TCR PBMC has not yet been tested in humans, our preclinical data does not suggest any obvious safety concerns.

## Secondary

### Feasibility

This protocol attempts to administer two cell-based therapies that require laboratory manipulation within the setting of Good Manufacturing Practices (GMP). Each cell therapy, the RV-NYESO TCR PBMC and the LV-NYESO TCR/sr39TK PBSC, will require strict lot release criteria of the final product before administration. Feasibility will be determined, and if 2 out of 6 patients cannot receive the intended cellular therapies, or if there is suboptimal TCR transgenic cell persistence *in vivo*, further accrual will not be warranted. The feasibility assessment will be based on:

1. Potential problems in the manufacturing of RV-NYESO TCR PBMC or the LV-NYESO TCR/sr39TK PBSC.
2. Potential problems in the delivery of the proposed combinatorial therapy with the addition of engineered stem cells to the basic NY-ESO-1 TCR ACT protocol.

### NY-ESO-1 TCR Transgenic T Cell Persistence.

The persistence of RV-NYESO TCR PBMC and LV-NYESO TCR/sr39TK PBSCcells will be determined by analyzing serial peripheral blood samples and tumor biopsy samples for the presence of T cells with the NY-ESO-1 TCR by dextramer analysis, polymerase chain reaction (PCR) and/or immune monitoring assays.

### Clinical Response

Clinical anti-tumor responses will be determined by RECIST objective response criteria. The rate of CR plus PR will be used to explore antitumor activity. Response assessment will be performed by comparing standard CT imaging scans and photographs of target lesions from baseline with repeated imaging obtained at day 60 and 120 after the TCR transduced PBMC and PBSC adoptive transfer.

## Exploratory

### *In Vivo* Imaging.

We will explore the use of changes in standardized uptake value (SUV) of [^18^F]FHBG PET between post-ACT, day 25 and day 120 scans (dates are approximate and can be modified during the study conduct) to study the biodistribution of LV-NYESO TCR/sr39TK PBSC and progeny T cells.

### Clinical Response per iRECIST.

Immunotherapy and cellular therapy interventions against cancer can lead to unique response patterns, including durable stable disease, delayed response after initial tumor burden increase (flare response), and regression of a target lesion with appearance of new lesions. In order to classify these, we will also classify tumor responses per iRECIST ([Seymour et al., 2017](#_ENREF_103)). In brief, this will reset the classification if RECIST Progressive Disease (PD) is followed at next time point by tumor shrinkage, with new overall response defined as “iUPD” (immune unconfirmed progressive disease). iCPD (immune confirmed progressive disease) would then be confirmed by repeat imaging 4-8 weeks later.

# BACKGROUND

## NY-ESO-1 Antigen as a Target for Immunotherapy

NY-ESO-1 belongs to a family of genes referred to as Cancer-Testis Antigens (CTAs) ([Gogas et al.](#_ENREF_44)). CTAs are generally only expressed embryonically and are not expressed in adult somatic tissues, but can be re-expressed in malignancies ([Gnjatic et al., 2006](#_ENREF_43)). This is precisely the case for NY-ESO-1. Studies have indicated that the NY-ESO-1 protein is only expressed in male spermatogonia, but evidence for its expression in all other adult somatic tissues has not been found ([Bolli et al., 2005](#_ENREF_15); [Jungbluth et al., 2001](#_ENREF_52); [Satie et al., 2002](#_ENREF_98); [Sato et al., 2005](#_ENREF_100)). On the other hand, the NY-ESO-1 protein has been observed to range in expression from 10-40% across a wide range of malignancies, and >80% in synovial sarcoma ([Barrow et al., 2006](#_ENREF_6); [Jungbluth et al., 2001](#_ENREF_52); [Odunsi et al., 2003](#_ENREF_79); [Sato et al., 2005](#_ENREF_100)). Together, these observations indicate that NY-ESO-1 is an ideal target for cellular based immunotherapies. In addition, a lack of major histocompatibility complex class-I (MHC-I) in the testes greatly reduces the potential for TCR based immunotherapeutics to induce orchitis ([Lois et al., 2001](#_ENREF_67)).

## Rationale for Immune-based Therapies for Advanced Malignancies.

Progress in our understanding of how the immune system recognizes and kills cancer cells has led to the notion that high levels of circulating, tumor-antigen-specific T cells may be required for effective antitumor responses. Active immunotherapy with several forms of cancer vaccines has shown that antigen-specific T cells can be activated and occasionally lead to antitumor responses ([Ribas, 2006](#_ENREF_86); [Ribas et al., 2003](#_ENREF_87)). However, the circulating levels of antigen-specific T cells and tumor response rates are much lower than desirable ([Rosenberg et al., 2004](#_ENREF_94)).

Adoptive transfer of clonally-expanded, tumor antigen-specific lymphocytes to lymphopenic hosts after nonmyeloablative conditioning chemotherapy has resulted in cell proliferation and persistent clonal repopulation ([Dudley et al., 2002a](#_ENREF_33)). This approach has resulted in an objective response rate of over 50% in patients with metastatic melanoma, that holds up in larger groups of patients ([Dudley et al., 2005](#_ENREF_35)). The major limitation for the broad clinical applicability of this approach is the requirement for large-scale *ex vivo* lymphocyte culture expansion (up to 10^11^ cells) ([Dudley et al., 2002a](#_ENREF_33)), that restricts this approach to a highly select group of patients.

## Lessons Learned from Targeting Tumor Associated Antigens with Immunotherapy

Multiple lines of evidence suggest that most tumor responses to immunotherapy involve the activation of tumor antigen-specific CD8+ cytotoxic T lymphocytes (CTLs) ([Blattman and Greenberg, 2004](#_ENREF_14)). These immune cells have a high affinity receptor, the TCR that specifically recognizes an 8-10 amino acid peptide of its ligand in a complex within an MHC molecule. A trial conducted at the NCI utilizing ACT directed against the NY-ESO-1 peptide demonstrated a clinical response in 5/11 patients with melanoma (with 2 patients experiencing a complete response (CR) with 20+ and 22+ months of follow-up) and a clinical response in 4/6 patients with synovial sarcoma ([Robbins et al., 2011b](#_ENREF_91)). In a follow up report by NCI, partial responses (PR) were observed in 10/18 synovial sarcoma and 7/20 melanoma patients and complete responses were observed in 1/18 synovial sarcoma and 4/20 melanoma patients ([Robbins et al., 2015](#_ENREF_88)). Furthermore, a multicenter clinical trial in myeloma patients sponsored by Adaptimmune using this same NY-ESO TCR has reported a near CR (nCR, defined as myeloma monoclonal band detectable only by sensitive immunofixation assay) or a CR in 14/20 patients, a very good PR (VGPR; ≥ 90% reduction in paraprotein levels) in 2/20 patients and a PR (50-90% reduction in paraprotein level) in 2/20 patients ([Rapoport et al., 2015](#_ENREF_85)). This provides an impetus for extending this type of therapy to other solid tumors without viable treatment options.

## Why Gene Therapy

The adoptive transfer of large numbers of antigen-specific T cells leads to the highest percentage of tumor regressions reported in patients with melanoma, but this approach is only feasible in a minority of patients where TIL can be cloned and expanded from melanoma metastases ([Dudley et al., 2002a](#_ENREF_33); [Dudley et al., 2005](#_ENREF_35)). Genetic engineering of T cells with optimal TCRs would make this approach more broadly applicable. Currently, there is no other approach that would allow the generation of large numbers of uniformly specific T cells directed against tumor associated antigens in a short (less than one week) *ex vivo* cell manipulation. Therefore, gene transfer techniques are the only approach that would allow testing this concept in human subjects, as supported by preliminary data from a published clinical trial at the Surgery Branch/NCI ([Morgan et al., 2006](#_ENREF_76)).

## TCR Gene Therapy

The transfer of TCR genes is necessary and sufficient to endow recipient T cells with the specificity of donor T cells ([Dembic et al., 1987](#_ENREF_31)). Genetically modified T cells carrying foreign TCRs respond to target antigen recognition through the transgenic TCR both *in vitro* and *in vivo*, leading to effective immune responses to viral and tumor challenges in murine adoptive transfer models ([Schumacher, 2002](#_ENREF_102)). T cells redirected by TCR gene transfer are fully functional after transfer into mice, and have been shown to expand dramatically (over three logs) after encounter with their cognate antigen *in vivo* ([Kessels et al., 2002](#_ENREF_54)). Preliminary data in humans provides evidence that the same is true in patients with metastatic melanoma and synovial sarcoma ([Johnson et al., 2009](#_ENREF_50); [Morgan et al., 2006](#_ENREF_76); [Robbins et al., 2011b](#_ENREF_91)).

## Retroviral Vector-based Human Gene Medicine

Retroviral-mediated gene transfer is an efficient means for the expression of a transgene into actively dividing primary cells. Up to 99% of T lymphocytes can be transduced if cells are non-specifically activated with anti-CD3 and IL-2 ([Johnson et al., 2009](#_ENREF_50); [Morgan et al., 2006](#_ENREF_76)).

## Safety and Regulatory Issues of Retroviral-based Gene Transfer Approaches

Retroviral vectors have a bias towards preferential integration in gene-rich regions, particularly near transcribed genes ([Rohdewohld et al., 1987](#_ENREF_92)). The theoretical concern of insertional mutagenesis had been recognized early with the use of retroviral vectors, since these viruses were known to cause tumors in mice, and their ability to transactivate normally silent genes has been a useful technique in the study of multiple oncogenes ([Suzuki et al., 2002](#_ENREF_107)). However, retroviral vector oncogenesis from insertional mutagenesis has proven to be extremely rare in human subjects.

Over 250 patients in over 40 clinical trials have received stem cells genetically modified with retroviral vectors ([Kohn et al., 2003a](#_ENREF_59)), and the only cases of malignant transformation are the two infants in the severe combined immune deficiency (SCID) study ([Hacein-Bey-Abina et al., 2003](#_ENREF_47); [McCormack and Rabbitts, 2004](#_ENREF_71)). Ten infants with X-linked SCID resulting from a mutation in the *γc* gene (a surface cytokine receptor) were successfully treated with bone marrow transplantation of autologous peripheral blood stem cells (PBSC) genetically modified to express the missing gene using retroviral vectors. Two of the infants developed a T-cell leukemia approximately 3 years later. In both cases, the retrovirus carrying the γc gene had inserted near *LMO2*, a proto-oncogene that is activated in some forms of acute leukemia ([Hacein-Bey-Abina et al., 2003](#_ENREF_47); [McCormack and Rabbitts, 2004](#_ENREF_71)). This event highlights the risks of insertional mutagenesis induced by DNA-integrating viral vectors. However, multiple lines of evidence suggest that this may be an isolated event linked to the possible oncogenic capacity of the γc gene when under the control of a retroviral promoter, with a second hit being the activation of an endogenous oncogene like *LMO2* ([Hacein-Bey-Abina et al., 2003](#_ENREF_47); [Kohn et al., 2003a](#_ENREF_59); [Kohn et al., 2003b](#_ENREF_60); [McCormack and Rabbitts, 2004](#_ENREF_71)). Therefore, particularities of this study (the *γc* gene functioning as a first hit for leukemogenesis, a predilection for vector insertion near the *LMO2* oncogene as second hit and the baseline immune deficiency of the patients not being able to control transformed clones) seem to have played a major role in these two cases ([Berns, 2004](#_ENREF_11)).

## Gene Therapy Utilizing Lentiviral Vectors

Lentiviral vectors were developed in the mid-1990’s from components of the HIV-1 lentivirus. Lentiviral vectors have several attributes that make them potentially safer and more effective than γ- retroviral vectors ([Naldini et al., 1996](#_ENREF_78); [Zufferey et al., 1998](#_ENREF_124); [Zufferey et al., 1997](#_ENREF_125)), specifically their ability to transduce cells that are non-dividing or quiescent, a characteristic of the most primitive and long-lived PBSC. Lentiviral vectors are more effective than γ-retroviral vectors, as indicated in studies using a number of surrogate assays for human PBSC function, including *in vivo* growth in NOD/SCID mice and eLTCIC assays of quiescent CD34+/CD38- cells ([Case et al., 1999](#_ENREF_19); [Miyoshi et al., 1999](#_ENREF_72)). Lentiviral vectors can effectively transduce cells within only 1-2 days of culture, in contrast to the 3-5 days needed for γ-retroviral vectors. This shorter culture duration has been clearly proven to increase the survival of pluripotent stem cells ([Kennedy et al., 2009](#_ENREF_53); [Mazurier et al., 2004](#_ENREF_70)). Transduction by lentiviral vectors was achieved even with a single “hit” on the first day of cell isolation, and did not require repeated cycles of exposure to the vector. Transduction could also be achieved in the absence of recombinant cytokines, although the efficiency was increased 2-fold with use of the cytokines IL-3, IL-6, and SCF. Ideally, use of lentiviral vectors will lead to more effective transduction of quiescent PBSC with preservation of stem cell function. Thus, lentiviral vectors show greatly improved ability to transduce human PBSC *ex vivo*, which may lead to increased numbers of gene-modified cells *in vivo*. More effective gene transfer of pluripotent hematopoietic stem cells may lead to more robust, rapid and long term immune reconstitution than what occurs with γ-retroviral vectors.

## Safety and Regulatory Issues Regarding Lentivirus Gene Transfer Approaches

Lentiviral vectors configured to lack the strong enhancer elements of retroviral long-terminal repeats, present in typical retroviral vectors, have proven to be safer ([Modlich et al., 2006](#_ENREF_73); [Modlich et al., 2008](#_ENREF_74); [Montini et al., 2006](#_ENREF_75); [Zychlinski et al., 2008](#_ENREF_126)). These factors may translate into a lower risk for causing pre-leukemic or leukemic disorders in gene therapy subjects. There have been multiple clinical trials that have successfully exploited lentiviral vectors in stem cell gene therapy for a variety of hereditary disorders. Lentiviral vectors have been utilized for treatment of adrenoleukodystrophy ([Cartier et al., 2009](#_ENREF_18)), β-thalassemia ([Cavazzana-Calvo et al., 2010](#_ENREF_20)), Wiskott-Aldrich syndrome ([Aiuti et al., 2013](#_ENREF_2)) and metachromatic leukodystrophy ([Biffi et al., 2013](#_ENREF_12)). All of these trials used autologous PBSC as the target cell population in association with partial to fully myeloablative conditioning using alkylating agents such as busulfan. Preliminary results indicate that these strategies can achieve effective long term and high level gene expression ([Cartier et al., 2009](#_ENREF_18)).

## NY-ESO-1 TCR-engineered T Cell Experience In Humans

1. NCI trial 08-C-0121 treated 38 patients using the MSGV-1G4-α95:LY retroviral vector generated from the same master cell bank as the one used for the vector in the current study. This study enrolled patients with synovial cell sarcoma and metastatic melanoma. 11/18 synovial sarcoma and 11/20 melanoma patients demonstrated objective clinical responses in this trial (NCT00670748). No toxicities in this trial were attributed to the transferred cells ([Robbins et al., 2011b](#_ENREF_91)). ([Robbins et al., 2015](#_ENREF_88)).
2. A total of 10 patients have received the infusion of NY-ESO-1 TCR transgenic cells to date at UCLA under IND #15167 with or without ipilimumab using the MSGV-1G4- α95:LY retroviral vector generated from the same master cell bank as the one used for the vector in the current study. Tumor types for those patients include synovial sarcoma, melanoma, liposarcoma and malignant peripheral nerve sheath tumor. At day 90, 1 patient had a partial response (PR), 3 patients had stable disease (SD), 6 patients had progressive disease (PD) or did not survive to day 90. The patient who had the partial response had a durable response; a complete response at 9.5 months and remains alive with a complete response at 2 1/2+ years post adoptive cell transfer. Serious adverse events (SAE) have been related to IL-2, ipilimumab and the conditioning chemotherapy. These include febrile neutropenia, pancytopenia, vision loss, IL-2 induced eosinophilia, cytokine storm, tachycardia, respiratory distress and elevated liver enzymes. No toxicities in this trial have been attributed to the transferred cells.
3. In a trial sponsored by Adaptimmune (NCT01352286), 14/20 patients had an nCR or CR and 4/20 had a VGPR or PR. There were no treatment-related fatalities and all serious adverse events were resolved ([Rapoport et al., 2015](#_ENREF_85)).
4. Currently, there are several other accruing clinical trials which employ genetically engineered TCRs against NY-ESO-1 (NCI trials: 01343043, 01350401, 00670748, 00871481, 01697527, 01477021 and 01333046). These trials are being conducted in patients with metastatic melanoma and synovial sarcoma.

# PRELIMINARY DATA

## Origin and Functionality of the NY-ESO-1 TCR

The retroviral vector, MSGV1-A2aB-1G4A-LY3H10, expressing the NY-ESO-1 TCR was obtained from the master cell bank maintained at Indiana University. The particular vector was deposited by Drs. Steven Rosenberg and Paul Robbins from the NCI. The parental 1G4 clone recognizing NY-ESO-1_157-165_ was originally derived from phage display libraries ([Li et al., 2005](#_ENREF_65)). This clone presents a substitution (alpha95:LY) in the CDR3 alpha region that enhances the affinity of the TCR for the NY-ESO-1_157-165_ peptide without losing antigen specificity after being transferred into CD4+ or CD8+ cells ([Robbins et al., 2008](#_ENREF_89)). As such, the modified 1G4 clone with the alpha chain mutation was selected to be used in an ACT trial at the NCI in tumors expressing NY-ESO-1.

## Preliminary Data at the Surgery Branch/NCI

**Using the NY-ESO-1 TCR Retroviral Vector (MSGV1-A2aB-1G4A-LY3H10) in Human Subjects**

Investigators at the Surgery Branch of the NCI reported on 38 patients with NY-ESO-1 positive metastatic melanoma and synovial sarcoma treated with the ACT of lymphocytes expressing the NY-ESO-1 TCR. The Surgery Branch protocol differs with the UCLA protocol in that up to 1.3 x 10^11^ TCR transgenic cells were administered, and that no DC vaccine was included. Patients in the NCI trial were conditioned with cyclophosphamide and fludarabine (25 mg/m^2^/d x 5 days) while the UCLA protocol administers fludarabine at 25 mg/m^2^/day x 4 days. The protocol was feasible and safe, with no reported grade 3 or 4 toxicities ([Robbins et al., 2011b](#_ENREF_91)). Partial responses (PR) were observed in 10/18 synovial sarcoma and 7/20 melanoma patients and complete responses (CR) were observed in 1/18 synovial sarcoma and 4/20 melanoma patients ([Robbins et al., 2015](#_ENREF_88)).

## Preliminary Data at UCLA

**Using the NY-ESO-1 TCR Retroviral Vector (MSGV1-A2aB-1G4A-LY3H10) in Human Subjects**

At UCLA, 10 patients with NY-ESO-1 positive tumors have been treated with the ACT of lymphocytes expressing the NY-ESO-1 TCR. Patients were conditioned with cyclophosphamide (60 mg/kg/day x 2 days) and fludarabine (25 mg/m^2^/day x 4 days). Patients were given IL-2 at 500,000 IU/m^2^ s.c. twice daily for either 7 or 14 days. The duration was reduced from twice daily for 14 days to 7 days following a number of serious adverse events related to the extended IL-2 administration. Objective clinical responses were seen in 1/8 patients to date. At day 90, 1 patient had a partial response, 3 patients had stable disease, 4 patients had progressive disease (PD) or did not survive to day 90 and 2 patients have not yet reached day 90. The patient who had the partial response had a durable response; a complete response at 9.5 months and remains alive with a complete response at 2 1/2+ years post adoptive cell transfer. Serious adverse events observed were related to the longer duration of IL-2 administration, ipilimumab and to the conditioning chemotherapy. These include febrile neutropenia, pancytopenia, vision loss, IL-2 induced eosinophilia, cytokine storm, tachycardia, respiratory distress and elevated liver enzymes. No toxicities have been attributed to the transferred cells.

## Preliminary Data by Adaptimmune

**Using the NY-ESO-1 TCR Lentiviral Vector in Human Subjects**

Adaptimmune has at least 7 studies that are either open or will open soon using NY-ESO-1 TCR in ACT protocols (NCT02588612, NCT01892293, NCT01567891, NCT01350401, NCT01343043, NCT01352286 and NCT01352286). A report of one of these studies has been published indicating that the treatment using T cells transduced with an NY-ESO-1 lentiviral vector was well tolerated without clinically apparent cytokine release syndrome in multiple myeloma patients. IL-6 levels were, however elevated. Objective clinical responses were observed in 16/20 patients ([Rapoport et al., 2015](#_ENREF_85)).

# ELIGIBILITY CRITERIA

## Inclusion Criteria

1. Stage IV or locally advanced unresectable cancers for subjects who have exhausted (or ineligible) all current available treatment options.
2. NY-ESO-1 positive malignancy by immunohistochemistry (IHC) utilizing commercially available NY-ESO-1 antibodies.
3. HLA-A*0201 (HLA-A2.1) positivity by molecular subtyping.
4. Age greater than or equal to 10 years old.
5. A minimum of one measurable lesion defined as:
   1. Meeting the criteria for measurable disease according to RECIST or having at least one or more metastatic lesions that is/are at least double the size of the CT or MRI slice thickness.
   2. Skin lesion(s) selected as non-completely biopsied target lesion(s) that can be accurately measured and recorded by color photography with a ruler to document the size of the target lesion(s).
6. ECOG performance status (PS) 0 or 1.
7. Adequate bone marrow and major organ function to undergo a PBSC transplant determined within 30-60 days prior to enrollment using standard phase 1 criteria for organ function defined as:
   1. Absolute neutrophil count (ANC) ≥ 1.5 x 10^9^cells/L.
   2. Platelets ≥ 100 x 10^9^/L.
   3. Hemoglobin ≥ 9 g/dL.
   4. Aspartate and alanine aminotransferases (AST, ALT) ≤ 2.5 x ULN (≤ 5 x ULN, if documented liver metastases are present).
   5. Total bilirubin ≤ 2 x ULN (except patients with documented Gilbert’s syndrome).
   6. Creatinine < 2 mg/dl (or a glomerular filtration rate > 60).
8. Must be willing and able to accept at least three leukapheresis procedures.
9. Must be willing and able to undergo three research PET scans.
10. Must be willing and able to provide written informed consent.

## Exclusion Criteria

1. Inability to purify ≥2.5 x 10^6^ CD34-enriched cells/kg of patient weight from the pooled G-CSF mobilized leukapheresis products. Note: This information will not be available until after the stem cell mobilization and the subsequent assessments are complete. Therefore, this information will not be available for the initial verification of eligibility.
2. Previously known hypersensitivity to any of the agents used in this study; known sensitivity to busulfan or fludarabine.
3. Received systemic treatment for cancer, including immunotherapy, within 28 days prior to initiation of conditioning chemotherapy administration within this protocol.
4. Potential requirement for systemic corticosteroids or concurrent immunosuppressive drugs based on prior history or received systemic steroids within the last 2 weeks prior to enrollment (inhaled or topical steroids at standard doses are allowed).
5. HIV seropositivity or other congenital or acquired immune deficiency state, which would increase the risk of opportunistic infections and other complications during chemotherapy-induced lymphodepletion. If there is a positive result in the infectious disease testing that was not previously known, the patient will be referred to their primary physician and/or infectious disease specialist.
6. Hepatitis B or C seropositivity with evidence of ongoing liver damage, which would increase the likelihood of hepatic toxicities from the chemotherapy conditioning regimen and supportive treatments. If there is a positive result in the infectious disease testing that was not previously known, the patient will be referred to their primary physician and/or infectious disease specialist.
7. Dementia or significantly altered mental status that would prohibit the understanding or rendering of informed consent and compliance with the requirements of this protocol.
8. Known clinically active brain metastases. Prior evidence of brain metastasis successfully treated with surgery or radiation therapy will not be exclusion for participation as long as they are deemed under control at the time of study enrollment and there are no neurological signs of potential brain metastases.
9. Pregnancy or breast-feeding. Female patients must be surgically sterile, postmenopausal for two years, have not yet achieved menarche, or must agree to use effective contraception during the period of treatment and for 6 months afterwards. All female patients with reproductive potential must have a negative pregnancy test (serum/urine) within 14 days from starting the conditioning chemotherapy. The definition of effective contraception will be based on the judgment of the study investigators.
10. Since IL-2 is administered following cell infusion:
    1. Patients will be excluded if they have a history of clinically significant electrocardiogram (ECG) abnormalities, symptoms of cardiac ischemia with evidence of ischemia on a cardiac stress test (stress thallium, stress MUGA, dobutamine echocardiogram or other stress test).
    2. Similarly, patients with a baseline LVEF < 45% will be excluded.
    3. Patients with ECG results of any conduction delays (PR interval >200 ms, QTC > 480 ms), sinus bradycardia (resting heart rate <50 beats per minute), sinus tachycardia (heart rate >120 beats per minute) will be evaluated by a cardiologist prior to starting the trial. Patients with any arrhythmias, including atrial fibrillation/atrial flutter, excessive ectopy (defined as >20 PVCs per minute), ventricular tachycardia or 3^rd^ degree heart block will be excluded from the study unless cleared by a cardiologist.
    4. Patients with pulmonary function test abnormalities as evidenced by a FEV_1_/FVC < 70% of predicted for normality will be excluded.
11. Bone marrow involvement based on prior CT or PET scans.
12. Active or recent HSV infection or cytomegalovirus (CMV) based on symptoms with positive swab culture and/or positive IgM screening, which would complicate the post-conditioning period.
13. Liver metastases with no other metastatic sites based on prior scans. There is a significant amount of liver uptake with [^18^F]FHBG. If there are only liver metastases, the signal of the PET tracer may be obscured by the high background level in the liver and no signal would be observed in the subject.

## Definition of Study Enrollment Date

Enrollment will be considered the time when the eligibility criteria is being entered into the study eligibility form after the screening tests have been completed to declare if the patient is able or not able to be considered for this research.

# STUDY PROCEDURES

Given the pilot nature of this study, the multiple procedures involved and the use of two cell-based therapies manufactured from patients’ blood samples, the timeline of the study is aimed at defining their approximate administration to patients. In general, changes in the range of ±10 days from the scheduled event will not be considered deviations from the study timeline. If there is a clear trend of procedures that need to be performed at different times in study subjects, the protocol will be amended to attempt to adjust the study timeline to make procedures in the clinical study schedule feasible.

## Baseline Screening Assessments

Patients will be screened to determine whether they meet full eligibility criteria after the informed consent form (ICF) is signed. Standard of care medical history, pertinent laboratory data and significant medical problems of the patient will form the basis for proceeding to evaluate the candidate in more detail. Following signing of the ICF and enrollment into the study, the remainder of screening procedures and tests will be completed. An attempt will be made to complete all tests within 30 days of starting participation in the clinical trial. However, given the multiple screening tests and the pre-study procedures to assess patient eligibility, tests older than 30 days may be acceptable and will limit unnecessary test repetition. Key tests will be repeated at the discretion of the treating physicians if older than 30 days. A pregnancy test must be completed within 14 days prior to dosing, if applicable.

**Screening tests**:

- Informed Consent.
- Office visit, vital signs and physical exam.
- Determination of HLA-A*0201 (HLA-A2.1) positivity by subtyping using at least intermediate resolution techniques (this testing may already be available from prior analysis).
- Determination of NY-ESO-1 positivity in malignant cells by IHC analysis (this can be done in archived tissue, in a new tumor biopsy or may already be available from prior analysis).
- Tumor Assessments (Imaging/Clinical): Documentation of baseline target and non‑target lesions by imaging techniques or by measurement of clinical lesion(s) must be performed. Documentation of skin lesion(s) that can be clearly visualized must be established by color photography, including a ruler to document size.
- Collect blood for:
  - - White blood cells with differential count and ANC, red blood cell (RBC) count, hemoglobin, hematocrit, and platelet count.
    - Blood Chemistries including: Calcium, chloride, total protein, albumin, potassium, random glucose, sodium, blood urea nitrogen (BUN), creatinine, AST (SGOT), ALT (SGPT), alkaline phosphatase, lactic acid dehydrogenase (LDH), total bilirubin, C-reactive protein (CRP), magnesium, phosphorous and uric acid.
    - Coagulation tests: PT/INR, PTT.
    - Infectious disease serologies: Human Immunodeficiency Virus (HIV), cytomegalovirus (CMV), Epstein-Barr virus (EBV), Hepatitis B virus (HBV), Hepatitis C virus (HCV) and Herpes Simplex Virus-1 (HSV-1) and -2 (HSV-2) serotests (unless already tested within the past 1 year).
    - Thyroid Function: Thyroid stimulating hormone (TSH), free T3 and free T4.
    - Autoantibody Panel: Anti-nuclear antibodies, antineutrophil cytoplasmic antibodies, antimicrosomal, antithyroglobulin, anti-liver-kidney microsomal, anti-islet cell antibodies, antibodies to Ro (SSA) or La (SSB), and antiphospholipid antibodies.
    - Cytokine analysis and immune monitoring: Baseline levels.
    - Additional transplant required testing: Rapid plasma reagin (RPR), Sickle cell screen, ABO/RH blood typing, Fibrinogen.

A trial investigator or a clinical study staff member will complete an Eligibility Checklist based on these results. The checklist will be reviewed by the compliance officer from the JCCC Office of Regulatory Compliance. If eligible to proceed, the leukapheresis will be scheduled and the pertinent laboratory staff will be notified of the anticipated dates of cell processing. If conditioning chemotherapy is scheduled greater than 3 months after the completion of the baseline screening assessments, the following screening tests will be repeated to re-evaluate key eligibility criteria prior to administration of conditioning chemotherapy: Hematology, blood chemistries, coagulation tests, thyroid function, auto-antibody panel, transplant required testing, pregnancy test, cardiac function test, pulmonary function tests and baseline PET CT as described above and in 8.2 below. This is to ensure that subjects who have declining functional status from rapid disease progression, active uncontrolled infection or new organ toxicity are not treated.

If patients require bridging therapy, they will need to have completed therapy and have a repeat disease evaluation by PET/CT within 30 days prior initiation of conditioning chemotherapy administration within this protocol.

## Initial Protocol Procedures

- **Baseline [^18^F]FDG PET/CT Scan.** A baseline PET/CT scan of the chest, abdomen and pelvis using [^18^F]FDG as the tracer will allow recording of the baseline measurable metastatic lesions for comparison with post-infusion CT scans to evaluate tumor responses by RECIST. Bone marrow involvement will also be able to be assessed. An attempt will be made for the baseline PET to be performed in conjunction with the baseline tumor assessment by CT scan to minimize radiation exposure. Note: Eligibility may be verified with a prior CT scan. A baseline scan for the study will be performed just prior to conditioning chemotherapy for the purposes of monitoring subsequent disease response to treatment.
- **Tumor Biopsy.** A biopsy of a malignant lesion accessible to outpatient sampling either surgically or by image-guided techniques will be collected whenever feasible. A minimum target of 5 x 5 x 5 mm tumor tissue will be collected if feasible. A new biopsy may not be required if an acceptable biopsy was performed within 60 days prior to screening.
- **Cardiac Function Tests.** An electrocardiogram (ECG) will record potential pre-existing conduction abnormalities for future comparison if needed. An echocardiogram (ECHO) will be performed to determine if the patient is fit to receive low dose IL-2. A LVEF lower than 45% would preclude participation.
- **Pregnancy Test.** All females of childbearing potential must have a negative serum or urine pregnancy test within 14 days from the initial dose of conditioning chemotherapy in order to be eligible for participation. Females who have undergone surgical sterilization or who have been postmenopausal for at least 2 years are not considered to be of childbearing potential.
- **Central Venous Access Catheter.** A central venous access catheter will be placed prior to the first apheresis. This should ideally be a permanent catheter, for example a Permacath or a Port-A-Cath.
- **Pulmonary Function Tests (PFT)**: Tests will be performed in all patients. If FEV_1_/FVC is < 70% of the predicted value, the patient will be excluded from participation.

## G-CSF Mobilized Leukapheresis (Day -180 to -21 including mDay 1 - 8)

### Collection of G-CSF and Plerixafor Mobilized Cells for Transduction and Unmodified PBSC for Cryopreservation.

Between 6 months and 3 weeks prior to infusion of cells, subjects will undergo G-CSF mobilization of CD34+ PBSC. Subjects will receive (or self-administer) 8-10 µg/kg/day metHuG-CSF (Neupogen® or other biosimilar) subcutaneously on “mobilization days” (mDay) 1-4 in the morning at approximately the same time every day. Peripheral blood may be collected for a CD34 count on mDay 4 to assess the mobilization status of the patient prior to the initiation of leukapheresis.To enhance mobilization of the CD34+ cells, plerixafor, a hematopoietic stem cell mobilizer, will be administered (0.24 mg/kg/day) subcutaneously daily beginning on the evening of mDay 4. Plerixafor will be administered in the clinic. Plerixafor will be administered in the evening prior to initiation of leukapheresis in the morning on mDay 5. Daily administration of G-CSF in the morning and plerixafor in the evening with daily leukaphereses will continue up to mDay 8 until a sufficient number of cells for backup and transduction are collected.

At least 2x10^6^ CD34+ un-manipulated cells/kg (without CD34+ cell isolation) must be obtained for transduction and backup. Backup cells will be cryopreserved in liquid nitrogen vapors (≤ - 140°C) in the UCLA Bone Marrow/Stem Cell Transplant Laboratory according to the their SOP for possible future use if there is lack of engraftment for up to 2 years after collection. If the cells are not used within 2 years of collection, the cells will be de-identified and used for research.

Mobilized CD34+ cells will be collected for transduction over a maximum of 3 days up to mDay 8. Since CD34+ cells can only be stored for 24 hours at room temperature or for 48 hours at 4°C prior to transduction, the patient’s participation in the trial will be terminated if a sufficient number of cells are not obtained within the 3 day period. At least ≥2.5 x 10^6^ CD34-enriched cells/kg have to be obtained for transduction with LV-optNYESOTCR/TK lentiviral vector to obtain the required final number of transduced cells.

If a sufficient number of cells are obtained, CD34+ PBSC will be isolated using the Miltenyi CliniMACS system, transduced and cryopreserved until ready for use after lot release testing (see 10.0).

After CliniMACS purification, if a sufficient number of CD34-enriched cells are not obtained, the patient’s participation in the trial will be terminated as the CD34+ cells can only be stored at room temperature for 24 hours prior to transduction.

Patients who are withdrawn from the study prior to administration of transduced CD34+ cells will resume normal clinical care and may be replaced with an additional subject. These patients are considered “screen failures” since they do not meet the criteria to be able to purify ≥2.5 x 10^6^ CD34-enriched cells/kg. Cells from patients who are screen failures will be stored for 1 year and if not used, the cells will be de-identified and used for research. Efficacy and safety assessments will not be carried out from the point of withdrawal, and data will not be recorded in the Case Report Forms (CRFs).

If the transduced PBSC meet the lot release criteria (~ 3-4 weeks), patients will then proceed to undergo an unmobilized partial leukapheresis to collect PBMC.

### Confirm Vector Copy Number and Transduction Efficiency Prior to Initiating Unmobilized Partial Leukapheresis

Prior to initiating the unmobilized partial leukapheresis to obtain PBMC for transduction, transduced CD34+ PBSC will undergo lot release testing that includes assessment of vector copy number (VCN) and transduction efficiency (see 10.0). These assays require 3-4 weeks.

If the transduction efficiency is greater than 50%, then the cells will either be diluted with unmodified cryopreserved PBSC (if available) or the subject will be mobilized again to obtain additional PBSC with which to dilute the transduced cells. Transduction efficiency greater than 50% is a rare event. This has not occurred in any of the 5 manufacturing validation runs to date.

### Supportive Therapy during G-CSF and Plerixafor Mobilization

- Tylenol and Claritin on the first day in case of bone pain (not aspirin or NSAIDS).
- Epi (epinephrine) kit in case of a possible allergic reaction which is a very rare occurrence.

## Unmobilized Partial Leukapheresis, Cell Processing and Admission to the Hospital

a. Day -5 Morning: Leukapheresis, Cell Processing. An unmobilized partial leukapheresis will be performed followed by cell processing for the manufacture of RV-NYESO TCR PBMC.

b. Day -5 Afternoon: Admission to the Hospital. Subject will be admitted to the Hematology/Oncology unit at the Ronald Reagan UCLA Medical Center.

- Subject will be asked about illnesses, injuries, side effects, and any medications subject has taken or medical procedures that have been done to subject since the prior visit.
- A full history will be taken and a physical exam will be performed on admission. A permanent catheter will have already been placed through which the subject will receive liquids in preparation for the chemotherapy regimen.
- Subject will have blood drawn including complete blood counts and blood chemistries. Most of these blood tests will be repeated daily or every other day during the subject’s stay in the hospital. Magnesium, phosphorus and uric acid will be assessed only upon admission and then will be monitored if clinically indicated.
- Subject will start to receive one or two drugs to prevent complications with infections, the antibiotic Bactrim to decrease the risk of *Pneumocystis carinii* pneumonia, and the drug Ganciclovir to decrease the risk of CMV and HSV-1. Ganciclovir will only be given to subjects who have evidence of prior exposure to the CMV or HSV virus. Both of these drugs will be stopped before administering the gene-modified NY-ESO-1 TCR cells, since both drugs would interfere with their function.
- Prior to receiving busulfan, subjects will be premedicated with anticonvulsants (e.g., benzodiazepines, phenytoin, valproic acid or levetiracetam) to prevent seizures. Anticonvulsants will be administered 12 hours prior to infusion of busulfan. Antiemetics will be administered prior to the infusion of busulfan.

## Days –5 to –2 Conditioning Chemotherapy Regimen

The conditioning chemotherapy will be initiated in the evening following the leukapheresis.

### Supportive Therapy during Chemotherapy Administration:

This therapy will be started on Day –5, counting day 0 as the day of TCR engineered PBSC infusion. The i.v. infusions, procedures and medications will be modified as indicated based on the attending physician and clinical team taking care of patients.

- Hydrate: Begin hydration with 0.9% Sodium Chloride Injection containing 10 meq/L of potassium chloride at 150 ml/hr (or 125ml/m^2^/hr for pediatric patients if this amount is <150ml/hr), starting approximately 4 hours (± 2 hours) pre-busulfan and continuing hydration until 24 hours after last chemotherapy infusion.
- Daily complete blood count (CBC), and comprehensive metabolic panel (CMP) as per standard procedures in the J-Medicine ward.
- Ondansetron (Zofran) 8-16 mg (or 0.15mg/kg/dose up to q6hrs for pediatric patients if <8mg/dose) i.v. on the days of chemotherapy (or similar antagonist of serotonin type 3 [5-HT3] receptors as per standard practices), lorazepam (Ativan) 2 mg i.v. q6h prn (or 0.05mg/kg/dose for pediatric patients if < 2mg/dose) and prochlorperazine (Compazine) 10 mg. i.v. q6h prn for patients >50 kg on the days of chemotherapy conditioning then po afterwards. Patients weighing < 50 kg may also have diphenhydramine (1-2mg/kg/dose every 6-8 hours, max dose 50mg) and metoclopramide 0.1-0.2mg/kg/dose every 6-8 hours, max dose 10mg) for breakthrough nausea/vomiting.
- Trimethoprim-Sulfamethoxazole (Bactrim) 1 tablet administered orally tid from Days –5 to –2, and restarted once the ANC is above 1 x 10^3^/μL until Day +100.
- Ganciclovir (Cytovene) 6 mg/kg on Days –5 to –2 and then discontinued. Ganciclovir will only be given to patients that are CMV or HSV seropositive at baseline or following standard practices.
- Posaconazole 200 mg po tid (or similar prophylactic antifungal based on standard practice) starting on the day of the first genetically modified cell reinfusion and continued until the ANC is above 1.5 x 10^3^/μL consistently or day +100, whichever comes first.
- Furosemide dosed as clinically indicated during the hydration period.
- Premedicate patients with anticonvulsants (e.g., benzodiazepines, phenytoin, valproic acid or levetiracetam) to prevent seizures reported with the use of high dose busulfan. Administer anticonvulsants 12 hours prior to busulfan to 24 hours after the last dose.

Dexamethasone 10 mg (or 0.6 mg/m^2^/dose for pediatric patients if <10 mg) may be added as an adjunct to other anti-nausea medications, but it should be discontinued before Day –1 to avoid its immune suppressive effect against RV-NYESO TCR PBMC,LV-NYESO TCR/sr39TK PBSC adoptive transfer, and low dose IL-2. Steroids should not be administered to patients post adoptive transfer unless discussed with the study principal investigator (PI).

Other medications, or changes in this medication plan, may be administered based on the decisions of the inpatient treating physician’s team.

### Chemotherapy Conditioning (Days -5 to -2):

**Busulfan (Days -5 to -2):**

Busulfan ([Russell et al., 2002](#_ENREF_96); [Ryu et al., 2007](#_ENREF_97); [Sato et al., 2015](#_ENREF_99); [Xhaard et al., 2014](#_ENREF_116)) will be administered i.v. starting with a dosage of 2 mg/kg per dose in two 12 hourly doses for 3 days (= total of 6 doses). The conditioning will be the same for all patients but may be adapted in case of uncontrolled infection. However, this scenario may be modified on a case-by-case basis per the treating physician and should be an exception.

The total AUC that can be reached in 3 days is 70,000-80,000 ng/ml x hr (16,666-19,048 μM/min), which is nearly ablative (99 % of cytoxicity for CD34+ cells). The targeted AUC after a single dose would be 12,000 – 13,000 ng/ml x hr (2,857-3,095 μM/min).

The AUC will be calculated after the first dose, and the dose will be adapted to reach the target net AUC of 70,000-80,000 ng/ml x hr. The AUC will be re-measured after the fifth or sixth dose to estimate total exposure. Optimizing the conditioning regimen will be an important factor for successfully achieving long-term engraftment of the gene-modified cells.

The first dose of busulfan will be administered at 10PM on Day -5 to allow collection of blood after the first dose through the night for assessment of busulfan levels in the morning.

Busulfan levels will be measured in serum or plasma by LC, GC, or MS methods. Blood samples must be centrifuged immediately and frozen at -20°C. Optimal time points for sample collection and calculation of AUC are a) immediately at the end of the infusion (time zero, for calculation after the 5^th^ or 6^th^ dose, a sample must be drawn prior to administration of the that dose) and b) 4, 5 and 6 hours after the end of busulfan infusion. The equivalent of the AUC in mg/kg unit will be also provided to the investigators.

A designated UCLA Pharmacist will calculate the AUCs and prescribe the necessary dose changes for all gene therapy patients in real time, if necessary. The busulfan levels will be measured and results transmitted to the designated UCLA Pharmacist for calculation of AUC and dose adjustment**.**

A wash-out period of at least 24 hours after completion of the 6^th^ busulfan dose infusion will be maintained prior to reinfusion of the transduced cells.

Assess liver function enzymes until Day 28 post-busulfan to monitor for sinusoidal obstruction syndrome following the J-service protocol for patient monitoring on busulfan. Monitor signs and symptoms of cardiac tamponade.

**Fludarabine (Day -5 to -2):**

Fludarabine 40 mg/m^2^/day i.v. daily over 30 minutes for 4 days. Maximum dose calculated based on actual body weight should not exceed 140% of doses calculated based on ideal body weight.

### Prophylactic Supportive Care Throughout the Pancytopenic Period.

In order to closely monitor patient care and decrease study-related toxicities, patients will receive prophylactic therapy throughout the pancytopenic period. The intravenous infusions, procedures and medications will be modified as indicated based on the attending physician and clinical team taking care of patients.

- During the preparative and pancytopenic period, patients will have a daily complete blood count (CBC) and comprehensive metabolic panel (CMP) including a hepatic panel.
- G-CSF 300 or 480 μg will be used following the standard J-Medicine protocol and clinical judgment starting on day +2. Pediatric patients will receive G-CSF 5 μg/kg daily also starting on day +2. This will continue until ANC >1000 for three consecutive days.
- Imipenem at 500 mg i.v. q6h. in case of fever > 38.2 ^o^C (other antibiotic in case of history of allergy to penicillin and derivates; exact antibiotic combination should be decided by the treatment team).
- Packed RBC transfusion to maintain a Hb > 8.0 g/dL, following the standard J-Medicine protocol and clinical judgment.
- Platelet transfusion to attempt to maintain a platelet count of > 10 x 10^3^/μL, (> 20 x 10^3^/μL if there is concomitant fever, or > 50 x 10^3^/μL if there is evidence of bleeding) following the standard J-Medicine protocol and clinical judgment.
- Norethindrone (Norlutate) 10mg po daily will be used for menstruating women as prophylaxis against menorrhagia.

## Day 0-1: LV-NYESO TCR/sr39TK PBSC and RV-NYESO TCR PBMC Infusion

### Premedication

Side effects following T cell and PBSC infusion include transient fever, chills, and/or nausea. For this reason, patients will receive pre-medication with:

- Approximately one hour prior to the infusion of each genetically modified cell therapy preparation, an i.v. infusion of Dextrose in 5%-Normal Saline (D5NS) at 250 ml/hr will begin and continue for 3 hours.
- Briefly (approximately 10 minutes) prior to the infusion, acetaminophen 650 mg po, diphenhydramine 25 mg i.v., and mannitol 12 g i.v. will be administered. Pediatric patients will receive 15mg/kg acetaminophen (max dose 650 mg), diphenhydramine 0.5mg/kg (max dose 25mg), and mannitol 0.25g/kg (max dose 12mg). These medications may be repeated every six hours as needed.
- A course of non-steroidal anti-inflammatory medication may be prescribed if the patient continues to have fever not relieved by acetaminophen.
- It is recommended that patients not receive systemic corticosteroids such as hydrocortisone, prednisone, prednisolone, or dexamethasone, at any time, except in the case of a life-threatening emergency, since this may have an adverse effect on T cell and PBSC survival. If corticosteroids are required for an acute infusion reaction, an initial dose of hydrocortisone 100 mg or dexamethasone 10 mg is recommended. Such subjects will not receive further immune modulating treatments within this protocol, including continued infusion of TCR transgenic cells or IL-2.

### Day 0: LV-NYESO TCR/sr39TK PBSC Infusion

Before LV-NYESO TCR/sr39TK PBSC infusion patients will receive pre-medication as indicated in section 8.6.1. The TCR genetically engineered PBSC will be administered on Day 0 to ensure that there is sufficient time to recover from the conditioning regimen. The product will be transported frozen to the in-patient unit in a dry cryo-container and labeled with a biohazard label. Cells will be thawed at the bedside and infused immediately. Patients will receive a single i.v. infusion of LV-NYESO TCR/sr39TK at a dose of 2.0 - 6.0 x 10^6^ transduced CD34+ PBSC/kg at a rate of ~ 10 ml/min through standard i.v. tubing without a filter. If the transduction efficiency is greater than 50%, then the number of transduced cells will be diluted to less than 50% with unmodified PBSC or the cells will not be administered. If the VCN is <0.1, then the cells will not be administered.

Following cell administration, the bag will be disposed of in the biohazard waste in the hospital.

### Day 1: RV-NYESO TCR PBMC Infusion

Before RV-NYESO TCR PBMC infusion patients will receive pre-medication as indicated in section 8.6.1. The TCR genetically engineered PBMC product will be transported to the in-patient unit immediately after lot release criteria have been verified on ice in a rigid-walled container with a tight fitting lid (i.e. Tupperware; Nalgene container etc.) and labeled with a biohazard label. A target minimum of 10^8^ and up to 10^9^ transduced PBMC will be infused at a rate of ~10 ml/min through standard i.v. tubing without a filter.

Following cell administration, the bag will be disposed of in the biohazard waste in the hospital.

### Patient Monitoring After LV-NYESO TCR/sr39TK PBSC and RV-NYESO TCR PBMC Infusion

- Patients will receive the TCR genetically modified PBMC and PBSC in an individual use room equipped with a high-efficiency particulate air (HEPA) filters that meets the precautions normally used for patients undergoing PBSC transplantation.
- Patients will be in a monitored bed during cell infusion, with routine vital signs recorded every 4 hours. Patients will be on constant cardiac monitoring on Days 0 and 1 during and after the PBSC and PBMC infusions. The vital checks are resumed to the routine floor schedule every 4 hours starting on Day 2.
- Sterile saline, acetaminophen and emergency drugs, including epinephrine [0.3-0.5 mg (0.3-0.5 ml of a 1:1000 solution), diphenhydramine (25-50 mg i.v.), and methylprednisolone (30-60 mg i.v.), should be readily available in the amount and formulation as per the standard treatment of a potential hypersensitivity/anaphylactic reaction.
- If there is an adverse event (AE) characterized by life-threatening hypoxia or hypotension, or any other infusion reaction qualifying as a DLT as described in [section 11.1.1.1](#_Definition_of_Dose), then further infusion of the TCR transgenic cells will be cancelled and no other immune stimulating approaches, such as IL-2, will be administered under this protocol.

**Febrile Reaction**

In the event that a subject develops sepsis or systemic bacteremia following PBMC or PBSC infusion, appropriate cultures and medical management will be initiated. If a contaminated PBMC or PBSC product is suspected, the product will be retested for sterility using archived samples that are stored in the UCLA HGCTF. In this case, the patient will be considered to have a DLT and will not receive more doses of IL-2. No other immune stimulating approaches, such as IL-2, will be administered to the patient under this protocol.

**Viral Prophylaxis**

For viral (CMV, EBV and adenovirus) prophylaxis post-transplant, intravenous immunoglobulin (IVIG) will be administered every 2 weeks beginning on Day +2 (per standard J-med procedures at the physicians discretion) and continuing beyond white blood cell engraftment through day +100. While this is not standard of care in hematopoietic stem cell transplant patients, as it does not improve survival ([Raanani et al., 2009](#_ENREF_82)), it has been shown to be effective at reducing the incidence of CMV infection/reactivation in pediatric patients following allogeneic stem cell transplant ([Goldstein et al., 2017](#_ENREF_45)). Furthermore, we will monitor CMV, EBV and adenovirus viral copy number twice weekly in the patient’s blood through white blood cell engraftment, and beyond engraftment weekly through day +100. In the event of a documented CMV infection, we can give alternative medications to ganciclovir, including foscarnet or cidofovir, both of which have activity against CMV.

## Low Dose IL-2 and Blood Draws for Immune Response Assessments

**Days 2-8: Low Dose IL-2 Administration**

Patients will receive IL-2 (Prometheus) at 500,000 IU/m^2^ IV twice daily for 7 days. Doses may be skipped depending on patient tolerance. Doses will be skipped if patients reach grade III or IV toxicity due to IL-2 except for the reversible grade III toxicities common to IL-2 such as diarrhea, nausea, vomiting, medically manageable hypotension, skin changes, anorexia, mucositis, dysphagia, or constitutional symptoms and laboratory changes. Patients receiving IL-2 will have a CBC and comprehensive metabolic panel evaluated per standard procedures in the stem cell transplantation ward, which is usually daily ([Bauer et al., 1995](#_ENREF_8); [Mackensen et al., 2006](#_ENREF_69); [Roper et al., 1992](#_ENREF_93); [Yee et al., 2002](#_ENREF_123)).

IL-2 administration will be held per the parameters below:

A) Hypotension <80 systolic or hypertension >160 systolic (or 95^th^ percentile for age and height, whichever is lower)

B) Sinus tachycardia > 120, sinus bradycardia <60, or any other arrythmias

C) Syncope

D) Chest pain

E) Seizures

F) Respirations of <10 or >30, or O2 Sat < 90% Room Air

G) Urine output of less than 2mL/kg/hr q4hr or 100 mL q4hr, whichever is lower

H) Change in mental status

I) Temperature more than 103°F/39.4°C, unresponsive to medication

**Fevers During IL-2 Administration**

Fevers are a known side effect of IL-2 administration. Since patients at the time of IL-2 dosing will also be recovering from the conditioning chemotherapy and will likely be pancytopenic, the supportive care during this period includes empiric treatment for infections and IL-2-based toxicities without resulting in a definition of DLT.

Supportive Care During Low Dose IL-2 Administration

Concomitant medications to control side effects of therapy will be given following the standard UCLA practices for high or low dose IL-2-based therapy. Meperidine (25-50 mg) will be given intravenously if severe chills develop. Other supportive therapy will be given as required and may include acetaminophen (0.15mg/kg, max dose 650 mg q6h), indomethacin (50-75 mg q6h) and famotidine (20 mg q12h). If patients require high doses of corticosteroid therapy they will be not receive further immune activating therapies under this protocol. Ondansetron 0.15 mg/kg/dose i.v. every 8 hours (or an equivalent antiemetic) may be administered for nausea or vomiting. Additional antiemetics will be administered as needed for nausea or vomiting. Antibiotic coverage for central venous catheters may be provided at the discretion of the treating physicians.

**Blood Collection for Immune Monitoring Assays**

Collection of 60-100 ml of peripheral blood is scheduled at screening and on study days 30, 45, 60, 75, 90 and 120 and at each long term follow up visit (every two to three months) as described in [section 8.15](#_Elimination_of_Transduced). Blood/cells on Day 120 can be obtained through leukapheresis. Blood draws on these days should be performed if considered safe, and may be skipped or delayed because patients are likely to have chemotherapy-induced pancytopenia and may be hemodynamically unstable after IL-2. These blood draws can be done ± 10 days from the intended days.

**Blood Collection for Plasma Cytokine Analyses**

At least 5 ml of blood in EDTA will be collected for plasma on days 0, 1, 3, 5, 7, 14 and 30 after ACT for cryopreservation/archiving. Blood for protein analysis will only be collected after day 30 if there is suspicion of a cytokine storm or an acute event occurs that warrants further collection of samples for cytokine/chemokine analyses.

## Hospital Discharge

Patients will be discharged from the hospital when the following criteria are met and following the clinical judgment of the treating physicians:

- ANC > 500/μL.
- Platelet count > 20,000/μL.
- Hemodynamically stable.
- Creatinine on downward trend after IL-2.
- Liver function tests stable.
- Not requiring daily blood product infusion.

## Day 25: NY-ESO-1 TCR Cell Tracking After Infusion

On Day 25 post adoptive cell transfer (± 10 days or when the treating physicians deem that the patient is stable enough to undergo these procedures), either as an inpatient or outpatient, the following will be performed to attempt to detect the RV-NYESO TCR PBMC and the PBMC progeny of the LV-NYESO TCR/sr39TK PBSC:

- [^18^F]FHBG PET/CT scan. PET scan using [^18^F]FHBG as a tracer will be performed to explore the use of molecular imaging to assess the biodistribution of the transgenic progeny cells. The CT scan will be attenuated.

## Days 30, 45, 60, 75, 90 and 120: Follow Up Study Visits

On study days 30, 45, 60, 75, 90 and 120 patients will be seen as outpatients in the oncology clinic for an office visit, vital sign determination, safety blood tests and collection of blood for immune monitoring analysis. Day 45 and 75 visits for patients who live far from campus can take place at patients’ primary physician’s clinic, where they will check vital signs and CBC, CMP and LDH values. The results will be send to UCLA study team in order to keep record of them. Also, we will request the primary physician’s clinic to send a lavender top tube with ~20cc of patient’s blood for analytics.

## Permissible Systemic Therapies During the Protocol On-study Period

The on-study period is defined as 120 days from the date of the TCR engineered ACT infusion unless there is overt tumor progression or DLTs leading to patient discontinuation. Systemic immune suppressive therapies will be avoided during this time, but they may be permissible in the following situations:

- Systemic corticosteroids: Used in case there is a suspicion or evidence of a DLT (as described in [section 11.1.1.1](#_Definition_of_Dose)) that may be related to autoimmune or inflammatory reactions, or cytokine storm. In this case, administration of IL-2 will not be continued.

## Day 60: Re-staging Evaluation

- Patients will be evaluated for antitumor activity after undergoing re-staging exams (RECIST criteria). A restaging CT of the chest, abdomen and pelvis will be performed. This is a standard of care timing of restaging scans in patients with metastatic cancers receiving oncologic treatments. Tumor biopsy will be performed whenever feasible.

## Day 120: NY-ESO-1 TCR Cell Tracking After Infusion

- [^18^F]FHBG PET/CT scan. PET scan of the chest, abdomen and pelvis using [^18^F]FHBG as a tracer will be performed to explore the use of molecular imaging to assess the biodistribution of the transgenic progeny cells. The CT scan will be attenuated.
- Blood will be collected for laboratory tests.
- Leukapheresis and plasmapheresis: This will be a partial (5 liter) leukapheresis for PBMC collection with the collection of 100 ml of plasma.

All patients will be followed thereafter. Re-staging exams will be performed every 2-3 months.

## Long-term Follow-up Plan

Regardless if followed on-study or off-study, patients will be asked to comply with the following long-term plan:

- For the first 2 years, office visits at least every 3 months.
- After 2 years, office visits or phone follow-up at least every 6 months.
- After 5 years, office visits or phone follow-up at least annually for a minimum of 15 years.
- Collection of 60-100 ml of blood for immune monitoring analysis and for replication competent retrovirus (RCR) testing at 3 and 6 months, and at one year post cell administration and then annually for 15 years (as described in [section 11.2.2](#_Transgenic_Cell_Persistence.)).

## Elimination of Transduced PBSC by Activation of Suicide Gene

In the unlikely event that the NY-ESO-1 transduced PBSC require elimination due to events such as evidence of a secondary malignancy, RCR or RCL, ganciclovir (Cytovene) 10 mg/kg/day will be administered for 14 days (or shorter if all signs and symptoms have regressed)([Ciceri et al., 2007](#_ENREF_26)). If ganciclovir is used, long term hematopoiesis should not be affected. Not all cells will be transduced (<50%) and carry the suicide gene. Therefore, a sufficient number of stem cells should remain if ganciclovir is administered. In the highly unlikely event that pancytopenia develops following administration of ganciclovir, a bone marrow biopsy will be performed.  If absence of precursor cells is observed, then the back up cells will be administered.

## Bone Marrow Biopsy and Administration of Unmodified Back Up Cells in the Event of Marrow Recovery Failure

If criteria for non-engraftment are met (persistent ANC < 500/μL or platelets < 50,000/µL) at Day +28, a bone marrow biopsy will be performed to confirm engraftment failure. Once this is confirmed, the previously cryopreserved, unmodified CD34+ cells will be thawed and re-infused according to the clinical laboratory standard operating procedure. The banked product will contain a minimum of 2x10^6^ cells/kg body weight, which is sufficient to regain normal hematopoiesis. In the very rare and unanticipated event that the banked unmodified cells fail to engraft, an emergent allogeneic hematopoietic stem cell transplant would be pursued.

# EXPERIMENTAL STUDY AGENTS

If a biosimilar or generic drug exists, we will use whatever the UCLA formulary pharmacy uses.

## PBMC Expressing NY-ESO-1 TCR (RV-NYESO TCR PBMC)

**Overview.** A leukapheresis product obtained from study patients will be transferred to the HGCTF, on the 14^th^ floor of the Factor Building. PBMC will be isolated by gradient centrifugation, and an aliquot of these cells will be cultured for 48 hours in the presence of anti-CD3 (OKT3) and IL-2 in order to stimulate T-cell growth to prepare for viral vector transduction. PBMC will be transduced with a clinical grade retrovirus vector expressing NY-ESO-1 TCR (MSGV1-A2aB-1G4A-LY3H10) using retronectin-coated plates or cell culture bags over two consecutive days and kept in culture for 96 hours from the time of the first retroviral transduction. Transduced cells will be harvested and infused fresh as soon as the lot release criteria are cleared. On the infusion day, patients will have completed a reduced intensity preparative regimen consisting of busulfan and fludarabine.

In the event that the patient is not clinically fit to receive the gene-modified RV-NYESO TCR PBMC infusion within 24 hours of the scheduled day of cell harvest, the cells will be cryopreserved on the harvest day for research use.

### Retrovirus Vector MSGV1-A2aB-1G4A-LY3H10

**Plasmid map MSGV1-A2aB-1G4A-LY3H10.**

**Product Description.** This vector is a splicing-optimized murine stem cell virus (MSCV)-derivate gammaretroviral vector. The expression of the TCR is driven by the MSCV promoter embedded in the 5’LTR and regulated by the splicing donor and acceptor in the vector backbone. The vector also contains the packaging signal that directs the incorporation of the vector RNA into the virions regulates the virus encapsidation. In this TCR vector, the alpha and beta chains of the NY-ESO-1 TCR are separated by a picornavirus P2A “self-cleaving” sequence. The particular vector that is being used in this trial contains a modified version of the non-codon optimized parental 1G4 vector in which the amino acids 95 and 96 of the CDR3 of the alpha chain have been modified to L and Y ([Robbins et al., 2008](#_ENREF_89); [Wargo et al., 2009](#_ENREF_113)).

**Classification:** Immunotherapeutic.

**Active Ingredient.** This retroviral vector has two active transgenes, the alpha and beta chains of a high affinity TCR that jointly recognizes the NY-ESO-1_157-165_ epitope in the context of HLA-A*0201.

- **NY-ESO-1 TCR:** The 1G4 high affinity TCR pair specific for NY-ESO-1_157-165_ presented by HLA-A*0201 was originally cloned from a patient with metastatic melanoma ([Jager et al., 1998](#_ENREF_49)). The TCR was engineered for higher specificity for NY-ESO-1 by modifying the CDR of the alpha chain of the TCR, leading to the current version of the 1G4 TCR used in this study ([Robbins et al., 2008](#_ENREF_89)).

**Mode of Action:** One functional complex will be generated by the active transgenes:

- **NY-ESO-1 TCR:** The NY-ESO-1 MSGV1-A2aB-1G4A-LY3H10 TCR is expected to redirect the specificity of the TCR transgenic T cells to the NY-ESO-1_157-165_ peptide presented by HLA-A2*0201 on the surface of malignant cells.

**Manufacturing Information for the Clinical Grade Vector:** The clinical grade MSGV1-A2aB-1G4A-LY3H10 TCR was generated at the IUVPF. The retroviral vector supernatant RV-NY-ESO-1-TCR was prepared starting from the pG13 master cell bank created for the clinical grade production of the same vector for clinical trials at the Surgery Branch/NCI. The retrovirus supernatant was preserved following current Good Manufacturing Practices (cGMP) conditions at IUVPF. The lot release test results are listed in [Table 4](#Table_4_Clinical_Grade_Retrovirus). The supernatant was stored upon the completion of production at least at –70^o^ C at IUVPF. Upon shipment on dry ice, the supernatant is stored at –80^o^ C at the HGCTF. This facility is equipped with around-the-clock temperature monitoring. Supernatant is thawed and used for *ex vivo* transduction of activated PBMC. There will be no re-use of the same unit of supernatant for different patients. The retroviral titer has been shown to be stable after immediate thawing and immediate administration (tissue culture wells previously coated with retronectin). Handling of the vector should follow the guidelines of Biosafety Level-2 (BSL-2).

**How Supplied:** The clinical grade vector is supplied by IUVPF following their Standard Operating Procedures (SOP) for retroviral vector suspension, labeling and cryopreservation.

**Storage:** Single use aliquots are stored in a viral bank established in a dedicated –80^o^C freezer with central, computerized monitoring system, with alarm and recording of all GMP storage systems located at the restricted access GMP Suite, 14^th^ floor of the Factor Building at UCLA.

**Stability:** Under the conditions of continuous storage at –80^o^C without freeze-thawing, it is expected that the retroviral vector is stable for at least 5 years. Aliquots of this vector will be recertified annually throughout the study period with selected assays for purity, identity and potency.

**Dose and Schedule:** For *ex vivo* use only.

**Route of Administration.** All use of this retrovirus vector is *ex vivo*, and no direct injection will be done in human subjects.

**Prior Human Experience.** This same retrovirus vector expressing the MSGV1-A2aB-1G4A-LY3H10 TCR and similarly used to transduce human PBMC has already been administered to humans within a phase 2 clinical trial at the Surgery Branch/NCI ([Robbins et al., 2011b](#_ENREF_91)) and at UCLA ([Singh AS, 2015](#_ENREF_106)). The prior human experience is as follows:

- **Retrovirus Vectors:** Retroviral vectors have undergone extensive testing in humans. Their main safety caveat is the development of insertional mutagenesis, which seems to be a rare event and may be related to the expressed transgene and the immune competency of the host. Over 250 patients in over 40 clinical trials have received stem cells genetically modified with retroviral vectors ([Kohn et al., 2003a](#_ENREF_59)) and the only cases of malignant transformation are the two infants in a clinical trial inserting the gammaC gene (a surface cytokine receptor) to PBSC of infants with X-linked SCID ([Hacein-Bey-Abina et al., 2003](#_ENREF_47); [McCormack and Rabbitts, 2004](#_ENREF_71)).
- **NY-ESO-1 TCR:** The same NY-ESO-1 TCR alpha and beta gene pair was tested at the Surgery Branch/NCI for its ability to redirect antigen specificity of human PBMC adoptively transferred to lymphopenic hosts in a protocol design similar to the one proposed herein using only transduced PBMC ([Robbins et al., 2011b](#_ENREF_91)). In addition, we have two clinical trials ongoing with the same NY-ESO-1 TCR transduced PBMC (NCT01697527 and NCT02070406) ([Singh AS, 2015](#_ENREF_106)). If we gain knowledge of the development of serious adverse events related to the NY-ESO-1 TCR transduced T cell administration, our protocol procedures and informed consent form will be modified accordingly. Moreover, there are have been at least 11 NCI registered trials that have accrued or are currently accruing patients that are evaluating the safety and efficacy of T cells trained to eliminate NY-ESO-1 positive malignancies. Safety concerns that arise from these trials will also be incorporated into this trial, if deemed appropriate by the study investigators.

**Reported Adverse Events and Potential Risks:** The potential adverse events are related to the viral vector, transgenes and genetically modified cells, as discussed in the following section.

### RV-NYESO TCR PBMC

**Product Description.** PBMC obtained from leukapheresis and activated for 48 hours with OKT3 (anti-CD3 antibody) and IL-2 will be infected with the clinical grade MSGV1-A2aB-1G4A-LY3H10 retroviral vector supernatant over two consecutive days, maintained in culture for 4 days from the start of transduction in IL-2 and then infused fresh into trial subjects. Aliquots of these cells will be used to fulfill the lot release criteria.

**Classification:** Immunotherapeutic.

**Active Ingredient.** As described for the retrovirus vector MSGV1-A2aB-1G4A-LY3H10.

**Mode of Action:** The genetically modified PBMC expressing surface NY-ESO-1 TCR will be expected to be redirected to the NY-ESO-1 antigen expressed by HLA-A2*0201 and NY-ESO-1 positive malignant cells. Recognition of the cognate antigen will result in the release of effector molecules from T cells, like the cytotoxic granules perforin and granzyme B, and the activation of cytokines like interferon-gamma, IL-2 and TNF-alpha.

**Manufacturing Information:** Genetically modified PBMC expressing NY-ESO-1 TCR will be manufactured by transduction of activated PBMC using the clinical grade retroviral vector MSGV1-A2aB-1G4A-LY3H10.

**Storage:** PBMC transduced for NY-ESO-1 TCR will be harvested within 96 hours from the first transduction and infused fresh. If cryopreserved, cells will be stored in a centrally-monitored liquid nitrogen freezer in the GMP suite located on the 14^th^ floor of Factor Building UCLA.

**Stability:** These genetically modified PBMC will be infused fresh. When stored in liquid nitrogen, PBMC are viable for over 2 years with minimal viability lost if not subject to freeze-thawing. Cells should be used within 6 months of manufacture.

**Dose and Schedule:** Administered once on day 0 of this protocol, receiving a minimum target cell dose of 10^8^ and up to 10^9^ PBMC transduced for NY-ESO-1 TCR.

**Administration:** Transduced cells will be transported from the GMP suite to the hospital bed in an infusion bag with over-wrap, on ice in a rigid-walled container with a tight fitting lid (i.e. Tupperware; Nalgene container etc.) labeled with a biohazard label.

**Unused Transgenic Cells.** Transduced cells generated but not administered to patients will be cryopreserved and banked for up to 5 years. Cells may be used for additional *in vitro* testing or research purposes beyond this period of time. Disposal of transgenic cells remaining in the bag and i.v. tubing after administration to patients will be done in a biohazard container that will be autoclaved before disposal.

**Prior Human Experience:** As described above, PBMC transduced with the same retrovirus vector MSGV1-A2aB-1G4A-LY3H10, is currently in clinical testing. (NCI trials: 01343043, 01350401, 00670748, 01352286, 01697527 and 02070406). Similarly, CD4 cells trained *in vivo* to recognize the NY-ESO-1 peptide was reported to induce a remission in a patient with metastatic melanoma ([Hunder et al., 2008](#_ENREF_48)).

**Reported Adverse Events and Potential Risks:** Potential risks are derived from the viral vector, the transgenes it will express and the infusion of genetically modified cells:

- **Retroviral Vectors:** There are two potential serious anticipated risks associated with retroviral vectors. One is related to its ability to randomly and permanently integrate in the genome of target cells, which may lead to altered expression of genes in these cells potentially inducing oncogenic changes. The second one is related to the possibility of its recombination and generation of replication competent virus that could potentially lead to an HIV-like disease.
  - **Risk of Insertional Mutagenesis with Retroviral Vectors.** The risks of insertional oncogenesis due to the integration of gene delivery vectors is likely a complex function of several factors, related to the type of vector used, the promoter and other transcriptional control elements of the vector, the transgene *per se*, as well as properties of the target cells. Moloney Leukemia Virus based gamma-retroviral vectors have been shown to have a high predilection for insertion into the 5’ region of actively transcribed genes, which may increase the risks for trans-activation of transcription from the cellular gene promoter ([Wu et al., 2003](#_ENREF_115)). Genes that modulate cell proliferation, such as the gammaC gene used to treat X-linked SCID may provide one component of a proliferative signal that plays a cooperative role in cellular transformation with cellular genes involved in cellular proliferation, such as LMO-2, when activated by insertional trans-activation ([Hacein-Bey-Abina et al., 2003](#_ENREF_47)). In contrast, effector proteins such as the TCR would not, *a priori*, be expected to confer an unregulated proliferative stimulus, although they may play a role in clonal expansion upon encounter with the cognate antigen.
  - **Risk of Replication Competent Retrovirus (RCR) with Retroviral Vectors.** Another risk is that genetic recombination within components of the vector system could lead to the generation of an RCR. This event would have the potential of generating infective retrovirus that may lead to oncogenesis or immune deficiency. Our patients will be screened at baseline to be HIV seronegative. Since there will be no wild type HIV virus present, the possibility of providing the missing genes for the generation of infective retrovirus *in vivo* would be lower than when retroviral vectors are administered to HIV positive subjects. RCR may be more likely to arise during packaging, when all of the components of an intact vector are present. Our vector lot has been screened for RCR per FDA guidelines, thus minimizing this exposure risk to subjects.
- **NY-ESO-1 TCR:** The main potential toxicities from the transgenic alpha and beta NY-ESO-1 TCR genes expressed in PBMC are derived from the NY-ESO-1 specificity of the TCR, or the mis-pairing with endogenous TCR genes leading to cells with potential autoimmune specificity.
  - **Autoimmune Toxicity against NY-ESO-1 Positive Cells.** Toxic events could be potentially derived from cytotoxic activity of the transgenic PBMC against NY-ESO-1 positive cells. However, studies to date have not detected expression of NY-ESO-1 in adult somatic tissues ([Bolli et al., 2005](#_ENREF_15); [Jungbluth et al., 2001](#_ENREF_52); [Satie et al., 2002](#_ENREF_98)). While spermatogonia express the NY-ESO-1 protein, the testes do not express MHC-1, thereby markedly decreasing the potential of a cellular immune response against the testes. Furthermore, a trial conducted at the NCI in which 17 patients underwent ACT with a TCR directed against NY-ESO-1_157-165_ did not detect any grade 3 or 4 toxicities, and there were no toxicities attributed to the transferred cells ([Robbins et al., 2015](#_ENREF_88)).
  - **Autoimmune Toxicity from Mispairing of TCR Chains.** The alpha and beta chains of the NY-ESO-1 TCR could undergo heterologous pairing with endogenous alpha and beta chains of TCR expressed by the transduced T cells, leading to misdirected T cells with newly acquired TCR specificities. These would be unpredictable and not subjected to the thymic selection process, potentially resulting in T cells with autoreactivities. If evidence of autoimmunity developed, patients would receive immune suppressive therapy as clinically indicated based on the severity of symptoms, using medications like corticosteroids, cyclosporin-A, mycophenolate mofetil, anti-TNF-alpha antibodies or anti-thymocyte globulin ([Tsai et al., 2008](#_ENREF_110)). When the TCR chains are expressed by PBSCs, it could result in allelic exclusion and the progeny T cells would not recombine or express endogenous TCRs (Don Kohn, unpublished). Therefore, the risk of TCR chain mispairing is markedly decreased with the PBSC TCR engineering approach.
- **NY-ESO-1 TCR Transgenic T Cells** The adoptive transfer of an *ex vivo* modified autologous cellular product may lead to potential toxicities related to the cell manipulation.
- Allergic Reaction and Cytokine Release Syndrome. The culture of cells *ex vivo* in media products to maintain lymphocyte viability, the use of reagents to activate cells, the retroviral transduction process, and the expression of foreign genes may lead to alterations in the cell product resulting in immediate untoward effects. The most serious would be infusion reactions leading to severe allergic reactions, or the triggering of a cytokine release syndrome. Therefore, the genetically modified cells will be re-infused in an inpatient setting, with continuous monitoring of vital signs and by experienced personnel within the Hematology-Oncology PBSC unit.
- **Contaminated Cell Product**

*Ex vivo* culture and manipulation of cells may result in a cell product contaminated by bacteria, fungus, mycoplasma or virus. With the goal of detecting potential contaminants, the genetically modified cells will undergo intensive lot release testing prior to infusion. Patients will be monitored carefully after cell infusion for potential signs of microbial infection, for example fever, respiratory distress or hypotension.

## CD34+ Cells Expressing NY-ESO-1 TCR/sr39TK (LV-NYESO TCR/sr39TK PBSC)

**Overview.** The investigational product is patient-specific and corresponds to autologous CD34+ cells transduced *ex vivo* with the LV-optNYESOTCR/TK vector containing the human NY-ESO-1 TCR gene. The product will be cryopreserved in final formulation, ready for the intended medical use. The starting materials used for the production of the investigational product consist of the viral vector and the patient’s CD34+ cells isolated from G-CSF and plerixafor mobilized PBMC using standard protocols for stem cell enrichment (CliniMACS^®^ CD34 reagent system).

### Lentiviral Vector pRRL-MSCV-optNYESO optsr39TK-WPRE-1

**Plasmid map pRRL-MSCV-optNYESO optsr39TK-WPRE-1**

9417 bp

**Product Description.** The lentiviral vector LV-optNYESOTCR/TK consists of 6199 bps including the 3^rd^ generation lentiviral self-inactivating (SIN) LTRs, internal promoter from the MSCV, packaging signal with the splicing donor and splicing acceptor sites, alpha chain and beta chain genes of the NY-ESO-1 TCR from Tumor Infiltrating Lymphocyte (TIL) clone 1G4 α95:LY, and the PET tracer marker/suicide gene sr39TK. The alpha and beta TCR chains and the sr39TK genes are linked by 2A self-cleaving sequences (P2A and T2A, respectively). Above is a schematic of plasmid encoding this vector and a schematic of the integrated proviral part of the vector.

**Classification:** Immunotherapeutic.

**Active Ingredient.** This lentiviral vector has three active transgenes, the alpha and beta chains of a high affinity TCR that jointly recognizes the NY-ESO-1_157-165_ epitope in the context of HLA- A*0201, and the HSV-1-sr39TK gene which serves both as a PET reporter and a suicide gene in case the gene modified PBSCs need to be eliminated.

- **NY-ESO-1 TCR α and β chains:** This is the same 1G4 high affinity TCR pair specific for NY-ESO-1_157-165_ presented by HLA-A*0201, originally cloned from a patient with metastatic melanoma (9), which is used for the gene modification of PBMC. However, these transgenes were codon-optimized and inserted into a lentiviral vector.
- **HSV1-sr39TK:** The mutated HSV1-sr39TK (sr39TK) is more efficient than the parental HSV-1-TK enzyme in the utilization of acycloguanosines as substrates ([Gambhir et al., 2000](#_ENREF_40)). Moreover, improved imaging of genetically labeled cells using PET imaging as well as greater suicide gene activity have been demonstrated with HSV-1-sr39TK. The difference between the native HSV-1-TK and the mutant sr39TK are 7 point mutations resulting in changes in 5 amino acids ([Gambhir et al., 2000](#_ENREF_40); [Yaghoubi et al., 2009](#_ENREF_120)). Important features are that the PET reporter/suicide transgene is carried into the progeny of expanding T cells without dilution of the signal and that this gene provides an improved safety feature for the lentivirally-transduced progeny of PBSC.

**Mode of Action:** The genetically modified PBSC expressing NY-ESO-1 TCR and HSV-1-sr39TK will be expected give rise to a renewable population of T cells expressing NY-ESO-TCR and HSV-1-sr39TK. One functional complex will be generated by the active transgenes:

- **NY-ESO-1 TCR α and β chains:** The NY-ESO-1 TCR is expected to direct the specificity of the T cell progeny of the TCR transgenic PBSC to the NY-ESO-1_157-165_ peptide presented by HLA-A2*0201 on the surface of malignant cells. Recognition of the cognate antigen will result in the release of effector molecules from T cells, like the cytotoxic granules perforin and granzyme B, and the activation of cytokines like interferon-gamma, IL-2 and TNF-alpha.
- **HSV-1-sr39TK:** The HSV-1-TK gene has been extensively used in clinical trials as a suicide gene ([Berger et al., 2006](#_ENREF_10); [Bonini et al., 1997](#_ENREF_16); [Giralt et al., 1999](#_ENREF_42); [Tiberghien et al., 2001](#_ENREF_109); [Verzeletti et al., 1998](#_ENREF_112)). The mutated sr39TK is more efficient than the parental enzyme in the utilization of acycloguanosines as substrates ([Black et al., 1996](#_ENREF_13)) improving the imaging of sr39TK genetically labeled cells using PET imaging ([Bonini et al., 1997](#_ENREF_16); [Dubey et al., 2003](#_ENREF_32); [Kim et al., 2004a](#_ENREF_55); [Lee et al., 2005](#_ENREF_64); [Prins et al., 2008](#_ENREF_81); [Radu et al., 2007](#_ENREF_83); [Shu et al., 2005](#_ENREF_104); [Shu et al., 2009](#_ENREF_105)). The HSV-1-TK gene marking and imaging approach has been successfully taken into patients by UCLA and Stanford investigators involved in the current proposal ([Yaghoubi et al., 2009](#_ENREF_120)).

**Manufacturing Information for the Clinical Grade Vector:** The clinical grade LV- optNYESOTCR/TK will be generated at the IUVPF by transient transfection in HEK293 cells using the packaging plasmids (pMDL, pMDG1, and pRSV-Rev) and the pRRL-MSCV-optNYESO-optsr39TK-WPRE vector. The vector will be pseudotyped with the VSV-G protein. Supernatant from the transfected cells will be harvested, filtered, benzonase treated to remove residual plasmid DNA, concentrated by tangential flow filtration and formulated in Ex-vivo 15 serum free medium. The lentiviral vector supernatant LV-optNYESOTCR/TK will be prepared and preserved following cGMP conditions at IUVPF. The supernatant will be stored upon the completion of production at least at –70^o^ C at IUVPF. Upon shipment on dry ice, the supernatant is stored at –80ºC in the HGCTF. This facility is equipped with around-the-clock temperature monitoring. Supernatant is thawed and used for *ex-vivo* transduction of activated PBSC. There will be no re-use of the same unit of supernatant for different patients. Handling of the vector should follow the guidelines of Biosafety Level-2 (BSL- 2).

**How Supplied:** The clinical grade vector will be supplied by IUVPF following their SOP for lentiviral vector suspension, labeling and cryopreservation.

**Storage:** Single use aliquots will be stored in a viral bank established in a dedicated –80^o^C freezer with a central, computerized monitoring system, with alarm and recording of all GMP storage systems located at the restricted access GMP Suite, 14th floor of the Factor Building at UCLA. **Stability:** Under the conditions of continuous storage at –80^o^C without freeze-thawing, it is expected that the lentiviral vector is stable for at least 5 years. Aliquots of this vector will be recertified annually throughout the study period with selected assays for purity, identity and potency.

### LV-NYESO TCR/sr39TK PBSC

**Product Description.** Autologous CD34+ cells will be purified from G-CSF mobilized PBSC using standard protocols for stem cell enrichment (CliniMACS^®^ CD34 reagent system), activated for 18±6h in serum-free medium, supplemented with the following cytokines: SCF (stem cell factor or c-kit ligand), FLT3L (FLT3 ligand), TPO (thrombopoietin) and IL-3, and transduced with the clinical grade LV-optNYESOTCR/TK vector supernatant for 18±6 h. Following transduction, the cells will be washed and formulated in cryopreservation solution, CryoStor® CS5. The formulated cell suspension will be filled into cryobag/s. The cryobag/s will be sealed in a secondary overwrap bag/s and placed in a metal cartridge/s. LV-NYESO TCR/sr39TK PBSC product will be cryopreserved using a controlled-rate freezer and stored in the vapor phase of a liquid nitrogen tank until ready to use. A quality control (QC) sample will be reserved for lot release testing i.e. purity, transduction efficiency, viability, and sterility.

**Classification:** Immunotherapeutic.

**Active Ingredient:** As described for the lentiviral vector LV-optNYESOTCR/TK.

**Mode of Action:** The genetically modified CD34+ cells expressing a transgenic NY-ESO-1 TCR are expected to give rise to T cells recognizing the NY-ESO-1 antigen presented by HLA-A2*0201 in NY-ESO-1 positive malignant cells. Recognition of the cognate antigen will result in the release of effector molecules from T cells, like the cytotoxic granules perforin and granzyme B, and the activation of cytokines like interferon-gamma, IL-2 and TNF-alpha.

**Manufacturing Information:** Genetically modified CD34+ PBSC transgenic for NY-ESO-1 TCR will be manufactured by transduction of activated CD34+ cells using the clinical grade lentiviral vector LV-optNYESOTCR/TK.

**Storage:** CD34+ cells transgenic for NY-ESO-1 TCR/sr39TK will be harvested and cryopreserved in the infusion bag. Cells will be stored in a centrally-monitored liquid nitrogen freezer located on the 14^th^ floor of Factor Building, UCLA.

**Stability:** The cryopreserved LV-NYESO TCR/sr39TK cell product stored in the vapor phase of LN2 is stable for up to 6 months post-cryopreservation and for up to 6h at 4-8°C or room temperature post-thaw. The cells will be used within 6 months of manufacture.

**Schedule and Dose**: LV-NYESO TCR/sr39TK PBSC will be administered once on day 0. The dose administered to each patient will be ≥2.0 - 6.0x10^6^ LV-NYESO TCR/sr39TK PBSC/kg.

The numbers of cells are based on the average number of CD34+ cells in the mobilized leukapheresis product from healthy donors that varies between 2.0 -13.0 ×10^6^/kg ([Kroger et al., 2002](#_ENREF_62)).

**Administration of transduced CD34+**: The final cell product will be transported in dry cryogenic container to the infusion site. It will be thawed at bedside per PBSC transplant routine. The study agent will be infused at the completion of thawing by i.v. injection over 10-30 minutes. The entire dose of cells will be administered; there will be no need to store and re-use “left-over” cells. During the administration period and afterwards, the subject will be monitored for any adverse events, such as allergic reaction(s) according to the standard clinical practice at UCLA. Because the study agent is a biologic, care must be taken in handling this product, such as using gloves. After the cells have been administered, the empty infusion bag will be disposed of as biohazardous waste.

**Prior Clinical Experience:** There are a number of current clinical trials that utilize lentivirus transduced hematopoietic stem cells in the treatment of various pathological conditions i.e NCT01410825 (WAS gene transfer to correct Wiskott-Aldrich Syndrome), NCT02247843 (modified beta-globin gene transfer to correct Sickle Cell Disease), NCT01560182 (ARSA gene transfer to correct Metachromatic Leukodystrophy), etc. No previous clinical trials have been performed with this particular clinical product.

## Reported Adverse Events and Potential Risks

**PBMC and PBSC Transgenic for NY-ESO-1 TCR:** Potential risks are derived from the viral vectors, the transgenes they will express, and the infusion of genetically modified cells into humans.

### Retroviral Vectors

There are two potential serious anticipated risks associated with retroviral vectors. One is related to its ability to randomly and permanently integrate in the genome of target cells, which may lead to altered expression of genes in these cells potentially inducing oncogenic changes. The second one is related to the possibility of its recombination and generation of replication competent virus that could potentially lead to an HIV-like disease.

- - **Risk of Insertional Mutagenesis with Retroviral Vectors.** The risk of insertional oncogenesis due to the integration of gene delivery vectors is likely a complex function of several factors, related to the type of vector used, the promoter and other transcriptional control elements of the vector, the transgene *per se*, as well as properties of the target cells. Moloney Leukemia Virus (MLV)-based gamma-retroviral vectors have been shown to have a high predilection for insertion into the 5’ region of actively transcribed genes, which may increase the risk for trans-activation of transcription from the cellular gene promoter ([Wu et al., 2003](#_ENREF_115)). Genes that modulate cell proliferation, such as the gammaC gene used to treat X-linked SCID may provide one component of a proliferative signal that plays a cooperative role in cellular transformation with cellular genes involved in cellular proliferation, such as LMO-2, when activated by insertional trans-activation ([Hacein-Bey-Abina et al., 2003](#_ENREF_47)). In contrast, effector proteins such as the TCR would not, *a priori*, be expected to confer an unregulated proliferative stimulus, although they may play a role in clonal expansion upon encounter with the cognate antigen.
  - **Risk of Replication Competent Retrovirus (RCR) with Retroviral Vectors.** Another risk is that genetic recombination within components of the vector system could lead to the generation of an RCR. This event would have the potential of generating an infective retrovirus that may lead to oncogenesis or immune deficiency. Our patients will be screened at baseline to ensure HIV seronegativity. Since there will be no wild type HIV virus present, the possibility of providing the missing genes for the generation of infective retrovirus *in vivo* is almost none. RCR may be more likely to arise during packaging, when all the components of an intact vector are present. Our vector lot has been screened for RCR per FDA guidelines, thus minimizing this exposure risk to subjects.

### Lentiviral Vectors

Similar to retroviral vectors, the potential risks of lentiviral vectors are insertional mutagenesis and the risk of recombination and generation of replication competent virus.

- **Risk of Insertional Mutagenesis with Lentiviral Vectors.** The risk of insertional mutagenesis is lower than that expected for retroviral vectors based on *in vitro* and *in vivo* experimental models using murine and human cells. There is a possibility that lentiviral vectors may carry a lower risk of insertional mutagenesis than retroviruses based on some studies in mice. No cases in humans have been reported to date ([Aiuti et al., 2012](#_ENREF_1); [Naldini, 2011](#_ENREF_77)).
- **Risk of Replication Competent Lentivirus (RCL) with Lentiviral Vectors.** The risk of RCL is similar to that expected for RCR. Since patients are screened at baseline to ensure HIV seronegativity, this risk is greatly reduced.

### NY-ESO-1 TCR

The main potential toxicities from the transgenic alpha and beta NY-ESO-1 TCR genes expressed in PBMC are derived from the NY-ESO-1 specificity of the TCR, or the mispairing with endogenous TCR genes leading to cells with potential autoimmune specificity.

- - **Autoimmune Toxicity against NY-ESO-1 Positive Cells.** Toxic events could be potentially derived from cytotoxic activity of the transgenic PBMC against NY-ESO-1 positive cells. However, studies to date have not detected expression of NY-ESO-1 peptide in adult somatic tissues ([Bolli et al., 2005](#_ENREF_15); [Jungbluth et al., 2001](#_ENREF_52); [Satie et al., 2002](#_ENREF_98)). While spermatogonia express the NY-ESO-1 protein, the testes do not express MHC-1, thereby markedly decreasing the potential of a cellular immune response against the testes. Furthermore, a recent trial conducted at the NCI in which 17 patients underwent ACT with a TCR directed against NY-ESO-1_157-165_ did not detect any grade 3 or 4 toxicities related to NY-ESO-1 recognition ([Robbins et al., 2011a](#_ENREF_90)). A recent publication indicated that in follow up there were no toxicities attributed to the transferred cells ([Robbins et al., 2015](#_ENREF_88)).
  - **Autoimmune Toxicity from Mispairing of TCR Chains.** The alpha and beta chains of the NY-ESO-1 TCR could undergo heterologous pairing with endogenous alpha and beta chains of TCR expressed by the transduced T cells, leading to misdirected T cells with newly acquired TCR specificities. These would be unpredictable and not subjected to the thymic selection process, potentially resulting in T cells with autoreactivities. If evidence of autoimmunity developed, patients would receive immune suppressive therapy as clinically indicated based on the severity of symptoms, using medications like corticosteroids, cyclosporin-A, mycophenolate mofetil, anti-TNF-alpha antibodies or anti-thymocyte globulin ([Tsai et al.](#_ENREF_110)). When the TCR chains are expressed by PBSCs, it could result in allelic exclusion and the progeny T cells would not recombine or express endogenous TCRs ([Giannoni et al., 2013](#_ENREF_41)). Therefore, the risk of TCR chain mispairing is markedly decreased with the PBSC TCR engineering approach.

### sr39TK

The HSV-1-sr39TK transgene has been transferred into human subjects. A replication competent oncolytic adenoviral vector encoding the HSV-1-sr39TK gene has been administered to subjects intratumorally ([Barton et al., 2008](#_ENREF_7); [Freytag et al., 2007](#_ENREF_37)). There were no dose-limiting toxicities or serious adverse events. The most common side effects included flu-like symptoms, transaminitis, and pancytopenia. In general, the treatment was well tolerated with only transient side-effects.

### RV-NYESO TCR PBMC

The adoptive transfer of an *ex vivo* modified autologous cellular product may lead to potential toxicities related to the cell manipulation.

#### Allergic Reaction and Cytokine Release Syndrome.

The culture of cells *ex vivo* in media products to maintain lymphocyte viability, the use of reagents to activate cells, the retroviral transduction process, and the expression of foreign genes may lead to alterations in the cell product resulting in immediate untoward effects. The most serious would be infusion reactions leading to severe allergic reactions, or the triggering of a cytokine release syndrome. Therefore, the genetically modified cells will be re-infused in an inpatient setting, with continuous monitoring of vital signs and by experienced personnel within the Hematology-Oncology PBSC unit.

#### Contaminated Cell Product.

*Ex vivo* culture and manipulation of cells may result in a cell product contaminated by bacteria, fungus, mycoplasma or virus. Cell products will be tested for purity prior to infusion. Patients will be monitored carefully after cell infusion for signs of microbial infection, for example fever, respiratory distress or hypotension.

## LV-NYESO TCR/sr39TK PBSC

The adoptive transfer of this *ex vivo* modified autologous cellular product may lead to potential toxicities related to the cell manipulation as described above for allergic reactions, cytokine release syndrome and contaminated product. The TCR transgenic PBSCs may have additional potential risks as follows:

### Amplification of Toxicities.

Since PBSC cells are self-renewing, it is possible that were one of the aforementioned toxicities to occur, the effect might be amplified. The sr39TK gene was engineered into the lentiviral vector to be used as a suicide gene in the event that side effects requiring their elimination should occur.

### Marrow Recovery Failure.

If criteria for non-engraftment are met (persistent ANC < 500/μl or platelets < 50,000/ul at Day +28, a bone marrow biopsy will be performed to confirm engraftment failure. Once this is confirmed, the previously cryopreserved unmodified CD34+ cells will be thawed and re-infused according to the clinical laboratory standard operating procedure. The banked product will contain a minimum of 2.0 x10^6^ cells/kg body weight, which is sufficient to regain normal hematopoiesis. In the very rare and unanticipated event that the banked unmodified cells fail to engraft, an emergent allogeneic hematopoietic stem cell transplant would be pursued.

## ADDITIONAL STUDY AGENTS

### IL-2 (aldesleukin, Proleukin, recombinant human Interleukin 2)

**Description:** Please refer to package insert for complete product information. Human recombinant IL-2 is a highly purified protein with a molecular weight of approximately 15,300 daltons. It is a lymphokine produced by recombinant DNA technology using a genetically engineered *E. coli* strain containing an analog of the human IL-2 gene.

**Mode of Action:** Immunotherapeutic agent that stimulates T and NK cell responses.

**How Supplied:** IL-2 is manufactured by Prometheus.

**Formulation/Reconstitution:** IL-2 is provided as single-use vials containing 22 million IU (~1.3 mg) IL-2 as a sterile, white to off-white lyophilized cake plus 50 mg mannitol and 0.18 mg sodium dodecyl sulfate, buffered with approximately 0.17 mg monobasic and 0.89 mg dibasic sodium phosphate to a pH of 7.5 (range 7.2 to 7.8). The vial is reconstituted with 1.2 mL of Sterile Water for Injection, USP, and the resultant concentration is 18 million IU/mL or 1.1 mg/mL. Diluent should be directed against the side of the vial to avoid excess foaming. Swirl contents gently until completely dissolved. Do not shake. Since vials contain no preservative, reconstituted solution should be used within 24 hours.

**Storage:** Intact vials are stored in the refrigerator (2^o^ – 8^o^C) protected from light. Each vial bears an expiration date.

**Dilution/Stability:** Reconstituted IL-2 should be further diluted with 50 mL of 5% Human Serum Albumin (HSA). The HSA should be added to the diluent prior to the addition of recombinant IL-2. Dilutions of the reconstituted solution over a 1000-fold range (i.e., 1 mg/mL to 1 µg/mL) are acceptable in either glass bottles or polyvinyl chloride bags. IL-2 is chemically stable for 48 hours at refrigerated and room temperatures, 2^o^ to 30^o^C.

**Dose and Schedule:** 500,000 IU/m^2^ q12 hours for a maximum of 14 doses.

**Administration:** The final dilution of IL-2 will be given IV.

**Toxicities:** Expected toxicities of IL-2 are listed in the product label. Grade III toxicities common to IL-2 include diarrhea, nausea, vomiting, hypotension, skin changes, anorexia, mucositis, dysphagia, or constitutional symptoms and laboratory changes.

### Busulfan (Busulfex^®^)

**Description:** Please refer to package insert for complete product information. Busulfan is an alkylating agent indicated for use in combination with cyclophosphamide as a conditioning regimen prior to allogeneic hematopoietic progenitor cell transplantation for chronic myelogenous leukemia.

**Mode of Action:** Busulfan is a bifunctional alkylating agent in which two labile methanesulfonate groups are attached to opposite ends of a four-carbon alkyl chain. In aqueous media, busulfan hydrolyzes to release the methanesulfonate groups. This produces reactive carbonium ions that can alkylate DNA. DNA damage is thought to be responsible for much of the cytotoxicity of busulfan.

**How Supplied:** Busulfex^®^ is packaged as a sterile solution in 10 mL single-use clear glass vials each containing 60 mg of busulfan at a concentration of 6 mg per mL for intravenous use, NDC 59148-070-90. Busulfex^®^ is distributed as a unit carton of eight vials NDC 59148-070-91.

**Stability:** Busulfex^®^ diluted in 0.9% Sodium Chloride Injection, USP or 5% Dextrose Injection, USP is stable at room temperature (25°C) for up to 8 hours but the infusion must be completed within that time. Busulfex^®^ diluted in 0.9% Sodium Chloride Injection, USP is stable at refrigerated conditions (2°C to 8°C) for up to 12 hours but the infusion must be completed within that time.

**Storage:** Unopened vials of Busulfex^®^ must be stored under refrigerated conditions between 2°C to 8°C (36°F to 46°F).

**Dose and Schedule:** 2 mg/kg per dose in two 12 hourly doses for 3 days (= total of 6 doses) intravenously via a central venous catheter.

The total AUC that can be reached in 3 days is 70,000-80,000 ng/ml x hr (16,666-19,048 μM/min), which is nearly ablative (99 % of cytoxicity for CD34+ cells). The targeted AUC after a single dose would be 12,000 – 13,000 ng/ml x hr (2,857-3,095 μM/min) where no side effects have been previously observed.

The AUC will be calculated after the first dose, and the dose will be adapted to reach the target net AUC of 70,000-80,000 ng/ml x hr. The AUC will be re-measured after the fifth or sixth dose to estimate total exposure. Optimizing the conditioning regimen will be an important factor for successfully achieving long-term engraftment of the gene-modified cells.

Busulfan levels will be measured in serum or plasma by LC, GC, or MS methods. Blood samples must be centrifuged immediately and frozen at -20°C. Optimal time points for sample collection and calculation of AUC are a) immediately at the end of the infusion (time zero, for calculation after the 5^th^ or 6^th^ dose, a sample must be drawn prior to administration of the that dose) and b) 4,6 and 8 hours after the end of busulfan infusion. The equivalent of the AUC in mg/kg unit will be also provided to the investigators.

A designated UCLA Pharmacist will calculate the AUCs and prescribe the necessary dose changes for all gene therapy patients in real time, if necessary. The busulfan levels will be measured and results transmitted to the designated UCLA Pharmacist for calculation of AUC and dose adjustment**.**

**Administration:** Busulfan will be administered i.v. starting with a dosage of 2 mg/kg per dose in two 12 hourly doses for 3 days (= total of 6 doses). A wash-out period of at least 24 hours after completion of the 6^th^ busulfan dose infusion will be maintained prior to reinfusion of the transduced cells.

Premedicate patients with anticonvulsants (e.g., benzodiazepines, phenytoin, valproic acid or levetiracetam) to prevent seizures reported with the use of high dose Busulfex^®^. Administer anticonvulsants 12 hours prior to infusion of Busulfex^®^. Administer antiemetics prior to the infusion of Busulfex^®^.

**DO NOT USE POLYCARBONATE SYRINGES OR POLYCARBONATE FILTER NEEDLES WITH BUSULFEX.**

Use an administration set with minimal residual hold-up volume (2-5 cc) for product administration. Busulfex^®^ is a cytotoxic drug. Follow applicable special handling and disposal procedures. Skin reactions may occur with accidental exposure. Use gloves when preparing Busulfex^®^. If Busulfex^®^ or diluted Busulfex^®^ solution contacts the skin or mucosa, wash the skin or mucosa thoroughly with water. Visually inspect parenteral drug products for particulate matter and discoloration prior to administration whenever the solution and container permit. Do not use if particulate matter is seen in the Busulfex^®^vial.

Busulfex^®^must be diluted prior to intravenous infusion with either 0.9% Sodium Chloride Injection, USP (normal saline) or 5% Dextrose Injection, USP (D5W). The diluent quantity should be 10 times the volume of Busulfex^®^ so that the final concentration of busulfan is approximately 0.5 mg per mL. Calculation of the dose for a 70 kg patient would be performed as follows:

(70 kg patient) x (0.8 mg per kg) ÷ (6 mg per mL) =9.3 mL Busulfex^®^(56 mg total dose).

To prepare the final solution for infusion, add 9.3 mL of Busulfex^®^ to 93 mL of diluent (normal saline or D5W) as calculated below:

(9.3mL Busulfex^®^) x (10) =93 mL of either diluent plus the 9.3 mL of Busulfex^®^to yield a final concentration of busulfan of 0.54 mg per mL (9.3 mL x 6 mg per mL ÷ 102.3 mL =0.54 mg per mL).

All transfer procedures require strict adherence to aseptic techniques, preferably employing a vertical laminar flow safety hood while wearing gloves and protective clothing. DO NOT put the Busulfex^®^into an intravenous bag or large-volume syringe that does not contain normal saline or D5W. Always add the Busulfex^®^to the diluent, not the diluent to the Busulfex^®^. Mix thoroughly by inverting several times. Infusion pumps should be used to administer the diluted Busulfex^®^solution. Set the flow rate of the pump to deliver the entire prescribed Busulfex^®^ dose over two hours. Prior to and following the infusion, flush the indwelling catheter line with approximately 5 mL of 0.9% Sodium Chloride Injection, USP or 5% Dextrose Injection, USP. DO NOT infuse concomitantly with another intravenous solution of unknown compatibility.

**WARNING: RAPID INFUSION OF BUSULFEX HAS NOT BEEN TESTED AND IS NOT RECOMMENDED.**

**Toxicities:** The most prevalent adverse reactions include myelosuppression, seizures in patients receiving high dose oral busulfan, Hepatic Veno-Occlusive Disease (HVOD) in patients with high busulfan plasma concentration verses time curve values (AUC), embryo-fetal toxicity, cardiac tamponade in pediatric patients with thalassemia receiving high dose oral busulfan and cyclophosphamide, bronchopulmonary dysplasia (rare) and cellular dysplasia in many organs.

### Fludarabine

**Description:** Please refer to package insert for complete product Information. Fludarabine phosphate is a synthetic purine nucleoside that differs from physiologic nucleosides in that the sugar moiety is arabinose instead of ribose or deoxyribose. Fludarabine is a purine antagonist antimetabolite.

**Mode of Action:** Chemotherapy agent.

**How Supplied:** Fludarabine is supplied by Bayer in a 50 mg vial as a fludarabine phosphate powder in the form of a white, lyophilized solid cake.

**Stability:** Following reconstitution with 2 mL of sterile water for injection at a concentration of 25 mg/ml, the solution has a pH of 7.7. The fludarabine powder is stable for at least 18 months at 2-8^o^C; when reconstituted, fludarabine is stable for at least 16 days at room temperature. Because no preservative is present, reconstituted fludarabine will typically be administered within 8 hours. Specialized references should be consulted for specific compatibility information. Fludarabine is dephosphorylated in serum, transported intracellularly and converted to the nucleotide fludarabine triphosphate; this 2-fluoro-ara-ATP molecule is thought to be required for the drug’s cytotoxic effects. Fludarabine inhibits DNA polymerase, ribonucleotide reductase, DNA primase, and may interfere with chain elongation, and RNA and protein synthesis.

**Storage:** Intact vials should be stored refrigerated (2-8^o^C).

**Dose and Schedule:** 40 mg/m^2^/day for 4 days. The dose of fludarabine will be adjusted based on estimation of the creatinine clearance (CrCl). For CrCl 30-70 the dose should be reduced by 20%, and for CrCl <30 – not given.

**Administration:** Fludarabine is administered as an i.v. infusion in 100 ml 0.9% sodium chloride, USP over 15 to 30 minutes. To prevent undue toxicity, the dose will be based on body surface area, but will not exceed a dose calculated on surface areas based on body weights greater than 140% of the maximum ideal body weight per height and weight.

**Toxicities:** At doses of 40 mg/m^2^/day for 4 days, the primary side effect is myelosuppression; however, thrombocytopenia is responsible for most cases of severe and life-threatening hematologic toxicity. Serious opportunistic infections have occurred in patients with chronic lymphocytic leukemia (CLL) treated with fludarabine. Hemolytic anemia has been reported after one or more courses of fludarabine with or without a prior history of a positive Coomb’s test; fatal hemolytic anemia has been reported. In addition, bone marrow fibrosis has been observed after fludarabine therapy. Other common adverse effects include malaise, fever, chills, fatigue, anorexia, nausea and vomiting, and weakness. Irreversible and potentially fatal central nervous system toxicity in the form of progressive encephalopathy, blindness, and coma is only rarely observed at the currently administered doses of fludarabine. More common neurologic side effects at the current doses of fludarabine include weakness, pain, malaise, fatigue, paresthesia, visual or hearing disturbances, and sleep disorders. Adverse respiratory effects of fludarabine include cough, dyspnea, allergic or idiopathic interstitial pneumonitis. Tumor lysis syndrome has been rarely observed in fludarabine treatment of CLL. Treatment on previous adoptive cell therapy protocols in the Surgery Branch/NCI have caused persistently low (below 200) CD4 counts, and one patient developed polyneuropathy manifested by vision blindness, and motor and sensory defects ([Dudley et al., 2002b](#_ENREF_34); [Dudley et al., 2005](#_ENREF_35)).

### Filgrastim [G-CSF] (Neupogen^®^) or Other Biosimilar

**Description:** Please refer to package insert for complete product information. Neupogen^®^ (Amgen) is human recombinant granulocyte colony stimulating factor (rhuG-CSF) used to mobilize autologous hematopoietic progenitor cells into the peripheral blood for collection by leukapheresis.

**Mode of Action:** G-CSF regulates the production of neutrophils within the bone marrow and affects neutrophil progenitor proliferation and differentiation. It mobilizes hematopoietic progenitor cells into the peripheral blood.

**How Supplied:** Single-dose‚ preservative-free, prefilled syringe with 27 gauge, ½ inch needle with an UltraSafe Needle Guard, containing 300 mcg/0.5 mL of filgrastim.

**Stability:** Prior to use‚ remove the vial or prefilled syringe from the refrigerator and allow G-CSF to reach room temperature for a minimum of 30 minutes and a maximum of 24 hours. Discard any vial or prefilled syringe left at room temperature for greater than 24 hours. Visually inspect G-CSF for particulate matter and discoloration prior to administration (the solution is clear and colorless). Do not administer G-CSF if particulates or discoloration are observed. Discard unused portion of G-CSF in vials or prefilled syringes; do not re-enter the vial. Do not save unused drug for later administration.

**Storage:** Store G-CSF at 2° to 8°C (36° to 46°F) in the carton to protect from light. Do not leave G-CSF in direct sunlight. Avoid freezing; if frozen, thaw in the refrigerator before administration. Discard G-CSF if frozen more than once. Avoid shaking.

**Dose and Schedule:** 8-10µg/kg/day subcutaneous injection. Administer for at least 4 days before first leukapheresis procedure and continue until last leukapheresis. Monitor neutrophil counts after 4 days of G-CSF‚ and discontinue G-CSF if the white blood cell (WBC) count rises to greater than 100‚000/mm^3^.

**Administration:** Inject G-CSF subcutaneously in the outer area of upper arms, abdomen, thighs, or upper outer areas of the buttock. Persons with latex allergies should not administer the G-CSF prefilled syringe, because the needle cap contains dry natural rubber (derived from latex).

**Toxicities:** The most prevalent adverse reactions (≥ 5% incidence) that have occurred in patients with cancer undergoing autologous peripheral blood progenitor cell collection using G-CSF have been bone pain, pyrexia, bone alkaline phosphatase increase and headache.

### Plerixafor (Mozobil)

**Description:** Please refer to package insert for complete product information. Plerixafor (Genzyme) is a hematopoietic stem cell mobilize and is used in combination with grulocyte-colony stimulating factor (G-CSF) to mobilize hematopoietic stem cells to the perpheral blood for collection.

**Mode of Action:** Plerixafor is an inhibitor of the CXCR4 chemokine receptor and blocks binding of its

cognate ligand, stromal cell-derived factor-lq (SDF-1a). SDF-la and CXCR4 are recognized to play a role in the trafficking and homing of human peripheral blood stem cells (PBSCs) to the marrow compartment. Once in the marrow, stem cell CXCR4 can act to help anchor these cells to the marrow matrix, either directly via SDF-1α or through the induction of other adhesion molecules. Treatment with plerixafor results in leukocytosis and elevations in circulating hematopoietic progenitor cells.

**How Supplied:** Each single-use vial is filled to deliver 1.2 mL of 20 mglmL solution containing 24 mg of plerixafor.

**Stability:** Each vial of plerixafor is intended for single use only. Any unused drug remaining after injection must be discarded.

**Storage:** Store at 25°C (77°F); excursions permitted to 15°-30°C (59°-86°F)

**Dose and Schedule:** 0.24 mg/kg body weight by subcutaneous (SC) injection. Use the patient's actual body weight to calculate the volume of plerixafor to be administered. Based on increasing exposure with increasing body weight, the plerixafor dose should not exceed 40 mg/day.
**Administration:** Begin treatment with plerixafor after the patient has received G-CSF once daily for four days. Administer plerixafor in the evening prior to initiation of each apheresis in the morning for up to 4 consecutive days.

**Toxicities:** The most common adverse reactions (≥ 10%) reported in patients who received plerixafor in conjunction with G-CSF regardless of causality and more frequent with plerixafor than placebo during PBSC mobilization and apheresis were diarrhea, nausea, fatigue, injection site reactions, headache, arthralgia, dizziness and vomiting.

## Laboratory Reagents

### OKT3

OKT3 (Miltenyi), is an anti-CD3 antibody used to activate T cells *in vitro* by engaging the CD3 complex. In the current clinical trial, OKT3 will be strictly used *ex vivo* for the activation of PBMC for retroviral transduction. A clinical grade OKT3 (Miltenyi) is a therapeutic monoclonal antibody approved by the FDA to treat rejection of transplanted organs, including the heart, kidneys and liver. However, this antibody is currently not being produced by the clinical grade manufacture. When used *in vivo* OKT3 antibodies eliminate CD3+ lymphocytes which is beneficial to treat graft rejections. It can induce anaphylactic or anaphylactoid reactions, cytokine release syndrome and a variety of constitutional symptoms when administered systemically. The source of OKT3 antibody for the current studies is obtained with a certificate of analysis providing its manufacturing and product characterization. In addition, the batches used within this study are further characterized by performing the following tests: gram stain, bacterial culture, fungal culture, mycoplasma culture, and endotoxin assay.

### Interleukin-2 (IL-2 )

The IL-2 used for *in vitro* stimulation of PBMC will be the same as described for systemic IL-2 treatment in patients. All pertinent information is described in that [section (9.4.1)](#_Lentiviral_Vector_pRRL-MSCV-optNYES).

### LentiBOOST^TM^

LentiBOOST^TM^ (SIRION BioTech) is a polaxamer-based adjuvant which permeabilizes cell membranes allowing lentivirus to enter cells and transduce them. The compound is receptor independent making it suitable for multiple cell types and has been previously demonstrated to enhance transduction of PBSC, HSC, and other cell types (Höfig, I. (2014), Hauber, I. (2018) and Schott, J. (2019)). Use of LentiBOOST is simple and relies on dilution of the GMP grade polymer stock 100-fold into the transduction mixture.

### RetroNectin®

RetroNectin® (CH-296, Takara/ Clontech) is a chimeric peptide of recombinant human fibronectin fragments produced in *E. coli*, consisting of three functional domains: a central cell-binding domain (type III repeat, 8-10), heparin-binding domain II (type III repeat, 12-14), and a CS1 site within the alternatively-spliced IIICS region. It is a 574 amino acid protein with a molecular weight of 63 kDa. When coated on the surface of containers such as culture dishes, petri dishes, flasks or cell culture bags, retronectin significantly enhances retrovirus-mediated gene transduction into mammalian cells. This enhancement is hypothetically due to co-localization of retroviral particles and target cells on the molecules of retronectin. Virus particles bind retronectin via interaction with heparin-binding domain II, and target cells bind mainly through the interaction of the cell surface integrin receptor VLA-4 with the fibronectin CS1 site. Additionally, cells may also bind through the interaction of another fibronectin ligand (RGDS in repeat 10) within the central cell-binding domain with a corresponding integrin receptor VLA-5 on the cell surface.

### Recombinant Human Stem Cell Factor (rhSCF)

The rhSCF (Milteneyi or equivalent) is a hematopoietic growth factor important for the survival, proliferation, and differentiation of hematopoietic stem cells and progenitor cells. GMP grade rhSCF will be used for *in-vitro* stimulation of CD34+ hematopoietic progenitor cells.

### Recombinant human Thrombopoietin (rhTPO)

The rhTPO (Milteneyi or equivalent) is a glycoprotein hormone and the major stimulator of megakaryopoiesis and platelet production. It promotes the proliferation of hematopoietic stem cells, primitive progenitors, megakaryocytes, and platelets. GMP grade rhTPO will be used for *in-vitro* stimulation of CD34+ hematopoietic progenitor cells.

**9.6.7. Recombinant human Interleukin-3 (IL-3)**

The rhIL-3 is a hematopoietic growth factor with a broad spectrum of biologic activities. These include the stimulation of the proliferation and differentiation of immature pluripotent hematopoietic stem cells and various lineage-committed progenitor cells, leading to the production of most of the major blood cell types. GMP grade rhIL-3 will be used for in-vitro stimulation of CD34+ hematopoietic progenitor cells.

**9.6.8.. Recombinant human Fms-Related Tyrosine Kinase 3 Ligand (rhFlt3L)**

The Flt3L is a growth factor that regulates early hematopoiesis. Flt3-ligand belongs to a small family of α-helical cytokines and promotes the proliferation and differentiation of primitive hematopoietic stem cells in synergy with other growth factors like G-CSF, GM-CSF, SCF and IL-3. GMP grade rhFlt3L will be used for *in-vitro* stimulation of CD34+ hematopoietic progenitor cells.

# GENERATION OF EXPERIMENTAL CELL THERAPIES

**NY-ESO-1-TCR TRANSDUCED T CELLS (RV-NYESO TCR PBMC) AND NY-ESO-1-TCR/sr39TK TRANSDUCED CD34^+^ CELLS (LV-NYESO TCR/sr39TK PBSC).**

Two types of cell therapies are included in this protocol:

The autologous PBMC from the non-mobilized leukapheresis of patients with NY-ESO-1+ advanced malignancies modified by ex vivo transduction using the retroviral vector MSGV1-A2aB-1G4A-LY3H10 (NY-ESO-1 TCR transgenic autologous T cells).

The autologous CD34+ PBSC from the G-CSF mobilized blood of patients with NY-ESO-1+ advanced malignancies modified by *ex vivo* transduction using the lentiviral vector LV-optNYESOTCR/TK (NY-ESO-1 TCRsr39TK transgenic autologous CD34+ cells).

Feasibility is a secondary endpoint of this protocol. Repetitive deviations to the procedures described in this section will be recorded in the batch records and the protocol will be modified accordingly and highlighted in the next submission to the institutional review board (IRB) and FDA and all of the appropriate regulatory committees.

## Generation of NY-ESO-1 TCR Transduced PBMC

### Leukapheresis Procedures

Patients will undergo an approximate 12 liter leukapheresis procedure to obtain PBMC for retroviral transduction. Any signs and symptoms of citrate toxicity due to apheresis (such as perioral paresthesia and muscle cramps) will be treated according to the UCLA Hemapheresis center standard procedures. The leukapheresis product will be transferred to the UCLA HGCTF for cell processing.

### PBMC Isolation

Following leukapheresis, the product will be separated to obtain the mononuclear fraction for PBMC isolation by layering the leukapheresis product over Ficoll and centrifuging at 500g for 30 minutes.

### PBMC Cell Activation.

An aliquot of PBMC obtained from the leukapheresis product will be cultured in AIMV media with 5% heat-inactivated AB serum at 10^6^ cells/mL and activated for 48 hours in the presence of OKT3 (50 ng/mL, anti-human CD3 antibody) and human IL-2 (300 IU/mL, aldesleukin) in order to stimulate T-cell growth to prepare for viral transduction.

### Clinical Grade Retrovirus MSGV1-A2aB-1G4A-LY3H10 Lot Release Testing

Prior to use, the clinical grade retroviral vector will require fulfilling the lot release criteria included in Table 4.

Table 4. Clinical Grade MSGV1-A2aB-1G4A-LY3H10 Retrovirus Lot Release Criteria

**Table 4a.** Certificate Of Analysis (COA) of the MSGV1-A2aB-1G4A-LY3H10 Master Cell Bank (MCB)

Table 4b. Certificate Of Analysis (COA) of the MSGV1-A2aB-1G4A-LY3H10 Final Product.

### Clinical Grade Retrovirus MSGV1-A2aB-1G4A-LY3H10 Annual Recertification

Single-use aliquots of the clinical grade retrovirus vector will be subjected to annual recertification, and the results of this testing will be included in the annual progress report for the FDA (Table 5).

Table 5. MSGV1-A2aB-1G4A-LY3H10 Retrovirus Annual Recertification

**Table 5a.** MSGV1-A2aB-1G4A-LY3H10 Retrovirus Annual Recertification Criteria

| Test Item | **Method** | **Limit of Detection** | **Acceptable Criteria** |
| --- | --- | --- | --- |
| Infectious viral vector titer  (Transgene expression) | NY-ESO-1_126-157_ tetramer or dextramer staining in activated PBMC | 0.03% NY-ESO-1_126-157_ tetramer or dextramer positive cells among CD3+ T lymphocytes | > 10% NY-ESO-1_126-157_ tetramer positive cells among CD3+ T lymphocytes |
| Physical viral vector titer | qRT-PCR | 5 x 10^3^ copies/µl | Report value |
| Sterility | Aerobic and anaerobic culture for bacteria and fungus | No growth within 14 days | No growth within 14 days |
| Mycoplasma | MycoAlert test |  | Negative (<1 ratio) |
| Endotoxin | Endosafe®PTS system | 0.1 EU/ml | <0.33 EU/ml for each sample |

Legend: cfu: colony-forming units; EU: endotoxin units.

**Table 5b.** MSGV1-A2aB-1G4A-LY3H10 Retrovirus Annual Recertification 2019

| Test | **Method** | **Limits** | **Results** | **Date** |
| --- | --- | --- | --- | --- |
| Infectious viral vector titer  (Transgene expression) | NY-ESO-1_126-157_ tetramer or dextramer staining in transduced PBMC | 0.03% NY-ESO-1_126-157_ tetramer or dextramer positive cells among CD3+ T lymphocytes | 59% NY-ESO-1_126-157_ tetramer positive cells among CD3+ T lymphocytes | 8/5/2019 |
| Physical viral vector titer | qRT-PCR | 5x103 viral particles/ ml | 7.53 x 105 viral particles/ ml | 9/19/2019 |
| Sterility | Aerobic and anaerobic culture for bacteria and fungus | No growth within 14 days | No growth within 14 days | 8/10/2019 |
| Mycoplasma | MycoAlert test | Negative (<1 ratio) | Negative (0.45 ratio) | 7/26/2019 |
| Endotoxin | Endosafe®PTS system | <0.33 EU/ml | <0.052 EU/ml | 9/20/2019 |

Legend: EU: endotoxin units.

### Retrovirus Transduction Method

The MSGV1-A2aB-1G4A-LY3H10 retrovirus vector lot will be stored in a monitored and locked ≤ -70ºC freezer in the UCLA HGCTF, 14^th^ floor, Factor Building (Viral Bank). Transductions will be performed in either retronectin-coated 6-well plates or bags.

**RetroNectin Coating.** Wells or bags will be pre-coated with RetroNectin**®** (Takara Bio Inc., Japan/ Clontech, USA), a recombinant chimeric fibronectin molecule. For 6-well plates: one ml of 10 μg/ml Retronectin® in sterile phosphate buffered saline is placed per well and incubated overnight at 4^o^C (alternatively at room temperature for 2 hours). For bags, Retronectin® is added to 1.7 µg/ml in sterile bags, and incubated at room temperature for 2±0.5 hours or overnight in the fridge (4-10°C). Wells or bags are blocked with 1.5 ml of HBSS with 2.5% HSA at room temperature for 30 minutes and washed with HBSS containing 2.5% HEPES.

**Retroviral Transduction.** **For 6-well plates**, 2 to 4 ml of thawed and 1:1 diluted MSGV1-A2aB-1G4A-LY3H10 retrovirus viral supernatant in TCR medium is applied to each retronectin-coated well of transduction #1 plates and centrifuged at 2000g for 2 hours at 32^o^C. Retroviral supernatant is removed from transduction #1 plates (leaving behind 1 ml), 2.0 x 10^6^ activated PBMC per well in AIMV plus 5% heat inactivated human AB serum supplemented by 300 IU/ml of IL-2 are added and centrifuged at 1000g for 10 minutes at 32^o^C. Plates are then incubated at 37^o^C overnight at 5% CO_2_. On the following day, after blocking and washing, 2 to 4 ml of thawed and 1:1 diluted MSGV1-A2aB-1G4A-LY3H10 retrovirus viral supernatant in TCR medium is applied to each retronectin-coated well of transduction #2 plates and centrifuged at 2000g for 2 hours at 32^o^C. Then, viral supernatant is removed from transduction #2 plates (leaving behind 1 ml). PBMC are transferred from transduction #1 plates to transduction #2 plates and centrifuged at 1000g for 10 minutes at 32^o^C. Plates are incubated at 37^o^C overnight at 5% CO_2_.

**For bags**, the 1:1 diluted MSGV1-A2aB-1G4A-LY3H10 retrovirus viral supernatant in TCR medium is applied to each bag of transduction #1 bags and kept at 4°C overnight with constant movement. Retroviral supernatant is removed from transduction #1 bag (leaving behind a few mls). A concentration of 0.5 x10^6^ cells/mL of activated PBMC in AIMV plus 5% heat inactivated human AB serum supplemented with 300 IU/ml of IL-2 in 200 mL are added to the bags. Bags are then incubated at 37^o^C overnight at 5% CO_2_. The bags are flipped to the other side to maximize the transduction efficiency after 2-3 hr of placement in the incubator. The same day as the first transduction, the 1:1 diluted MSGV1-A2aB-1G4A-LY3H10 retrovirus viral supernatant in TCR medium is applied to each bag of transduction #2 bags and kept at 4°C overnight with constant movement. On the following day, after blocking and washing, viral supernatant is removed from transduction #2 bags (leaving behind a few mls). PBMC are transferred from transduction #1 bags to transduction #2 bags. Then the bags are incubated at 37^o^C overnight at 5% CO_2_. The bags are flipped to the other side to maximize the transduction efficiency after 2-3 hr of placement in the incubator.

**Post-transduction Expansion.** At the end of the transduction, cells are washed and maintained at 37^o^C, 5% CO_2_ in AIMV plus 5% heat inactivated human AB serum supplemented by 300 IU/ml of IL-2 at a density of approximately 0.7-1 x 10^6^ cells/ml for up to 96 hours from the initial transduction.

### Transgenic PBMC Product Washing and Bagging

After completion of the cell transduction procedure and short term *ex vivo* expansion, the NY-ESO-1 TCR transduced PBMC product is subjected to washings, resuspended in saline containing 1% HSA, put into an infusion bag (or kept in the bag transduced in) and kept in a 4°C refrigerator until lot release clearance and i.v. infusion. Lot release tests are performed on aliquots of this final product. In the event that the patient is not able to receive the infusion within 24 hours of the scheduled harvest, cells will be re-suspended in cryopreservation medium and put into an infusion bag. Cryopreservation will be at < -130^o^C in a monitored liquid nitrogen freezer at the GMP suite.

### Transgenic PBMC Product Labeling

The final NY-ESO-1 TCR transgenic PBMC will be labeled using a preprinted study label with the following information:

- Patient UCLA identification number.
- Patient’s initials.
- Subject laboratory clinical trial code.
- Date of cell preparation, with an expiration date of 24 hours.
- Initials of person who prepared the vaccine.
- Labeled with “For autologous use only”.
- Labeled as “Not Tested for Biohazards” (due to no specific testing for HIV, HepC, HepB and other adventitious viruses in the final product).

### Transgenic PBMC in-process Testing and Final Product Lot Release Testing

**In Process Testing.** We will conduct the following in-process testing (Table 6).

Table 6. Transgenic PBMC In-process Testing

**Table 6a.** Activated PBMC (Day 2) In-process Testing:

| **Test Item** | **Acceptability Criteria** | Results |
| --- | --- | --- |
| Gram Stain | Negative | Negative |
| Bacterial Culture | No growth | No growth x 14 days |
| Fungal Culture | No growth | No growth x 14 days |

**Table 6b.** Transduced PBMC (Day 5) In-process Testing:

| Test Item | **Acceptable Criteria** |
| --- | --- |
| MycoAlert test | Negative; ratio < 1 (reading B/reading A) |
| PCR/ELISA mycoplasma assay if the MycoAlert results are equivocal or positive | Negative by PCR |
| TCR transgene expression | >10% of T cells by NY-ESO-1_126-157_ tetramer or dextramer staining |
| TCR transgene functionality | > 600 pg/ml/million cells of NY-ESO-1 specific IFN-γ production by ELISA |

**Final Product Testing and Lot Release Criteria.** In Table 7a, the results that will be required before administering the cells to the patients are described, and in Table 7b, the results that need to be in process at that time but results may not be available at the time of administration to patients are described.

Table 7. Transgenic PBMC Lot Release Testing

**Tab****le 7a.** NY-ESO-1 TCR Transgenic PBMC Lot Release Testing with Results Available Before Administration to Patients:

| Test Item | **Acceptable Criteria** |
| --- | --- |
| Viability | > 70% |
| Gram stain^a,d^ | Negative |
| Endotoxin Assay ^b,d^ | ≤ 5 EU/ kg body weight per dose |
| Mycoplasma rapid test^c,d^ | Negative; ratio < 1 (readingB/readingA) |
| TCR transgene expression | >10% of T cells by NY-ESO-1_126-157_ tetramer or dextramer staining |

a. Performed on the final product by the UCLA Clinical Microbiology Laboratory. All sterility cultures will be followed up to 14 days. If the cultures become positive after the patient has received the cells, the patient will be started on empiric antibiotics.

b. Performed on the final product using the Endosafe®PTS system (Charles River).

c. We will perform PCR/ELISA mycoplasma assay if the MycoAlert results are equivocal or positive. The release specifications for the PCR mycoplasma test should be "negative by PCR".

d. To avoid false positive results, if the test results are positive, it will be repeated with a second sample from the final product.

**Plan in Case of Sub-Optimal TCR Transgene Expression.** The following will be our plan in case of results different from our proposed acceptable criteria:

- - Any sub-optimally transduced product can also be administered at the discretion of the treating physician team.

**Table 7b.** NY-ESO-1 TCR Transgenic PBMC Lot Release Testing with Results Available After Administration to Patients:

| Test Item | **Acceptable Criteria** |
| --- | --- |
| Bacterial culture^a^ | No growth x 14 days |
| Fungal culture^a^ | No growth x 14 days |
| GalV S^+^  /L^-^ RCR assay | Sample archived for later use if required. Not tested prospectively in all samples. |
| TCR transgene functionality | > 600 pg/ml/million cells of NY-ESO-1 specific IFN-γ production by ELISA |

a. To avoid false positive results, if the growth is observed, the culture will be repeated with a second sample from the final product.

**Plan in Case of Positive Results of Tests Not Available at the Time of TCR Transgenic Cell Administration to Patients.** The following will be our plan in case of results different from our proposed acceptable criteria:

- - Bacterial or fungal culture: An antibiogram will be obtained on the culture to typify the contaminant. The patient will be contacted, two peripheral blood draws will be collected for culture, and the patient will be started on therapy, first with broad spectrum antibiotics or antifungals, and then adapted to the antibiogram of the cultures.
  - RCR: Since the TCR transgenic cell manufacture process will include *ex vivo* culture for up to 4 days from the first transduction, RCR will not be tested prospectively in the final product following the Guidance for Industry document by the Center for Biologics Evaluation and Research (CBER), FDA from November 2006. Samples for storage for potential testing for RCR will be obtained from peripheral blood cells at 3, 6 and 12 months. Thereafter, samples will be collected annually and stored depending on the course of the patient. If a sample from a patient is positive for RCR by PCR test, the stored sample of the final product will be tested. The patient will be contacted, two peripheral blood draws will be collected for confirmation, and the patient will be started on therapy with combination antiretrovirals used to treat HIV infection.

**Notification of Regulatory Agencies for Positive Results of TCR Transgenic Cell Administration to Patients Or Sub-Optimal TCR Transgene Expression.** If a preparation with bacterial, fungal, mycoplasma or RCR contamination, or one with sub-optimal TCR transgene expression has been administered to a patient, this event should be reported to the following agencies and committees within 48 hours of first knowledge:

- - Human Gene and Cell Therapy Facility Director
  - UCLA IRB.
  - UCLA Internal Scientific Peer Review Committee (ISPRC)/ Data Safety Monitoring Board (DSMB).
  - UCLA Institutional Biosafety Committee (IBC).
  - Recombinant Advisory Committee (RAC).
  - FDA.

**Additional Optional Testing of TCR Transgenic Cells.** The release tests will be used to determine whether the final cell product can be released for infusion (see Tables above). In addition, the cell product may undergo further characterization apart from the release testing. The vector-specific proviral copy number per cell may be determined. If higher than 5, we will propose a new dilution of the vector supernatant before using it for cell transduction. The sites of integration may be defined as the structure of the integrated transgene. Detailed flow cytometry and TCR chain usage analysis may be performed. The product may be further characterized for measurable transgene products (Table 8).

Table 8. NY-ESO-1 TCR Transgenic PBMC Optional Additional Testing

| Characterization Assays | Comments |
| --- | --- |
| Vector proviral copy per transduced cell | Quantification of the copy number of provirus by qRT-PCR |
| Final product phenotyping | CD3, CD4, CD8, CD27, CD28, CD45RA/RO, CD62L, CCR7, PD-1, Tim-3, CD57, CD95, TIGIT, LAG3, CCR6 |
| Transgene integration site analysis | Determination of integration site(s) |
| Structure of integrated transgene | Stability of integrated transgene |
| Detailed flow cytometry | Phenotype of cells |
| Other characterizations | Telomere loss, telomerase expression, Th1 vs Th2 pattern, T regulatory and Myeloid suppressor cell expression in pre/post expansion T cells |

## Generation of NY-ESO-1 TCR/sr39TK Transduced CD34+ cells

### CD34+ Cell Mobilization Procedure and Leukapheresis

Subjects will receive (or self-administer) 8-10 µg/kg/day metHuG-CSF (Neupogen® or other biosimilar) subcutaneously on “mobilization days” (mDay) 1-4 in the morning at approximately the same time every day. To enhance mobilization of the CD34+ cells, plerixafor, will be administered (0.24 mg/kg/day) subcutaneously daily beginning on the evening of mDay 4. Plerixafor will be administered in the clinic. Plerixafor should be administered in the evening prior to initiation of leukapheresis in the morning on mDay 5. Daily administration of G-CSF in the morning and plerixafor in the evening and daily leukaphereses will continue until a sufficient number of cells for backup and for transduction are collected up to mDay 8.

At least 2.0 x10^6^ CD34+ un-manipulated cells/kg (without CD34+ cell isolation) must be obtained for backup. Backup cells will be cryopreserved in liquid nitrogen vapors (≤ - 140°C) in the UCLA Bone Marrow/Stem Cell Transplant Laboratory according to the their SOP for possible future use if there is lack of engraftment ([Figure 2](#Figure2)).

Mobilized cells will be collected for transduction over a maximum of 3 days up to mDay 8. Since CD34+ cells can only be stored for 24 hours at room temperature or up to 48 hours at 4°C prior to transduction, the patient’s participation in the trial will be terminated if a sufficient number of cells are not obtained within the 3 day period. At least ≥2.5 x 10^6^ CD34-enriched cells/kg have to be obtained for transduction with LV-optNYESOTCR/TK lentiviral vector to obtain the required final number of transduced cells.

If a sufficient number of cells are obtained, leukapheresis collections over a maximum of 3 days will be pooled and CD34+ PBSC will be isolated using the Miltenyi CliniMACS system following the manufacturer’s instructions. The purified CD34+ cell fraction will be characterized for total cell number, viability, % of CD34+ cells and CFU-GM content by CFU assay in methylcellulose. If a sufficient number of cells are obtained (≥2.5×10^6^ cells/kg), the CD34-enriched cells will be resuspended in serum-free medium, supplemented with SCF/FLT3L/TPO and IL-3 at a density of at least 1x10^6^/ml. The cells will be cultured for 18±6 hours prior to transduction with clinical grade LV-optNYESOTCR/TK vector. LV-optNYESOTCR/TK transduced CD34+ cells will be cryopreserved until infusion on Day 0. Lot release testing will be performed prior to Day 0. If the transduction efficiency is > 50%, un-manipulated PBSC can be used to dilute the transduced cells to < 50% or the cells will not be administered.

### Distribution of the CD34+ PBSCs obtained by Leukaphereses

The following is the general plan for the use of cells obtained from the daily mobilized leukapheresis products:

- - ≥2.0 x 10^6^ un-modified cells/kg: Used for cryopreservation of backup cells in case of delayed hematopoietic engraftment or bone marrow failure. These cells will be processed following the standard protocols of the UCLA Bone Marrow Clinical Laboratory.
  - ≥2.5 x 10^6^ CD34-enriched cells/kg (over a maximum of 3 days): Used for TCR engineered PBSC manufacture in the GMP laboratory.
  - If transduction efficiency is > 50%, un-manipulated PBSC may be collected for dilution to < 50% transduced cells. These cells will be processed following the standard protocols of the UCLA Bone Marrow Clinical Laboratory.

### CD34+ Cell Isolation

Following leukapheresis, the CD34+ cell population will be enriched using the CliniMACS®CD34 reagent system, which consists of CliniMACS® Plus Instrument, CliniMACS® single use, sterile, disposable tubing set and CliniMACS® PBS/EDTA buffer following a manufacturing standard operating procedure (SOP) developed based on the manufacturer's instructions. The resulting cell fraction will be characterized for total cell number, viability, percentage of CD34+ cells and CFU potential.

### CD34+ Cell Activation.

Purified CD34+ cells will be resuspended in serum-free medium, supplemented cytokines rhSCF (50ng/mL), rhFLT3L (50ng/mL), rhTPO (50ng/mL) and rhIL-3 (20 ng/mL) at a density of at least 1.0 x10^6^/mL. All cytokines and culture reagents are approved for *ex vivo* clinical use. A small sample will also be removed for sterility testing. The cells will be cultured for 18±6 hours prior to transduction with clinical grade LV-optNYESOTCR/TK vector.

### Clinical Grade Lentivirus LV-optNYESOTCR/TK Lot Release Testing

Table 9a. Clinical Grade Lentivirus LV-optNYESOTCR/TK Lot Release Criteria (Lot #040417L1)

| TEST | METHOD | ACCEPTABILITY CRITERIA | Results | Date |
| --- | --- | --- | --- | --- |
| Vector insert | Southern Blot analysis | Vector size consistent with predicted fragment size | Vector size consistent with predicted fragment size | 1/11/2018 |
| Physical viral vector titer | P24 ELISA | Report Value | 6.6 x 10^7^ pg/mL | 1/11/2018 |
| Infectious titer | Assay using HT29 cells | ≥2.0 x 10^7^ ifu/mL | 3.88 x 10^8^ ifu/mL | 7/17/2017 |
| Sterility | Aerobic and anaerobic culture for bacteria and fungus | No growth within 14 days | No growth within 14 days | 11/3/2017 |
| Mycoplasma | Cuture and vero indicator cells | Negative | Negative | 12/1/2017 |
| *In vitro* viral assay | Assay on MRC-5, vero and A549 cells | No CPE or hemadsorption | No CPE or hemadsorption | 1/17/2018 |
| Replication competent lentivirus testing (RCL) | Co-culture of end production cells with C8166 cells with amplification and indicator phases  Supernatant testing on C8166 cells with amplification and indicator phases | No evidence of RCL | No evidence of RCL | 1/11/2018 |
| Endotoxin | Limulus amebocyte lysate | <100 EU/ml each sample | >0.3 and < 0.6 EU/ml | 1/11/2018 |
| Sterility Method Suitability | Bacteriostasis/Fungistasis | Pas | Pass | 11/3/2017 |
| Residual total DNA | Quantitative PCR | Report result | 4.17x10^4^ pg DNA/100 μL test article extracted | 11/14/2017’ |
| Residual benzonase | ELISA | Report result | 1.602 ng/ml at a 1:1.11 dilution and below limit of detection (0.195 ng/ml) at a 1:10 dilution | 10/19/2017 |
| Transfer of residual E1A^a^ | qPCR | Negative | <10 copies/0.2μg DNA^b^ | 1/11/2018 |
| Transfer of residual SV40^a^ | qPCR | Negative | <10 copies/0.2μg DNA^b^ | 1/11/2018 |

^a^On day 21 post transduction

^b^The limit of detection for this assay is 10 copies/0.2μg DNA. A result of <10 copies/0.2 μg DNA is considered negative.

### Clinical Grade LV-optNYESOTCR/TK Annual Recertification

Single-use aliquots of the clinical grade LV-optNYESOTCR/TK vector will be subjected to annual recertification, and the results of this testing will be included in the annual progress report for the FDA (Table 10).

Table 10. LV-optNYESOTCR/TK Annual Recertification

**Table 10a.** LV-optNYESOTCR/TK Annual Recertification Criteria

| Test Item | **Method** | **Limit of Detection** | **Acceptability Criteria** |
| --- | --- | --- | --- |
| Physical viral vector titer | P24 Elisa | 5 x 10^3^ copies/µ**L** | Report value |
| Functional viral titer | HT-29 transduction followed by ddPCR | 50 TU/ml ([Ruelle et al., 2014](#_ENREF_95)) | Report value |

ddPCR- Droplet Digital PCR

Table 10b. LV-optNYESOTCR/TK Lentivirus Annual Recertification 2019 (Lot #040417L1)

| Test | Method | Limit of Detection | Results | Date |
| --- | --- | --- | --- | --- |
| Physical viral vector titer | P24 ELISA | 5 x 10^3^ copies/µL | 5.1 x 10^7^ pg/mL | 3/26/2019 |
| Functional viral titer | HT29 transduction followed by ddPRC | 50 TU/ml | 2.67 x 10^8^ TU/mL | 3/21/2019 |

ddPCR = Droplet Digital PCR

### LV-optNYESOTCR/TK Transduction Method

LV-optNYESOTCR/TK vector will be stored in a monitored and locked ≤ -70ºC freezer in the UCLA GMP Suite, 14^th^ floor, Factor Building (Viral Bank). Transductions will be performed in T75/ T175 flasks or cell culture bags with LentiBOOST.

**Pre-stimulation:** The cells will be plated at a concentration of 1x10^6^ cells/ml in flasks/bags, pre-stimulated for 18 ± 6 hours (~37° C, 5% CO2) in serum-free X-VIVO15 medium and supplemented with the following cocktail of cytokines: rhSCF (50ng/ml), Flt-3 ligand (50ng/ml), TPO (50 ng/ml) and IL-3 20ng/ml. In process sterility samples will be collected and submitted for analysis.

Lentiviral transduction: After 18±6 hours of pre-stimulation, non-adherent cells will be removed from the culture flasks and spun down. An aliquot of supernatant will be collected and submitted for sterility testing. The non-adherent cells will be re-suspended in fresh cytokine-containing medium and added back to the original flasks containing the adherent cell fraction. The LV-NYESO sr39TK vector will be added at a final multiplicity of infection (MOI) of 15 (TU/cell). LentiBOOST stock will be added for a final 1x concentration. The transduction mixture of cells and virus will be rocked to ensure distribution of LentiBOOST and lentivirus. The total transduction period is 18 hours ± 6 hours at ~37° C, 5% CO2.

### LV-NYESO TCR/sr39TK PBSC (LV-optNYESOTCR/TK Transduced CD34+) Cell Formulation and Packaging

The cells will be washed with wash buffer (HBSS + 1% HSA) and then resuspended and formulated in cryopreservation solution, CryoStor® CS5. The formulated cell suspension will be filled into cryobags. The final formulation volume will account for the QC tests, RCL (1% of the final product) and reserve sample/s that will be collected from the cell product container immediately following the fill procedure and cryopreserved separately in sterile cryovials. Cell product, RCL, reserve and QC samples will be cryopreserved using a controlled-rate freezer at the same time under the same conditions. Upon completion of the controlled freeze, the cell product cryobags and QC samples will be transferred to storage in the vapor phase of LN2. Cryobags with cell product will be stored in the vapor phase of LN2 until released and required for administration into the subject. The cryopreserved QC sample will be thawed and subjected to lot release testing.

### LV-NYESO TCR/sr39TK PBSC (LV-optNYESOTCR/TK Transduced CD34+) Labeling and storage

The final product with LV-NYESO TCR/sr39TK PBSC (LV-optNYESOTCR/TK transduced CD34+ cells) will be labeled using a preprinted study label with the following information:

- Title line specifying cell product name
- Patient Identification Number (PIN)
- Medical Record Number
- Date and Time of Processing
- Number of Cells and volume of the cell product formulation
- Initials of Laboratory personnel
- Caution: New Drug – Limited by Federal Law for Investigational Use
- Product type and excipients
- For Autologous Use Only. Not Evaluated for Infectious Substances. Properly identify intended recipient and component or unit.
- Warning: This product may transmit infectious agents.
- Do not irradiate
- A Biohazard symbol

The product will be stored in the vapor phase of a centrally monitored liquid nitrogen freezer until ready to use.

### LV-NYESO TCR/sr39TK PBSC (LV-optNYESOTCR/TK Transduced CD34+ Cells) In-process Testing and Final Product Lot Release Testing

Samples are collected before, during, and at the end of the procedure for cell viability (trypan blue stain or AO/DAPI stain ), cell count, sterility (for bacteria, fungi, and mycoplasma) and intracellular transgene expression by flow cytometry, VCN analysis and percentage of LV-NY-ESO-TCR/TK positive CFUs. Sample for RCL testing will be banked and tested only if warranted (i.e. if follow-up patient samples are positive for RCL).

**In Process Testing.** We will conduct the following in-process tests (Table 11).

Table 4. Transduced CD34+ Cells In-process Testing

**Table 11a.** In-process Testing: Mobilized leukapheresis product

| **Test Item** | **Acceptability Criteria** | Results |
| --- | --- | --- |
| CD34+ enumeration (pooled) | ≥2.0×10^6^cells/kg | Assessed cell number |
| CBC | N/A | CBC results |

**Table 11b.** Post-CliniMACS separation (Day 0) In-process Testing:

| **Test Item** | **Acceptability Criteria** | Results |
| --- | --- | --- |
| Bacterial Culture | Negative at the day of cryopreservation | No growth |
| CD34+ enumeration | ≥2.5×10^6^ cells/kg | Assessed cell number |
| CFU assay | Report results | Assessed CFU number |

**Table 11C.** 18±6h Post-stimulation (Day 1) and 18±6h post transduction and prior to cryopreservation (Day 2) In-process Testing:

| **Test Item** | **Acceptability Criteria** | Results |
| --- | --- | --- |
| 18±6h Post-stimulation (Day 1) | | |
| Bacterial Culture | Negative at the day of cryopreservation | No growth |
| 18±6h Post transduction (Day 2) | | |
| CD34+ enumeration | ≥2.5×10^6^ cells/kg | Assessed cell number |
| Bacterial Culture | Negative at the day of cryopreservation | No growth |

In Table 12a, Final lot release criteria that will be required before administering the cells to the patients are described.

Table 12. LV-NYESO TCR/sr39TK PBSC (LV-optNYESOTCR/TK-transduced CD34+ Cells):

Final Product Lot Release Criteria

**Table 12a.** LV-optNYESOTCR/TK-transduced CD34+ Cells: Final Product Lot Release Criteria (Results Available Before Administration to Patients)

| **Test** | **Method** | **Acceptance Criteria** |
| --- | --- | --- |
| CD34 enumeration | ISHAGE  flow cytometric method | ≥2.0 - 6.0 ×10^6^ CD34+cells/kg |
| % CD34 purity | ISHAGE  flow cytometric method | ≥50% |
| Cell viability | Trypan Blue manual count or AO/DAPI Nucleocounter NC200 automated count | ≥70% |
| Vector Copy Number | ddPCR | 0.1-2 copies/cell |
| Sterility^b^ | Sterility culture | Negative |
|  | Fungal culture | Negative |
| Endotoxin | Endosafe®PTS | ≤ 5 EU/Kg |
| Mycoplasma^a^ | MycoAlert test | Ratio<1 |
| CFU assay | CFU potential Methylcellulose | ≥10% |
| % LV-NYESO+ CFUs | ddPCR | 5%-50% LV-NYESO+ colonies* |
| Vβ13.1^#^ expression | Flow Cytometry | 5%-50% of Vb13.1+ cells* |

*In order to make sure that the repertoire of endogenous TCRs will not be compromised, at least 50% of the CD34+ PBSC delivered to the patient will be untransduced.

1. We will perform a PCR/ELISA mycoplasma assay if the MycoAlert results are equivocal or positive.
2. To avoid false positive results, if the test results are positive, it will be repeated with a second sample from the final product.

**Table 12b.** LV-optNYESOTCR/TK-transduced CD34+ Cells: Final Product Lot Release Criteria (Results Available After Administration to Patients)

| **Test Item** | **Acceptable Criteria** |
| --- | --- |
| GALV S+/L- RCL assay | Sample archived for later use if required. Not tested prospectively in all samples. |

**Plan in Case of Positive Results of Tests Not Available at the Time of TCR Transgenic Cell Administration to Patients.** The following will be our plan in case of results different from our proposed acceptable criteria:

RCL: Since LV-optNYESOTCR/TK transgenic CD34+ cell manufacture process will not exceed 4 day *ex vivo* post-transduction expansion, RCL will not be tested prospectively in the final product following the current FDA recommendations for Testing of Replication Competent Retrovirus (RCR)/Lentivirus (RCL) in Retroviral and Lentiviral Vector Based Gene Therapy Products from November 19, 2010. If a sample from a patient tested at 3, 6 or 12 months or later is positive for RCR by PCR test, the stored sample of the final product will be tested. The patient will be contacted, two peripheral blood draws will be collected for confirmation, and the patient will be started on therapy with combination antiretrovirals used to treat HIV infection.

**Additional Optional Testing of TCR Transgenic CD34+ Cells.** The release tests will be used to determine whether the final cell product can be released for infusion (see Tables above). In addition, the cell product may undergo further characterization apart from the release testing. The vector-specific proviral copy number per cell may be determined. If higher than 2, we will propose a new dilution of the vector supernatant before using it for cell transduction. The sites of integration may be defined as the structure of the integrated transgene. Detailed flow cytometry and TCR chain usage analysis may be performed. The product may be further characterized for measurable transgene products (Table 13).

Table 13. LV-NYESO TCR/sr39TK PBSC (NY-ESO-1 TCR/sr39TK Transgenic CD34+ Cell)

Optional Additional Testing

| **Characterization Assays** | **Comments** |
| --- | --- |
| Vector proviral copy per transduced cell | Quantification of the copy number of provirus by qRT-PCR |
| Phenotyping of PBMCs | General Leukocyte panel:  CD56, CD16, CD45, CD14, CD15, CD38, ,CD33, CD117, CD19, CD20, CD3, HLA-DR, CD123, , CD11c, CD4, CD127.  T cell panel:  CD45, CD3, CD4, CD8, CD27, CD28, CD45RA/RO,  CD62L, CCR7, PD-1, Tim-3, CD95, CD57, LAG-3, TIGIT. |
| Transgene integration site analysis | Determination of integration site(s) |
| Structure of integrated transgene | Stability of integrated transgene |
| Other characterizations | Telomere loss, telomerase expression, Th1 vs Th2 pattern, T regulatory and Myeloid suppressor cell expression in pre/post expansion T cells |
| In-vitro TCR transgene functionality | NY-ESO-1 TCR/sr39TK Transgenic CD34+ will be differentiated into mature T cells using artificial thymic organoid culture (ATOC). Target cell induced activation of NY-ESO-1 TCR/sr39TK positive T cells will be assessed by IFN-gamma release. |

# EVALUATION OF STUDY ENDPOINTS

## Primary Study Endpoint

### Safety and Definition of Dose Limiting Toxicity (DLT)

**Criteria for safety evaluation.** Safety will be assessed by monitoring and recording potential adverse effects of the treatment using the Common Toxicity Criteria at each study visit. Subjects will be monitored by medical histories, physical examinations and blood studies to detect potential toxicities from the treatment.

**Protocol safety observation period.** The period of safety evaluation to define a DLT is 90 days from the day of TCR transgenic cell infusion (Day 0).

**DLTs and Adverse Events (AEs) developing after the safety observation period.** Late developing protocol-related adverse events, beyond the 90-day DLT observation study period, will be captured in the study CRFs and will be reported to the FDA in a timely manner depending on their severity. If they qualify as serious adverse events (SAEs), then they will be reported immediately. Adverse reactions will be reported to the FDA according to 21 CFR 312.32. Prolonged cytopenia, infections, and autoimmune AEs will be indicated as AEs of special interest, and will be notated as such in CRFs and in all real-time reporting.

**Substitution of patients based on voluntary withdrawal.** If a patient withdraws early from participation voluntarily without a DLT or due to disease progression before the first 60 days of the DLT observation period, this patient will be substituted to allow adequate assessment of safety.

**Timing of enrollment.** There will be a staggered enrollment of subjects as follows:

- All enrollees will start on study with a minimum of 1 week between Day 0 (gene-modified stem cell administration) of a subject and Day -5 (unmobilized leukapheresis for collection of cells for gene-modified T cell manufacture) of the next subject
- There will be at least 3 months between Day 0 for the first subject and enrollment of the second subject.

#### Definition of Dose Limiting Toxicities (DLT)

- After the first 3 subjects are dosed, the cohort will be expanded to 6 subjects.
- If less than 2/6 Dose Limiting Toxicities (DLTs) are observed, the treatment will be considered safe.
- If DLTs are observed in 2 or more of 6 subjects, then the treatment will have exceeded the 33% DLT rate, and the study will be terminated.

Adverse events are defined following NCI Common Terminology Criteria for Adverse Events (CTCAE) v5.0. DLTs are defined as:

- Grade 2 or greater allergic reaction/hypersensitivity (except for fever or rash) at least possibly related to infusion.
- Grade 3 or greater toxicity, with specified exceptions, which is at least possibly related to the infusion of genetically modified NY ESO-1 TCR engineered PBMC or PBSC.
- Grades ≥ 2 autoimmune toxicity at least possibly related to infusion.
- Any Grade of skin rashes related to Toxic Epidermal Necrolysis/Steven Johnson’s Syndrome
- Grade 3 or greater skin ulceration and skin rashes with desquamation.
- Grade 3 or greater toxicity due to cytokines that does not decrease to ≤ Grade 1 in 72 hours.
- Intubation due to respiratory distress that is related to the TCR engineered ACT therapy (except IL-2 induced capillary leakage).
- Grade 3 or higher toxicity during IL-2 administration not reversible with medicines or holding IL-2 within 24 hours.
- Grade 2 or greater bronchospasm requires discontinuation T-cell infusion.
- Engraftment failure or delayed engraftment (ANC < 500/µL at +28 days).
- Any adverse event that leads to a discontinuation of T-cell infusion.
- Administration of a contaminated preparation of NY-ESO-1 TCR engineered cells.

The known toxicities and side effects of the chemotherapy preparative regimen, as listed in the protocol or package insert, or IL-2) or G-CSF cytokine administration adverse events, as listed in the protocol or package insert, will not be considered for the assessment of DLTs. Furthermore, prolonged cytopenia, infections, and autoimmune AEs will be indicated as AEs of special interest, and will be notated as such in CRFs and in all real-time reporting.

## Secondary Study Endpoints:

### Feasibility

After enrolling 3 patients, followed up for a minimum of 1 month after the last subject has received the infusion of the NY-ESO-1 TCR transgenic cells, an assessment of protocol feasibility will be done by the study investigators. The feasibility assessment will be based on potential problems in the manufacturing of NY-ESO-1 TCR engineered T cells and stem cells [(Table 14).](#Table_13_Criteria_Study_Unfeasible)

Table 14. Criteria to Declare the Study Unfeasible

| **Parameter** | **Criteria for Unfeasibility** |
| --- | --- |
| Feasibility of generation NY-ESO-1 TCR transgenic T cells that meet the lot release criteria. | 2 or more preparations not meeting the lot release criteria. |
| Feasibility of generating NY-ESO-1 TCR/sr39TK transgenic stem cells that meet the lot release criteria. | 2 or more preparations not meeting the lot release criteria. |

### Transgenic Cell Persistence.

Engraftment and persistence of transduced T cells and progeny T cells will be assessed in PBMC samples obtained before adoptive transfer and at 14, 30, 60, 90 and 120 days after transgenic cell adoptive transfer. Thereafter, if possible, sampling will be every 3 months during the first 2 years, and then every 6 to 12 months. Analysis will be performed both using immune monitoring and molecular techniques. Detection of surface expression of the NY-ESO-1 TCR transgenic protein will be analyzed both by MHC dextramer analysis and staining for the specific region. Molecular analysis of the engraftment and persistence of transduced T cells and progeny T cells bearing the NY-ESO-1 TCR transgene will be assessed via ddPCR using retroviral and lentiviral element primers to quantitate the vector copy number, respectively. This testing will provide data to estimate the *in vivo* survival of lymphocytes derived from the infused cells.

#### Long Term Monitoring for Replication Competent Retrovirus (RCR) and

#### Replication Competent Lentivirus (RCL).

Analysis for detection of RCR and RCL by PCR will be performed at the IUVPF under the supervision of Dr. Ken Cornetta. Samples taken from cells prior to infusion will be archived and blood samples obtained from study patients at 3, 6 and 12 months post cell administration will be tested. Blood samples will be archived annually thereafter if all previous testing has been negative.

If a patient dies or develops a neoplasm (other than recurrence of the original malignancy) during this clinical trial, efforts will be made to assay a biopsy sample for RCR and RCL. If any post-treatment samples are positive, then the baseline final product will be tested for RCR and RCL. Further analysis of the RCR and RCL and more extensive patient follow-up will be undertaken, in consultation with the UCLA IRB, RAC and FDA.

#### Analysis of Retroviral and Lentiviral Insertion Sites in Long Term

#### Persisting NY-ESO-1 TCR Clones

It is possible that expansion of specific T-cell clones will be observed as tumor reactive T cells proliferate in response to tumor antigens. Therefore, care will be taken to track T cell persistence both immunologically and molecularly. Blood samples for persistence of TCR gene transduced cells will be obtained at 3, 6 and 12 months, and then annually thereafter. If any patient shows a high level of persistence of TCR gene transduced cells at month 6 (by semi quantitative DNA-PCR using primers specific for vector sequences) the previously archived samples will be subjected to techniques that would allow the identification of clonality of persisting TCR gene transduced cells. Such techniques may include T cell cloning or LAM-PCR. Clonality analysis is available through the IUVPF ([Patterson, 2005](#_ENREF_80)).

If a predominant or monoclonal T cell clone derived from TCR gene transduced cells is identified during the follow-up, the integration site and sequence will be identified and subsequently analyzed against the human genome database to determine whether the sequences are associated with any known human cancers. If a predominant integration site is observed, the T cell cloning or LAM-PCR test will be used at an interval of no more than three months after the first observation to see if the clone persists or is transient. In all instances where monoclonality is persistent and particularly in instances where there is expansion of the clone, regardless of whether or not the sequence is known to be associated with a known human cancer, the subject should be monitored closely for signs of malignancy.

#### Immunological Monitoring.

Immunological monitoring will consist primarily of quantifying T cells bearing surface NY-ESO-1 TCR by NY-ESO-1_126-157_/MHC dextramer analysis. Functional assays like ELISA, intracellular cytokine staining (ICS), and/or multicytokine array assays will complement the results. Immunological assays will be compared between 1) pre-infusion PBMC and PBSC, 2) an aliquot of the engineered peripheral blood lymphocytes and stem cells at the time of infusion and 3) cells recovered from patients’ peripheral blood after adoptive transfer.

Our published definitions for a positive or negative immunological response using standardized MHC tetramer analyses will be used ([Comin-Anduix et al., 2006](#_ENREF_27)). These definitions are based on the assay performance specifications by defining changes that are beyond the assay variability with a 95% confidence level. For the other assays, differences of 1 fold will be considered indicative of true biologic differences.

Additional testing may be performed with other assays, for example intracellular phosphoprotein staining at the single cell level for signaling networks, multiplexed microfluidic and nanotechnology-based immune monitoring assays ([Kwong et al., 2009](#_ENREF_63)).

### Objective Response

#### Malignancy status

To assess clinical response all patients who have received an infusion of RV-NYESO TCR PBMC and LV-NYESO TCR/sr39TK PBSC and have tumor assessments at baseline and during the study follow up will be considered evaluable for tumor response.

Potential objective responses to this combinatorial immunotherapy will be recorded following RECIST v1.1 criteria ([Therasse et al., 2000](#_ENREF_108)). Appropriate evaluations, including physical exam, pictures of visible lesions and imaging exams, will be evaluated at screening and then Day 60, or at discontinuation of study. The duration of patient evaluation may be extended if additional outpatient visits are required to assess duration of tumor response or time to progression.

#### Definition of Measurable and Non-measurable Lesions

Measurements of all lesions should be recorded in metric units. All baseline evaluations must be performed as close as possible to the first day of study treatment. The same method of assessment and the same technique should be used to characterize each identified and reported lesion at baseline and during follow-up.

1. **Measurable Lesions:**

- Lesions that can be accurately measured in at least 1 dimension (longest diameter to be recorded) as ≥2.0 cm with conventional imaging techniques, ≥1.0 cm with spiral CT scan with slices of 5 mm thickness, or no less than double the slice thickness using modern CT scanners with thinner slices.
- Malignant lymph node: to be considered pathologically enlarged and measurable, a lymph node must be ≥15mm in the short axis when assessed by CT scan. At baseline and in follow up, only the short axis will be measured and followed.
- Skin nodules that can be clearly documented by color photography, including a ruler, to document the maximum diameter of the target lesion(s), regardless of their size.

1. **Non-measurable Lesions:**

- All other malignant lesions: Previously irradiated lesions are non-measurable except in cases of documented progression of the lesion since the completion of radiation therapy.

#### Definition of Target and Non-Target Lesions

Patients must have at least one measurable lesion at baseline to be included in the analysis of this endpoint. Baseline documentation of tumor sites may include imaging assessment of disease in the chest, abdomen and pelvis.

1. **Target Lesions:**

- Up to 10 total lesions, a maximum of 5 lesions per organ, that are representative of all involved organs may be selected and recorded as target lesions at baseline. Target lesions should be selected on the basis of their size (lesions with the longest diameter) and their suitability for accurate repetitive measurements (either by imaging techniques or clinically). The sum of the longest diameter for *all* *target lesions* will be calculated and reported as the baseline sum longest diameter. The baseline sum longest diameter will be used as reference to further characterize the objective tumor response of the disease.

1. **Non-Target Lesions:**

- All other lesions (or sites of disease) should be identified as non-target lesions and recorded as non-target lesions at baseline. Measurement of non-target lesions at baseline is not required and should be recorded as “present.” Each non-target lesion should be documented as either present, absent or new in each subsequent evaluation.

#### Objective Response Classifications

The following RECIST criteria will be the primary method utilized in this study for the assessment and reporting of tumor response data:

- Complete Response (CR): Disappearance of all evidence of tumor in target and non-target lesions. CR must be confirmed by repeat assessments performed no less than 4 weeks after the criteria for response are first met to qualify as CR. This definition includes a pathological CR when hyperpigmented lesions visible by exam are biopsied and demonstrate no viable measurable melanoma and pigment accumulated in phagocytic cells.
- Partial Response (PR): At least a 30% decrease in the sum of the longest diameter (LD) of target lesions taking as reference the baseline sum LD. Non-target lesions may persist provided there is no unequivocal progression in these lesions. PR must be confirmed by repeat assessments performed no less than 4 weeks after the criteria for response are first met to qualify as PR;
- Progressive Disease (PD): At least a 20% increase in the sum LD of the target lesions from the smallest sum LD recorded since the beginning of therapy or the appearance of one or more new lesions or unequivocal progression of existing non-target lesions; and
- Stable Disease (SD): Measurements demonstrating neither sufficient shrinkage to qualify for PR nor sufficient increase to qualify as PD after the start of treatment taking as reference the smallest sum LD since the treatment started. During this time, non‑target lesions may persist provided there is no unequivocal progression in these lesions.

#### Frequency of Tumor Measurements

Tumor assessments for response will be performed starting at the end of 2 months (Day 60) and every 2-3 months subsequently. Tumor assessment will also be performed at discontinuation from the study, unless these have been performed within the last 4 weeks prior to discontinuation. CR and PR must be confirmed by repeat assessments performed no less than 4 weeks after the criteria for response are first met to qualify as such response. Additional tumor measurements may be completed as needed.

#### Assessment of Response for In-Transit Metastasis

In patients with in-transit metastasis only, at least one in-transit metastatic lesion should not be excised. If there is only one measurable lesion, biopsies should not interfere with the measurement of the largest diameter of the lesion. Response will be assessed as above, taking the measurement from pictures with a build-in ruler.

#### Duration of Response

The duration of overall complete response will be measured from the time measurement criteria has been first met for CR until the first date that recurrent or progressive disease is objectively documented.

The duration of overall response will be measured from the time measurement criteria is met for CR/PR (whichever is first recorded) until the first date that recurrent or progressive disease is objectively documented (taking as reference for progressive disease the smallest measurements recorded since the treatment started).

#### Time to Disease Progression

Time to disease progression is defined as the length of time from the date of cell infusion (Day 0) to the date of progressive disease first documented, or death whichever occurs first. Subjects lost to follow-up will be censored at their last available tumor assessment.

## Exploratory study endpoints:

### Evaluation of LV-NYESO TCR/sr39TK PBSC Biodistribution

#### *In Vivo* Imaging with [^18^F] FHBG-PET

The ability to non-invasively image T cell responses to cancer hinges upon the detection of TCR transgenic cells with PET probes. The PBSCs in this study are transduced with a lentiviral vector carrying the sr39TK reporter gene. The sr39TK protein phosphorylates an experimental PET reporter probe, [^18^F] FHBG, upon i.v. administration. FHBG becomes trapped in the sr39TK-expressing PBSCs and progeny T cells upon phosphorylation and is detectable by PET imaging. The US Food and Drug Administration has previously approved its use as an investigational new drug in clinical trials (IND no. 61880) ([Yaghoubi and Gambhir, 2006](#_ENREF_119)). We are currently filing a new IND for [^18^F]FHBG to be used in this clinical trial.

Patients will be injected with 200 MBq [^18^F]FHBG approximately 25 days and 120 days after ACT, with allowed variability of ± 10 days and ± 30 days, respectively.. One-hour post injection, a PET/CT scan will be acquired. Scans of the extremities will be performed in patients with metastatic lesions in the arms or legs. The CT scan will be performed in a “low dose mode” (110 kVp, 30 mAs anode current) resulting in an effective dose of less than 1.3 mSv per scan ([Brix et al., 2005](#_ENREF_17); [Yaghoubi et al., 2001](#_ENREF_117)). These doses are comparable with typical diagnostic CT or PET scans and below the limits set by federal regulations for research studies involving radioactive imaging agents (http://www.access.gpo.gov/nara/cfr/waisidx_01/21cfr361_01.html).

Regional uptake of [^18^F]FHBG within metastatic tumor sites and secondary lymphoid organs will be quantified by SUV normalized to the body weight of the patient. As an internal quality control, SUVs will also be determined for several normal organs, such as muscle, liver and lungs. These measurements will allow us to identify technical problems in the SUV calculations, such as partially paravenous tracer administration ([Czernin et al., 2006](#_ENREF_29); [Weber, 2005](#_ENREF_114)). An attempt will be made to have all imaging studies performed on the same PET/CT system in order to eliminate confounding effects of differences in scanner sensitivity, spatial resolution or image processing.

### Evaluation of Delayed Clinical Response

**11.3.2.1 Evaluation of Clinical Response per iRECIST**

Immunotherapy and cellular therapy interventions against cancer can lead to unique response patterns, including durable stable disease, delayed response after initial tumor burden increase (flare response), and regression of a target lesion with appearance of new lesions. In order to classify these, we will also classify tumor responses per iRECIST ([Seymour et al., 2017](#_ENREF_103)). In brief, this will reset the classification if RECIST Progressive Disease (PD) is followed at next time point by tumor shrinkage, with new overall response defined as “iUPD” (immune unconfirmed progressive disease). iCPD (immune confirmed progressive disease) would then be confirmed by repeat imaging (PET/CT or CT, as appropriate and clinically feasible) 4-8 weeks later.

## Definition of Study Failure Due to Failure of Meeting the Study Endpoints.

Study failure according to safety and feasibility considerations is defined as follows:

1. Unexpected SAEs that are related to the transduction process or research reagent and required that the study be stopped.
2. 2 or more DLT.
3. Considered to be an unfeasible approach as defined by:

i. Failure to generate acceptable numbers of transduced PBMC or PBSC. One patient may be replaced for this criterion.

ii. Failure of generation of transduced PBMC or PBSC to meet lot release criteria. One patient may be replaced for this criterion.

iii. Requirement for therapeutic doses of systemic corticosteroid therapy during the trial. This will be a reason for DLT definition and patients will not be replaced.

iv. Need for treatment of newly identified brain metastases. This will not be a definition of DLT and patients will be withdrawn from the protocol due to disease progression.

v. Standard reasons of refusal, non-compliance, intercurrent significant illness or need for major surgery, patient request. In this case the following will be done:

- 1. One patient will be replaced if the study participation is stopped due to intercurrent significant illness or need for major surgery.
  2. Patients who stop participation due to patient request or for being non-compliant will be substituted to allow adequate number of patients.
  3. Every attempt will be made to continue to monitor all patients for short and long-term toxicities.

# ADVERSE EVENT REPORTING

## Definition of an Adverse Event (AE)

An adverse event (AE) is any untoward medical occurrence in a clinical investigation patient. An AE can therefore be any unfavorable and unintended sign (including an abnormal laboratory finding), symptom or disease temporarily associated with the use of a medicinal (investigational) product, whether or not related to the medicinal (investigational) product.

Adverse events encountered during the clinical study will be recorded on the Case Report Form (CRF). Adverse events should be collected beginning from the time the patient receives the first administration of drug therapy. The investigator must evaluate and document the adverse event for severity, grade it according to the NCI Common Toxicity Guideline, document the causal relationship to the study drug, take appropriate action to care for the patient and document the outcome. All information recorded in the case report form must be verifiably documented in the source documents (i.e., medical record or physician office chart).

The investigator is to record all solicited AEs and all AEs spontaneously reported by the patient. Adverse events in clinical investigation patients include any change from the patient’s baseline (pretreatment) condition. This includes symptoms, physical findings or clinical syndromes and encompasses the following:

1. Any suspected adverse medication reactions.
2. Any reactions from medication overdose, abuse, withdrawal, sensitivity, or toxicity.
3. Apparently unrelated illnesses, including the worsening of a pre-existing illness (see Pre-existing Conditions, below).
4. Injury or accidents. Note that if a medical condition is known to have caused the injury or accident (e.g., a fall secondary to dizziness), the medical condition (dizziness) and the accident (fall) should be reported as two separate adverse events. The outcome of the accident (e.g., hip fracture secondary to the fall) should be recorded under Comments.
5. Abnormalities in physiological testing or physical examination (findings that require clinical intervention or further investigation beyond ordering a repeat [confirmatory] test).
6. Laboratory abnormalities that require clinical intervention or further investigation (beyond ordering a repeat [confirmatory] test) unless they are associated with an already reported clinical event. Laboratory abnormalities associated with a clinical event (e.g., elevated liver enzymes in a patient with jaundice) should be described under Comments on the report of the clinical event rather than listed as a separate adverse event.
7. Prolonged cytopenia, infections, and autoimmune AEs will be indicated as AEs of special interest, and will be notated as such in CRFs and in all real-time reporting.

## Definition of a Serious Adverse Event (SAE)

An AE that meets one or more of the following criteria/outcomes is classified as serious:

- Results in **death**.
- Is **life-threatening**. Life-threatening means that the patient was at immediate risk of death from the reaction as it occurred, i.e., it does not include a reaction which hypothetically might have caused death had it occurred in a more severe form.
- Requires inpatient **hospitalization or prolongation of existing hospitalization**.
- Results in **persistent or significant disability/incapacity**. Disability is defined as a substantial disruption of a person’s ability to conduct normal life functions.
- Is a **congenital anomaly/birth defect**.
- Is an **important medical event**. An important medical event is an event that may not result in death, be life-threatening, or require hospitalization but may be considered an SAE when, based upon appropriate medical judgment, it may jeopardize the patient and may require medical or surgical intervention to prevent one of the outcomes listed in the definitions for SAEs. Examples of such medical events include allergic bronchospasm requiring intensive treatment in an emergency room or at home, blood dyscrasias or convulsions that do not result in inpatient hospitalization, or the development of drug dependency or drug abuse.

Suspected transmission of an infectious agent (e.g., any organism, virus or infectious particle, pathogenic or non-pathogenic) via the study drug is an SAE. Although pregnancy, overdose, and cancer are not always serious by regulatory definition, these events must be handled as SAEs.

Progression of the malignancy under trial should not be reported as an adverse event. However, if the malignancy has a fatal outcome during the trial or within the safety-reporting period, then disease progression must be recorded as a Grade 5 serious adverse event.

Medical and scientific judgment should be exercised in deciding whether expedited reporting is appropriate in other situations, such as important medical events that may not be immediately life-threatening or result in death or hospitalization, but may jeopardize the patient or may require intervention to prevent one of the other outcomes listed in the definition above. These events are to be considered serious and subject to reporting procedures specified below.

## Pre-existing Conditions

In this trial, a pre-existing condition (i.e., a disorder present prior to study start and noted on the pretreatment medical history/physical examination form) should not be reported as an AE unless the condition worsens or episodes increase in frequency during the AE reporting period.

Planned hospital admissions or surgical procedures for an illness or disease that was present before the patient received any study drug are not to be considered AE’s unless the condition deteriorated in an unexpected manner during the trial (e.g., surgery was performed earlier than planned).

## Procedures

Diagnostic and therapeutic non-invasive and invasive procedures, such as surgery, should not be reported as adverse events. However, the medical condition for which the procedure was performed should be reported if it meets the definition of an adverse event. For example, an acute appendicitis that begins during study should be reported as the AE, and the resulting appendectomy noted under comments.

## Assessing Severity of Adverse Events

AEs will be graded according to the NCI CTCAE (Version 4.0) (http://ctep.cancer.gov/forms/CTCAEv4.pdf). Those AEs not covered by these criteria will be graded on a five-point scale (mild, moderate, severe, life-threatening, death) and reported in the detail indicated on the CRF. The definitions are as follows:

- Grade 1 Mild; asymptomatic or mild symptoms; clinical or diagnostic observations only; intervention not indicated.
- Grade 2 Moderate; minimal, local or noninvasive intervention indicated; limiting age appropriate instrumental activities of daily living (ADL).
- Grade 3 Severe or medically significant but not immediately life-threatening; hospitalization or prolongation of hospitalization indicated; disabling; limiting self-care ADL.
- Grade 4 Life-threatening consequences; urgent intervention indicated.
- Grade 5 Death related to AE.

## Classification of Causality

NOT RELATED: A causal relationship between the study treatment and the adverse event is not a reasonable possibility.

RELATED: A causal relationship between the study treatment and the adverse event is a reasonable possibility. The investigator must further qualify the degree of certainty as “POSSIBLE” or “PROBABLE”.

Items to be considered when assessing the relationship of an adverse event to the study

treatment are:

- Temporal relationship of the onset of the event to the initiation of the study treatment.
- The course of the event, considering especially the effect of discontinuation of study treatment or reintroduction of study treatment, as applicable.
- Whether the event is known to be associated with the study treatment, or with other similar treatments.
- The presence of risk factors in the study patient known to increase the occurrence of the event.
- The presence of non-study treatment related factors that are known to be associated with the occurrence of the event.

## Seriousness and Non-Serious Adverse Events

Clarification should be made between the terms “serious” and “severe” since they ARE NOT synonymous. The term “severe” is often used to describe the intensity (severity) of a specific event (as in mild, moderate, or severe myocardial infarction); the event itself, however, may be of relatively minor medical significance (such as a severe headache). This is NOT the same as “serious,” which is based on patient/event outcome or action criteria described above and are usually associated with events that pose a threat to a patient’s life or functioning. A severe AE does not necessarily need to be considered serious. For example, persistent nausea of several hours duration may be considered severe nausea but not an SAE. On the other hand, a stroke resulting in only a minor degree of disability may be considered mild, but would be defined as an SAE based on the above noted criteria. Seriousness (not intensity) serves as a guide for defining regulatory reporting obligations.

## Reporting of AEs and SAEs

All AEs and SAEs will be reported to the FDA according to 21 CFR 312.32. All AEs spontaneously reported by the patient and/or in response to an open question from study personnel or revealed by observation, physical examination or other diagnostic procedures will be recorded on the appropriate page of the CRF. Any clinically relevant deterioration in laboratory assessments or other clinical findings is considered an AE and will be recorded on the appropriate pages of the CRF. When possible, signs and symptoms indicating a common underlying pathology should be noted as one comprehensive event.

AEs will be reported beginning at the time of the first study treatment administration on mDay 1 through 90 days after administration of the transgenic cells on Day 0. AEs will not be reported in the follow-up phase (after Day 90) unless they are deemed to be related to study drug. Any known untoward event that occurs subsequent to the AE reporting period that, in the investigator’s opinion, is possibly related to the investigational medication/product must also be reported as an adverse event.

All AEs that occur in trial patients during the AE reporting period specified in the protocol should be recorded in the CRF, irrespective of their assessed relationship to the study drug. Prolonged cytopenia, infections, and autoimmune AEs will be indicated as AEs of special interest, and will ne notated as such in CRFs and in all real-time reporting.

All AEs must be reported to the IRB, FDA and all other appropriate agencies (e.g., IBC and NIH for gene medicine trials.) The assessment and reporting must occur in writing in an annual report.

SAEs including patient deaths will be reported from informed consent through 90 days after administration of the transgenic cells on Day 0. ALL deaths, including any death that occurs during the follow-up period, must be reported whether or not considered causally related to the study drug. Any SAEs possibly related to study drug should be reported beyond 90 days after administration of the transgenic T cells and stem cells.

If the event is **Serious and Non-Life Threatening**, the event should be reported in writing within 10 working days by the Principal Investigator to the UCLA IRB, JCCC DSMB, and to all appropriate agencies (NIH/RAC, the UCLA IBC and the FDA).

If the event is **Fatal or Life Threatening**, the event should be reported within 1 working day to the UCLA IRB, JCCC DSMB, ISPRC, IBC and within 3 working days to the FDA and NIH/RAC. For institutional trials and for gene medicine trials, the JCCC ORC and Gene Medicine Compliance Officers will assist the investigator to ensure that all serious adverse events are properly documented and reported in accordance with federal and institutional requirements.

Events that meet the SAE definition will be reported within the following timelines based on the time of first knowledge of the SAE by a study investigator:

- SAE other than resulting in fatal or life threatening events:
  - Report within 10 working days
  - Report to PI, IRB, ISPRC, DSMB, IBC, NIH/RAC and FDA
- Fatal or life threatening events:
  - Report within 1 working days to PI, IRB, ISPRC, DSMB and IBC
  - Report within 3 working days to NIH/RAC and FDA

The study coordinator will work with the treating physician to fill out an FDA MedWatch Form and the forms required for each agency/committee for SAEs. All adverse events that occur in a research study overseen by the JCCC DSMB must be submitted to the DSMB, regardless of relationship, expectedness, or seriousness.

The SAE information will include a minimum of the following: patient number; a narrative description of the event; and an assessment by the investigator as to the intensity of the event and relatedness to study drug. SAEs reported should match the data provided on the CRF.

In general, any SAE will be reported as soon as it is known by the study investigators and enough data is gathered for report filing. A description of the SAE is generated and forms for each of the aforementioned committees are completed. The SAE forms specific to each committee and the SAE description is emailed to each committee or submitted through the appropriate web-based system (eg. webIRB and SafetyNet). Initial reports may be completed or amended with any new relevant additional information. Copies of all reports will be maintained in the study regulatory file.

## Follow-up of Serious Adverse Events

All SAEs should be monitored until they are resolved or are clearly determined to be due to a patient’s stable or chronic condition or intercurrent illness(es). The resolution and outcome of such events should be documented on the CRF.

## Withdrawal Due to Adverse Events

Withdrawal due to adverse event should be distinguished from withdrawal due to insufficient response, according to the definition of adverse event noted earlier, and recorded on the appropriate adverse event CRF page. When a patient withdraws due to a serious adverse event, the serious adverse event must be reported in accordance with the reporting requirements defined below.

# COMPLIANCE WITH GOOD CLINICAL PRACTICE, ETHICAL CONSIDERATIONS, INFORMED CONSENT

## Compliance with Good Clinical Practice and Ethical Considerations

The study will be conducted in accordance with applicable FDA regulations, the guidelines of ICH Good Clinical Practices (GCP) (CPMP/ICH/135/95), as well as the demands of national drug and data protection laws and other applicable regulatory requirements. Approval will be obtained from the appropriate regulatory authorities.

The Institutional Review Board (IRB) and Institutional Scientific Peer Review Committee (ISPRC) will review all appropriate study documentation in order to safeguard the rights, safety and well-being of the patients. The protocol, informed consent form, safety updates, annual progress reports, and any revisions to these documents will be provided to the IRB by the investigator.

## Patient Confidentiality

In order to maintain patient privacy, all CRFs, study drug accountability records, study reports and communications will identify the patient by initials where permitted and/or by the assigned patient number. The patient’s confidentiality will be maintained and will not be made publicly available to the extent permitted by the applicable laws and regulations.

## Regulatory Approvals

The Protocol and ICF must be approved by the following committees before initiation of patient enrollment:

Local Committees:

- Institutional Review Board (IRB).
- Institutional Biosafety Committee (IBC).
- Institutional Scientific Peer Review Committee (ISPRC).
- Data Safe Monitoring Board (DSMB).
- Medical Radiation Safety Committee (MRSC).

Federal Committees:

- Recombinant DNA Advisory Committee (RAC).
- Food and Drug Administration (FDA).

## Subject Recruitment

Study subjects will be invited to participate by the study investigators as part of the discussion of standard and experimental therapy for locally advanced or metastatic melanoma and for advanced malignancies for which no suitable standard of care exists. Patients may access the study investigators through:

1. Subjects may be referred from UCLA or outside clinics. No patient will be approached without a referral or agreement from the patient’s physician in order to uphold patient confidentiality.
2. Subjects may be identified by the study investigators from their own patient pools while discussing standard and experimental options for advanced or metastatic melanoma.
3. Study flyers will be posted in public places, including the oncology clinic waiting room, for subject self-referral.
4. Subjects may self-refer to this study when inquiring about clinical trial options at UCLA.
5. This study will be listed at the NIH web site listing active clinical trials, which also assures trial registration at a public site.

## Gender and Minorities

There will be no discrimination based upon sex or race. Subjects of any gender and ethnicity with an advanced malignancy have been and will be considered in the proposed clinical trials. There has been and will certainly be no discrimination due to gender or ethnicity, but the epidemiology of malignancy and HLA haplotypes may result in uneven minority population distribution.

Most subjects referred to the UCLA Division of Hematology and Oncology come from the UCLA Oncology Network, with over 50 offices throughout the Los Angeles County and Western US, including areas where there is a majority of subjects of Asian, Hispanic and African American descent. Cutaneous malignant melanoma occurs less frequently among non-white populations than among whites. Data from the California Cancer Registry reveal that, between 1988 and 1993, 95.5% of subjects with melanoma in California were non-Hispanic whites, 3.5% were Hispanic, 0.5% Asian, and 0.5% non-Hispanic blacks ([Cress and Holly, 1997](#_ENREF_28)).

## Children

We have reduced the lower age limit for this protocol to 10 years old to allow enrollment of younger age patients with one of the target histologies, synovial sarcoma. The use of the reduced intensity regimen in this protocol is a major procedure that entails serious discomforts and hazards for the patient, such that fatal complications are possible. It is therefore only appropriate to carry out this experimental procedure in the context of life threatening metastatic cancer. However, patients with age less than 16 will be considered on a case-by-case basis. Additionally, ovarian cancer and most solid tumor malignancies in which NY-ESO-1 is known to be expressed are rarely found in patients <16 years old. Since synovial sarcoma does occur in children; we will address whether these patients would potentially benefit from this trial on a case by case basis with our pediatric sarcoma team. Since May 21, 2020, three patients ≥ 18 years old have been treated successfully, and the treatment has been shown to be safe, reason why we considered that patients 10-17 years old should be enrolled in the trial. Furthermore, NYESO1 TCR transgenic cell therapy trials have previously been open for enrollment by patients as young as 4 year old (Ramachandra et al., 2019). Also, genetically modified stem cells using busulfan-based conditioning have been widely reported by Dr. Don Kohn's group at UCLA (Bradford et al., 2020; Kohn et al., 2020).

## Informed Consent Procedure

### Informed Consent Form (ICF)

This study will be conducted in compliance with UCLA IRB, IBC, ISPRC, the RAC and the FDA, and relevant informed consent regulations. A written informed consent, properly witnessed and executed, will be obtained from each patient prior to clinical trial entry. The consent form will be in compliance with all applicable local, state, and federal regulations including 21 CFR Part 50, Subparts B and C, and will be approved by UCLA medical IRB2. The ICF must contain a language readily understood by the potential study subjects. Each prospective study patient must be informed of the purpose, procedures, and potential hazards and anticipated benefits of the study, as well as their right to refuse to participate or to withdraw at any time without prejudice. The original consent form, signed and dated by the patient and by the person who conducted the informed consent discussion, will be included in the patient’s UCLA medical chart and will be available for review by the FDA or other Regulatory Authorities. A copy of the signed ICF will be provided to the patient.

There will be one consent form describing all of the study procedures, including the administration of genetically modified cells and the procedures for gene transfer. The rationale for including gene transfer and non-gene transfer procedures in the same consent form is that their separation would result in unnecessary duplication of text and non-linear explanation of the study procedures.

### Procedure of Consent

Subjects will be seen by one of the study investigators who will explain the study in detail including procedures, potential risks and side effects. Subjects will be encouraged to take additional time to consider the study and will be given the opportunity to take home the ICF for review and consideration. They will then be invited to meet with one of the investigators at another time. Subjects will be encouraged to have significant others or relatives with them at the time of the interviews. Questions from subjects, significant others and relatives will be encouraged. If the subject is non-English speaking, the study investigators will have the informed consent translated in the subject’s native language. Ample time will be allowed for discussions and clarification of the goals of the research project with the trial investigators. Potential subjects will be asked if they wish to participate and told that it is voluntary and refusal will not affect their relationship with their physician or the care given to them at UCLA. No coercion will be used.

## Subject Enrollment

Subjects will be enrolled at the time the ICF is signed. Fulfillment of the eligibility criteria for each new subject enrolled will be prospectively and independently monitored by the JCCC ORC Compliance Officer.

# QUALIFICATIONS OF PERSONNEL AND STUDY FACILITIES

## Qualifications of Investigators

**The UCLA Human Gene Medicine and Melanoma Programs:** The first human gene transfer clinical trial conducted by Drs. Economou, Glaspy and McBride was initiated in 1992. This was a double retroviral vector gene marking of tumor infiltrating lymphocytes (TIL) ([Economou et al., 1996](#_ENREF_36)). Two adenoviral vector gene transfer-based clinical trials were conducted by Drs. Economou, Glaspy, McBride and Ribas at UCLA. These clinical trials involved the generation of genetically manipulated dendritic cell (DC)-based cellular vaccines using adenoviral vectors for melanoma and hepatocellular carcinoma (INDs 9908 and 11299). In more recent years, Dr. Ribas has initiated 4 gene therapy clinical trials involving genetically engineered PBMC and DC-based cellular vaccines using retroviral vectors for melanoma and sarcoma (INDs 13859 and 15167).

- **Theodore Scott Nowicki, M.D., Ph.D.** Clinical Instructor, Division of Pediatric Hematology and Oncology at UCLA. Dr. Nowicki is a translational pediatric oncologist with an interest in tumor immunotherapy for sarcomas.
- **Antoni Ribas, M.D., Ph.D.**, Professor of Medicine, Surgery, and Molecular and Medical Pharmacology, Director of the Tumor Immunology Program Area at the JCCC at UCLA. Dr. Ribas is a translational medical oncologist and tumor immunologist, conducting experimental clinical trials in patients with melanoma.
- **James S. Economou, M.D. Ph.D.**, Professor of Surgery and Microbiology, Immunology, and Molecular Genetics, Chief of the Division of Surgical Oncology and the Director of the Melanoma Program at UCLA. Dr. Economou is an accomplished translational tumor immunologist and pioneer in clinical trials with retrovirus-modified TILs.
- **John A. Glaspy, M.D., M.P.H.**, Professor of Medicine, Vice-Chair of the Division of Hematology-Oncology, Director of the JCCC Clinical Research Unit Shared Resource at UCLA, and the Associate Chief, UCLA 100 Medical Plaza Community Oncology Practice. Dr. Glaspy is a leading expert in clinical research, with a focus on melanoma and the development of cytokines and growth factors for human use.
- **Bartosz Chmielowski, M.D., Ph.D.** Assistant Professor of Medicine in the Division of Hematology-Oncology at UCLA. He is an expert in thymic development ([Chmielowski et al., 1999](#_ENREF_22); [Chmielowski et al., 2000](#_ENREF_23); [Chmielowski et al., 2002](#_ENREF_24)) and in the care of patients with malignant melanoma.
- **Noah Federman, M.D.** Associate Professor of Pediatrics, Hematology/Oncology and Director of the Pediatric Bone and Soft Tissue Sarcoma Program at UCLA. Dr. Federman is a board certified pediatric hematologist/oncologist and an expert in treating bone and soft tissue sarcomas in children Involving high dose chemotherapy, myeolablative and nonmyeloablative conditioning regimens, and allogeneic and autologous stem cell transplant.
- **Sant Chawla, M.D.** Associate Clinical Professor of Medicine in the Division of Hematology-Oncology at ULCA and the Director of the Sarcoma Oncology Clinic in Santa Monica. He is a leading expert in treating patients with sarcoma.
- **Gary Schiller, M.D.** Professor of Medicine in the Division of Hematology-Oncology and Director of the Hematological Malignancies/Stem Cell Transplantation Unit at UCLA. Dr. Schiller is a leading expert in bone marrow/stem cell transplantation for hematological malignancies.
- **Sarah Larson, M.D.** Assistant Professor of Medicine in the Division of Hematology-Oncology. Dr. Larson is an expert in bone marrow/stem cell transplantation for hematological malignancies.
- **Alistair Cochran, M.D.** Professor of Pathology and Laboratory Medicine at UCLA. Dr. Cochran has a special interest in dermatopathology.
- **Deborah Wong, M.D., Ph.D.** Assistant Professor in Medicine Division of Hematology and Oncology at UCLA.  Dr. Wong is a translational medical oncologist whose research and clinical interest is in improving the understanding and the treatment of patients with melanoma.
- **Anusha Kalbasi, M.D.** Clinical Instructor in Radiation Oncology at UCLA. Dr. Kalbasi is a radiation oncologist that specializes in the treatment of sarcoma and melanoma. He is experienced in advanced treatment approaches, including intensity-modulated radiotherapy (IMRT), stereotactic body radiotherapy (SBRT), image-guided radiotherapy, and proton therapy. Dr. Kalbasi’s research is focused on immunotherapeutic strategies for the treatment of solid malignancies.
- **Begoña Comin-Anduix, Ph.D.** Adjunct Professor, Division of Surgical Oncology at UCLA. An expert in immune monitoring and culture of human lymphocytes ([Comin-Anduix et al., 2006](#_ENREF_27)). Will lead the manufacturing and laboratory analysis of patient-derived samples.
- **Eunice M. Kim. Clinical Laboratory Technician**, Bone Marrow Stem Cell Laboratory. Will oversee the processing and storage of the unmodified stem cells in the trial.
- **Ignacio Baselga Carretero, Ph.D.** Postdoctoral Fellow, Division of Hematology-Oncology at UCLA. Experienced in coordinating clinical care and managing data for subjects on clinical trials as well as regulatory submissions. Will coordinate clinical care and manage data during the conduct of this clinical trial.
- **Cristina Puig Saus, Ph.D.** Associate Project Scientist, Study Director for the Good Laboratory Practice (GLP) safety study, Division of Hematology-Oncology at UCLA. An expert in viral vectors, preclinical adoptive cell transfer studies and IND-enabling GLP studies for adoptive cell transfer trials using lentiviral and retroviral vectors.
- **Cole W. Peters, Ph.D.** Associate Project Scientist, Division of Pediatric Hematology and Oncology at UCLA. An expert in viral vectors and IND-enabling GLP studies for adoptive cell transfer trials using lentiviral and retroviral vectors.
- **Giulia Parisi, Ph.D.** Associate Project Scientist, Division of Hematology-Oncology at UCLA. An expert in preclinical adoptive cell transfer studies and gene analysis by vector copy number.
- **Beatriz Campos, Ph.D.** Associate Project Scientist, Department of Microbiology, Immunology and Molecular Genetics at UCLA. An expert in clinical trials with genetically engineered stem cells using lentivirus vectors and gene analysis by vector copy number.
- **Donald Kohn, M.D.** Professor of Pediatrics and Microbiology, Immunology and Molecular Genetics at UCLA. Dr. Kohn is an international leader in clinical trials of HSC genetically modified using retroviral vectors ([Kohn et al., 1999](#_ENREF_57); [Kohn et al., 1998](#_ENREF_58); [Kohn et al., 2003a](#_ENREF_59); [Kohn et al., 1995](#_ENREF_61); [Schmidt et al., 2003](#_ENREF_101)).
- **Joshua Sasine, M.D.** Hematologist and Oncologist at the David Geffen School of Medicine at UCLA. He is the Medical Director of the Chimeric Antigen Receptor T-Cell (CAR-T) Program.
- **The UCLA AIDS Institute Gene Therapy Program.** Two clinical trials of HSC gene therapy in patients with HIV infection and AIDS, have been led by Dr. Zack (INDs 7328 and 10183). These trials involve introduction of an anti-HIV ribozyme into autologous CD34+ hematopoietic progenitor cells from HIV+ adults ([Amado et al., 2004](#_ENREF_4); [Amado et al., 1999](#_ENREF_5)).
- **Jerome A. Zack, Ph.D.** Professor of Medicine and Vice Chair of Microbiology, Immunology, and Molecular Genetics at UCLA. Dr. Zack is a thymocyte development, embryonic stem cells and HIV immunity expert ([Galic et al., 2006](#_ENREF_38)).
- **Zoran Galic, Ph.D.** Assistant Professor of Medicine at UCLA. Dr. Galic is an embryonic stem cell and thymocyte development expert ([Galic et al., 2006](#_ENREF_38)).
- **Lili Yang, Ph.D.**, Assistant Professor in Microbiology, Immunology and Molecular Genetics at UCLA. Dr. Yang is an expert in TCR genetic cloning and the genetic engineering of HSC ([Yang and Baltimore, 2005](#_ENREF_121); [Yang et al., 2002](#_ENREF_122)).

**The UCLA *In Vivo* Cellular and Molecular Imaging Center:**

- **Martin Allen-Auerbach, M.D.** Associate Clinical Professor of Pharmacology and Medical Director of Nuclear Medicine at UCLA. Dr. Allen-Auerbach is involved in clinical studies investigating the role of molecular imaging with PET/CT to gain insight into malignant disease processes and monitoring of cancer treatment.
- **Owen N. Witte, M.D.** Howard Hughes Medical Institute Investigator at UCLA, Professor of Microbiology, Immunology, and Molecular Genetics, President's Chair in Developmental Immunology, Professor of Molecular and Medical Pharmacology, and Director of the Institute of Stem Cell Biology and Medicine at UCLA. Dr. Witte is a recognized molecular biologist who has pioneered an approach to non-invasively image antitumor T cell responses in living animals ([Dubey et al., 2003](#_ENREF_32); [Shu et al., 2005](#_ENREF_104)).
- **Johannes Czernin, M.D.**, Professor of Molecular and Medical Pharmacology at UCLA. Dr. Czernin is a leading expert in the use of metabolic imaging for assessment of tumor response in patients ([Czernin et al., 2006](#_ENREF_29); [Weber, 2005](#_ENREF_114)).

**Biostatistics, Analytical Support & Evaluation (BASE) Unit**

- **Xiaoyan Wang, Ph.D.** Assistant Professor of Medicine at UCLA. Dr. Wang is an experienced biostatistician in gene therapy clinical trials.

In conclusion, the study investigators are very familiar with the use of retroviral and lentiviral vectors to genetically modify primary lymphocytes and hematopoietic stem cells, the requirements of GMP standards for *ex vivo* cell culture and genetic manipulation, the requirements for good clinical practices (GCP) standards for conducting human experimental clinical trials, and have fully equipped clinical facilities that have been successfully reviewed by regulatory agencies for the conduct of cellular therapy and gene transfer studies.

## Personnel Training

**a. Clinical Personnel**: The following will be required courses, training sessions or certificates required for clinical study personnel:

- UCLA IRB Human subjects certificate.
- UCLA IRB Health Insurance Portability and Accountability Act (HIPAA) certificate.
- Certificate in Good Clinical Practice.
- UCLA Human Gene and Cell Therapy Program Educational Course.

**b.** **Laboratory Personnel**: The following courses, training sessions or certificates will be required for study laboratory personnel involved in TCR transgenic cell manufacturing:

- UCLA IRB Human subjects certificate.
- UCLA IRB HIPPA certificate.
- UCLA current Good Manufacturing Practice training.
- UCLA Human Gene and Cell Therapy Program Educational Course.
- UCLA IBC compliance training.
- UCLA EH&S training on BSL-2.
- UCLA EH&S certificate in the use of biosafety cabinets.
- UCLA EH&S shipping of Biological Material.
- UCLA EH&S training in biological waste management.
- UCLA EH&S training on Bloodborne Pathogens.

## Human and Gene Cell Therapy Facility (HGCTF) at UCLA

UCLA has a dedicated, fully equipped and functional GMP suite specifically designed for cellular and gene transfer clinical trials. This facility is located on the 14^th^ floor of the JCCC Factor building and is jointly managed by the Office of the Dean of Medicine. It consists of a 2395 square feet BSL-2 suite with 7 HEPA-filtered rooms. This secured area has centralized monitoring of air and equipment linked to the study investigators’ pagers. Access is restricted to investigators with certified training. The 4 positive pressure laboratories are designed for *ex vivo* cell manipulation of cells, since it minimizes the possibility of outside pathogen contamination. The 3 negative pressure laboratories are designed for gene transfer procedures, since it ensures that the recombinant vectors will not contaminate the surrounding environment. There have been 18 investigator-initiated clinical trials in which the cell or gene therapy product has been manufactured by the investigator team in the GMP suite within RAC and FDA compliance.

## Gene Therapy Viral Bank

The clinical grade MSGV1-A2aB-1G4A-LY3H10 and LV-optNYESOTCR/TK will be stored in a dedicated –80^o^C freezer with central, computerized monitoring system, with alarm and recording of all HGCTF storage systems (refrigerators, freezers and incubators). This freezer is located at the HGCTF. This freezer is designed for exclusive use as a viral bank for ongoing gene therapy trials at UCLA, and is located in a secure area for containment to ensure proper storage. Access to the facility is guarded by two security doors with restricted access only to adequately trained and authorized individuals. Successful completion of GMP training is required to gain access to this HGCTF.

Under these conditions, retroviral and lentiviral vectors retain activity for a period exceeding 5 years if not subjected to freeze-thawing. Each aliquot of the final retroviral and lentiviral vector is calculated to be for a single use, therefore avoiding freeze-thawing cycles. All activities related to the GMP retroviral vector stored in the viral bank will be recorded in appropriate SOPs. This includes initial lot shipment, viral vector storage, viral vector dispensing, remaining samples after use to generate retrovirus and lentiviral gene-modified cell therapies, and yearly recertification.

## Clinical Facilities

Chemotherapy conditioning and NY-ESO-1 TCR cell adoptive transfer will be delivered in an isolation hospital bed in the Hematology/Oncology Hematopoietic Stem Cell ([Kohn et al.](#_ENREF_57)) transplantation ward. This is a fully staffed inpatient ward with experienced nurses, house officers, clinical consultants and attending physicians. This ensures that, in the event of an adverse reaction or any other adverse event, treatment will be promptly available. Patients will be placed in individual use rooms equipped with a HEPA filters that meet the precautions normally used for patients undergoing HSC transplantation. While receiving the TCR transgenic PBMC and PBSC adoptive transfer, patients will be continuously monitored.

The Division of Hematology and Oncology at UCLA’s outpatient clinic is located at the UCLA 100 Medical Plaza, Suite 550. This facility is staffed with 1 office manager, 3 front desk assistants, 6 nurses, 2 phlebotomists, 3 insurance specialists and 2 nurse practitioners.

The UCLA Hemapheresis Unit is located at the 6^th^ floor of the 200 Medical Plaza building, Suite 660. This unit has 5 staff nurses on a one-to-one basis, a supervisor (nurse manager), a coordinator, and a Hematology/Oncology Medical Director and attending doctors.

## Compliance with Protocol Procedures and Rights and Welfare of Study Participants

**JCCC Office of Regulatory Compliance (ORC).** The manager of this office will oversee the adequate conduct of all the JCCC DSMB required safety oversight activities including real time eligibility review, monitoring and auditing of this clinical trial.

**JCCC ORC Clinical Research Compliance Officer~~.~~** The JCCC ORC compliance officer is responsible for real-time eligibility review, monitoring and auditing of studies to ensure regulatory compliance. The compliance officer will prospectively review adherence to the protocol, RAC and FDA procedures. The Compliance Officer is also responsible for confirming that the data recorded on the CRFs is consistent with the information contained in the study source documents.

# DATA SAFETY MONITORING BOARD, STUDY OVERSIGHT, DATA COLLECTION AND ANALYSIS

## Data Safety Monitoring Board (DSMB)

The JCCC DSMB was constituted in January 2001. The DSMB membership consists of representatives of the Cancer Center clinical research community. The Director of the Cancer Center appoints members to a term of 2 years and each member is selected for their professional expertise in oncology practice and research.

The JCCC DSMB meets monthly to review all serious adverse event reports for JCCC institutional clinical trials at UCLA and those encountered in the UCLA TORI network where the JCCC DSMB has oversight. All serious adverse event reports, which have been filed since the previous meeting, are presented to the committee for review.

For all JCCC studies, where the JCCC DSMB has primary oversight for AE review, all AEs occurring within these studies shall be reported to the JCCC DSMB in a timely manner consistent with the UCLA IRB time requirements [7 days, 24 hours for a death] regardless of relationship or seriousness. The JCCC Office of Regulatory Compliance (ORC) will review all submissions and staff will enter the information into the JCCC Clinical Trials database. Reports are generated for full JCCC DSMB review of those SAEs that have some component of relatedness to the study drug and may, at the discretion of the JCCC compliance officers, include SAE reports that may be incorrectly assessed by the PI. For institutional trials, where the JCCC DSMB has primary DSMB review responsibility, the office will request that the PI generate cumulative adverse event reports for biannual or annual review.

The DSMB reviews each Serious Adverse Event reported and determines whether or not protocol modifications are warranted to ensure patient safety. In this review, prior occurrences of similar toxicity with the therapy under study are taken into consideration, as well as the seriousness of the event and the likelihood that it was related to a study drug. The DSMB may recommend no changes to the study if the event is expected or related to other causes such as the patient’s underlying condition. The DSMB may request an expert’s advice of other non-UCLA Principal Investigator with national experience to support their deliberations and decisions. The JCCC DSMB has the authority to recommend to the UCLA IRB the immediate halt to the study (i.e., discontinuation of any further treatment of enrolled patients and discontinuation of enrollment of new patients) should there be any serious unexpected toxicity that warrants further investigation. JCCC DSMB correspondences are addressed to the Principal Investigator and copied to the UCLA IRB. Minutes of the DSMB meetings are recorded and processed into the computer file.

## JCCC DSMB Internal Monitoring Plan

The NIH and NCI policy statements allow for variable monitoring and reporting plans, commensurate with the potential risks and with the size and complexity of the trial. The monitoring plan must be sufficiently rigorous and effective to ensure subject safety and to ensure protocol compliance and data validity and integrity.

### Level of Risk of a Study

For trials overseen by the JCCC DSMB, the JCCC DSMB will determine the degree of risk of the study and will ensure that there are procedures in place to ensure the safety of the subjects that are enrolled in the trial. The intensity level of the monitoring is determined by the risk category. Some of the factors that must be considered when assigning the Level of Risk category include:

1. A biostatistical design and appropriate procedures for proper data management so that the information collected can be properly validated.
2. Appropriate Serious Adverse Event reporting procedures must be in place.
3. The study duration must be appropriate and must be based on a realistic rate of enrollment.
4. Data collection and data management must be adequate to verify and ensure subject safety.

### Assignment of risk

Assigning risk ensures that the data and safety monitoring is based on the level of risk (low, medium, or high) to ensure that the data and safety monitoring activities are appropriate. Below are some of the criteria used to make a decision regarding the assignment of risk:

- Expected duration of the study based upon the estimated rate of one.
- Type of study population (e.g., children, geriatric)
- The procedures used in the trial are commensurate with the degree of risk.
- Adequate data management systems in place and appropriate case report forms
- Proper serious adverse event reporting procedures in place
- Proper biostatistical design and data analysis procedures in place.

## Monitoring/Auditing Activities

The compliance officer of the JCCC ORC will monitor the clinical records for all human subjects enrolled onto JCCC institutional trials overseen by the JCCC DSMB. The JCCC compliance officer will perform real time review of informed consent form processes and the meeting of all inclusion and exclusion criteria at study entry. Active monitoring will offer the JCCC study teams prospective information that can be used to enhance the quality of research being performed contemporaneously. Auditing is a review of historic performance of the research effort and is performed on case report forms, regulatory files and source documents to measure the quality of the research effort in a retrospective manner.

## Detailed Reporting Mechanism for Adverse Events

Each protocol is required to have a detailed description of the Adverse Event Reporting method. The DSMB expects each protocol to abide by the reporting time line and definitions consistent with the NCI and FDA guidelines.

An adverse event is any undesirable experience associated with the use of a medical product in a patient (any unfavorable and unintended sign, symptom or disease temporally associated with the use of a medical treatment or procedure regardless of whether it is considered related to the medical treatment or procedure). Any clinical adverse event must be recorded on the case report form during the course of the study. The investigator must evaluate and document the adverse event for severity, grade it according to the NCI Common Toxicity Guideline, document the causal relationship to the study drug under study, take appropriate action to care for the patient and to document the outcome. All information recorded in the case report form must be verifiably documented in the source (i.e., medical record or physician office chart).

When an adverse event occurs, it is the responsibility of the investigator to evaluate and record into the source documents, the nature of the symptom, prescribe the appropriate remedy and to report the event. The adverse event must be reported annually to the sponsor, the IRB, FDA and any other appropriate agency (e.g. ISPRC).

If the event is **Serious and Unexpected**, the event should be reported immediately (in writing within 7 days, 24 hours for death) by the Principal Investigator to the IND sponsor, UCLA IRB, JCCC DSMB and to any appropriate agency; the NIH and the UCLA IBC for gene medicine trial; directly to the FDA if it is an institutional IND trial; and to the NCI if it is an NCI sponsored trial. For institutional trials and for gene medicine trials, the JCCC ORC and Gene Medicine Compliance Officers will assist the investigator to ensure that all serious adverse events are properly documented and reported in accordance to federal and institutional requirements.

All adverse events that occur in a research study overseen by the JCCC DSMB must be submitted, regardless of relationship, expectedness or seriousness.

## Data Recording and Retention of Study Data

Data recording will be done in compliance with GCP. Medical records and notes should be clearly marked and permit easy identification of participation by an individual in the specified clinical trial. Medical records and source data should be completed within a reasonable amount of time for purposes of timely data collection and entry.

The investigators are to record all data with respect to protocol procedures, drug administration, laboratory data, safety data and efficacy ratings on the CRFs. The CRF may be a printed, optical or electronic document. All corrections on a CRF and on source documents must be made in a way that does not obscure the original entry. The correct data must be inserted, dated and initialed/authorized by study site personnel. If the change is not obvious, provide a reason.

# STATISTICAL METHODS

This is a pilot phase 1 clinical trial aimed at determining the safety and feasibility of a combined immunotherapy regimen consisting of the adoptive cell transfer of gene modified PBMC and PBSC using the MSGV1-A2aB-1G4A-LY3H10 retroviral vector and the lentiviral LV-optNYESOTCR/TK vector administered after a reduced intensity conditioning chemotherapy, followed by low dose IL-2. The assessment of safety and feasibility is based on the study of 6 subjects depending on the presence/absence of DLTs ([Section 1.5](#_Study_Objectives)).

## Sample Size Determination

This phase 1 clinical trial includes a dosing regimen of RV-NYESO TCR PBMC and LV-NYESO TCR/sr39TK PBSC (Section 1.5). A total of 6 patients will be enrolled with one cohort of 3 subjects to be enrolled initially.

- After the first 3 subjects are dosed, the cohort will be expanded to 6 subjects.
- If less than 2/6 Dose Limiting Toxicities (DLTs) are observed, the treatment will be considered safe.
- If DLTs are observed in 2 or more of 6 subjects, then the treatment will have exceeded the 33% DLT rate, and the study will be terminated.

If a subject withdraws early from participation voluntarily without a DLT or due to disease progression before the first 60 days of the DLT observation period, the subject will be substituted to allow adequate assessment of safety.

## Definition of Evaluable Patients

Patients evaluable for the primary analysis are those fully enrolled in the study after meeting the inclusion/exclusion criteria. A patient who received the gene modified PBMC and PBSC and has a baseline assessment (Day 0) of NY-ESO-1 specific T cell levels and the presence of lentiviral NY-ESO-1/TK specific PBSC and at least one on-study assessment will be considered evaluable for NY-ESO-1 specific T cell and stem cell response. Other patients will be considered un-evaluable and excluded from the primary analysis.

## Primary Endpoint Analysis

The primary endpoint of this study (defined in [Section 11.1](#_Primary_Study_Endpoint)) is the safety profile. The safety endpoint will be evaluated using halting procedures ([Section 16.5](#_Study_Stopping_and)) based on toxicities listed in Section [11.1.1](#_Definition_of_Dose). Simple descriptive statistics will be used to summarize toxicities observed after each TCR transgenic cell infusion in terms of type (organ affected or laboratory determination such as absolute neutrophil count), severity (by Toxicity Table) and nadir or maximum values for the laboratory measures, time of onset (i.e. course number), duration, and reversibility or outcome. Tables will be created to summarize these toxicities and side effects. Baseline information (e.g. the extent of prior therapy) and demographic information will be presented, as well, to describe the patients treated in this pilot study. All responses will be reported.

## Secondary and Exploratory Endpoint Analysis

The secondary endpoints of this study (defined in [Section 11.2](#_Secondary_Study_Endpoints:)) include: feasibility, transgenic cell persistence, replication competence of the retrovirus and lentivirus, insertion site analysis, immune monitoring and clinical response (efficacy). The exploratory endpoint of this study is evaluation of NY-ESO-1 TCR transgenic cell tumor trafficking (imaging/biodistribution). Descriptive statistics including simple summary measures and plots appropriate for longitudinal data will be used to analyze secondary objectives, which mainly involve PET-imaging standard uptake values (SUVs) within metastatic tumor sites and secondary lymphoid organs and serial cell counts of gene-containing cells in peripheral blood over the 4-month period following gene therapy. If appropriate, single group inferences of SUVs and counts will be made with two-sided exact confidence intervals.

## Study Stopping and Non-stopping Rules.

It is recognized that AEs can occur frequently in this population based on the underlying malignancy and these can be SAEs. The review of SAEs will form the basis for potential early stopping of the study. Only unexpected SAEs that are related to the transduction process/research reagent would define a stopping rule. The review of these adverse events, and any decision to prematurely stop subject enrollment, will be determined by the UCLA DSMB and reviewed by the IRB.

Absolute stopping rules for this clinical trial will be:

1. Any death that is possibly related to the investigational agents namely RV-NYESO TCR PBMC or LV-NYESO TCR/sr39TK PBSC.
2. Two or more grade 4 events that are possibly related to RV-NYESO TCR PBMC or LV-NYESO TCR/sr39TK PBSC.
3. Any event of hematological malignancy must be considered as study stopping criteria until oncogenesis related to retroviral mutagenesis, lentiviral mutagenesis, EBV lymphoma and Post Transplant Lymphoproliferative Disorder can be excluded.
4. Activation of the suicide gene feature of the sr39TK transgene by administration of ganciclovir
5. More than 1 engraftment failure.
6. Greater than 2 severe infections requiring transfer to the ICU.

Premature termination of the clinical trial may occur because of a regulatory authority decision, change in opinion of the FDA, RAC, IRB, the DSMB, or determination that there are problems in the cell product generation or the safety of their administration as described in the assessment of primary or secondary study endpoints*.* Additionally, recruitment may be stopped for reasons of protocol violations, or inadequate data recording.

# REFERENCES

Aiuti, A., Bacchetta, R., Seger, R., Villa, A., and Cavazzana-Calvo, M. (2012). Gene therapy for primary immunodeficiencies: Part 2. Curr Opin Immunol *24*, 585-591.

Aiuti, A., Biasco, L., Scaramuzza, S., Ferrua, F., Cicalese, M.P., Baricordi, C., Dionisio, F., Calabria, A., Giannelli, S., Castiello, M.C.*, et al.* (2013). Lentiviral hematopoietic stem cell gene therapy in patients with Wiskott-Aldrich syndrome. Science (New York, NY) *341*, 1233151.

Alyea, E.P., Kim, H.T., Ho, V., Cutler, C., DeAngelo, D.J., Stone, R., Ritz, J., Antin, J.H., and Soiffer, R.J. (2006). Impact of conditioning regimen intensity on outcome of allogeneic hematopoietic cell transplantation for advanced acute myelogenous leukemia and myelodysplastic syndrome. Biology of blood and marrow transplantation : journal of the American Society for Blood and Marrow Transplantation *12*, 1047-1055.

Amado, R.G., Mitsuyasu, R.T., Rosenblatt, J.D., Ngok, F.K., Bakker, A., Cole, S., Chorn, N., Lin, L.S., Bristol, G., Boyd, M.P.*, et al.* (2004). Anti-human immunodeficiency virus hematopoietic progenitor cell-delivered ribozyme in a phase I study: myeloid and lymphoid reconstitution in human immunodeficiency virus type-1-infected patients. Human gene therapy *15*, 251-262.

Amado, R.G., Mitsuyasu, R.T., Symonds, G., Rosenblatt, J.D., Zack, J., Sun, L.Q., Miller, M., Ely, J., and Gerlach, W. (1999). A phase I trial of autologous CD34+ hematopoietic progenitor cells transduced with an anti-HIV ribozyme. Human gene therapy *10*, 2255-2270.

Barrow, C., Browning, J., MacGregor, D., Davis, I.D., Sturrock, S., Jungbluth, A.A., and Cebon, J. (2006). Tumor antigen expression in melanoma varies according to antigen and stage. Clinical cancer research : an official journal of the American Association for Cancer Research *12*, 764-771.

Barton, K.N., Stricker, H., Brown, S.L., Elshaikh, M., Aref, I., Lu, M., Pegg, J., Zhang, Y., Karvelis, K.C., Siddiqui, F.*, et al.* (2008). Phase I study of noninvasive imaging of adenovirus-mediated gene expression in the human prostate. Molecular therapy : the journal of the American Society of Gene Therapy *16*, 1761-1769.

Bauer, M., Reaman, G.H., Hank, J.A., Cairo, M.S., Anderson, P., Blazar, B.R., Frierdich, S., and Sondel, P.M. (1995). A phase II trial of human recombinant interleukin-2 administered as a 4-day continuous infusion for children with refractory neuroblastoma, non-Hodgkin's lymphoma, sarcoma, renal cell carcinoma, and malignant melanoma. A Childrens Cancer Group study. Cancer *75*, 2959-2965.

Ben-Barouch, S., Cohen, O., Vidal, L., Avivi, I., and Ram, R. (2016). Busulfan fludarabine vs busulfan cyclophosphamide as a preparative regimen before allogeneic hematopoietic cell transplantation: systematic review and meta-analysis. Bone Marrow Transplant *51*, 232-240.

Berger, C., Flowers, M.E., Warren, E.H., and Riddell, S.R. (2006). Analysis of transgene-specific immune responses that limit the in vivo persistence of adoptively transferred HSV-TK-modified donor T cells after allogeneic hematopoietic cell transplantation. Blood *107*, 2294-2302.

Berns, A. (2004). Good news for gene therapy. N Engl J Med *350*, 1679-1680.

Biffi, A., Montini, E., Lorioli, L., Cesani, M., Fumagalli, F., Plati, T., Baldoli, C., Martino, S., Calabria, A., Canale, S.*, et al.* (2013). Lentiviral hematopoietic stem cell gene therapy benefits metachromatic leukodystrophy. Science *341*, 1233158.

Black, M.E., Newcomb, T.G., Wilson, H.M., and Loeb, L.A. (1996). Creation of drug-specific herpes simplex virus type 1 thymidine kinase mutants for gene therapy. Proceedings of the National Academy of Sciences of the United States of America *93*, 3525-3529.

Blattman, J.N., and Greenberg, P.D. (2004). Cancer immunotherapy: a treatment for the masses. Science (New York, NY) *305*, 200-205.

Bolli, M., Schultz-Thater, E., Zajac, P., Guller, U., Feder, C., Sanguedolce, F., Carafa, V., Terracciano, L., Hudolin, T., Spagnoli, G.C.*, et al.* (2005). NY-ESO-1/LAGE-1 coexpression with MAGE-A cancer/testis antigens: a tissue microarray study. Int J Cancer *115*, 960-966.

Bonini, C., Ferrari, G., Verzeletti, S., Servida, P., Zappone, E., Ruggieri, L., Ponzoni, M., Rossini, S., Mavilio, F., Traversari, C.*, et al.* (1997). HSV-TK gene transfer into donor lymphocytes for control of allogeneic graft-versus-leukemia. Science (New York, NY) *276*, 1719-1724.

Bradford, K. L., Pearl, M., Kohn, D. B., Weng, P., Yadin, O., De Oliveira, S. N., & Moore, T. B. (2020). AT1R Activating Autoantibodies in Hematopoietic Stem Cell Transplantation. Biology of Blood and Marrow Transplantation.

Brix, G., Lechel, U., Glatting, G., Ziegler, S.I., Munzing, W., Muller, S.P., and Beyer, T. (2005). Radiation exposure of patients undergoing whole-body dual-modality 18F-FDG PET/CT examinations. Journal of nuclear medicine : official publication, Society of Nuclear Medicine *46*, 608-613.

Cartier, N., Hacein-Bey-Abina, S., Bartholomae, C.C., Veres, G., Schmidt, M., Kutschera, I., Vidaud, M., Abel, U., Dal-Cortivo, L., Caccavelli, L.*, et al.* (2009). Hematopoietic stem cell gene therapy with a lentiviral vector in X-linked adrenoleukodystrophy. Science (New York, NY) *326*, 818-823.

Case, S.S., Price, M.A., Jordan, C.T., Yu, X.J., Wang, L., Bauer, G., Haas, D.L., Xu, D., Stripecke, R., Naldini, L.*, et al.* (1999). Stable transduction of quiescent CD34(+)CD38(-) human hematopoietic cells by HIV-1-based lentiviral vectors. Proceedings of the National Academy of Sciences of the United States of America *96*, 2988-2993.

Cavazzana-Calvo, M., Payen, E., Negre, O., Wang, G., Hehir, K., Fusil, F., Down, J., Denaro, M., Brady, T., Westerman, K.*, et al.* (2010). Transfusion independence and HMGA2 activation after gene therapy of human beta-thalassaemia. Nature *467*, 318-322.

Chae, Y.S., Sohn, S.K., Kim, J.G., Cho, Y.Y., Moon, J.H., Shin, H.J., Chung, J.S., Cho, G.J., Yang, D.H., Lee, J.J.*, et al.* (2007). New myeloablative conditioning regimen with fludarabine and busulfan for allogeneic stem cell transplantation: comparison with BuCy2. Bone Marrow Transplant *40*, 541-547.

Chmielowski, B., Muranski, P., and Ignatowicz, L. (1999). In the normal repertoire of CD4+ T cells, a single class II MHC/peptide complex positively selects TCRs with various antigen specificities. Journal of immunology (Baltimore, Md : 1950) *162*, 95-105.

Chmielowski, B., Muranski, P., Kisielow, P., and Ignatowicz, L. (2000). On the role of high- and low-abundance class II MHC-peptide complexes in the thymic positive selection of CD4(+) T cells. Int Immunol *12*, 67-72.

Chmielowski, B., Pacholczyk, R., Kraj, P., Kisielow, P., and Ignatowicz, L. (2002). Presentation of antagonist peptides to naive CD4+ T cells abrogates spatial reorganization of class II MHC peptide complexes on the surface of dendritic cells. Proceedings of the National Academy of Sciences of the United States of America *99*, 15012-15017.

Chodon, T., Comin-Anduix, B., Chmielowski, B., Koya, R.C., Wu, Z., Auerbach, M., Ng, C., Avramis, E., Seja, E., Villanueva, A.*, et al.* (2014). Adoptive transfer of MART-1 T-cell receptor transgenic lymphocytes and dendritic cell vaccination in patients with metastatic melanoma. Clinical cancer research : an official journal of the American Association for Cancer Research *20*, 2457-2465.

Ciceri, F., Bonini, C., Marktel, S., Zappone, E., Servida, P., Bernardi, M., Pescarollo, A., Bondanza, A., Peccatori, J., Rossini, S.*, et al.* (2007). Antitumor effects of HSV-TK-engineered donor lymphocytes after allogeneic stem-cell transplantation. Blood *109*, 4698-4707.

Comin-Anduix, B., Gualberto, A., Glaspy, J.A., Seja, E., Ontiveros, M., Reardon, D.L., Renteria, R., Englahner, B., Economou, J.S., Gomez-Navarro, J.*, et al.* (2006). Definition of an immunologic response using the major histocompatibility complex tetramer and enzyme-linked immunospot assays. Clinical cancer research : an official journal of the American Association for Cancer Research *12*, 107-116.

Cress, R.D., and Holly, E.A. (1997). Incidence of cutaneous melanoma among non-Hispanic whites, Hispanics, Asians, and blacks: an analysis of california cancer registry data, 1988-93. Cancer Causes Control *8*, 246-252.

Czernin, J., Weber, W.A., and Herschman, H.R. (2006). Molecular imaging in the development of cancer therapeutics. Annu Rev Med *57*, 99-118.

de Lima, M., Couriel, D., Thall, P.F., Wang, X., Madden, T., Jones, R., Shpall, E.J., Shahjahan, M., Pierre, B., Giralt, S.*, et al.* (2004). Once-daily intravenous busulfan and fludarabine: clinical and pharmacokinetic results of a myeloablative, reduced-toxicity conditioning regimen for allogeneic stem cell transplantation in AML and MDS. Blood *104*, 857-864.

Dembic, Z., Haas, W., Zamoyska, R., Parnes, J., Steinmetz, M., and von Boehmer, H. (1987). Transfection of the CD8 gene enhances T-cell recognition. Nature *326*, 510-511.

Dubey, P., Su, H., Adonai, N., Du, S., Rosato, A., Braun, J., Gambhir, S.S., and Witte, O.N. (2003). Quantitative imaging of the T cell antitumor response by positron-emission tomography. Proceedings of the National Academy of Sciences of the United States of America *100*, 1232-1237.

Dudley, M.E., Wunderlich, J.R., Robbins, P.F., Yang, J.C., Hwu, P., Schwartzentruber, D.J., Topalian, S.L., Sherry, R., Restifo, N.P., Hubicki, A.M.*, et al.* (2002a). Cancer regression and autoimmunity in patients after clonal repopulation with antitumor lymphocytes. Science (New York, NY) *298*, 850-854.

Dudley, M.E., Wunderlich, J.R., Yang, J.C., Hwu, P., Schwartzentruber, D.J., Topalian, S.L., Sherry, R.M., Marincola, F.M., Leitman, S.F., Seipp, C.A.*, et al.* (2002b). A phase I study of nonmyeloablative chemotherapy and adoptive transfer of autologous tumor antigen-specific T lymphocytes in patients with metastatic melanoma. J Immunother *25*, 243-251.

Dudley, M.E., Wunderlich, J.R., Yang, J.C., Sherry, R.M., Topalian, S.L., Restifo, N.P., Royal, R.E., Kammula, U., White, D.E., Mavroukakis, S.A.*, et al.* (2005). Adoptive cell transfer therapy following non-myeloablative but lymphodepleting chemotherapy for the treatment of patients with refractory metastatic melanoma. J Clin Oncol *23*, 2346-2357.

Economou, J.S., Belldegrun, A.S., Glaspy, J., Toloza, E.M., Figlin, R., Hobbs, J., Meldon, N., Kaboo, R., Tso, C.L., Miller, A.*, et al.* (1996). In vivo trafficking of adoptively transferred interleukin-2 expanded tumor-infiltrating lymphocytes and peripheral blood lymphocytes. Results of a double gene marking trial. The Journal of clinical investigation *97*, 515-521.

Freytag, S.O., Movsas, B., Aref, I., Stricker, H., Peabody, J., Pegg, J., Zhang, Y., Barton, K.N., Brown, S.L., Lu, M.*, et al.* (2007). Phase I trial of replication-competent adenovirus-mediated suicide gene therapy combined with IMRT for prostate cancer. Molecular therapy : the journal of the American Society of Gene Therapy *15*, 1016-1023.

Galic, Z., Kitchen, S.G., Kacena, A., Subramanian, A., Burke, B., Cortado, R., and Zack, J.A. (2006). T lineage differentiation from human embryonic stem cells. Proceedings of the National Academy of Sciences of the United States of America *103*, 11742-11747.

Gambhir, S.S., Barrio, J.R., Wu, L., Iyer, M., Namavari, M., Satyamurthy, N., Bauer, E., Parrish, C., MacLaren, D.C., Borghei, A.R.*, et al.* (1998). Imaging of adenoviral-directed herpes simplex virus type 1 thymidine kinase reporter gene expression in mice with radiolabeled ganciclovir. Journal of nuclear medicine : official publication, Society of Nuclear Medicine *39*, 2003-2011.

Gambhir, S.S., Bauer, E., Black, M.E., Liang, Q., Kokoris, M.S., Barrio, J.R., Iyer, M., Namavari, M., Phelps, M.E., and Herschman, H.R. (2000). A mutant herpes simplex virus type 1 thymidine kinase reporter gene shows improved sensitivity for imaging reporter gene expression with positron emission tomography. Proceedings of the National Academy of Sciences of the United States of America *97*, 2785-2790.

Giannoni, F., Hardee, C.L., Wherley, J., Gschweng, E., Senadheera, S., Kaufman, M.L., Chan, R., Bahner, I., Gersuk, V., Wang, X.*, et al.* (2013). Allelic exclusion and peripheral reconstitution by TCR transgenic T cells arising from transduced human hematopoietic stem/progenitor cells. Mol Ther *21*, 1044-1054.

Giralt, S., Khouri, I., and Champlin, R. (1999). Non myeloablative "mini transplants". Cancer Treat Res *101*, 97-108.

Gnjatic, S., Nishikawa, H., Jungbluth, A.A., Gure, A.O., Ritter, G., Jager, E., Knuth, A., Chen, Y.T., and Old, L.J. (2006). NY-ESO-1: review of an immunogenic tumor antigen. Adv Cancer Res *95*, 1-30.

Gogas, H., Ioannovich, J., Dafni, U., Stavropoulou-Giokas, C., Frangia, K., Tsoutsos, D., Panagiotou, P., Polyzos, A., Papadopoulos, O., Stratigos, A.*, et al.* (2006). Prognostic significance of autoimmunity during treatment of melanoma with interferon. N Engl J Med *354*, 709-718.

Goldstein, G., Rutenberg, T.F., Mendelovich, S.L., Hutt, D., Oikawa, M.T., Toren, A., and Bielorai, B. (2017). The role of immunoglobulin prophylaxis for prevention of cytomegalovirus infection in pediatric hematopoietic stem cell transplantation recipients. Pediatr Blood Cancer *64*.

Gschweng, E.H., McCracken, M.N., Kaufman, M.L., Ho, M., Hollis, R.P., Wang, X., Saini, N., Koya, R.C., Chodon, T., Ribas, A.*, et al.* (2014). HSV-sr39TK positron emission tomography and suicide gene elimination of human hematopoietic stem cells and their progeny in humanized mice. Cancer Res *74*, 5173-5183.

Hacein-Bey-Abina, S., Von Kalle, C., Schmidt, M., McCormack, M.P., Wulffraat, N., Leboulch, P., Lim, A., Osborne, C.S., Pawliuk, R., Morillon, E.*, et al.* (2003). LMO2-associated clonal T cell proliferation in two patients after gene therapy for SCID-X1. Science (New York, NY) *302*, 415-419.

Hunder, N.N., Wallen, H., Cao, J., Hendricks, D.W., Reilly, J.Z., Rodmyre, R., Jungbluth, A., Gnjatic, S., Thompson, J.A., and Yee, C. (2008). Treatment of metastatic melanoma with autologous CD4+ T cells against NY-ESO-1. N Engl J Med *358*, 2698-2703.

Jager, E., Chen, Y.T., Drijfhout, J.W., Karbach, J., Ringhoffer, M., Jager, D., Arand, M., Wada, H., Noguchi, Y., Stockert, E.*, et al.* (1998). Simultaneous humoral and cellular immune response against cancer-testis antigen NY-ESO-1: definition of human histocompatibility leukocyte antigen (HLA)-A2-binding peptide epitopes. J Exp Med *187*, 265-270.

Johnson, L.A., Morgan, R.A., Dudley, M.E., Cassard, L., Yang, J.C., Hughes, M.S., Kammula, U.S., Royal, R.E., Sherry, R.M., Wunderlich, J.R.*, et al.* (2009). Gene therapy with human and mouse T-cell receptors mediates cancer regression and targets normal tissues expressing cognate antigen. Blood *114*, 535-546.

June, C.H. (2007). Adoptive T cell therapy for cancer in the clinic. J Clin Invest *117*, 1466-1476.

Jungbluth, A.A., Antonescu, C.R., Busam, K.J., Iversen, K., Kolb, D., Coplan, K., Chen, Y.T., Stockert, E., Ladanyi, M., and Old, L.J. (2001). Monophasic and biphasic synovial sarcomas abundantly express cancer/testis antigen NY-ESO-1 but not MAGE-A1 or CT7. Int J Cancer *94*, 252-256.

Kennedy, D.R., McLellan, K., Moore, P.F., Henthorn, P.S., and Felsburg, P.J. (2009). Effect of ex vivo culture of CD34+ bone marrow cells on immune reconstitution of XSCID dogs following allogeneic bone marrow transplantation. Biology of blood and marrow transplantation : journal of the American Society for Blood and Marrow Transplantation *15*, 662-670.

Kessels, H.W., Wolkers, M.C., and Schumacher, T.N. (2002). Adoptive transfer of T-cell immunity. Trends Immunol *23*, 264-269.

Kim, J.W., Hung, C.F., Juang, J., He, L., Kim, T.W., Armstrong, D.K., Pai, S.I., Chen, P.J., Lin, C.T., Boyd, D.A.*, et al.* (2004a). Comparison of HPV DNA vaccines employing intracellular targeting strategies. Gene Ther *11*, 1011-1018.

Kim, Y.J., Dubey, P., Ray, P., Gambhir, S.S., and Witte, O.N. (2004b). Multimodality imaging of lymphocytic migration using lentiviral-based transduction of a tri-fusion reporter gene. Mol Imaging Biol *6*, 331-340.

Kohn, D.B., Bauer, G., Rice, C.R., Rothschild, J.C., Carbonaro, D.A., Valdez, P., Hao, Q., Zhou, C., Bahner, I., Kearns, K.*, et al.* (1999). A clinical trial of retroviral-mediated transfer of a rev-responsive element decoy gene into CD34(+) cells from the bone marrow of human immunodeficiency virus-1-infected children. Blood *94*, 368-371.

Kohn, D. B., Booth, C., Kang, E. M., Pai, S. Y., Shaw, K. L., Santilli, G., *et al.* (2020). Lentiviral gene therapy for X-linked chronic granulomatous disease. Nature Medicine, 26(2), 200-206.

Kohn, D.B., Hershfield, M.S., Carbonaro, D., Shigeoka, A., Brooks, J., Smogorzewska, E.M., Barsky, L.W., Chan, R., Burotto, F., Annett, G.*, et al.* (1998). T lymphocytes with a normal ADA gene accumulate after transplantation of transduced autologous umbilical cord blood CD34+ cells in ADA-deficient SCID neonates. Nature medicine *4*, 775-780.

Kohn, D.B., Sadelain, M., Dunbar, C., Bodine, D., Kiem, H.P., Candotti, F., Tisdale, J., Riviere, I., Blau, C.A., Richard, R.E.*, et al.* (2003a). American Society of Gene Therapy (ASGT) ad hoc subcommittee on retroviral-mediated gene transfer to hematopoietic stem cells. Molecular therapy : the journal of the American Society of Gene Therapy *8*, 180-187.

Kohn, D.B., Sadelain, M., and Glorioso, J.C. (2003b). Occurrence of leukaemia following gene therapy of X-linked SCID. Nat Rev Cancer *3*, 477-488.

Kohn, D.B., Weinberg, K.I., Nolta, J.A., Heiss, L.N., Lenarsky, C., Crooks, G.M., Hanley, M.E., Annett, G., Brooks, J.S., el-Khoureiy, A.*, et al.* (1995). Engraftment of gene-modified umbilical cord blood cells in neonates with adenosine deaminase deficiency. Nature medicine *1*, 1017-1023.

Kroger, N., Renges, H., Sonnenberg, S., Kruger, W., Gutensohn, K., Dielschneider, T., Cortes-Dericks, L., and Zander, A.R. (2002). Stem cell mobilisation with 16 microg/kg vs 10 microg/kg of G-CSF for allogeneic transplantation in healthy donors. Bone marrow transplantation *29*, 727-730.

Kwong, G.A., Radu, C.G., Hwang, K., Shu, C.J., Ma, C., Koya, R.C., Comin-Anduix, B., Hadrup, S.R., Bailey, R.C., Witte, O.N.*, et al.* (2009). Modular nucleic acid assembled p/MHC microarrays for multiplexed sorting of antigen-specific T cells. J Am Chem Soc *131*, 9695-9703.

Lee, C.C., Sui, G., Elizarov, A., Shu, C.J., Shin, Y.S., Dooley, A.N., Huang, J., Daridon, A., Wyatt, P., Stout, D.*, et al.* (2005). Multistep synthesis of a radiolabeled imaging probe using integrated microfluidics. Science (New York, NY) *310*, 1793-1796.

Li, Y., Moysey, R., Molloy, P.E., Vuidepot, A.L., Mahon, T., Baston, E., Dunn, S., Liddy, N., Jacob, J., Jakobsen, B.K.*, et al.* (2005). Directed evolution of human T-cell receptors with picomolar affinities by phage display. Nat Biotechnol *23*, 349-354.

Link, H.A., L (1996). Allogeneic Transplantation of Peripheral Blood Progenitor Cells. Annals of Oncology *7*, 41-45.

Lois, C., Refaeli, Y., Qin, X.F., and Van Parijs, L. (2001). Retroviruses as tools to study the immune system. Curr Opin Immunol *13*, 496-504.

Ma, C., Fan, R., Ahmad, H., Shi, Q., Comin-Anduix, B., Chodon, T., Koya, R.C., Liu, C.C., Kwong, G.A., Radu, C.G.*, et al.* (2011). A clinical microchip for evaluation of single immune cells reveals high functional heterogeneity in phenotypically similar T cells. Nature medicine *17*, 738-743.

Mackensen, A., Meidenbauer, N., Vogl, S., Laumer, M., Berger, J., and Andreesen, R. (2006). Phase I study of adoptive T-cell therapy using antigen-specific CD8+ T cells for the treatment of patients with metastatic melanoma. J Clin Oncol *24*, 5060-5069.

Mazurier, F., Gan, O.I., McKenzie, J.L., Doedens, M., and Dick, J.E. (2004). Lentivector-mediated clonal tracking reveals intrinsic heterogeneity in the human hematopoietic stem cell compartment and culture-induced stem cell impairment. Blood *103*, 545-552.

McCormack, M.P., and Rabbitts, T.H. (2004). Activation of the T-cell oncogene LMO2 after gene therapy for X-linked severe combined immunodeficiency. N Engl J Med *350*, 913-922.

Miyoshi, H., Smith, K.A., Mosier, D.E., Verma, I.M., and Torbett, B.E. (1999). Transduction of human CD34+ cells that mediate long-term engraftment of NOD/SCID mice by HIV vectors. Science *283*, 682-686.

Modlich, U., Bohne, J., Schmidt, M., von Kalle, C., Knoss, S., Schambach, A., and Baum, C. (2006). Cell-culture assays reveal the importance of retroviral vector design for insertional genotoxicity. Blood *108*, 2545-2553.

Modlich, U., Schambach, A., Brugman, M.H., Wicke, D.C., Knoess, S., Li, Z., Maetzig, T., Rudolph, C., Schlegelberger, B., and Baum, C. (2008). Leukemia induction after a single retroviral vector insertion in Evi1 or Prdm16. Leukemia *22*, 1519-1528.

Montini, E., Cesana, D., Schmidt, M., Sanvito, F., Ponzoni, M., Bartholomae, C., Sergi, L.S., Benedicenti, F., Ambrosi, A., Di Serio, C.*, et al.* (2006). Hematopoietic stem cell gene transfer in a tumor-prone mouse model uncovers low genotoxicity of lentiviral vector integration. Nat Biotechnol *24*, 687-696.

Morgan, R.A., Dudley, M.E., Wunderlich, J.R., Hughes, M.S., Yang, J.C., Sherry, R.M., Royal, R.E., Topalian, S.L., Kammula, U.S., Restifo, N.P.*, et al.* (2006). Cancer regression in patients after transfer of genetically engineered lymphocytes. Science (New York, NY) *314*, 126-129.

Naldini, L. (2011). Ex vivo gene transfer and correction for cell-based therapies. Nat Rev Genet *12*, 301-315.

Naldini, L., Blomer, U., Gallay, P., Ory, D., Mulligan, R., Gage, F.H., Verma, I.M., and Trono, D. (1996). In vivo gene delivery and stable transduction of nondividing cells by a lentiviral vector. Science *272*, 263-267.

Odunsi, K., Jungbluth, A.A., Stockert, E., Qian, F., Gnjatic, S., Tammela, J., Intengan, M., Beck, A., Keitz, B., Santiago, D.*, et al.* (2003). NY-ESO-1 and LAGE-1 cancer-testis antigens are potential targets for immunotherapy in epithelial ovarian cancer. Cancer Res *63*, 6076-6083.

Patterson, A.P. (2005). Minutes of the Recombinant DNA Advisory Committee - 9/21/05. <http://www4odnihgov/oba/rac/minutes/RAC_minutes_09-05pdf>.

Prins, R.M., Shu, C.J., Radu, C.G., Vo, D.D., Khan-Farooqi, H., Soto, H., Yang, M.Y., Lin, M.S., Shelly, S., Witte, O.N.*, et al.* (2008). Anti-tumor activity and trafficking of self, tumor-specific T cells against tumors located in the brain. Cancer immunology, immunotherapy : CII *57*, 1279-1289.

Raanani, P., Gafter-Gvili, A., Paul, M., Ben-Bassat, I., Leibovici, L., and Shpilberg, O. (2009). Immunoglobulin prophylaxis in hematopoietic stem cell transplantation: systematic review and meta-analysis. J Clin Oncol *27*, 770-781.

Radu, C.G., Shu, C.J., Shelly, S.M., Phelps, M.E., and Witte, O.N. (2007). Positron emission tomography with computed tomography imaging of neuroinflammation in experimental autoimmune encephalomyelitis. Proceedings of the National Academy of Sciences of the United States of America *104*, 1937-1942.

Ramachandran, I., Lowther, D. E., Dryer-Minnerly, R., Wang, R., Fayngerts, S., Nunez, D., *et al.* (2019). Systemic and local immunity following adoptive transfer of NY-ESO-1 SPEAR T cells in synovial sarcoma. Journal for immunotherapy of cancer, 7(1), 1-14.

Rambaldi, A., Grassi, A., Masciulli, A., Boschini, C., Mico, M.C., Busca, A., Bruno, B., Cavattoni, I., Santarone, S., Raimondi, R.*, et al.* (2015). Busulfan plus cyclophosphamide versus busulfan plus fludarabine as a preparative regimen for allogeneic haemopoietic stem-cell transplantation in patients with acute myeloid leukaemia: an open-label, multicentre, randomised, phase 3 trial. Lancet Oncol *16*, 1525-1536.

Rapoport, A.P., Stadtmauer, E.A., Binder-Scholl, G.K., Goloubeva, O., Vogl, D.T., Lacey, S.F., Badros, A.Z., Garfall, A., Weiss, B., Finklestein, J.*, et al.* (2015). NY-ESO-1-specific TCR-engineered T cells mediate sustained antigen-specific antitumor effects in myeloma. Nat Med *21*, 914-921.

Ribas, A. (2006). Update on immunotherapy for melanoma. J Natl Compr Canc Netw *4*, 687-694.

Ribas, A., Timmerman, J.M., Butterfield, L.H., and Economou, J.S. (2003). Determinant spreading and tumor responses after peptide-based cancer immunotherapy. Trends Immunol *24*, 58-61.

Robbins, P.F., Kassim, S.H., Tran, T.L., Crystal, J.S., Morgan, R.A., Feldman, S.A., Yang, J.C., Dudley, M.E., Wunderlich, J.R., Sherry, R.M.*, et al.* (2015). A pilot trial using lymphocytes genetically engineered with an NY-ESO-1-reactive T-cell receptor: long-term follow-up and correlates with response. Clinical cancer research : an official journal of the American Association for Cancer Research *21*, 1019-1027.

Robbins, P.F., Li, Y.F., El-Gamil, M., Zhao, Y., Wargo, J.A., Zheng, Z., Xu, H., Morgan, R.A., Feldman, S.A., Johnson, L.A.*, et al.* (2008). Single and dual amino acid substitutions in TCR CDRs can enhance antigen-specific T cell functions. Journal of immunology (Baltimore, Md : 1950) *180*, 6116-6131.

Robbins, P.F., Morgan, R.A., Feldman, S.A., Yang, J.C., Sherry, R.M., Dudley, M.E., Wunderlich, J.R., Nahvi, A.V., Helman, L.J., Mackall, C.L.*, et al.* (2011a). Tumor regression in patients with metastatic synovial cell sarcoma and melanoma using genetically engineered lymphocytes reactive with NY-ESO-1. Journal of clinical oncology : official journal of the American Society of Clinical Oncology *29*, 917-924.

Robbins, P.F., Morgan, R.A., Feldman, S.A., Yang, J.C., Sherry, R.M., Dudley, M.E., Wunderlich, J.R., Nahvi, A.V., Helman, L.J., Mackall, C.L.*, et al.* (2011b). Tumor regression in patients with metastatic synovial cell sarcoma and melanoma using genetically engineered lymphocytes reactive with NY-ESO-1. J Clin Oncol *29*, 917-924.

Rohdewohld, H., Weiher, H., Reik, W., Jaenisch, R., and Breindl, M. (1987). Retrovirus integration and chromatin structure: Moloney murine leukemia proviral integration sites map near DNase I-hypersensitive sites. Journal of virology *61*, 336-343.

Roper, M., Smith, M.A., Sondel, P.M., Gillespie, A., Reaman, G.H., Hammond, G.D., Levitt, D., Rosolen, A., Colamonici, O.R., Neckers, L.M.*, et al.* (1992). A phase I study of interleukin-2 in children with cancer. Am J Pediatr Hematol Oncol *14*, 305-311.

Rosenberg, S.A., Yang, J.C., and Restifo, N.P. (2004). Cancer immunotherapy: moving beyond current vaccines. Nature medicine *10*, 909-915.

Ruelle, J., Yfantis, V., Duquenne, A., and Goubau, P. (2014). Validation of an ultrasensitive digital droplet PCR assay for HIV-2 plasma RNA quantification. Journal of the International AIDS Society *17*, 19675.

Russell, J.A., Tran, H.T., Quinlan, D., Chaudhry, A., Duggan, P., Brown, C., Stewart, D., Ruether, J.D., Morris, D., Glick, S.*, et al.* (2002). Once-daily intravenous busulfan given with fludarabine as conditioning for allogeneic stem cell transplantation: study of pharmacokinetics and early clinical outcomes. Biology of blood and marrow transplantation : journal of the American Society for Blood and Marrow Transplantation *8*, 468-476.

Ryu, S.G., Lee, J.H., Choi, S.J., Lee, J.H., Lee, Y.S., Seol, M., Hur, E.H., Lee, S.H., Bae, K.S., Noh, G.J.*, et al.* (2007). Randomized comparison of four-times-daily versus once-daily intravenous busulfan in conditioning therapy for hematopoietic cell transplantation. Biology of blood and marrow transplantation : journal of the American Society for Blood and Marrow Transplantation *13*, 1095-1105.

Satie, A.P., Rajpert-De Meyts, E., Spagnoli, G.C., Henno, S., Olivo, L., Jacobsen, G.K., Rioux-Leclercq, N., Jegou, B., and Samson, M. (2002). The cancer-testis gene, NY-ESO-1, is expressed in normal fetal and adult testes and in spermatocytic seminomas and testicular carcinoma in situ. Lab Invest *82*, 775-780.

Sato, M., Kako, S., Matsumoto, K., Oshima, K., Akahoshi, Y., Nakano, H., Ugai, T., Yamasaki, R., Wada, H., Ishihara, Y.*, et al.* (2015). Pharmacokinetics study of once-daily intravenous busulfan in conditioning regimens for hematopoietic stem cell transplantation. International journal of hematology *101*, 497-504.

Sato, S., Noguchi, Y., Wada, H., Fujita, S., Nakamura, S., Tanaka, R., Nakada, T., Hasegawa, K., Nakagawa, K., Koizumi, F.*, et al.* (2005). Quantitative real-time RT-PCR analysis of NY-ESO-1 and LAGE-1a mRNA expression in normal tissues and tumors, and correlation of the protein expression with the mRNA copy number. Int J Oncol *26*, 57-63.

Schmidt, M., Carbonaro, D.A., Speckmann, C., Wissler, M., Bohnsack, J., Elder, M., Aronow, B.J., Nolta, J.A., Kohn, D.B., and von Kalle, C. (2003). Clonality analysis after retroviral-mediated gene transfer to CD34+ cells from the cord blood of ADA-deficient SCID neonates. Nature medicine *9*, 463-468.

Schumacher, T.N. (2002). T-cell-receptor gene therapy. Nat Rev Immunol *2*, 512-519.

Seymour, L., Bogaerts, J., Perrone, A., Ford, R., Schwartz, L.H., Mandrekar, S., Lin, N.U., Litiere, S., Dancey, J., Chen, A.*, et al.* (2017). iRECIST: guidelines for response criteria for use in trials testing immunotherapeutics. Lancet Oncol *18*, e143-e152.

Shu, C.J., Guo, S., Kim, Y.J., Shelly, S.M., Nijagal, A., Ray, P., Gambhir, S.S., Radu, C.G., and Witte, O.N. (2005). Visualization of a primary anti-tumor immune response by positron emission tomography. Proceedings of the National Academy of Sciences of the United States of America *102*, 17412-17417.

Shu, C.J., Radu, C.G., Shelly, S.M., Vo, D.D., Prins, R., Ribas, A., Phelps, M.E., and Witte, O.N. (2009). Quantitative PET reporter gene imaging of CD8+ T cells specific for a melanoma-expressed self-antigen. Int Immunol *21*, 155-165.

Singh AS, C.B., Berent-Maoz B, Comin-Anduix B, Hu-Lieskovan S, Federman N, Kaplan-Lefko P, Huang RR, Seja E, Chodon T, Koya R, Eilber FC, Glaspy JA and Ribas A (2015). Adoptive Cellular Therapy with Autologous Lymphocytes Genetically Modified to express an NY-ESO-1 Specific T-Cell Receptor and Dendritic Cell Vaccination with or without Ipilimumab in Patients with Advanced Sarcomas and Melanoma. Paper presented at: Connective Tissue Oncology Society (CTOS) Annual Meeting (Salt Lake City, Utah ).

Suzuki, T., Shen, H., Akagi, K., Morse, H.C., Malley, J.D., Naiman, D.Q., Jenkins, N.A., and Copeland, N.G. (2002). New genes involved in cancer identified by retroviral tagging. Nat Genet *32*, 166-174.

Therasse, P., Arbuck, S.G., Eisenhauer, E.A., Wanders, J., Kaplan, R.S., Rubinstein, L., Verweij, J., Van Glabbeke, M., van Oosterom, A.T., Christian, M.C.*, et al.* (2000). New guidelines to evaluate the response to treatment in solid tumors [see comments]. J Natl Cancer Inst *92*, 205-216.

Tiberghien, P., Ferrand, C., Lioure, B., Milpied, N., Angonin, R., Deconinck, E., Certoux, J.M., Robinet, E., Saas, P., Petracca, B.*, et al.* (2001). Administration of herpes simplex-thymidine kinase-expressing donor T cells with a T-cell-depleted allogeneic marrow graft. Blood *97*, 63-72.

Tsai, J., Lee, J.T., Wang, W., Zhang, J., Cho, H., Mamo, S., Bremer, R., Gillette, S., Kong, J., Haass, N.K.*, et al.* (2008). Discovery of a selective inhibitor of oncogenic B-Raf kinase with potent antimelanoma activity. Proc Natl Acad Sci U S A *105*, 3041-3046.

Vatakis, D.N., Koya, R.C., Nixon, C.C., Wei, L., Kim, S.G., Avancena, P., Bristol, G., Baltimore, D., Kohn, D.B., Ribas, A.*, et al.* (2011). Antitumor activity from antigen-specific CD8 T cells generated in vivo from genetically engineered human hematopoietic stem cells. Proceedings of the National Academy of Sciences of the United States of America *108*, E1408-1416.

Verzeletti, S., Bonini, C., Marktel, S., Nobili, N., Ciceri, F., Traversari, C., and Bordignon, C. (1998). Herpes simplex virus thymidine kinase gene transfer for controlled graft-versus-host disease and graft-versus-leukemia: clinical follow-up and improved new vectors. Human gene therapy *9*, 2243-2251.

Wargo, J.A., Robbins, P.F., Li, Y., Zhao, Y., El-Gamil, M., Caragacianu, D., Zheng, Z., Hong, J.A., Downey, S., Schrump, D.S.*, et al.* (2009). Recognition of NY-ESO-1+ tumor cells by engineered lymphocytes is enhanced by improved vector design and epigenetic modulation of tumor antigen expression. Cancer immunology, immunotherapy : CII *58*, 383-394.

Weber, W.A. (2005). Use of PET for monitoring cancer therapy and for predicting outcome. Journal of nuclear medicine : official publication, Society of Nuclear Medicine *46*, 983-995.

Wu, X., Li, Y., Crise, B., and Burgess, S.M. (2003). Transcription start regions in the human genome are favored targets for MLV integration. Science (New York, NY) *300*, 1749-1751.

Xhaard, A., Rzepecki, P., Valcarcel, D., Santarone, S., Furst, S., Serrano, D., De Angelis, G., Kruger, W., and Scheid, C. (2014). Optimization of health-care organization and perceived improvement of patient comfort by switching from intra-venous BU four-times-daily infusions to a once-daily administration scheme in adult hematopoietic stem cell recipients. Bone marrow transplantation *49*, 509-512.

Yaghoubi, S., Barrio, J.R., Dahlbom, M., Iyer, M., Namavari, M., Satyamurthy, N., Goldman, R., Herschman, H.R., Phelps, M.E., and Gambhir, S.S. (2001). Human pharmacokinetic and dosimetry studies of [(18)F]FHBG: a reporter probe for imaging herpes simplex virus type-1 thymidine kinase reporter gene expression. Journal of nuclear medicine : official publication, Society of Nuclear Medicine *42*, 1225-1234.

Yaghoubi, S.S., Barrio, J.R., Namavari, M., Satyamurthy, N., Phelps, M.E., Herschman, H.R., and Gambhir, S.S. (2005). Imaging progress of herpes simplex virus type 1 thymidine kinase suicide gene therapy in living subjects with positron emission tomography. Cancer Gene Ther *12*, 329-339.

Yaghoubi, S.S., and Gambhir, S.S. (2006). Measuring herpes simplex virus thymidine kinase reporter gene expression in vitro. Nat Protoc *1*, 2137-2142.

Yaghoubi, S.S., Jensen, M.C., Satyamurthy, N., Budhiraja, S., Paik, D., Czernin, J., and Gambhir, S.S. (2009). Noninvasive detection of therapeutic cytolytic T cells with 18F-FHBG PET in a patient with glioma. Nat Clin Pract Oncol *6*, 53-58.

Yang, L., and Baltimore, D. (2005). Long-term in vivo provision of antigen-specific T cell immunity by programming hematopoietic stem cells. Proceedings of the National Academy of Sciences of the United States of America *102*, 4518-4523.

Yang, L., Qin, X.F., Baltimore, D., and Van Parijs, L. (2002). Generation of functional antigen-specific T cells in defined genetic backgrounds by retrovirus-mediated expression of TCR cDNAs in hematopoietic precursor cells. Proceedings of the National Academy of Sciences of the United States of America *99*, 6204-6209.

Yee, C., Thompson, J.A., Byrd, D., Riddell, S.R., Roche, P., Celis, E., and Greenberg, P.D. (2002). Adoptive T cell therapy using antigen-specific CD8+ T cell clones for the treatment of patients with metastatic melanoma: in vivo persistence, migration, and antitumor effect of transferred T cells. Proc Natl Acad Sci U S A *99*, 16168-16173.

Zufferey, R., Dull, T., Mandel, R.J., Bukovsky, A., Quiroz, D., Naldini, L., and Trono, D. (1998). Self-inactivating lentivirus vector for safe and efficient in vivo gene delivery. J Virol *72*, 9873-9880.

Zufferey, R., Nagy, D., Mandel, R.J., Naldini, L., and Trono, D. (1997). Multiply attenuated lentiviral vector achieves efficient gene delivery in vivo. Nature biotechnology *15*, 871-875.

Zychlinski, D., Schambach, A., Modlich, U., Maetzig, T., Meyer, J., Grassman, E., Mishra, A., and Baum, C. (2008). Physiological promoters reduce the genotoxic risk of integrating gene vectors. Mol Ther *16*, 718-725.

**Summary of Amendments**

**Amendment 1**

**Date: February 22, 2017**

Protocol

- Added Anusha Kalbasi
- Section 1.3: Clarified that clinical response will be evaluated at restaging exams, not only on Day 60.
- Section 1.5: Clarified enrollment intervals.
- Figure 3: Changed PBMC Collection to Research Blood Collection in include cytokine analysis.
- Section 1.7: Removed Day 45 blood collection and clarified what to do in the event of greater than 50% transduction efficiency.
- Section 2: Updated table
- Section 8.1: Added blood collection at baseline for cytokines and immune monitoring.
- Section 8.5.1: Added premedications for busulfan.
- Section 8.7: Clarified that blood at Day 120 can be obtained through leukapheresis.

Informed Consent

- Added Anusha Kalbasi
- Removed PICI as funding source

**Amendment 2**

**Date: April 22, 2017**

Protocol

- Removed Sarah Larson
- Modified the trial design from a dose escalation study to a single dose of each cell product with an expansion cohort (Section 1, 11.1.1).
- Modified the conditioning regimen from cyclophosphamide, fludarabine and either busulfan or total body irradiation to busulfan and fludarabine only and described as reduced intensity conditioning (Section 1, 2, 8.5.2, 9).
- Added a rationale for the modified chemotherapy conditioning regimen (Section 1.2).
- Added explanation of sr39TK mutation (Section 1.2).
- Modified the study duration (Section 1.6).
- Modified the description of collection of the mobilized leukapheresis product to indicate that cells for back up will be collected prior to collecting cells for transduction (Section 1.7, 8.3.1, 10.2).
- Added statement to indicate that unused unmodified stem cells will be disposed of after 2 years (Section 1.7, 8.3).
- Added statement to indicate that bag will be disposed of in biohazard waste in the hospital after infusion (Section 8.6).
- Modified cell product naming convention (Section 1.7).
- Modified the background for the primary objective to align with updated trial design (Section 4.1.1).
- Updated our experience from our previous NYESO TCR PBMC trial (Section 5.10).
- Clarified that subjects 16-17 years of age will only be enrolled after the therapy has been shown to be safe in 3 subjects (Section 7.1, 13.6).
- Removed the exclusion criteria regarding 3 prior myelotoxic treatment regimens (Section 7.2).
- Updated premedication for chemotherapy (Section 8. 5.1).
- Added section to clarify administration of back up cells for marrow recovery failure (Section 8.16, 9.7).
- Updated description of gene-modified PBSC (Section 9.4.2).
- Added details regarding PK assessment for busulfan dose and administration (Section 9.8.2).
- Removed fungal stain for cell products (Section 10.1.9).
- Updated manufacture procedures and lot release criteria for gene-modified PBSC (Section 10).
- Modified annual recertification criteria for lentiviral vector (Section 10.2.6).
- Removed definition of MTD (Section 11.1.1).
- Clarified DLT definitions (Section 11.1.1.1).
- Revised sample size determination (Section 16.1).

Informed Consent

- Removed Sarah Larson
- Modified study design from dose escalation to single dose of each cell product
- Modified the description of collection of the mobilized leukapheresis product to indicate that cells for back up will be collected prior to collecting cells for transduction
- Added statement to indicate that unused unmodified stem cells will be disposed of after 2 years
- Clarified information regarding administration of mobilization medications
- Modified chemotherapy conditioning regimen and modified risks accordingly
- Clarified potential risks

**Amendment 3**

**Date: May 23, 2017**

Protocol (Modifications made per FDA)

- Modified inclusion criteria language to specify that subjects must have exhausted or must be ineligible for all current available treatment options (Sections 1.4 and 7.1).
- Modified DLT criteria per FDA guidance (Section 11.1.1.1).
- Added statement to indicate that all AEs and SAEs will be reported to FDA according to 21 CFR 312.32 (Sections 11.1.1 and 12.8).

Informed Consent

- No changes were made to the ICF.

**Amendment 4**

**Date: May 25, 2017**

Protocol (Modifications made per FDA)

- Modified the protocol to indicate that if the VCN is <0.1, then the PBSC will not be administered (Section 1.2 and 8.6.2).
- Corrected title to delete the extra -1 in NY-ESO-1.
- Modified the duration of stability of the gene-modified PBSC from 24 hours post thaw to 6 hours post thaw per recent stability data (Section 9.4.2).
- Added Sarah Larson.
- Added FHBG IND #.

Informed Consent

- Added Sarah Larson

**Amendment 5**

**Date: August 30, 2017**

Protocol

- Added RAC and NCT numbers
- Modified the chemotherapy regimen schedule. Busulfan will start on Day -5 at 10PM and go through the morning of Day -2. Pre-busulfan hydration was changed from 12 hours to 4 hours. Fludarabine will be administered from Day -4 to Day -3.
- Clarified that back up cells that are not used within 2 years are de-identified and used for research and that cells from patients who screen fail will be de-identified and used for research if not used within 1 year.
- Clarified that PBSC collection is up to mDay 8. If cells are collected in a shorter period of time, no more collections are required.
- Added screening tests required for transplant clearance.
- Clarified that a biosimilar can be used instead of Neupogen.

Informed Consent

- Clarified that cells will be de-identified and used for research if not required.
- Modified chemotherapy schedule.
- Added screening tests.
- Clarified that PBSC collection is up to mDay 8.

**Amendment 6**

**Date: January 24, 2018**

Protocol

- Clarified timing of collection of CD34+ cells for backup and transduction (Section 1.7, 8.3.1, 10.2.1 and 10.2.2)
- Clarified process for dilution of cells if transduction efficiency is greater than 50% (Section 1.7, 8.3.2)
- Clarified eligibility criteria regarding size of lesions (Section 7.1)
- Screening Tests. Incorporated re-evaluation of key eligibility criteria if conditioning chemotherapy is scheduled greater than 3 months after the completion of the baseline screening assessments. Also added that if patients require bridging therapy, they will need to have completed it and have a repeat disease evaluation within 30 days prior initiation of conditioning chemotherapy administration (Section 8.1).
- Clarified monitoring of patients following LV-NYESO TCR/sr39TK PBSC and RV-NYESO TCR PBMC Infusion (Section 8.6.4)
- Added that in the highly unlikely event that pancytopenia develops following administration of ganciclovir, a bone marrow biopsy will be performed, and if absence of precursor cells, back up cells will be administered (Section 8.15).
- Updated the annual recertification data for the retroviral vector (Section 10.1.5)
- Added the lot release data for the second batch of lentiviral vector that is available for use (Section 10.2.5)
- Modified the DLT definitions per the FDA’s recommendations for NYSCTMM (Section 11.1.1.1)
- Modified study stopping rules per the FDA’s recommendations for NYSCTMM (Section 16.5)
- Updated name of manufacturing facility

Informed Consent

- Clarified timing of collection of cells
- Clarified that key screening tests will be repeated if greater than 3 months since screening completed prior to initiation of chemotherapy
- Added risk factor to MRI scan for gadolinium retention in brain tissue

**Amendment 7**

**Date: October 8, 2018**

Protocol

- Change of Principal Investigator (PI) and co-PI: Current PI Antoni Ribas, M.D., Ph.D. New PI Theodore Scott Nowicki M.D., Ph.D. Current co-PI Siwen Hu-Lieskovan, M.D., Ph.D. New co-PI Antoni Ribas, M.D., Ph.D.
- Added Joshua Sasine, M.D.
- Removed Arun Singh, M.D.
- Removed Siwen Hu-Lieskovan, M.D., Ph.D.
- Only one cohort will be included in the study, with a maximum of 6 patients (Sections 1.5, 11.1, 11.4, 16 and Table 1)
- Study duration prolonged to 36 months (Section 1.6 Study Duration)
- Clarified that the 2^nd^ leukapheresis (unmobilized) is partial (Section 1.7, 8.3 and 8.4, Table 3)
- Indicated that FHBG scans can be performed on days 25 +/- 10 and 120 +/- 30 (Section 2 (Table 2), Section 11.3.1)
- Updated annual recertification table (2018) for lentiviral vector (Section 10.2.6).
- Clarified that premeds will be administered before transgenic cell infusion (Sections 8.6.2 and 8.6.3)
- Corrected units for Busulfan conditioning. The total AUC that can be reached in 3 days is 70,000-80,000 ng/ml x hr. The targeted AUC after a single dose would be 12,000 – 13,000 ng/ml x hr
- Removed particularly low recruitment as a reason to stop recruitment (Section 16.5)
- Removed mycoplasma rapid test criteria as it is a secondary test in case the MycoAlert test is positive (Table 7a). This information is already provided as a footnote.
- Removed lot release results for LV-NYESO TCR/sr39TK Lot #111015L12 since the vector has been used up (Section 10.2.5). The annual recertification results for the previous lot have been removed.

Informed Consent

- Change of Principal Investigator (PI) and co-PI: Current PI Antoni Ribas, M.D., Ph.D. New PI Theodore Scott Nowicki M.D., Ph.D. Current co-PI Siwen Hu-Lieskovan, M.D., Ph.D. New co-PI Antoni Ribas, M.D., Ph.D.
- Added Joshua Sasine, M.D.
- Removed Arun Singh, M.D.
- Removed Siwen Hu-Lieskovan, M.D., Ph.D.

**Amendment 8**

**Date: January 9, 2019**

Protocol

- Removed Daniel Shin, M.D., Ph.D.
- Modified fludarabine regimen from 2 days at 30mg/m^2^/dose to 4 days at 40mg/m^2^/dose
- Modified G-CSF to allow for a range of 8-10µg/kg
- Modified the CTCAE version from 4 to 5

Informed Consent

- Removed Daniel Shin, M.D., Ph.D.
- Modified fludarabine regimen from 2 days to 4 days

**Amendment 9**

**Date: February 21, 2019**

Protocol

- Added clinical response per iRECIST as an exploratory endpoint (Sections 1.3, 4.3.2 and 11.3.2)
- Added viral prophylaxis (Section 8.6.4)
- Added plan in case of sub-optimal PBMC TCR transgene expression (Section 10.1.9)
- Added that prolonged cytopenia, infections, and autoimmune AEs will be identified as AEs of special interest (Section 11.1.1, 12.1 and 12.8)

Informed Consent

- No change

**Amendment 10**

**Date: June 6, 2019**

Protocol

- Changed the MOI for the lentiviral transduction from 50 TU/cell to 25 TU/cell (Section 10.2.7)
- Updated chemotherapy regimen in Figure 1
- Added Ignacio Baselga
- Removed Justin Tran

Informed Consent

- No change

**Amendment 11**

**Date: July 3, 2019**

Protocol

- Added Arun Singh
- Removed Beata Berent-Maoz and Paula Cabrera

Informed Consent

- Added Arun Singh

**Amendment 12/13***

**Date: November 4, 2019**

Protocol

- Added Sant Chawla
- Changed the timing of staggering enrollment of subjects on study (Section 1.5)
- Clarified timing of plerixafor administration (Section 1.7, 8.3.1, 9.5.5, 10.2.1)
- Modified collection of mobilized cells for transduction from a maximum of 2 days if stored at room temperature to 3 days if stored at 4°C (Section 1.7, 8.3.1, 10.2.1, 10.2.2)
- Clarified the order of collection of mobilized cells for transduction vs backup (Section 2)
- Clarified that the number of CD34-enriched cells will not be available until after the stem cell mobilization and therefore will not be included in the initial verification of eligibility (Section 7.2)
- Clarified that bone marrow involvement and liver metastases for eligibility will be based on prior scans (Section 7.2, 8.2)
- Added that a CD34 count from peripheral blood may be collected to assess mobilization (Section 8.3.1)
- Clarified the timing of the start of chemotherapy administration (Section 8.3)
- Updated the annual recertification data for the retroviral vector (Section 10.1.5)
- Added that retroviral transduction can occur in 6-well plates or in bags (Section 1.7, 10.1.6)
- Modified the final acceptance criteria (post administration) for TCR transgene functionality for RV-NYESO TCR PBMC from >10,000 pg/ml/million cells of IFNγ production to >600 pg/ml/million cells (Section 10.1.9)
- Added the annual recertification data for the lentiviral vector (Section 10.2.6)

Informed Consent

- Added Sant Chawla at the Sarcoma Oncology Center in Santa Monica

*This amendment is 12/13, because we were planning to send 2 separate amendments, one to add Sant Chawla and another to include all of the manufacturing changes. To expedite the amendment process, we sent Am 13 with all of the changes to the FDA and planned to send Am 12 to add Sant Chawla to the IRB. We were advised, however, to include all of the changes in 1 amendment, therefore, it became 12/13 since it was already sent as 13 to the FDA.

**Amendment 15**

**Date: March 11, 2020**

Protocol

- Removed Paula Kaplan-Lefko
- Removed Giulia Parisi
- Removed Barbara Nowicki
- Added blood collection points for Day 45, 75 and 120 (Section 2 and 8.7)
- Updated follow-up visits from Day 45 and 75 clinic visits can take place at patient’s primary physician’s clinic, where they can check vital signs, CBC, CMP and LDH values (Section 8.1).

Informed Consent

- Updated follow-up visits from Day 45 and 75 clinic visits can take place at patient’s primary physician’s clinic, where they can check vital signs, CBC, CMP and LDH values (Section 8.1).

**Amendment 16**

**Date: April 28, 2020**

Protocol

- Updated schedule of events virus prophylaxis and viral detection(Section 2)
- Updated Virus Prophylaxis section adding clarifications in order to detect CMV, EBV and adenovirus (Section 8.6)

Informed Consent

- Added Viral Prophylaxis explanation

**Amendment 17**

**Date: June 25, 2020**

Protocol

- Eligibility criteria reduced to greater or equal to 10 years old
- Clarification on the pregnancy/breast feeding exclusion criteria
- Added pediatrics measurements, values and drug levels in Section 8.5 and 8.6
- Clarification of Busulfan levels on Section 8.5.2 and 9.5.2,
- Clarification on parameters to held IL-2 administration (Section 8.7)
- Clarification on the supportive Care During Low Dose IL-2 Administration (Section 8.7)
- IL-2 administration changed to IV instead of SC (Section 8.7 and 9.5.1)
- Clarification on children eligibility (Section 13.6)
- Removed Paula Kaplan-Lefko from Section 14.1

Informed Consent

- n/a

**Amendment 18**

**Date: June 23, 2021**

Protocol

- Adding Cole Peters to the personnel
- Including LentiBOOST instead of retronectin cell transduction enhancer for Lentiviral transduction of PBSCs

Informed Consent

- n/a

**Amendment 19**

**Date: May 13, 2022**

Protocol

- Change from MOI 25 TU/cell to MOI 15 TU/cell. (Section 10.2.7)
- Replace Plasmalyte-A/DMSO (Cryoserv) (5%)/Pentastarch (6%)/HSA (5%) with CryoStor® CS5 as the freezing medium for cryopreservation. (Sections 9.2.2 and 10.2.8)
- Add automated cell counting and keep manual cell counting (trypan blue exclusion). Automated cell counting will be done using Nucleocounter NC200 machine, cells are dyed with Acridine Orange (AO) and 4’,6-diamidino-2-phenylindole (DAPI). (Section 10.2.10, Table 12a)

Informed Consent

- n/a
