# Supplementary material for: Human cancer-targeted immunity via transgenic hematopoietic stem cell progeny
Source: Nat Commun. 2025 Jul 1;16:5599. doi: 10.1038/s41467-025-60816-z (PMC12219382; doi:10.1038/s41467-025-60816-z)
Supplement: Supplementary file 6 — Supplementary Data 4 [file 41467_2025_60816_MOESM6_ESM.docx]

### Supplementary Data 4. Retrovirus (pMSGV1_LTR_to_LTR ) pMSGV1-retro nucleotide sequence

>pMSGV1_LTR_to_LTR

tgaaagaccccacctgtaggtttggcaagctagcttaagtaacgccattttgcaaggcatggaaaatacataactgagaatagagaagttcagatcaaggttaggaacagagagacagcagaatatgggccaaacaggatatctgtggtaagcagttcctgccccggctcagggccaagaacagatggtccccagatgcggtcccgccctcagcagtttctagagaaccatcagatgtttccagggtgccccaaggacctgaaaatgaccctgtgccttatttgaactaaccaatcagttcgcttctcgcttctgttcgcgcgcttctgctccccgagctcaataaaagagcccacaacccctcactcggcgcgccagtcctccgatagactgcgtcgcccgggtacccgtattcccaataaagcctcttgctgtttgcatccgaatcgtggactcgctgatccttgggagggtctcctcagattgattgactgcccacctcgggggtctttcatttggaggttccaccgagatttggagacccctgccCagggaccaccgacccccccgccgggaggtaagctggccagcggtcgtttcgtgtctgtctctgtctttgtgcgtgtttgtgccggcatctaatgtttgcgcctgcgtctgtactagttagctaactagctctgtatctggcggacccgtggtggaactgacgagttcggaacacccggccgcaaccctgggagacgtcccagggacttcgggggccgtttttgtggcccgacctgagtccTaaaatcccgatcgtttAggactctttggtgcaccccccttagaggagggatatgtggttctggtaggagacgagaacctaaaacagttcccgcctccgtctgaatttttgctttcggtttgggaccgaagccgcgccgcgcgtcttgtctgctgcagcatcgttctgtgttgtctctgtctgactgtgtttctgtatttgtctgaAaatatgggcccgggctagcctgttaccactcccttaagtttgaccttaggtcactggaaagatgtcgagcggatcgctcacaaccagtcggtagatgtcaagaagagacgttgggttaccttctgctctgcagaatggccaacctttaacgtcggatggccgcgagacggcacctttaaccgagacctcatcacccaggttaagatcaaggtcttttcacctggcccgcatggacacccagaccaggtcccctacatcgtgacctgggaagccttggcttttgacccccctccctgggtcaagccctttgtacaccctaagcctccgcctcctcttcctccatccgccccgtctctcccccttgaacctcctcgttcgaccccgcctcgatcctccctttatccagccctcactccttctctaggcgcccccatatggccatatgagatcttatatggggcacccccgccccttgtaaacttccctgaccctgacatgacaagagttactaacagcccctctctccaagctcacttacaggctctctacttagtccagcacgaagtctggagacctctggcggcagcctaccaagaacaactggaccgaccggtggtacctcacccttaccgagtcggcgacacagtgtgggtccgccgacaccagactaagaacctagaacctcgctggaaaggaccttacacagtcctgctgaccacccccaccgccctcaaagtagacggcatcgcagcttggatacacgccgcccacgtgaaggctgccgaccccgggggtggaccatcctctagaccgccatggagaccctcttgggcctgcttatcctttggctgcagctgcaatgggtgagcagcaaacaggaggtgacgcagattcctgcagctctgagtgtcccagaaggagaaaacttggttctcaactgcagtttcactgatagcgctatttacaacctccagtggtttaggcaggaccctgggaaaggtctcacatctctgttgcttattcagtcaagtcagagagagcaaacaagtggaagacttaatgcctcgctggataaatcatcaggacgtagtactttatacattgcagcttctcagcctggtgactcagccacctacctctgtgctgtgaggcccctgtacggaggaagctacatacctacatttggaagaggaaccagccttattgttcatccgtatatccagaaccctgaccctgccgtgtaccagctgagagactctaaatccagtgacaagtctgtctgcctattcaccgattttgattctcaaacaaatgtgtcacaaagtaaggattctgatgtgtatatcacagacaaaactgtgctagacatgaggtctatggacttcaagagcaacagtgctgtggcctggagcaacaaatctgactttgcatgtgcaaacgccttcaacaacagcattattccagaagacaccttcttccccagcccagaaagttcctgtgatgtcaagctggtcgagaaaagctttgaaacagatacgaacctaaactttcaaaacctgtcagtgattgggttccgaatcctcctcctgaaagtggccgggtttaatctgctcatgacgctgcggctgtggtccagccgggccaagcggtccggatccggagccaccaacttcagcctgctgaagcaggccggcgacgtggaggagaaccccggccccatgagcatcggcctcctgtgctgtgcagccttgtctctcctgtgggcaggtccagtgaatgctggtgtcactcagaccccaaaattccaggtcctgaagacaggacagagcatgacactgcagtgtgcccaggatatgaaccatgaatacatgtcctggtatcgacaagacccaggcatggggctgaggctgattcattactcagttgGTGCtggtatcactgaccaaggagaagtccccaatggctacaatgtctccagatcaaccacagaggatttcccgctcaggctgctgtcggctgctccctcccagacatctgtgtacttctgtgccagcagttacgtcgggaacaccggggagctgttttttggagaaggctctaggctgaccgtactggaggacctgaaaaacgtgttcccacccgaggtcgctgtgtttgagccatcagaagcagagatctcccacacccaaaaggccacactggtatgcctggccacaggcttctaccccgaccacgtggagctgagctggtgggtgaatgggaaggaggtgcacagtggggtcagcacagacccgcagcccctcaaggagcagcccgccctcaatgactccagatactgcctgagcagccgcctgagggtctcggccaccttctggcagaacccccgcaaccacttccgctgtcaagtccagttctacgggctctcggagaatgacgagtggacccaggatagggccaaacccgtcacccagatcgtcagcgccgaggcctggggtagagcagactgtggcttcacctccgagtcttaccagcaaggggtcctgtctgccaccatcctctatgagatcttgctagggaaggccaccttgtatgccgtgctggtcagtgccctcgtgctgatggctatggtcaagagaaaggattccagaggctagaattctgcagtcgacggtaccgcgggcccgggatccgataaaataaaagattttatttagtctccagaaaaaggggggaatgaaagaccccacctgtaggtttggcaagctagcttaagtaacgccattttgcaaggcatggaaaatacataactgagaatagagaagttcagatcaaggttaggaacagagagacagcagaatatgggccaaacaggatatctgtggtaagcagttcctgccccggctcagggccaagaacagatggtccccagatgcggtcccgccctcagcagtttctagagaaccatcagatgtttccagggtgccccaaggacctgaaaatgaccctgtgccttatttgaactaaccaatcagttcgcttctcgcttctgttcgcgcgcttctgctccccgagctcaataaaagagcccacaacccctcactcggcgcgccagtcctccgatagactgcgtcgcccgggtacccgtgtatccaataaaccctcttgcagttgcatccgacttgtggtctcgctgttccttgggagggtctcctctgagtgattgactacccgtcagcgggggtctttca
