# Supplementary material for: Human cancer-targeted immunity via transgenic hematopoietic stem cell progeny
Source: Nat Commun. 2025 Jul 1;16:5599. doi: 10.1038/s41467-025-60816-z (PMC12219382; doi:10.1038/s41467-025-60816-z)
Supplement: Supplementary file 9 — Reporting Summary [file 41467_2025_60816_MOESM9_ESM.pdf]

nature portfolio

Corresponding author(s): Theodore S. Nowicki, MD, PhD

Last updated by author(s): Apr 24, 2025

Reporting Summary

Nature Portfolio wishes to improve the reproducibility of the work that we publish. This form provides structure for consistency and transparency in reporting. For further information on Nature Portfolio policies, see our Editorial Policies and the Editorial Policy Checklist.

Statistics

For all statistical analyses, confirm that the following items are present in the figure legend, table legend, main text, or Methods section.

n/a	Confirmed	
☐	☒	The exact sample size (*n*) for each experimental group/condition, given as a discrete number and unit of measurement
☒	☐	A statement on whether measurements were taken from distinct samples or whether the same sample was measured repeatedly
☐	☒	The statistical test(s) used AND whether they are one- or two-sided<br>*Only common tests should be described solely by name; describe more complex techniques in the Methods section.*
☐	☒	A description of all covariates tested
☐	☒	A description of any assumptions or corrections, such as tests of normality and adjustment for multiple comparisons
☐	☒	A full description of the statistical parameters including central tendency (e.g. means) or other basic estimates (e.g. regression coefficient) AND variation (e.g. standard deviation) or associated estimates of uncertainty (e.g. confidence intervals)
☐	☒	For null hypothesis testing, the test statistic (e.g. *F*, *t*, *r*) with confidence intervals, effect sizes, degrees of freedom and *P* value noted<br>*Give P values as exact values whenever suitable.*
☒	☐	For Bayesian analysis, information on the choice of priors and Markov chain Monte Carlo settings
☐	☒	For hierarchical and complex designs, identification of the appropriate level for tests and full reporting of outcomes
☒	☐	Estimates of effect sizes (e.g. Cohen's *d*, Pearson's *r*), indicating how they were calculated

Our web collection on statistics for biologists contains articles on many of the points above.

Software and code

Policy information about availability of computer code

Data collection	Chromium Next GEM Single Nuclei Multiome ATAC + Gene Expression Library & Gel Bead Kit from 10x Genomics was used according to manufacturer’s protocol (CG000338 Rev F). Briefly, following nuclei isolation, transposition was performed, nuclei were counted using trypan blue, around 20,000 total nuclei were loaded, and nuclei and barcoded beads were isolated in oil droplets on the Chromium Controller instrument. Otherwise, no software for data collection were used.
Data analysis	Cell Ranger Arc (v2.0.2) from 10x Genomics (with Count functionality) was used for aligning reads to the human genome reference (GRCh38) with the addition of the lentivirus and retrovirus plasmids. The reference file was downloaded from the 10x Genomics website (https://support.10xgenomics.com/single-cell-gene-expression/software/downloads/latest). The custom sequences were added following 10x Genomics build a custom reference for Cell Ranger Arc (mkref) (https://support.10xgenomics.com/single-cell-multiome-atac-gex/software/pipelines/latest/tutorial/mkref). The parameters used with Count functionality include --localcores=16 --localmem=96. For the sequenced PBMC nuclei, Seurat v.5.0.155 was used for all analyses. The Azimuth Reference for Human PBMC app, a reference-based mapping pipeline led by the New York Genome Center Mapping Component as part of the NIH Human Biomolecular Atlas Project (HuBMAP) was used to generate cell typing for the multiome data.

For manuscripts utilizing custom algorithms or software that are central to the research but not yet described in published literature, software must be made available to editors and reviewers. We strongly encourage code deposition in a community repository (e.g. GitHub). See the Nature Portfolio guidelines for submitting code & software for further information.

Data

Policy information about availability of data

All manuscripts must include a data availability statement. This statement should provide the following information, where applicable:

- Accession codes, unique identifiers, or web links for publicly available datasets
- A description of any restrictions on data availability
- For clinical datasets or third party data, please ensure that the statement adheres to our policy

Raw sequencing data are available via dbGaP (accession number phs003898.v1). Source data for other figures are provided as a Source Data file. All other data (PET/CT images) are available upon request from the corresponding author only due to patient confidentiality laws.

Research involving human participants, their data, or biological material

Policy information about studies with human participants or human data. See also policy information about sex, gender (identity/presentation), and sexual orientation and race, ethnicity and racism.

Reporting on sex and gender	Sex and gender are reported for the patients treated, however the small number of patients (2 male, 1 female) preclude any significant analyses regarding sex and gender for this study.
Reporting on race, ethnicity, or other socially relevant groupings	Full demography for the patients treated, including race/ethnicity, are reported, however the small number of patients preclude any significant analyses regarding race or ethnicity.
Population characteristics	Full demography for the patients treated, including race/ethnicity, are reported, however the small number of patients preclude any significant analyses these.
Recruitment	Patients
Ethics oversight	UCLA IRB, UCLA DSMB

Note that full information on the approval of the study protocol must also be provided in the manuscript.

Field-specific reporting

Please select the one below that is the best fit for your research. If you are not sure, read the appropriate sections before making your selection.

☒ Life sciences ☐ Behavioural & social sciences ☐ Ecological, evolutionary & environmental sciences

For a reference copy of the document with all sections, see nature.com/documents/nr-reporting-summary-flat.pdf

Life sciences study design

All studies must disclose on these points even when the disclosure is negative.

Sample size	Sample size calculation was not performed due to this being a phase 1 non-randomized study. For all translational experiments on patient materials, we used as many replicates as was feasible given the limitations in human specimen material available.
Data exclusions	No data exclusions.
Replication	Replication not relevant or feasible (given that this was a phase 1 non-randomized study).
Randomization	Randomization not relevant (phase 1 non-randomized study).
Blinding	Blinding not relevant (phase 1 non-randomized study).

Reporting for specific materials, systems and methods

We require information from authors about some types of materials, experimental systems and methods used in many studies. Here, indicate whether each material, system or method listed is relevant to your study. If you are not sure if a list item applies to your research, read the appropriate section before selecting a response.

Materials & experimental systems

n/a	Involved in the study
☒	☐ Antibodies
☐	☒ Eukaryotic cell lines
☒	☐ Palaeontology and archaeology
☒	☐ Animals and other organisms
☐	☒ Clinical data
☒	☐ Dual use research of concern
☒	☐ Plants

Methods

n/a	Involved in the study
☒	☐ ChIP-seq
☐	☒ Flow cytometry
☒	☐ MRI-based neuroimaging

Eukaryotic cell lines

Policy information about cell lines and Sex and Gender in Research

Cell line source(s)	M257 cells were isolated and generated by the Antoni Ribas as previously described (Sondergaard et al J Trans Med 2010). Cells are from a male patient.
Authentication	Identity of these cells will be verified by flow cytometry in our laboratory, as well as genotyping of the cells against archival data obtained from isolation.
Mycoplasma contamination	Cell lines were tested annually for mycoplasma contamination via MycoALERT kit.
Commonly misidentified lines (See ICLAC register)	NA

Clinical data

Policy information about clinical studies

All manuscripts should comply with the ICMJE guidelines for publication of clinical research and a completed CONSORT checklist must be included with all submissions.

Clinical trial registration	NCT03240861
Study protocol	Full protocol is attached as a supplemental document
Data collection	Patients were non-randomly recruited, treated, and followed at UCLA between Jan 2018 and June 2022
Outcomes	The objective clinical response rate was assessed on day +60 scans and recorded following modified Response Evaluation Criteria in Solid Tumors (RECIST1.1).

Plants

Seed stocks	NA
Novel plant genotypes	NA
Authentication	NA

Flow Cytometry

Plots

Confirm that:

☒ The axis labels state the marker and fluorochrome used (e.g. CD4-FITC).

☒ The axis scales are clearly visible. Include numbers along axes only for bottom left plot of group (a 'group' is an analysis of identical markers).

☒ All plots are contour plots with outliers or pseudocolor plots.

☒ A numerical value for number of cells or percentage (with statistics) is provided.

Methodology

Sample preparation	Samples were cryopreserved peripheral blood mononuclear cells, and were processed as previously described. Peripheral blood mononuclear cells (PBMC) were obtained by Ficoll-Hypaque (Amersham Pharmacia, Piscataway, NJ) centrifugation of whole blood or the leukapheresis product. PBMCs were cryopreserved in liquid nitrogen in RPMI (Life Technologies Bethesda Research Laboratories, Gaithersburg, MD) supplemented with 20% (all as v/v) heat-inactivated AB human serum (OmegaSci., Inc., Tarzana, CA) and 10% DMSO (Sigma, St. Louis, MO). PBMCs were cryopreserved at a concentration of 5 × 106/mL.
Instrument	Thermo Attune
Software	FlowJo
Cell population abundance	As previously described in our previously published methods paper, Comin-Anduix Clinical Cancer Research 2006.
Gating strategy	As previously described in our previously published methods paper, Comin-Anduix Clinical Cancer Research 2006.

☒ Tick this box to confirm that a figure exemplifying the gating strategy is provided in the Supplementary Information.
